# Dynamic enhancement of drug product labels to support drug safety, efficacy, and effectiveness

**DOI:** 10.1186/2041-1480-4-5

**Published:** 2013-01-26

**Authors:** Richard D Boyce, John R Horn, Oktie Hassanzadeh, Anita de Waard, Jodi Schneider, Joanne S Luciano, Majid Rastegar-Mojarad, Maria Liakata

**Affiliations:** 1 Department of Biomedical Informatics, University of Pittsburgh, Offices at Baum, 5607 Baum Blvd, Pittsburgh, PA, USA; 2 Department of Pharmacy, University of Washington, Seattle, WA, USA; 3 IBM T.J. Watson Research Center, Yorktown Heights, NY, USA; 4 Elsevier Labs, Jericho, VT, USA; 5 Digital Enterprise Research Institute, National University of Ireland, Galway, Ireland; 6 Tetherless World Constellation, Rensselaer Polytechnic Institute, Troy, NY, USA; 7 University of Wisconsin, Milwaukee, WI, USA; 8 Department of Computer Science, Aberystwyth University, Wales, UK; 9 Text mining group, EBI-EMBL, Hinxton, Cambridge, UK

**Keywords:** Regulatory science, Drug information services, Drug labeling, Linked data, Scientific discourse ontologies, Drug interactions, Pharmacokinetics, Treatment efficacy, Treatment effectiveness, Comparative effectiveness research

## Abstract

Out-of-date or incomplete drug product labeling information may increase the risk of otherwise preventable adverse drug events. In recognition of these concerns, the United States Federal Drug Administration (FDA) requires drug product labels to include specific information. Unfortunately, several studies have found that drug product labeling fails to keep current with the scientific literature. We present a novel approach to addressing this issue. The primary goal of this novel approach is to better meet the information needs of persons who consult the drug product label for information on a drug’s efficacy, effectiveness, and safety. Using FDA product label regulations as a guide, the approach links drug claims present in drug information sources available on the Semantic Web with specific product label sections. Here we report on pilot work that establishes the baseline performance characteristics of a proof-of-concept system implementing the novel approach. Claims from three drug information sources were linked to the *Clinical Studies*, *Drug Interactions*, and *Clinical Pharmacology* sections of the labels for drug products that contain one of 29 psychotropic drugs. The resulting Linked Data set maps 409 efficacy/effectiveness study results, 784 drug-drug interactions, and 112 metabolic pathway assertions derived from three clinically-oriented drug information sources (ClinicalTrials.gov, the National Drug File – Reference Terminology, and the Drug Interaction Knowledge Base) to the sections of 1,102 product labels. Proof-of-concept web pages were created for all 1,102 drug product labels that demonstrate one possible approach to presenting information that dynamically enhances drug product labeling. We found that approximately one in five efficacy/effectiveness claims were relevant to the *Clinical Studies* section of a psychotropic drug product, with most relevant claims providing new information. We also identified several cases where all of the drug-drug interaction claims linked to the *Drug Interactions* section for a drug were potentially novel. The baseline performance characteristics of the proof-of-concept will enable further technical and user-centered research on robust methods for scaling the approach to the many thousands of product labels currently on the market.

## Introduction

The drug product label (also called “package insert”) is a major source of information intended to help clinicians prescribe drugs in a safe and effective manner. Out-of-date or incomplete product label information may increase the risk of otherwise preventable adverse drug events (ADEs). This is because many prescribers and pharmacists refer to drug product labeling for information that can help them make safe prescribing decisions [1,2]. A prescribing decision might be negatively affected if the label fails to provide information that is needed for safe dosing, or to properly manage (or avoid) the co-prescribing of drugs known to interact. Prescribing decision-making might also be indirectly affected if 1) the clinician depends on third-party drug information sources, and 2) these sources fail to add information that is available in the scientific literature but not present in the product label.

In recognition of these concerns, the US Federal Drug Administration (FDA) Code of Federal Regulations (CFR) Title 21 Part 201 Section 57 requires drug labels to include specific information for FDA-approved drugs [3]. Mandated information includes clinical studies that support a drug’s efficacy for its approved indications, known pharmacokinetic properties, clearance data for special populations, and known clinically-relevant drug-drug interactions. Unfortunately, for each of these types of information, product labeling fails to keep current with the scientific literature. For example: 

• Marroum and Gobburu noted deficiencies in the pharmacokinetic information provided by product labels, especially for drugs approved in the 1980s [1],

• Boyce *et al.* found that the product label provided quantitative data on age-related clearance reductions for only four of the 13 antidepressants for which such data was available [4],

• Steinmetz *et al.* found that quantitative information on clearance changes in the elderly was present in only 8% of 50 product inserts that they analyzed, [5], and

• Hines *et al.* noted drug-drug interaction information deficiencies in 15% of the product labels for drugs that interact with the narrow therapeutic range drug warfarin [6].

We present a novel approach to addressing product labeling information limitations such as those listed above. The primary goal of this novel approach is to better meet the information needs of persons who consult the drug product label for information on a drug’s efficacy, effectiveness, and safety. The approach is based on the hypothesis that a computable representation of the drug effectiveness and safety claims present in product labels and other high quality sources will enable novel methods for drug information retrieval that do a better job of helping drug experts, clinicians, and patients find complete and current drug information than current search engines and bibliographic databases.

**Figure 1:**
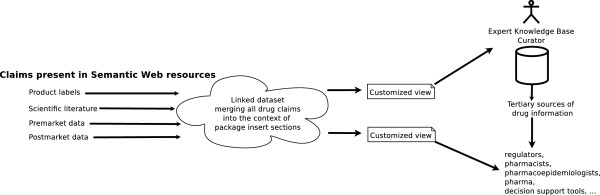
The general architecture of a system to provide dynamically enhanced views of drug product labeling using Semantic Web technologies.

Figure 1 is an overview of the system that we envision. Claims about drugs are currently present in sources of drug information such as the drug product label, studies and experiments published in the scientific literature, premarket studies and experiments reported in FDA approval documents, and post-market data sources such as drug effectiveness reviews and drug information databases. Many of these sources are available, or are becoming available, on the Semantic Web. Using FDA product label regulations as a guide [3], a new linked data set would be created that links claims present in drug information sources available on the Semantic Web to relevant product label sections. The linked data set would create and automatically update claim-evidence networks [7-11] to make transparent the motivation behind specific claims. Customized views of the linked dataset would be created for drug experts including clinicians, researchers, and persons who maintain tertiary drug information resources (i.e., proprietary drug information products).

The objective of this paper is to report on our pilot work that establishes the feasibility of the novel approach and the baseline performance characteristics of a proof-of-concept system. Because there is a broad range of content written into product labels, and the novel approach requires synthesizing research from multiple areas of research, we have organized this paper to report progress in three complementary areas: 

1. *Linking relevant Semantic Web resources to the product label:* We describe a basic proof-of-concept that demonstrates the Semantic Web technologies and Linked Data principles [12,13] that we think are necessary components of a full-scale system. The proof-of-concept consists of a set of web pages created using existing Semantic Web datasets, and demonstrates one possible approach to presenting information that dynamically enhances particular product label sections.

2. *First steps towards the automated extraction of drug efficacy and effectiveness claims:* Focusing on drug efficacy and effectiveness studies registered with ClinicalTrials.gov, we describe the methods and baseline performance characteristics of a pilot pipeline that automatically obtains claims from the scientific literature and links it to the *Clinical Studies* section of the product label for psychotropic drugs.

3. *A descriptive summary of challenges to the automated claim extraction of metabolic pathways:* We provide a descriptive analysis of the challenges to the automated identification of claims about a drug’s metabolic pathways in full text scientific articles. The analysis is based on manual identification of these claims for a single psychotropic drug.

## Results

### Linking relevant semantic web resources to the product label

**Figure 2:**
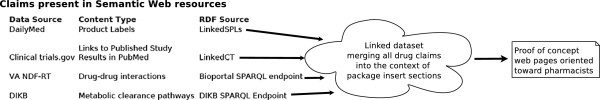
The architecture of the proof-of-concept system described in this paper that demonstrates the dynamic enhancement of drug product labels using Semantic Web technologies.

Twenty-nine active ingredients used in psychotropic drug products (i.e., antipsychotics, antidepressants, and sedative/hypnotics) that were marketed in the United States at the time of this study were selected as the target for the proof-of-concept.^a^ These drugs were chosen because they are very widely prescribed and a number of these “newer” psychotropic drugs are involved in drug-drug interactions [14]. Figure 2 shows the architecture of the proof-of-concept system that we developed for these drugs. As the figure shows, four data sources were used in the proof-of-concept. One of the sources (DailyMed) contained the text content of the three product label sections that were the focus of this study (Clinical Studies, Drug Interactions, and Clinical Pharmacology). The other three sources were chosen because they contain rigorous scientific claims that we expected to be relevant to pharmacists seeking information about the efficacy, effectiveness, and safety of a drug. These three resources, and the claims they provided, were: 

1. LinkedCT:^b^ Drug efficacy and effectiveness studies registered with ClinicalTrials.gov that have published results (as indicated by an article indexed in PubMed) [15,16]

2. National Drug File – Reference Terminology (NDF-RT):^c^ Drug-drug interactions listed as critical or significant in the Veteran’s Administration [17,18]

3. The Drug Interaction Knowledge Base (DIKB):^d^ Pharmacokinetic properties observed in pharmacokinetic studies involving humans [19].

In order for the proof-of-concept to link claims from these three sources to sections from the product labels for the chosen drugs, we first implemented a Linked Data representation of all product labels for the psychotropic drugs used in our study. We constructed the Linked Data set from the Structured Product Labels (SPLs) available in the National Library of Medicine’s DailyMed resource.^e^ A total of 36,344 unique SPLs were transformed into an RDF graph and loaded into an RDF store that provides a SPARQL endpoint.^f^ We refer to this resource as “LinkedSPLs” throughout the remainder of this text. LinkedSPLs contained product labels for all 29 psychotropic drugs in this study.

We then created a separate RDF graph with mappings between product label sections and claims present in the three drug information sources. This graph was imported it into the same RDF store as LinkedSPLs. The graph has a total of 209,698 triples and maps 409 efficacy/effectiveness study results, 784 NDF-RT drug-drug interactions, and 112 DIKB pathway claims to the sections of 1,102 product labels.^g^ Considering mappings on a label-by-label basis (see Listing Listing 1 The total number of “claim” mappings present in the proof-of-concept RDF graph by drug product label), the graph has an average of 50 mappings per product label (mean:50, median:50). Twenty-four labels had the fewest number of mappings (2), and two had greatest number of mappings (135). Table 1 shows the counts for all mappings grouped by each drug in the study. The next three sections provide more detail on the specific mappings created for each product label section.

**Table 1:** Counts of product labels and all linked claims

**Drug**	**Number of product labels**	**Number of VANDFRT**		**Number of DIKB inhibits/substrate**		**ClinicalTrials.gov**	**Published results**
	**for products containing**	**DDIs found for**		**of assertions with evidence**		**studies involving the**	**from ClinicalTrials.gov**
	**the drug**	**the drug**		**found for the drug**		**drug**	**studies involving the drug**
		Significant	Critical	Evidence for	Evidence against		
*Antidepressants*							
Amitriptyline	57	16	8	0	0	1	1
Amoxapine	2	15	8	0	0	0	0
Bupropion	111	7	4	2	0	5	44
Citalopram	85	25	9	2*	4*	4	25
Desipramine	15	16	10	0	0	0	0
Doxepin	32	15	9	0	0	0	0
Duloxetine	17	26	8	3	4	4	4
Escitalopram	20	13	3	4*	5*	6	9
Fluoxetine	90	51	14	2	0	8	22
Imipramine	19	18	10	0	0	1	4
Mirtazapine	55	2	5	4	9	1	22
Nefazodone	5	39	20	3	6	0	0
Nortriptyline	29	16	11	0	0	3	24
Paroxetine	60	33	11	2	0	3	40
Selegiline	11	2	47	0	0	1	1
Sertraline	74	28	8	2	0	3	27
Tranylcypromine	2	3	61	0	0	3	71
Trazodone	38	8	10	1	0	2	2
Trimipramine	2	17	10	0	0	0	0
Venlafaxine	66	21	6	3	3	2	2
*Antipsychotics*							
Aripiprazole	15	4	0	2	13	3	3
Clozapine	9	29	2	3	1	3	9
Olanzapine	42	0	1	1	0	5	13
Quetiapine	33	8	0	1	9	4	9
Risperidone	71	13	0	2	1	23	70
Ziprasidone	22	54	23	2*	9*	1	6
*Sedative Hypnotics*							
Eszopiclone	11	7	0	1	7	1	1
Zaleplon	24	0	0	1	1	0	0
Zolpidem	85	0	0	2	0	0	0

^*^Citalopram, escitalopram, and ziprasidone were each mapped to one claim for which there was both supporting and refuting evidence in the DIKB. Counts of product labels for each drug and claims that were linked to drug product labeling from three Linked Data drug information sources.

### Listing 1 The total number of “claim” mappings present in the proof-of-concept RDF graph by drug product label

PREFIX poc: <http://purl.org/net/nlprepository/dynamic-spl-enhancement-poc#>

SELECT ?spl COUNT(DISTINCT ?mapping) WHERE {

{

## mappings for the Clinical Studies section ##

poc:linkedct-result-map ?spl ?mapping.

?mapping poc:linkedct-result-drug ?drug.

} UNION {

## mappings for the Drug Interactions section ##

poc:ndfrt-ddi-map ?spl ?mapping.

?mapping poc:ndfrt-ddi-drug ?drug.

} UNION {

## mappings for the Clinical Pharmacology section ##

poc:dikb-pk-map ?spl ?mapping.

?mapping poc:dikb-pk-drug ?drug.

}}

GROUP BY ?spl

ORDER BY ?spl

#### Automatic linking of study abstracts from ClinicalTrials.gov to the Clinical Studies section

The *Clinical Studies* section of the product label could be mapped to the abstract of at least one published result for 22 of the 29 psychotropic drugs (76%) (see Table 1). Seven drugs (24%) were not mapped to any published result. The largest number of mappings was for risperidone, with 70 published results mapped to 71 product labels. There was a considerable difference between the mean and median number of published results that were mapped when such a mapping was possible (mean: 19, median: 9).

#### Automatic linking of VA NDF-RT drug-drug interactions to the Drug Interactions section

The *Drug Interactions* section of the product label could be mapped to at least one NDF-RT drug-drug interaction for 27 of the 29 psychotropic drugs (93%). Table 1 shows the counts for all published result mappings for each drug in the study. The number of mappings to drug-drug interactions labeled “Significant” in the NDF-RT (see Section “Methods” for explanation) ranged from 2 (mirtazapine and selegiline) to as many as 54 (ziprasidone) with a mean of 19 and a median of 16. For “Critical” drug-drug interactions, the number of mappings ranged from one (olanzapine) to 61 (tranylcypromine) with a mean of 13 and median of 9.

Table 2 shows the counts and proportion of linked drug-drug interaction claims that were noted as *potentially novel* to the Drug Interaction section of at least one *antidepressant* product label. For these drugs, a potentially novel interaction was an NDF-RT interaction that 1) was not mentioned in the Drug Interaction section of a product label based on a case-insensitive string match, and 2) was not listed as an interacting drug based on our review (prior to the study) of a single manually-reviewed product label for the listed drug (see Section “Methods” for further details). At least one potentially novel interaction was linked to a product label for products containing each of the 20 antidepressants. The largest number of potentially novel “Significant” interactions was for nefazodone and fluoxetine (31 and 28 respectively), while tranylcypromine and selegiline had the largest number of potentially novel “Critical” interactions (33 and 23 respectively). All of the “Significant” drug interactions mapped to seven antidepressants (35%) were novel, while all of the “Critical” interactions mapped to five antidepressants (25%) were novel. These results are exploratory and it is not known how many of the potentially novel interactions are truly novel.

**Table 2:** Counts of potentially novel drug-drug interaction claims

**Drug**	**Number of VA-NDFRT DDIs**		**Number of VA-NDFRT DDIs that were potentially novel**	
	**found for the drug**		**to at least one product label. N (%)**	
	**Significant**	**Critical**	**Significant**	**Critical**
Amitriptyline	16	8	8(50)	3 (38)
Amoxapine	15	8	11 (73)	4 (50)
Bupropion	7	4	5 (71)	3 (75)
Citalopram	25	9	5 (20)	4 (44)
Desipramine	16	10	16 (100)	6 (60)
Doxepin	15	9	15 (100)	9 (100)
Duloxetine	26	8	12 (46)	3 (38)
Escitalopram	13	3	3 (23)	1 (33)
Fluoxetine	51	14	28 (55)	8 (57)
Imipramine	18	10	18 (100)	6 (60)
Mirtazapine	2	5	1 (50)	1 (20)
Nefazodone	39	20	31 (80)	11 (55)
Nortriptyline	16	11	16 (100)	11 (100)
Paroxetine	33	11	15 (46)	5 (45
Selegiline	2	47	1 (50)	23 (49)
Sertraline	28	8	7 (25)	3 (38)
Tranylcypromine	3	61	1 (33)	33 (54)
Trazodone	8	10	8 (100)	10 (100)
Trimipramine	17	10	17 (100)	10 (100)
Venlafaxine	21	6	21 (100)	6 (100)

The number and proportion of VA NDF-RT drug-drug interactions that were noted as *potentially novel* to the Drug Interaction section of at least one antidepressant product label. For these drugs, a potentially novel interaction was an NDF-RT interaction that was 1) not mentioned in the Drug Interaction section of a drug’s product label based on a case-insensitive string match, and 2) not listed as an interacting drug based on our review (prior to the study) of a single manually-reviewed product label the listed drug.

#### Automatic linking of metabolic pathways claims from the drug interaction knowledge base to the Clinical Pharmacology section

The *Clinical Pharmacology* section of the product label could be mapped to at least one metabolic pathway claim for 20 of the 29 psychotropic drugs (69%). Table 1 shows the counts for all pathway mappings for every drug in the study stratified by whether the DIKB provided supporting or refuting evidence for the mapped claim. Thirteen of the 20 drugs that were mapped to pathway claims with supporting evidence were also mapped to claims with refuting evidence. In most cases, these mappings were to different pathway claims, as only three drugs (citalopram, escitalopram, and ziprasidone) were mapped to individual claims with both supporting and refuting evidence. Three pathway claims had both supporting and refuting evidence, 40 pathway claims had only supporting evidence, and 69 claims had only refuting evidence.

#### Generation of web page mashups

**Figure 3:**
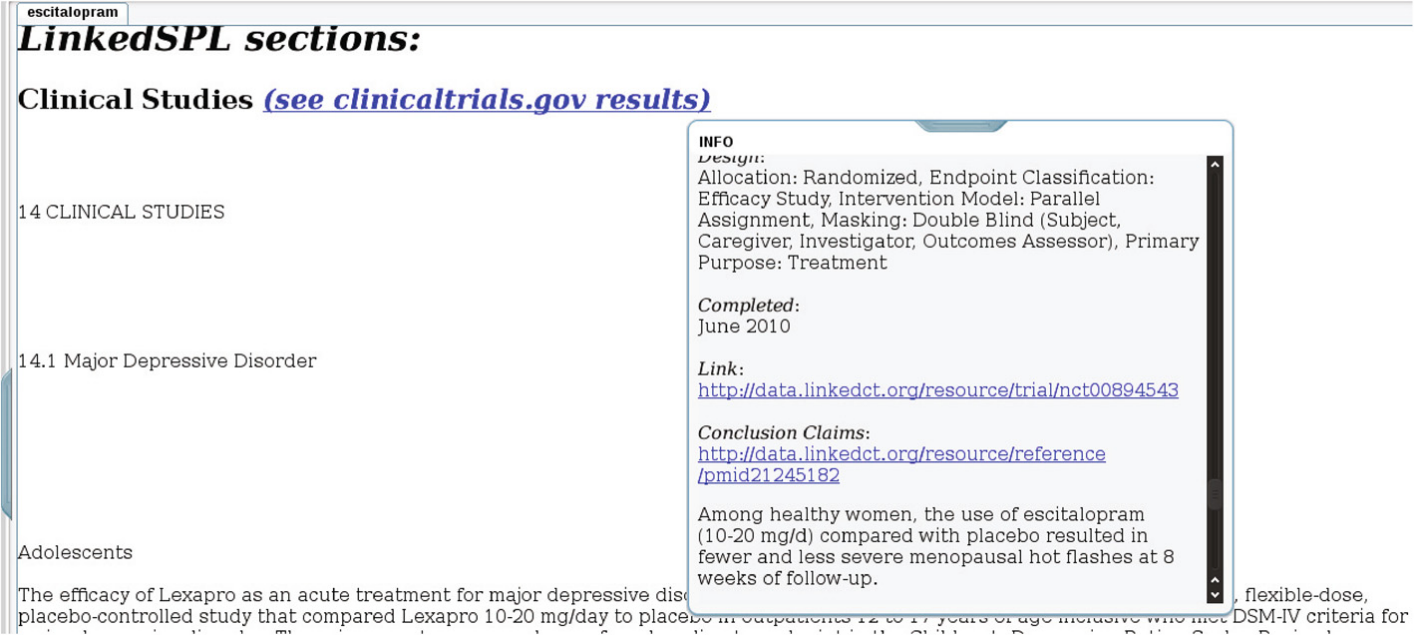
**A Clinical Study section from an escitalopram product label as shown in the proof-of-concept.** In this example, an efficacy claim is being shown that was routed from the abstract of a published result for study registered in ClinicalTrials.gov.

**Figure 4:**
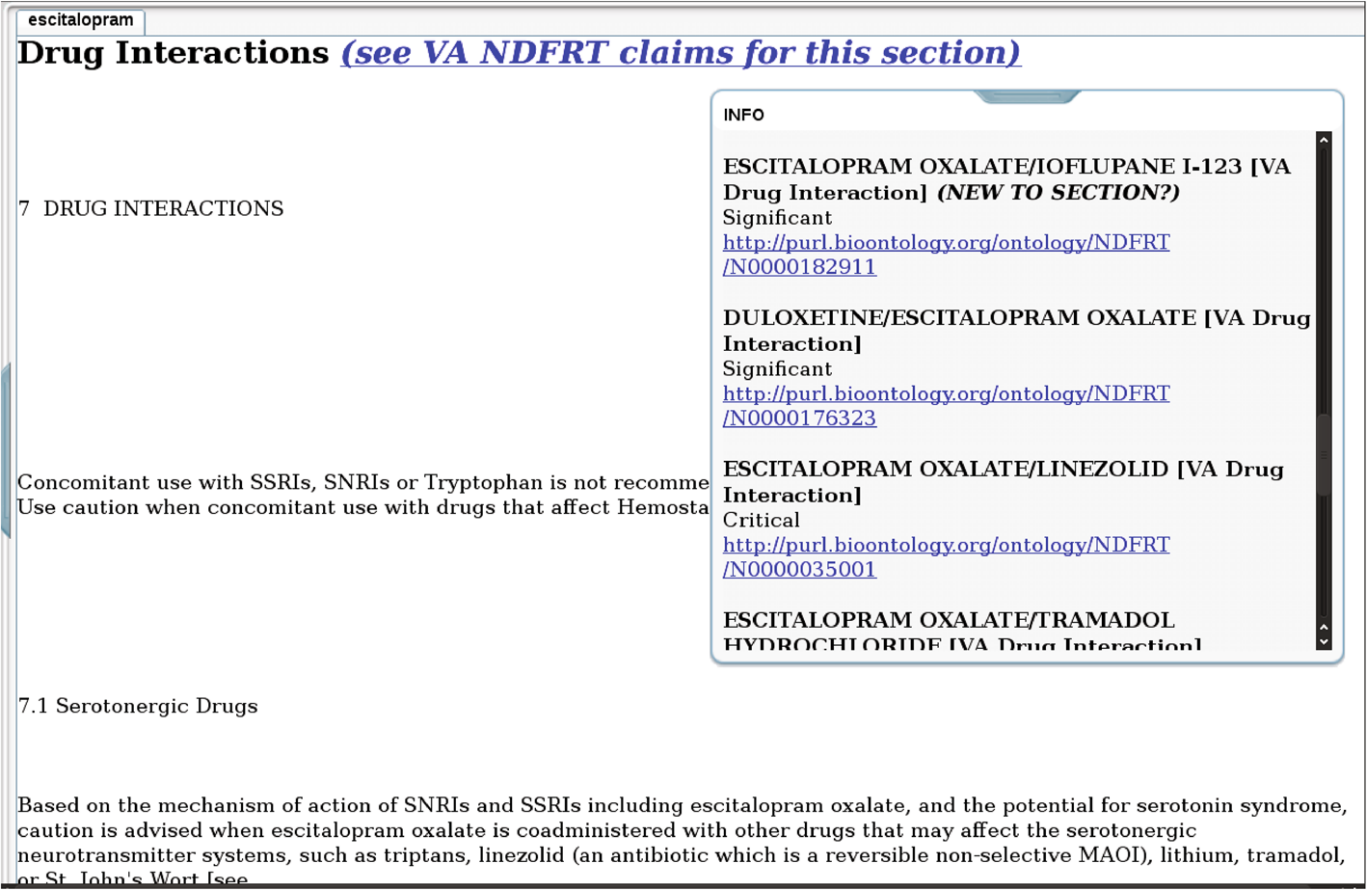
**A Drug Interactions section from an escitalopram product label as shown in the proof-of-concept.** In this example, several “Significant” NDF-RT drug-drug interactions are being shown. The interaction marked as ***New to Section?*** was not found by manual inspection of a single product label for an escitalopram drug product, nor by an automated case-insensitive string search of the Drug Interactions section of the escitalopram product label.

**Figure 5:**
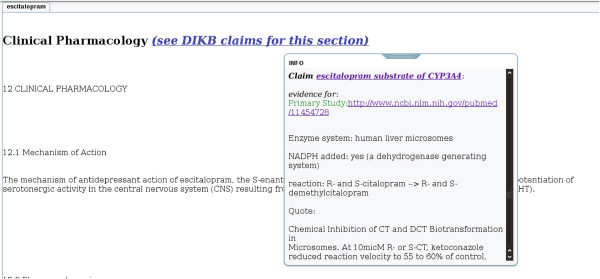
**A Clinical Pharmacology section from an escitalopram product label as shown in the proof-of-concept.** In this example, an DIKB metabolic pathway claim with supporting evidence is being shown.

The mappings described above were used to generate web pages that demonstrate one possible way that users could be presented with information that dynamically enhances product label sections. A total of 1,102 web pages were generated by the proof-of-concept using a version of LinkedSPLs that was synchronized with DailyMed content as of October 25, 2012. The web pages are publicly viewable at http://purl.org/net/nlprepository/outfiles-poc.^h^ Figures 3, 4 and 5 show examples of the web pages generated by the proof-of-concept for the three sections we chose to focus on.

### First steps towards the automated extraction of drug efficacy and effectiveness claims

It is important to note that, for drug efficacy and effectiveness claims, the proof-of-concept implements only one of the two steps that are needed to implement a fully automated claim extraction process. While the proof-of-concept retrieves text sources from which drug efficacy and effectiveness claims can be extracted (i.e., PubMed abstracts), these claims remain written in unstructured text. We hypothesized that sentences containing claims could be automatically extracted using a pipeline that processed the text of the abstracts returned from the LinkedCT query using an algorithm that automatically identifies sentences stating conclusions. To test the precision and recall of this approach, we first created a reference standard of these conclusion claims for a randomly chosen subset of psychotropic drugs. We then evaluated a publicly-available system called SAPIENTA [20] that can automatically identify conclusion sentences in unstructured scientific text.

#### Development of a reference standard of relevant claims

**Figure 6:**
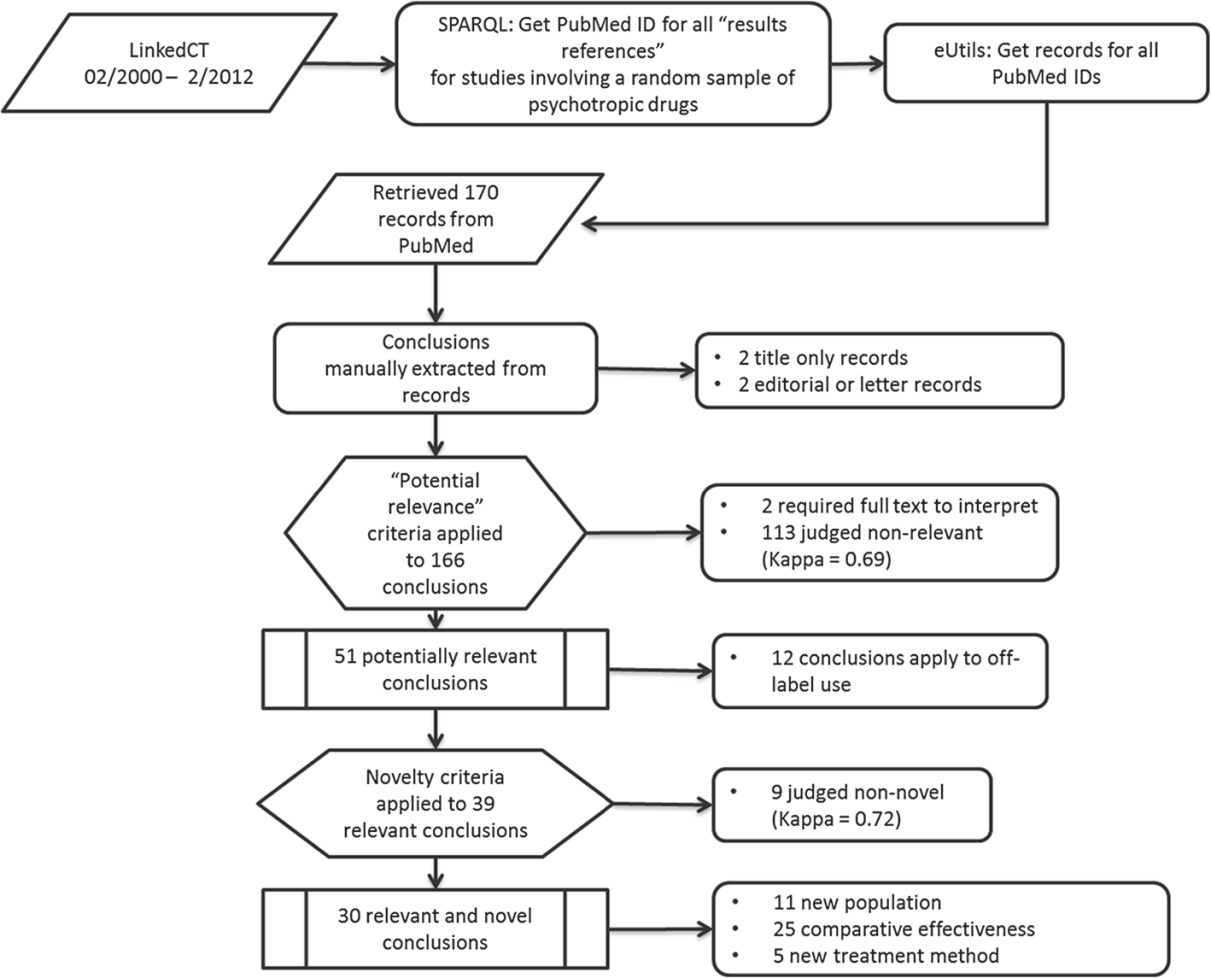
**A flow diagram of the process and results of identifying relevant and novel conclusions from efficacy and effectiveness studies routed to the product label *Clinical Studies *section via LinkedCT.**

Figure 6 shows the results of identifying relevant and novel conclusion claims from efficacy and effectiveness studies routed to the *Clinical Studies* section via LinkedCT. Table 3 lists results for each of the nine randomly-selected psychotropic drugs. A total of 170 abstracts were routed from PubMed to the *Clinical Studies* section of the products labels for the nine randomly sampled psychotropics. Four of the abstracts were either not clinical studies, or provided no other text content besides the title. These were dropped from further analysis. Of the 166 remaining conclusions, two were not interpretable without reading the full text article and 113 were judged to not be relevant to a pharmacist viewing the *Clinical Studies* section. For the remaining 51 relevant conclusions, the inter-rater agreement prior to reaching consensus was 0.69, reflecting “substantial” agreement according to the criteria of Landis and Koch [21].

**Table 3:** Relevance and novelty of conclusion claims based on manual validation

**Drug**	**ClinicalTrials.gov**	**Published results from ClinicalTrials.gov**			
	**studies involving the drug**	**studies involving the drug**			
		**N**	**Relevant N (%)**	**Novel (indication)**	**Novel (off-label use)**
*Antidepressants*					
Citalopram	4	25	5 (20)	5	
Duloxetine	4	4	4 (100)	3	
Escitalopram	6	9	3 (33)	1	2
Mirtazapine	1	22	1 (5)	1	0
Nortriptyline	3	24	2 (8)	1	1
Venlafaxine	2	2	2 (100)	1	1
*Antipsychotics*					
Olanzapine	5	13	7 (54)	6	1
Risperidone	23	70	26 (37)	21	5
*Sedative Hypnotics*					
Eszopiclone	1	1	1 (100)	0	1

The relevance and novelty of conclusion claims linked from three Linked Data drug information sources to the product labeling for nine randomly selected psychotropic drugs.

Twelve of the 51 relevant conclusions were judged to apply to uses of the drug other than those for which it was approved for by the FDA. Of the 39 relevant conclusions that applied to an approved indication, 30 were judged to be novel to the *Clinical Studies* section of at least one product label for a product containing the drug. Inter-rater agreement prior to reaching consensus on the novelty of these 30 relevant and novel conclusions was also substantial with a Kappa of 0.72.

#### Determination of the precision and recall of an automated extraction method

**Figure 7:**
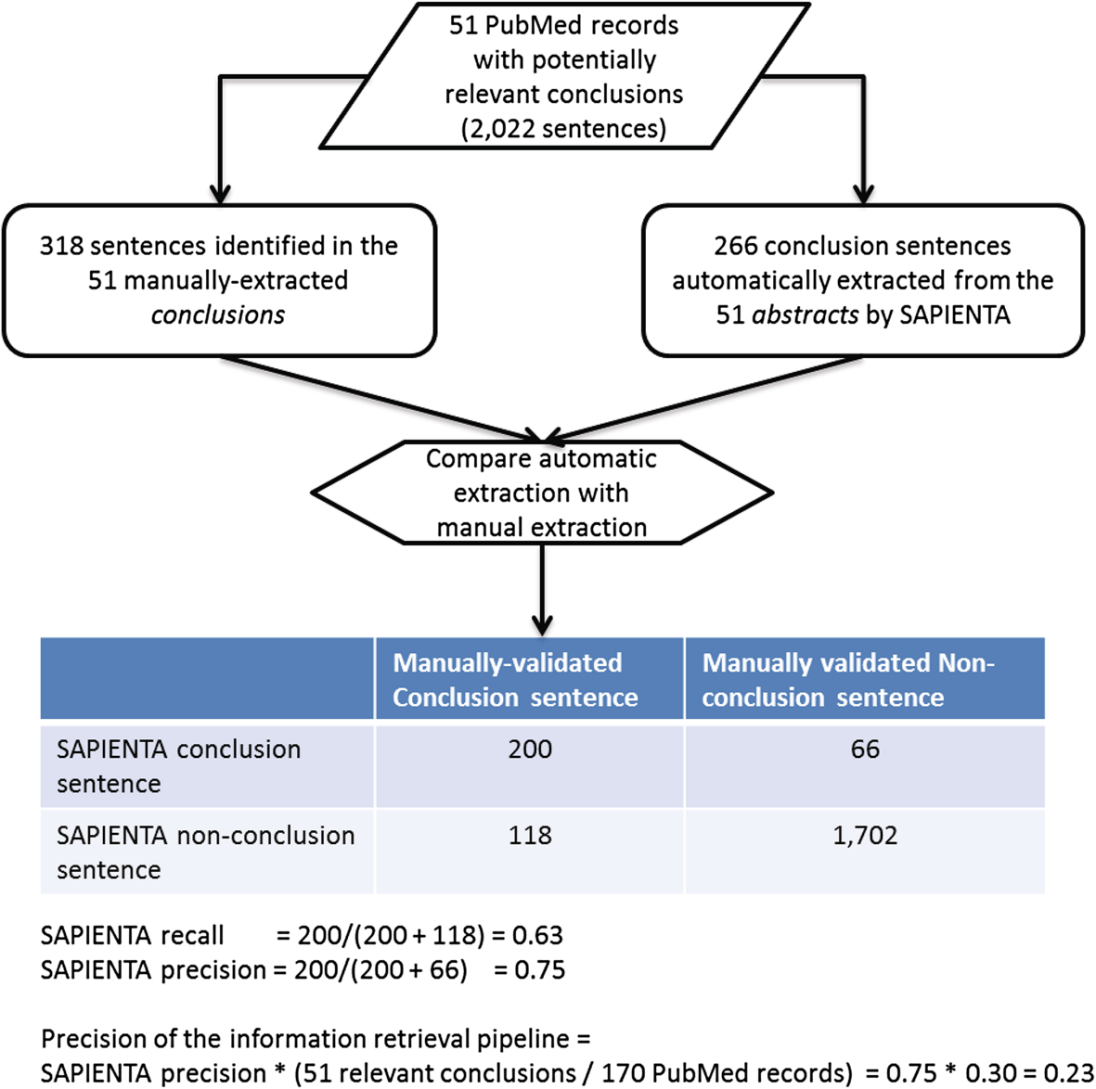
Determining the baseline information retrieval performance of the proof-of-concept system.

Figure 7 shows the results of determining the baseline information retrieval performance of the proof-of-concept system. SAPIENTA processed the same 170 abstracts mentioned in the previous section that were routed from PubMed to the *Clinical Studies* section of the product labels for the nine randomly sampled psychotropics. Of the more than 2,000 sentences in the 170 abstracts, the program automatically classified 266 sentences as Conclusions. In comparison, the conclusion claims extracted manually from the abstracts consisted of 318 sentences. Using these sentences as the reference standard, the recall, precision, and balanced F-measure for SAPIENTA was 0.63, 0.75, and 0.68 respectively. By combining these results with the precision of routing ClinicalTrials.gov study results to the *Clinical Studies* section via LinkedCT results in an overall “pipeline precision” of 0.23.

### A descriptive summary of challenges to the automated extraction of claims about a drug’s metabolic pathways

Although the proof-of-concept made links from claims about a drug’s metabolic pathways present in the DIKB resource to the *Clinical Pharmacology* section of the product label, the DIKB has claims for only a small subset (<100) of the thousands of drugs currently on the market. To further investigate the feasibility of automatically extracting claims about a drug’s pharmacokinetic properties, we manually traced the evidence for a small number of claims pertaining to the pharmacokinetics of escitalopram that the proof-of-concept linked from the DIKB to drug product labels. The goal of this effort was to see if there were particular patterns that we might use in future language analytics systems.

We found that the inhibition and substrate claims are derived from two texts, one describing a set of experiments to deduce the metabolic properties (i.e., biotransformation and enzyme inhibition) for escitalopram [22], and one a product label produced by Forest Labs [23]. As an example, there are two pieces of evidence against the claim “escitalopram inhibits CYP2C19” – first, from the Forest Labs text... 

In vitro enzyme inhibition data did not reveal an inhibitory effect of escitalopram on CYP3A4, -1A2, -2C9, -2C19, and -2E1. Based on in vitro data, escitalopram would be expected to have little inhibitory effect on in vivo metabolism mediated by these cytochromes.

...and second, from the Moltke *et al.* paper: 

CYP2C19. R- and S-CT were very weak inhibitors, with less than 50 percent inhibition of S-mephenytoin hydroxylation even at 100micM. R- and S-DCT also were weak inhibitors. R- and S-DDCT were moderate inhibitors, with mean IC50 values of 18.7 and 12.1micM, respectively. Omeprazole was a strong inhibitor of CYP2C19, as was the SSRI fluvoxamine (see Table 2).

The claim “escitalopram is a substrate of CYP2C19” is motivated by the following evidence in Moltke *et al.*: 

At 10micM R- or S-CT, ketoconazole reduced reaction velocity to 55 to 60 per cent of control, quinidine to 80 per cent of control, and omeprazole to 80 to 85 per cent of control (Figure 6). When the R- and S-CT concentration was increased to 100 M, the degree of inhibition by ketoconazole increased, while inhibition by quinidine decreased (Figure 6). These findings are consistent with the data from heterologously expressed CYP isoforms.

The validity of this claim depends on an assumption (“omeprazole is an *in vitro* selective inhibitor of enzyme CYP2C19”) which is a separate DIKB claim, supported by a draft FDA guidance document [24].

The next claim is that escitalopram’s primary clearance route is *not* by renal excretion and it is derived from the following sentence in the Forest Laboratories text: 

Following oral administrations of escitalopram, the fraction of drug recovered in the urine as escitalopram and S-demethylcitalopram (S-DCT) is about 8 per cent and 10 per cent, respectively. The oral clearance of escitalopram is 600 mL/min, with approximately 7 per cent of that due to renal clearance.

The connection between the evidence and the claim requires the domain knowledge that renal excretion is roughly the same as the fraction of dose recovered in urine.

Finally, the evidence for claims pertaining to escitalopram’s metabolites again comes from the Forest Labs text: 

Escitalopram is metabolized to S-DCT and S-didemethylcitalopram (S-DDCT).

From these examples, we ascertained four issues that present major challenges for the automated extraction of drug claims from a text source: 

**Self-referencing and anaphora.** In narrative text, coherence is often created by creating anaphoric co-reference chains - where entities at other locations in the text are referred to by pronouns (it, they) and determiners (these, this). This makes sentences such as these very easy for humans to read: 

R-CT and its metabolites, studied using the same procedures, had properties very similar to those of the corresponding S-enantiomers.

However, automatically identifying the entities referred by these referents “its metabolites”, “the same procedures”, “similar properties”, and “the corresponding S-enantiomers” is a non-trivial task.

**Use of ellipsis** Often statements are presented in a compact manner, where the full relations between drugs and proteins are omitted, as in this example: 

Based on established index reactions, S-CT and S-DCT were negligible inhibitors (IC50 > 100 *μ*M) of CYP1A2, -2C9, -2C19, -2E1, and -3A, and weakly inhibited CYP2D6 (IC50 = 70 - 80 *μ*M)

A computational system would need to “unpack” this statement to read the following list of relations (a total of 12 statements). 

• S-CT (escitalopram) was a negligible inhibitor ((IC50 >100 *μ*M) of CYP1A2

• S-CT (escitalopram) was a negligible inhibitor ((IC50 >100 *μ*M) of CYP2C9

• ...

**Domain knowledge is needed to be able to resolve anaphora.** The metabolites referred to in the phrase “R-CT and its metabolites”, above, which is referred to six times in the text, are not explicitly described in the text. For even a human to be able to define what they are it is necessary that they know that the following sentence contains a definition of the metabolites studied: 

Transformation of escitalopram (S-CT), the pharmacologically active S-enantiomer of citalopram, to S-desmethyl-CT (S-DCT), and of S-DCT to S-didesmethyl-CT (S-DDCT), was studied in human liver microsomes and in expressed cytochromes (CYPs).

Interestingly, this information is given only in the abstract of the paper.

**Key components are provided in other papers.** As with textual coherence, inter-textual coherence, embedding the current text in the corpus of known literature, is an important function of the text. In certain cases key elements of the paper, such as the methods, are entirely described through a reference, e.g.: 

Average relative *in vivo* abundances [... ] were estimated using methods described in detail previously (Crespi, 1995; Venkatakrishnan et al., 1998 a,c, 1999, 2000, 2001; von Moltke et al., 1999 a,b; Störmer et al., 2000).

There is of course no way to ascertain what methods were used without (computational) access to these references; even so it might well not be obvious or easy to identify the relevant methods in the referenced texts.

## Discussion

To the best of our knowledge, this is the first study to demonstrate how claims about drug safety, efficacy, and effectiveness present in Semantic Web resources can be linked to the relevant sections of drug product labels. While we focused on only three drug information resources and a relatively small set of marketed drugs, the resulting Linked Data set contains a considerable number of claims that might help meet pharmacist information needs. We emphasize that this was a pilot study and our results are exploratory.

It is noteworthy that the labels for all 1,102 drug products containing the drugs in our study could be linked to at least one claim, and that, on average, 50 claims could be linked to each product label. This suggests that there are ample claims available on the Semantic Web that can be linked to drug product labeling. One concern is that, while the approach might do a good job of linking *more* information with the product label, it might be poor at providing the *right* kind of information. Our analysis of a relatively simple automated approach that combines a routing strategy with an existing scientific discourse analysis program (SAPIENTA) found that about one in five efficacy/effectiveness conclusion claims would be relevant to the *Clinical Studies* section of a psychotropic drug product, the majority of which would provide the pharmacist with new information about an indicated use of the drug (Figure 6).

We also found evidence that if we performed this endeavor at scale, many relevant and novel drug-drug interaction claims would be found that could be linked to the *Drug Interactions* section of the product label. At least one potentially novel interaction was linked to all 20 antidepressants, and there were several cases where *all* of the drug-drug interactions linked to the *Drug Interactions* section for an antidepressant were potentially novel. However, these results require further validation to ensure that differences in how the drugs are referred to between drug information sources, and between product labels, are properly accounted for. For example, an NDF-RT interaction between digoxin and nefazodone was incorrectly marked as potentially novel to nefazodone product labels because the NDF-RT referred to digoxin by “digitalis”, a broad synonym for drugs derived from foxglove plants that are used to treat cardiac arrhythmias.

A manual inspection of potentially novel interactions linked to several antidepressant product labels by co-investigator JRH (a pharmacist and drug-interaction expert) suggested that several of the linked interactions would complement product label information. For example, the NDF-RT interaction between escitalopram and tapentadol was potentially novel to all 20 escitalopram product labels. While no explanation for this NDF-RT interaction is provided in the resource, it is possibly based on the potential for tapentadol to interact in an additive way with selective serotonin reuptake inhibitors (SSRIs). This interaction might increase the risk of an adverse event called “serotonin syndrome.” The labels for all SSRIs appear to provide a generally-stated class based interaction between SSRIs and other drug affecting the serotonin neurotransmitter pathway. However, one would have to know that tepentadol fits in this category. Another example is the NDF-RT interaction between metoclopramide and escitalopram. As with the other example, this interaction was potentially novel to all escitalopram product labels and no explanation was provided in the NDF-RT resource. The possible reason that the NDF-RT notes the interaction is that escitalopram is a weak inhibitor of the Cytochrome P450 2D6 metabolic enzyme which is a potentially important clearance pathway for metoclopramide. Thus, the drug combination might increase the risk of metoclopramide toxicity in some patients leading to adverse events such as Tardive Dyskinesia.

Manual inspection also identified examples of potentially novel NDF-RT interactions that might not be mentioned in the label due to indeterminate evidence. Three NDF-RT interactions involved amoxapine as an object drug and rifampin, rifabutin, and rifapentine as precipitant drugs. No explanation was accessible from the NDF-RT resource and no clear mechanism was apparent based on the drugs’ metabolic properties. For example, while rifampin is a known inducer of certain Cytochrome P450s (especially Cytochrome P450 3A4), we were unable to find evidence of an induction interaction between rifampin and amoxapine by searching a rifampin product label [25]. Similarly, no results were returned from the PubMed query RIFAMPIN AMOXAPINE INTERACTION. The same was true for searches conducted for rifabutin and rifapentine. Thus, while it is possible that these interactions are missing from the product label, it is also possible that insufficient evidence for the clinical relevance of the interaction justifies their exclusion.

The concern that drug-drug interactions are often based on poor evidence (such as single case reports or predictions) was raised at a recent multi-stakeholder conference focusing on the drug-drug interaction evidence base [26]. Another concern raised at the conference was that there is currently no standard criteria for evaluating the evidence for interactions. This leads to considerable variation in the drug-drug interactions listed across drug information sources [14]. In future work we plan to develop methods that construct more complete claim-evidence networks for drug-drug interactions that go beyond establishing the potential for the interaction [27], to also provide evidence of the potential risk of harm in patients with specific characteristics.

Inspection of the 113 non-relevant abstracts for published results (see Figure 6) suggests that our approach to identifying studies that were about a specific drug returned many false positives. We think that this issue is primarily due to how we linked the published results from studies registered in ClinicalTrials.gov to the drugs included in our study. In LinkedCT, entities tagged in ClinicalTrials.gov as “interventions” for a study are mapped to entities tagged as “drugs” in DrugBank using a combination of semantic and syntactic matching that has been shown to notably improve the linkage results compared with matching by strings tokens alone [28]. However, many studies have multiple interventions. For example, study NCT00015548 (The CATIE Alzheimer’s Disease Trial)^i^ lists three antispychotics and one antidepressant as interventions. As a result, the published results for NCT00015548 that we linked to product labels for the antidepressant drug (citalopram) included many results that were actually about the effectiveness of one of the antipsychotic drugs. Changing how we address this issue should result in a significant improvement in the pipeline precision of the automated system. One possibility would be to exclude published results that do not mention an indicated or off-label use of the drug (e.g., “depression” in the case of citalopram). Future work should focus creating and validating a weighted combination of such filters.

The manual analysis of metabolic pathway claims pertaining to escitalopram found several factors that might complicate automated extraction (complex anaphora, co-reference, ellipsis, a requirement for domain knowledge, and recourse to external documents via citations). These offer some pointers to future work on automated extraction. However, it is also useful to consider how new innovations in science publishing might enable the author of a scientific paper to annotate a claim written into his/her scientific article. To be feasible, this requires usable tools and a set of simple standards that make annotation during the publishing process efficient. Efforts along these lines are currently being pioneered by groups such as the Neuroscience Information Framework^j^.

We approached this proof-of-concept primarily thinking about a pharmacist’s information needs, but as Figure 1 shows, there are other potential stakeholders such as regulators, pharmacoepidemiologists, the pharmaceutical industry, and designers of clinical decision support tools. The FDA has recently set challenging goals for advancing regulatory science [29] making the agency a particularly important stakeholder for future work. One regulatory science application of the approach might be to identify possible quality issues in drug product labels. For example, Listing Listing 2 A query for all NDF-RT drug interactions that are potentially novel to the Drug Interactions section of bupropion product labels shows a direct query for all NDF-RT drug interactions that are potentially novel to the Drug Interactions section of any bupropion product label. The result of this query makes it evident that there are three NDF-RT interactions (bupropion/carbamazepine, bupropion/phenelzine, and bupropion/tamoxifen) that are potentially novel to some bupropion product labels but not others. Assuming that the interactions are truly novel (which is not validated at this time), this finding might indicate inconsistency across product labels that could require further investigation.

### Listing 2 A query for all NDF-RT drug interactions that are potentially novel to the Drug Interactions section of bupropion product labels

PREFIX poc: <http://purl.org/net/nlprepository/dynamic-spl-enhancement-poc#>

SELECT ?label COUNT(DISTINCT ?spl) WHERE {

poc:ndfrt-ddi-map ?spl ?ddiMap.

?ddiMap poc:ndfrt-ddi-drug "bupropion".

?ddiMap poc:ndfrt-ddi-label ?label.

?ddiMap poc:ndfrt-ddi-severity ?severe.

OPTIONAL{?ddiMap poc:ndfrt-ddi-potentially-novel ?novel.}

FILTER (BOUND(?novel))

}

GROUP BY ?label

ORDER BY ?label

Doctors and patients might also benefit from dynamically enhanced product label information. For example, the proof-of-concept linked numerous NDF-RT drug-drug interactions involving Ioflupane I-123 to the labels for SSRI drugs. In all cases, these were marked as potentially novel to the Drug Interactions section of the label. Ioflupane I-123 is used to help radiologists test adult patients for suspected Parkinsonian syndrome using a brainscan. The concern here is that the SSRIs might alter the ability of Ioflupane to bind to dopamine transporters, possibly reducing the effectiveness of the brainscan [30]. Radiologists and patients, in addition to pharmacists, might benefit from knowledge of this interaction. With the current trend for participatory medicine, patients are playing a greater role in their health and we think that its important in future work to consider how the novel approach could be used to help them avoid adverse drug reactions by self monitoring (or monitoring for someone whose care they manage).

### Limitations

There are some potential limitations to this study. While we evaluated the relevance and novelty of the efficacy/effectiveness conclusion claims, our evaluation included only a small number of randomly-selected drugs. It is possible that the performance characteristics we found for the nine psychotropics are not generalizable to all psychotropic drug products, or to products containing drugs from other classes. A similar potential limitation exists for drug-drug interactions. Due to resource limitations, we could only examine the potential novelty of interactions linked to antidepressant drug products and the results might be different for other drugs or drug classes.

We linked claims from three information sources that we expected to be relevant to pharmacists seeking information about the efficacy, effectiveness, and safety of a drug. However, the drug information sources we chose might not be representative of all sources of drug claims on the Semantic Web because we chose sources known to be clinically oriented. Due to the hypothesis-driven nature of basic and translational science, we expect that information sources designed to support these user groups might provide a smaller proportion of claims that would be relevant to pharmacists and other clinicians. A scaled approach may require labeling each included drug information resource with meta-data describing its purpose and construction. This would enable claims to be filtered to meet the needs of various user groups.

Finally, the results of our evaluation of SAPIENTA may have been influenced by how we defined conclusion claims. The SAPIENTA system labels any given sentence with one of 11 possible core scientific concept tags (of which Conclusion is one), and so is designed to identify all likely Conclusion sentences. However, the research librarian who helped to produce the reference standard extracted consecutive sentences that he judged were part of a conclusions *section*, rather than attempting to identify *every* sentence that reported a conclusion. Thus, some of the SAPIENTA Conclusion sentences that were judged to be false positives might have contained informative conclusions. A similar issue is that our evaluation was performed on abstracts rather than full text articles. While SAPIENTA was originally trained on full text articles from a different scientific domain, its performance in this task might have been influenced by the concise and structured organization of biomedical abstracts. Future work should examine the approach’s “pipeline precision” using full text articles and a less section-based approach to defining conclusion claims.

### Related work

In recent years, the field of biological text mining has focused on automatically extracting biomedical entities and their relationships from both the scientific literature and the product label. The goal of much of this work has been to facilitate curation of biological knowledge bases [31,32]. While it seems that very little research has been directed toward the extraction of claims about a drug’s effectiveness or efficacy, there has been a growing interest in the recognition of drug entities, and the extraction of drug side-effects and interactions. With respect to the dynamic enhancement of drug product labeling, these methods can be divided into those that 1) identify claims present in product labeling and 2) produce claims that may be linkable to the product label.

#### Methods that identify claims present in product labeling

Duke *et al.* developed a program to extract adverse events written into the product label that was found to have a recall of 92.8% and a precision of 95.1% [33]. Comparable work by Kuhn *et al.* associated 1,400 side effect terms with more than 800 drugs [34]. In previous work co-author RDB produced a manually-annotated corpus of pharmacokinetic drug-drug interactions and high-performance algorithm for extracting drug-drug interactions from drug product labels [35]. The corpus was built by two annotators who reached consensus on 592 pharmacokinetic drug-drug interactions, 3,351 active ingredient mentions, 234 drug product mentions, and 201 metabolite mentions present in over 200 sections extracted from 64 drug product labels. The drug interaction extraction algorithm achieved an F-measure of 0.859 for the extraction of pharmacokinetic drug-drug interactions and 0.949 for determining if the modality of the interactions (i.e., a positive interaction or confirmation that no interaction exists). Efforts on product labels outside of the United States include Takarabe *et al.* who describe the automated extraction of over 1.3 million drug-interactions from Japanese product labels [36]. Also, Rubrichi and Quaglini reported excellent performance (macro-averaged F-measure: 0.85 vs 0.81) for a classifier they designed to assign drug-interaction related semantic labels to text of the drug interaction section of Italian “Summary of Product Characteristics” documents [37].

#### Methods that produce claims that may be linkable to the product label

Multiple translational researchers have produced new algorithms for identifying drug-drug interactions and metabolic pathways. Segura-Bedmar constructed a drug-drug interaction corpus [38] consisting of documents from DrugBank annotated with drug-drug interactions. This corpus was the focus of ten research papers presented at the recent “Challenge Task on Drug-Drug Interaction Extraction” held at the 2011 SemEval Conference [39]. The best performing system in this challenge achieved an F-measure of 0.657 [40]. A second round of this challenge is being held in 2013 with a corpus expanded to include drug-drug interactions from MEDLINE. Percha *et al.* built on work done by Coulet *et al.*[41] on extracting and characterizing drug-gene interactions from MEDLINE to to infer new drug-drug interactions [42].

Recent work by Duke *et al* used a template based approach to extract metabolic pathways from the scientific literature, and then used the extracted metabolic pathways to make drug-interaction predictions [43]. While similar to the work of Tari *et al.*[44], Duke *et al*. went further by developing a pipeline for gathering pharmacoepidemiologic evidence of the association of the predicted drug interactions with specific adverse events. Their approach of linking population data on the risk of specific adverse events in patients exposed to specific drug-drug interactions is groundbreaking, and has the potential to address the challenge of knowing with any confidence how risky a potential drug-drug interaction will be for a particular patient population [26]. By linking drug-drug interaction claims with data on exposure and adverse events, clinicians may be better able to assess the risk of allowing their patient to be exposed to a potential interaction. We would like to integrate this and similar research in our future work on the dynamic enhancement of the *Drug Interactions* section of the product label.

## Conclusions

We have demonstrated the feasibility of a novel approach to addressing known limitations in the completeness and currency of product labeling information on drug safety, efficacy, and effectiveness. Our evaluation of a proof-of-concept implementation of the novel approach suggests that it is potentially effective. The baseline performance characteristics of the proof-of-concept will enable further technical and user-centered research on robust methods for scaling the approach to the many thousands of product labels currently on the market.

## Methods

### Linking relevant semantic web resources to the product label

SPLs are documents written in a Health Level Seven standard called *Structured Product Labeling* that the FDA requires industry to use when submitting drug product label content [45]. More specifically, an SPL is an XML document that specifically tags the content of each product label section with a unique code from the Logical Observation Identifiers Names and Codes (LOINC *Ⓡ*) vocabulary [46]. The SPLs for all drug products marketed in the United States are available for download from the National Library of Medicine’s DailyMed resource [47]. At the time of this writing, DailyMed provides access to more than 36,000 prescription and over-the-counter product labels.

The SPLs for all FDA-approved prescription drugs were downloaded from the National Library of Medicine’s DailyMed resource. We created an RDF version of the data using a relational-to-RDF mapping approach. This approach was chosen because it allows for rapid prototyping of RDF properties and tools are available that provide a convenient method for publishing the data in human navigable web pages. Custom scripts were written that load the content of each SPL into a relational database. The relational database was then mapped to an RDF knowledge base using the D2R relational to RDF mapper [48]. The mapping from the relational database to RDF was derived semi-automatically and enhanced based on our design goals, and a final RDF dataset was generated which is hosted on a Virtuoso RDF server^k^ that provides a SPARQL endpoint.

Listing Listing 3 Queries for product label content and metadata present in the “LinkedSPLs” RDF graph shows the SPARQL query used to retrieve content from the *Clinical Studies*, *Drug Interactions*, and *Clinical Pharmacology* sections of the product label data for each psychotropic drug.

### Listing 3 Queries for product label content and metadata present in the “LinkedSPLs” RDF graph

PREFIX rdfs: <http://www.w3.org/2000/01/rdf-schema#>

PREFIX dailymed: <http://dbmi-icode-01.dbmi.pitt.edu/linkedSPLs/vocab/resource/>

PREFIX foaf: <http://xmlns:m.com/foaf/0.1/>

## Get metadata for the SPLs of all products containing a drug ##

SELECT ?label ?splId ?version ?setId ?org ?date ?homepage

WHERE {

  ?splId rdfs:label ?label.

  ?splId dailymed:subjectXref <%s>. ## The URI to the drug in DrugBank ##

  ?splId dailymed:versionNumber ?version.

  ?splId dailymed:setId ?setId.

  ?splId dailymed:representedOrganization ?org.

  ?splId dailymed:effectiveTime ?date.

  ?splId foaf:homepage ?homepage.

}

## Get the three sections of interest for a specific SPL ##

##(substituting an ?splid value from the above query for %s) ##

SELECT ?textClinicalStudies ?textDrugInteractions ?textClinicalPharmacology

WHERE {

  OPTIONAL { <%s> dailymed:clinicalStudies ?textClinicalStudies }

  OPTIONAL { <%s> dailymed:drugInteractions ?textDrugInteractions}

  OPTIONAL{ <%s> dailymed:clinicalPharmacology ?textClinicalPharmacology }

}

#### Automatic linking of study abstracts from ClinicalTrials.gov to the Clinical Studies section

We wrote a custom Python script^l^ that queried the Linked Data representation of SPLs for the *Clinical Studies* sections of each of the drugs included in this study (see Listing Listing 4 LinkedCT Query for study results indexed in PubMed). For each returned section, the script queried the LinkedCT SPARQL endpoint for clinical studies registered with ClinicalTrials.gov that were tagged in LinkedCT as 1) related to the drug that was the active ingredient of the product for which the section was written, and 2) having at least one published result indexed in PubMed. The former criterion was met for a study if LinkedCT provided an RDF Schema seeAlso property to DrugBank for the drug. The latter criterion was met if LinkedCT had a trial_results_reference property for the study. The result of this process was a mapping from the meta-data for each published result to the *Clinical Studies* section from a product label.

### Listing 4 LinkedCT Query for study results indexed in PubMed

PREFIX rdfs: <http://www.w3.org/2000/01/rdf-schema#>

PREFIX linkedct: <http://data.linkedct.org/vocab/resource/>

SELECT ?trial, ?title, ?design, ?completion, ?reference

WHERE {

 ?trial a <http://data.linkedct.org/vocab/resource/trial>;

     linkedct:trial_intervention ?inter;

     linkedct:study_design ?design;

     linkedct:official_title ?title;

     linkedct:completion_date ?completion;

     linkedct:trial_results_reference ?reference.

 ?inter rdfs:seeAlso <%s>. ## the URI to the drug in DrugBank ##

}

#### Automatic linking of VA NDF-RT drug-drug interactions to the Drug Interactions section

We extended the custom Python script to query the Linked Data representation of SPLs for the *Drug Interactions* sections of each of the drugs included in this study. For each returned section, the script queried the BioPortal SPARQL endpoint for drug-drug interactions in the NDF-RT resource involving the drug that was identified as the active ingredient of the product for which the section was written (see Listing Listing 5 BioPortal Query for NDF-RT drug-drug interactions). The NDF-RT labels the drug-drug interactions that it provides “Critical” or “Significant” reflecting judgment by members of the national Veteran’s Administration (VA) formulary on the potential importance of the interaction [18]. Because they are considered to have a greater potential for risk, those interactions labeled “Critical” are less modifiable by local VA formularies than interactions labeled “Significant.” The script queried for interactions tagged with either label. The result of this process was a mapping between the content of the *Drug Interactions* section from a product label to a list of one or more NDF-RT drug-drug interactions.

### Listing 5 BioPortal Query for NDF-RT drug-drug interactions

PREFIX owl: <http://www.w3.org/2002/07/owl#>

PREFIX xsd: <http://www.w3.org/2001/XMLSchema#>

PREFIX rdfs: <http://www.w3.org/2000/01/rdf-schema#>

PREFIX skos: <http://www.w3.org/2004/02/skos/core#>

PREFIX ndfrt: <http://purl.bioontology.org/ontology/NDFRT/>

SELECT DISTINCT ?s ?label ?severity

FROM <http://bioportal.bioontology.org/ontologies/NDFRT>

WHERE {

  ?s ndfrt:NDFRT_KIND ?o;

  skos:prefLabel ?label;

  ndfrt:SEVERITY ?severity. FILTER (regex(str(?o), “interaction”, “i”))

  ?s ndfrt:has_participant ?targetDrug.

  ?s ndfrt:STATUS “Active” ^∧∧^xsd:string.

  ?targetDrug skos:prefLabel “%s”@EN. ## Preferred label for the drug in the

NDF-RT ##

}

The script was expanded to test how many NDF-RT interactions might be novel to the Drug Interactions section of each drug product label. A *potentially novel* interaction was defined as an NDF-RT interaction that was 1) not mentioned in the Drug Interaction section of a product label based on a case-insensitive string match, and 2) not listed in a reference set of interactions created prior to the study as part of work done for [4]. The reference set listed pharmacokinetic and pharmacodynamic interactions derived by manually inspecting a single product label for each *antidepressant* drug. The reference set (Additional file 1: Table S4) was created by two reviewers who were both informaticists specializing in drug information. Interactions involving drug classes were expanded to include all drugs in the class using class assignments in the NDF-RT terminology. The reference set did not include interactions from antipsychotic or sedative hypnotic drug product labels. For these drugs, only the first criterion mentioned above was used to identify a *potentially novel* interaction.

#### Automatic linking of metabolic pathway claims from the Drug Interaction Knowledge Base to the Clinical Pharmacology section

We extended the custom Python script once more to query the Linked Data representation of SPLs for the *Clinical Pharmacology* sections of each of the drugs included in this study. For each returned section, the script queried the DIKB SPARQL endpoint for claims about the pharmacokinetic drug properties of the active ingredient of the product for which the section was written (see Listing Listing 6 Queries to the DIKB for pharmacokinetic drug property claims). The DIKB provides meta-data on the sources of evidence for each claim and uses terms from the SWAN scientific discourse ontology [8] to label each evidence source as one that either supports or refutes the claim. The script queried for pharmacokinetic drug property claims with either supporting or refuting evidence sources. The result of this process was a mapping between the content of the *Clinical Pharmacology* section from a product label to a list of one or more pharmacokinetic drug property claims and associated evidence sources.

### Listing 6 Queries to the DIKB for pharmacokinetic drug property claims

PREFIX swanco: <http://purl.org/swan/1.2/swan-commons#>

PREFIX dikbD2R: <http://dbmi-icode-01.dbmi.pitt.edu:2020/vocab/resource/>

## The enzymes that the drug is a substrate of ##

SELECT ?asrtId ?enz ?evFor ?evAgainst

WHERE {

  ?asrtId dikbD2R:object <%s>. ## Drug URI in the DIKB ##

  ?asrtId dikbD2R:slot dikbD2R:substrate_of.

  ?asrtId dikbD2R:value ?enz.

  OPTIONAL {?asrtId swanco:citesAsSupportingEvidence ?evFor }

  OPTIONAL {?asrtId swanco:citesAsRefutingEvidence ?evAgainst }

}

## The enzymes that the drug inhibits ##

SELECT ?asrtId ?enz ?evFor ?evAgainst

WHERE {

  ?asrtId dikbD2R:object <%s>. ## Drug URI in the DIKB ##

  ?asrtId dikbD2R:slot dikbD2R:inhibits. ?asrtId dikbD2R:value ?enz.

  OPTIONAL {?asrtId swanco:citesAsSupportingEvidence ?evFor}

  OPTIONAL {?asrtId swanco:citesAsRefutingEvidence ?evAgainst }

}

#### Generation of web page mashups

The same Python script used to generate mappings was extended to write a single web page for each drug product that included the text content of three sections mentioned above. A link was placed above each section that enabled users to view the claims that had been mapped to that section in a pop-up window. The pop-ups showing claims linked to the *Drug Interactions* section provide a cue to the user when the linked interactions were potentially novel to the label (see above for further detail). Similarly, the popups for claims linked to the *Clinical Pharmacology* section cued the user when a specific metabolic pathway claim may be novel to the product label based on a simple string search of the text of the *Clinical Pharmacology* section for the metabolic enzyme reported in the linked claim.

The Rialto Javascript widget library was used to generate the web pages and popups.^m^ All code and data for the proof-of-concept is archived at the Swat-4-med-safety Google Code project.^o^

### First steps towards the automated extraction of drug efficacy and effectiveness claims

#### Development of a reference standard of relevant claims

Figure 6 provides a flow diagram of the process for identifying relevant and novel conclusions from efficacy and effectiveness studies routed to the product label *Clinical Studies* section via LinkedCT. Nine psychotropic drugs were selected randomly from the 29 psychotropic drugs used to create the proof-of-concept. Any study registered in ClinicalTrials.gov that was associated with one of the nine drugs in LinkedCT, and that had published results (see Listing Listing 4 LinkedCT Query for study results indexed in PubMed), was included in the development of the reference standard. Abstracts for papers publishing results from a study were retrieved from PubMed using the PubMed identifier found in the URI values assigned to the trial_results_reference property in the query shown in Listing Listing 4 LinkedCT Query for study results indexed in PubMed.

We then manually identified conclusions from each abstract. A single research librarian with training in drug information retrieval identified conclusions written into the abstract. Abstracts describing clinical studies tend to share a similar structure consisting of brief introduction, methods, conclusions, and results sections. Therefore, the librarian extracted consecutive sentences that he judged were part of a conclusions *section* rather than attempting to annotate every sentence that reported a conclusion.

Once these conclusion claims were manually extracted, two reviewers (the librarian and co-author RDB) independently determined which of them would be potentially relevant to the *Clinical Studies* section of a product label for each drug in our study. The criteria for “potentially relevant” was based on the language of section “(15)/14 Clinical studies” of CFR 201 which states that this section of the label should describe at least one clinical efficacy study for each labeled indication. Because pharmacists would be the target users for the system that we envision, we expanded the relevance criteria to include: 

1. any study involving a population different from the average where it was shown that the drug should be used slightly differently in order to be safe or effective, and

2. efficacy or effectiveness studies for the off-label uses mentioned in a widely-used drug information source [49].

The reviewers made relevance judgements independently and based only on information in the abstract. The agreement of two reviewers over random chance (Kappa) was calculated before the reviewers reached consensus on a final set of relevant conclusions. Disagreements were resolved by co-investigator JRH who is also a pharmacist. The same pharmacist reviewed the consensus judgments and noted if each potentially relevant conclusion refers to the efficacy/effectiveness of the drug for an labeled indication, or an off-label use mentioned in a widely-used drug information source [49]. Another round of review was done by JRH and the research librarian focusing on the novelty of relevant claims. These reviewers compared each relevant conclusion with the text of the Clinical Studies section from a single product label for the intervention drug. The label sections were sampled by convenience in the first week of August 2012. As was done for relevance judgements, Kappa was calculated before the reviewers reached consensus on a final set of novel conclusions. Finally, descriptive statistics and counts were derived for the following: 

• The number of potentially relevant conclusions present in PubMed abstracts that could be routed via ClinicalTrials.gov.

• The number of potentially relevant conclusions that would be novel to the Clinical Studies section.

• The number of potentially relevant conclusions that deal with off-label uses of a drug.

#### Determination of the precision and recall of an automated extraction method

Figure 7 shows a flow diagram of the process we implemented for determining the baseline information retrieval performance of a fully automated extraction method that could be implemented in the proof-of-concept system. A publicly available online system called SAPIENTA [20] was used to automatically annotate sentences in the same text sources that were used to create the reference standard. The tool annotated each sentence with one of 11 core scientific concepts (Hypothesis, Motivation, Background, Goal, Object, Method, Experiment, Model, Result, Observation, Conclusion). The system uses Conditional Random Field models [50] that have been trained on 265 papers from chemistry and biochemistry, and makes classification decisions according to a number of intra-sentential features as well as features global to the document structure.

The sentences automatically classified by SAPIENTA as Conclusions were compared with the conclusions manually-extracted by the research librarian to determine the precision and recall of SAPIENTA for identifying conclusion sentences. We also calculated an overall “pipeline precision” which combined the precision of the LinkedCT queries for retrieving text sources from which drug efficacy and effectiveness claims can be extracted with the precision of SAPIENTA for automatically extracting conclusion sentences. “Pipeline recall” was not evaluated because it would have required a systematic search for articles relevant to the efficacy and effectiveness for each study drug, something that was not feasible for this study.

## Endnotes

^a^The 29 active ingredients used for this study were: amitriptyline, amoxapine, aripiprazole, bupropion, citalopram, clozapine, desipramine, doxepin, duloxetine, escitalopram, eszopiclone, fluoxetine, imipramine, mirtazapine, nefazodone, nortriptyline, olanzapine, paroxetine, quetiapine, risperidone, selegiline, sertraline, tranylcypromine, trazodone, trimipramine, venlafaxine, zaleplon, ziprasidone, and zolpidem.^b^LinkedCT maintained by co-author OH and is available at http://linkedct.org/.^c^The NDF-RT is maintained by the Veteran’s Administration. A publicly available version of the resource is present in the Bioportal at http://purl.bioontology.org/ontology/NDFRT.^d^Co-author RDB maintains the DIKB, it is accessible at http://purl.org/net/drug-interaction-knowledge-base/.^e^The DailyMed website is located at http://dailymed.nlm.nih.gov/dailymed/.^f^Sample product label data in LinkedSPLs can be viewed at http://purl.org/net/linkedspls. The SPARQL endpoint is at http://purl.org/net/linkedspls/sparql.^g^The graph has 161 metabolic pathway mappings but 49 are to the same claims with different evidence items. Thus, there are 112 unique metabolic pathway claims.^h^Please note that the proof-of-concept web pages work for Internet Explorer 7.0 and 8.0, Mozilla 5.0, Firefox ≥ 2.0, and Google Chrome Version 22. They are known to not work on Safari, Internet Explorer 9.0, and versions of Internet Explorer (≤ 6.0).^i^This study is viewable in ClinicalTrials.gov at http://clinicaltrials.gov/ct2/show/NCT00015548.^j^The home page for the Neuroscience Information Framework is http://www.neuinfo.org/.^k^We use an Open Source version of Virtuoso http://virtuoso.openlinksw.com/ available as an Ubuntu package.^l^The exact script used for this study is located at https://swat-4-med-safety.googlecode.com/svn/trunk/analyses/pilot-study-of-potential-enhancements-07162012/scripts.^m^The homepage for the Rialto project is http://rialto.improve-technologies.com/wiki/.^o^The Swat-4-med-safety Google Code project is locate at http://swat-4-med-safety.googlecode.com.

## Abbreviations

FDA: Federal drug administration; NDF-RT: National drug file – reference terminology; DIKB: Drug interaction knowledge base; ADE: Adverse drug event; CFR: Code of federal regulations; SPL: Structured product Label; SSRI: Selective serotonin reuptake inhibitor; LOINCⓇ: Logical observation identifiers names and codes.

## Competing interests

The authors acknowledge no conflicts of interest. JRH is author and publisher of drug-interaction reference books including *The Top 100 Drug Interactions: A Guide to Patient Management.*

## Authors’ contributions

RDB conceived of the study, led its design and coordination, and drafted the manuscript. ML, AdW, JS, JRH and MRM made significant contributions to the analysis and interpretation of data. Specifically, ML helped to design and implement the automatic conclusion extraction experiments with SAPIENTA. AdW performed the manual analysis of metabolic pathway claims. JRH helped to develop and apply the criteria for relevance and novelty used to classify conclusions found in the abstracts analyzed in this study. MRM helped to determine the if drug-drug interactions returned from the NDF-RT were previously found in drug product labeling. OH and JSL made significant contributes as well. OH developed LinkedCT, pointed out that study conclusions could be routed through the resource, and helped to revise the draft manuscript. JS and JSL participated in the design and coordination of the study from inception to completion. All authors read and approved the final manuscript.

## Authors’ information

RDB is an Assistant Professor of Biomedical Informatics and a scholar in the University of Pittsburgh Comparative Effectiveness Research Program funded by the Agency for Healthcare Research and Quality. JRH is a Professor of Pharmacy at the University of Washington and a Fellow of the American College of Clinical Pharmacy. He is also one of the founders of the Drug Interaction Foundation that has developed standardized methods of evaluating potential drug interactions and outcome-based criteria for rating the potential significance of drug interactions. OH holds a PhD in Computer Science from University of Toronto, and is currently a Research Staff Member at IBM T.J. Watson Research Center and a research associate at University of Toronto’s database group. AdW is Disruptive Technologies Director at Elsevier Labs. Her scientific discourse analysis work is done in collaboration with the Utrecht University Institute of Linguistics. JS is writing her dissertation on argumentation and semantic web at the Digital Enterprise Research Institute. JSL is a Research Associate Professor at the Tetherless World Constellation, Rensselaer Polytechnic Institute. MRM is a Masters Student in Biomedical Informatics at University of Wisconsin-Milwaukee. ML is an Early Career Leverhulme Trust research fellow with expertise in text mining, natural language processing and computational biology. She is based at the European Bioinformatics Institute (EMBL-EBI) in Cambridge, UK, and also affiliated with Aberystwyth University, UK.

## Supplementary Material

Additional file 1 **Table S4.** The full list of drug-drug interactions (DDIs) affecting drugs indicated for the treatment of depression. The list was created based on a search conducted in the summer of 2011 using a convenience sample of package inserts available at that time. One package insert was retrieved for each of the included antidepressants. Whenever possible, package inserts were retrieved from the Physician’s Desk Reference (PDR). In cases where we could find no relevant package insert in the PDR, one was retrieved from the National Library of Medicine’s DailyMed website. RDB and RG identified statements referring to pharmacokinetic DDIs and pharmacodynamic DDIs. Pharmacokinetic DDIs needed to report a quantitative effect on AUC and/or Cl of an antidepressant. All pharmacodynamic DDIs that could be identified from package insert text were included. Click here for file
