# Supplementary material for: Dynamic enhancement of drug product labels to support drug safety, efficacy, and effectiveness
Source: J Biomed Semantics. 2013 Jan 26;4:5. doi: 10.1186/2041-1480-4-5 (PMC3698101; doi:10.1186/2041-1480-4-5)
Supplement: Additional file 1 — Table S4. The full list of drug-drug interactions (DDIs) affecting drugs indicated for the treatment of depression. The list was created based on a search conducted in the summer of 2011 using a convenience sample of package inserts available at that time. One package insert was retrieved for each of the included antidepressants. Whenever possible, package inserts were retrieved from the Physician’s Desk Reference (PDR). In cases where we could find no relevant package insert in the PDR, one was retrieved from the National Library of Medicine’s DailyMed website. RDB and RG identified statements referring to pharmacokinetic DDIs and pharmacodynamic DDIs. Pharmacokinetic DDIs needed to report a quantitative effect on AUC and/or Cl of an antidepressant. All pharmacodynamic DDIs that could be identified from package insert text were included. [file 2041-1480-4-5-S1.PDF]

Object drug/Class	Precipitant drug/Class	Pharmacokinetic/pharmacodynamic effect	Product label extracted from	As mentioned in the label or based on expansion of the drug class?
Aminoketone	Amantadine	Yes-non-specific adverse experiences	GSK 2011	expansion
Aminoketone	Anticholinergic	Yes-non-specific adverse experiences	GSK 2011	expansion
Aminoketone	Antipsychotic	Yes-seizure	GSK 2010	expansion
Aminoketone	Aripiprazole	Yes-seizure	GSK 2010	expansion
Aminoketone	Asenapine	Yes-seizure	GSK 2010	expansion
Aminoketone	CNS depressants	Yes-non-specific adverse experiences	GSK 2011	expansion
Aminoketone	Chlorpromazine	Yes-seizure	GSK 2010	expansion
Aminoketone	Cimetidine	Yes	GSK 2011	expansion
Aminoketone	Citalopram	No	GSK 2011	expansion
Aminoketone	Clozapine	Yes-seizure	GSK 2010	expansion
Aminoketone	Efavirenz	???	GSK 2011	expansion
Aminoketone	H2 blockers	Yes	GSK 2011	expansion
Aminoketone	Haloperidol	Yes-seizure	GSK 2010	expansion
Aminoketone	Iloperidone	Yes-seizure	GSK 2010	expansion
Aminoketone	Levodopa	Yes-non-specific adverse experiences	GSK 2011	expansion
Aminoketone	Lithium	Yes-seizure	GSK 2010	expansion
Aminoketone	Methylxanthine	Yes-seizure	GSK 2010	expansion
Aminoketone	Olanzapine	Yes-seizure	GSK 2010	expansion
Aminoketone	Paliperidone	Yes-seizure	GSK 2010	expansion
Aminoketone	Perphenazine	Yes-seizure	GSK 2010	expansion
Aminoketone	Pimozide	Yes-seizure	GSK 2010	expansion
Aminoketone	Protease Inhibitor	???	GSK 2011	expansion
Aminoketone	Quetiapine	Yes-seizure	GSK 2010	expansion
Aminoketone	Risperidone	Yes-seizure	GSK 2010	expansion
Aminoketone	Ritonavir	???	GSK 2011	expansion
Aminoketone	SSRI	No	GSK 2011	expansion
Aminoketone	Theophylline	Yes-seizure	GSK 2010	expansion
Aminoketone	Thioridazine	Yes-seizure	GSK 2010	expansion
Aminoketone	Thioridizine	Yes-seizure	GSK 2010	expansion
Aminoketone	Thiothixene	Yes-seizure	GSK 2010	expansion
Aminoketone	Ziprasidone	Yes-seizure	GSK 2010	expansion
Amitriptyline	Alprazolam	Yes- Neuroleptic Malignant Syndrome	Mylan 2009	expansion
Amitriptyline	Amantadine	Yes-Hyperpyrexia, Paralytic ileus	Mylan 2009	expansion
Amitriptyline	Amitriptyline	Yes-serotonin syndrome	Mylan 2009	expansion
Amitriptyline	Amoxapine	Yes-serotonin syndrome	Mylan 2009	expansion
Amitriptyline	Anticholinergic	Yes-Hyperpyrexia, Paralytic ileus	Mylan 2009	original
Amitriptyline	Antipsychotic	Yes- Neuroleptic Malignant Syndrome	Mylan 2009	original
Amitriptyline	Aripiprazole	Yes- Neuroleptic Malignant Syndrome	Mylan 2009	expansion
Amitriptyline	Asenapine	Yes- Neuroleptic Malignant Syndrome	Mylan 2009	expansion
Amitriptyline	Atropine	Yes-Hyperpyrexia, Paralytic ileus	Mylan 2009	expansion
Amitriptyline	Benzodiazepine	Yes- Neuroleptic Malignant Syndrome	Mylan 2009	original
Amitriptyline	Benztropine	Yes-Hyperpyrexia, Paralytic ileus	Mylan 2009	expansion
Amitriptyline	Biperiden	Yes-Hyperpyrexia, Paralytic ileus	Mylan 2009	expansion
Amitriptyline	Buspirone	Yes-serotonin syndrome	Mylan 2009	original
Amitriptyline	Chlorpromazine	Yes- Neuroleptic Malignant Syndrome	Mylan 2009	expansion
Amitriptyline	Citalopram	Yes-serotonin syndrome	Mylan 2009	expansion
Amitriptyline	Clomipramine	Yes-serotonin syndrome	Mylan 2009	expansion
Amitriptyline	Clozapine	Yes- Neuroleptic Malignant Syndrome	Mylan 2009	expansion
Amitriptyline	Desipramine	Yes-serotonin syndrome	Mylan 2009	expansion
Amitriptyline	Desvenlafaxine	Yes-serotonin syndrome	Mylan 2009	expansion
Amitriptyline	Dextromethorphan	Yes-serotonin syndrome	Mylan 2009	original
Amitriptyline	Diazepam	Yes- Neuroleptic Malignant Syndrome	Mylan 2009	expansion
Amitriptyline	Dicyclomine	Yes-Hyperpyrexia, Paralytic ileus	Mylan 2009	expansion
Amitriptyline	Difenoxin	Yes-Hyperpyrexia, Paralytic ileus	Mylan 2009	expansion

Amitriptyline	Diphenoxylate	Yes-Hyperpyrexia, Paralytic ileus	Mylan 2009	expansion
Amitriptyline	Doxepin	Yes-serotonin syndrome	Mylan 2009	expansion
Amitriptyline	Duloxetine	Yes-serotonin syndrome	Mylan 2009	expansion
Amitriptyline	Escitalopram	Yes-serotonin syndrome	Mylan 2009	expansion
Amitriptyline	Fentanyl	Yes-serotonin syndrome	Mylan 2009	original
Amitriptyline	Fluoxetine	Yes-serotonin syndrome	Mylan 2009	expansion
Amitriptyline	Fluvoxamine	Yes-serotonin syndrome	Mylan 2009	expansion
Amitriptyline	Glycopyrronium	Yes-Hyperpyrexia, Paralytic ileus	Mylan 2009	expansion
Amitriptyline	Haloperidol	Yes- Neuroleptic Malignant Syndrome	Mylan 2009	expansion
Amitriptyline	Hyoscyamine	Yes-Hyperpyrexia, Paralytic ileus	Mylan 2009	expansion
Amitriptyline	Iloperidone	Yes- Neuroleptic Malignant Syndrome	Mylan 2009	expansion
Amitriptyline	Imipramine	Yes-serotonin syndrome	Mylan 2009	expansion
Amitriptyline	Ipratropium cation	Yes-Hyperpyrexia, Paralytic ileus	Mylan 2009	expansion
Amitriptyline	Ipratropium	Yes-Hyperpyrexia, Paralytic ileus	Mylan 2009	expansion
Amitriptyline	Isocarboxazid	Yes-Hyperpyretic crises, severe convulsions, death	Mylan 2009, Sandoz 2010, Watson 2007 (doxepin), Mylan 2007, Sandoz 2010b, Watson 2009, Duramed 2007, Ciba-Gigy 1996	expansion
Amitriptyline	Isocarboxazid	Yes-serotonin syndrome	Mylan 2009	expansion
Amitriptyline	Linezolid	Yes-serotonin syndrome	Mylan 2009	original
Amitriptyline	Lithium	Yes- Neuroleptic Malignant Syndrome	Mylan 2009	expansion
Amitriptyline	Lorazepam	Yes- Neuroleptic Malignant Syndrome	Mylan 2009	expansion
Amitriptyline	MAOI	Yes-serotonin syndrome	Mylan 2009	original
Amitriptyline	Maprotiline	Yes-serotonin syndrome	Mylan 2009	expansion
Amitriptyline	Mepenzolate	Yes-Hyperpyrexia, Paralytic ileus	Mylan 2009	expansion
Amitriptyline	Meperidine	Yes-serotonin syndrome	Mylan 2009	original
Amitriptyline	Methscopolamine	Yes-Hyperpyrexia, Paralytic ileus	Mylan 2009	expansion
Amitriptyline	Milnacipran	Yes-serotonin syndrome	Mylan 2009	expansion
Amitriptyline	Nefazodone	Yes-serotonin syndrome	Mylan 2009	expansion
Amitriptyline	Nortriptyline	Yes-serotonin syndrome	Mylan 2009	expansion
Amitriptyline	Odansetran	Yes-serotonin syndrome	Mylan 2009	original
Amitriptyline	Olanzapine	Yes- Neuroleptic Malignant Syndrome	Mylan 2009	expansion
Amitriptyline	Paliperidone	Yes- Neuroleptic Malignant Syndrome	Mylan 2009	expansion
Amitriptyline	Paroxetine	Yes-serotonin syndrome	Mylan 2009	expansion
Amitriptyline	Pentazocine	Yes-serotonin syndrome	Mylan 2009	original
Amitriptyline	Perphenazine	Yes- Neuroleptic Malignant Syndrome	Mylan 2009	expansion
Amitriptyline	Phenelzine	Yes-Hyperpyretic crises, severe convulsions, death	Mylan 2009, Sandoz 2010, Watson 2007 (doxepin), Mylan 2007, Sandoz 2010b, Watson 2009, Duramed 2007, Ciba-Gigy 1996	expansion
Amitriptyline	Phenelzine	Yes-serotonin syndrome	Mylan 2009	expansion
Amitriptyline	Phenobarbital	Yes-Hyperpyrexia, Paralytic ileus	Mylan 2009	expansion
Amitriptyline	Pimozide	Yes- Neuroleptic Malignant Syndrome	Mylan 2009	expansion
Amitriptyline	Procyclidine	Yes-Hyperpyrexia, Paralytic ileus	Mylan 2009	expansion
Amitriptyline	Propantheline	Yes-Hyperpyrexia, Paralytic ileus	Mylan 2009	expansion
Amitriptyline	Protriptyline	Yes-serotonin syndrome	Mylan 2009	expansion
Amitriptyline	Pseudoephedrine	Yes-Hyperpyrexia, Paralytic ileus	Mylan 2009	expansion
Amitriptyline	Quetiapine	Yes- Neuroleptic Malignant Syndrome	Mylan 2009	expansion

Amitriptyline	Rasagiline	Yes-Hyperpyretic crises, severe convulsions, death	Mylan 2009, Sandoz 2010, Watson 2007 (doxepin), Mylan 2007, Sandoz 2010b, Watson 2009, Duramed 2007, Ciba-Gigy 1996	expansion
Amitriptyline	Rasagiline	Yes-serotonin syndrome	Mylan 2009	expansion
Amitriptyline	Risperidone	Yes- Neuroleptic Malignant Syndrome	Mylan 2009	expansion
Amitriptyline	SEROTONIN-1B AND SERC	Yes-serotonin syndrome	Mylan 2009	expansion
Amitriptyline	SNRI	Yes-serotonin syndrome	Mylan 2009	original
Amitriptyline	SSRI	Yes-serotonin syndrome	Mylan 2009	original
Amitriptyline	Scopolamine	Yes-Hyperpyrexia, Paralytic ileus	Mylan 2009	expansion
Amitriptyline	Selegiline	Yes-Hyperpyretic crises, severe convulsions, death	Mylan 2009, Sandoz 2010, Watson 2007 (doxepin), Mylan 2007, Sandoz 2010b, Watson 2009, Duramed 2007, Ciba-Gigy 1996	expansion
Amitriptyline	Selegiline	Yes-serotonin syndrome	Mylan 2009	expansion
Amitriptyline	Sertraline	Yes-serotonin syndrome	Mylan 2009	expansion
Amitriptyline	St Johns wort	Yes-serotonin syndrome	Mylan 2009	original
Amitriptyline	Sumatriptan	Yes-serotonin syndrome	Mylan 2009	original
Amitriptyline	Sympathomimetic	Yes-serotonin syndrome	Mylan 2009	expansion
Amitriptyline	TCA	Yes-serotonin syndrome	Mylan 2009	original
Amitriptyline	Temazepam	Yes- Neuroleptic Malignant Syndrome	Mylan 2009	expansion
Amitriptyline	Thioridazine	Yes- Neuroleptic Malignant Syndrome	Mylan 2009	expansion
Amitriptyline	Thioridizine	Yes- Neuroleptic Malignant Syndrome	Mylan 2009	expansion
Amitriptyline	Thiothixene	Yes- Neuroleptic Malignant Syndrome	Mylan 2009	expansion
Amitriptyline	Tiotropium	Yes-Hyperpyrexia, Paralytic ileus	Mylan 2009	expansion
Amitriptyline	Tramadol	Yes-serotonin syndrome	Mylan 2009	original
Amitriptyline	Tranylcypromine	Yes-Hyperpyretic crises, severe convulsions, death	Mylan 2009, Sandoz 2010, Watson 2007 (doxepin), Mylan 2007, Sandoz 2010b, Watson 2009, Duramed 2007, Ciba-Gigy 1996	expansion
Amitriptyline	Tranylcypromine	Yes-serotonin syndrome	Mylan 2009	expansion
Amitriptyline	Trazodone	Yes-serotonin syndrome	Mylan 2009	expansion
Amitriptyline	Trimipramine	Yes-serotonin syndrome	Mylan 2009	expansion
Amitriptyline	Tropicamide	Yes-Hyperpyrexia, Paralytic ileus	Mylan 2009	expansion
Amitriptyline	Tryptophan	Yes-serotonin syndrome	Mylan 2009	original
Amitriptyline	Venlafaxine	Yes-serotonin syndrome	Mylan 2009	expansion
Amitriptyline	Vilazodone	Yes-serotonin syndrome	Mylan 2009	expansion
Amitriptyline	Ziprasidone	Yes- Neuroleptic Malignant Syndrome	Mylan 2009	expansion
Amitriptyline	chlordiazepoxide	Yes- Neuroleptic Malignant Syndrome	Mylan 2009	expansion
Amitriptyline	clonazepam	Yes- Neuroleptic Malignant Syndrome	Mylan 2009	expansion
Amitriptyline	clorazepic acid	Yes- Neuroleptic Malignant Syndrome	Mylan 2009	expansion
Amitriptyline	estazolam	Yes- Neuroleptic Malignant Syndrome	Mylan 2009	expansion
Amitriptyline	flurazepam	Yes- Neuroleptic Malignant Syndrome	Mylan 2009	expansion
Amitriptyline	midazolam	Yes- Neuroleptic Malignant Syndrome	Mylan 2009	expansion

Amitriptyline	oxazepam	Yes- Neuroleptic Malignant Syndrome	Mylan 2009	expansion
Amoxapine	4-hydroxybutanoic acid	Yes-additive CNS depressant effects	Watson 2009	expansion
Amoxapine	Amantadine	Yes-Paralytic ileus	Watson 2009	expansion
Amoxapine	Anticholinergic	Yes-Paralytic ileus	Watson 2009	original
Amoxapine	Atropine	Yes-Paralytic ileus	Watson 2009	expansion
Amoxapine	Benztropine	Yes-Paralytic ileus	Watson 2009	expansion
Amoxapine	Biperiden	Yes-Paralytic ileus	Watson 2009	expansion
Amoxapine	CNS depressants	Yes-additive CNS depressant effects	Watson 2009	original
Amoxapine	Dicyclomine	Yes-Paralytic ileus	Watson 2009	expansion
Amoxapine	Difenoxin	Yes-Paralytic ileus	Watson 2009	expansion
Amoxapine	Diphenoxylate	Yes-Paralytic ileus	Watson 2009	expansion
Amoxapine	Glycopyrronium	Yes-Paralytic ileus	Watson 2009	expansion
Amoxapine	Hyoscyamine	Yes-Paralytic ileus	Watson 2009	expansion
Amoxapine	Ipratropium cation	Yes-Paralytic ileus	Watson 2009	expansion
Amoxapine	Ipratropium	Yes-Paralytic ileus	Watson 2009	expansion
Amoxapine	Isocarboxazid	Yes-Hyperpyretic crises, severe convulsions, death	Mylan 2009, Sandoz 2010, Watson 2007 (doxepin), Mylan 2007, Sandoz 2010b, Watson 2009, Duramed 2007, Ciba-Gigy 1996	expansion
Amoxapine	Levodopa	Yes-additive CNS depressant effects	Watson 2009	expansion
Amoxapine	Mepenzolate	Yes-Paralytic ileus	Watson 2009	expansion
Amoxapine	Methscopolamine	Yes-Paralytic ileus	Watson 2009	expansion
Amoxapine	Methylphenidate	Yes-additive CNS depressant effects	Watson 2009	expansion
Amoxapine	Phenelzine	Yes-Hyperpyretic crises, severe convulsions, death	Mylan 2009, Sandoz 2010, Watson 2007 (doxepin), Mylan 2007, Sandoz 2010b, Watson 2009, Duramed 2007, Ciba-Gigy 1996	expansion
Amoxapine	Phenobarbital	Yes-Paralytic ileus	Watson 2009	expansion
Amoxapine	Procyclidine	Yes-Paralytic ileus	Watson 2009	expansion
Amoxapine	Propantheline	Yes-Paralytic ileus	Watson 2009	expansion
Amoxapine	Pseudoephedrine	Yes-Paralytic ileus	Watson 2009	expansion
Amoxapine	Rasagiline	Yes-Hyperpyretic crises, severe convulsions, death	Mylan 2009, Sandoz 2010, Watson 2007 (doxepin), Mylan 2007, Sandoz 2010b, Watson 2009, Duramed 2007, Ciba-Gigy 1996	expansion
Amoxapine	Scopolamine	Yes-Paralytic ileus	Watson 2009	expansion

Amoxapine	Selegiline	Yes-Hyperpyretic crises, severe convulsions, death	Mylan 2009, Sandoz 2010, Watson 2007 (doxepin), Mylan 2007, Sandoz 2010b, Watson 2009, Duramed 2007, Ciba-Gigy 1996	expansion
Amoxapine	Tiotropium	Yes-Paralytic ileus	Watson 2009	expansion
Amoxapine	Tranylcypromine	Yes-Hyperpyretic crises, severe convulsions, death	Mylan 2009, Sandoz 2010, Watson 2007 (doxepin), Mylan 2007, Sandoz 2010b, Watson 2009, Duramed 2007, Ciba-Gigy 1996	expansion
Amoxapine	Tropicamide	Yes-Paralytic ileus	Watson 2009	expansion
Bupropion	Amantadine	Yes-non-specific adverse experiences	GSK 2011	original
Bupropion	Anticholinergic	Yes-non-specific adverse experiences	GSK 2011	expansion
Bupropion	Antipsychotic	Yes-seizure	GSK 2010	original
Bupropion	Aripiprazole	Yes-seizure	GSK 2010	expansion
Bupropion	Asenapine	Yes-seizure	GSK 2010	expansion
Bupropion	CNS depressants	Yes-non-specific adverse experiences	GSK 2011	expansion
Bupropion	Chlorpromazine	Yes-seizure	GSK 2010	expansion
Bupropion	Cimetidine	Yes	GSK 2011	original
Bupropion	Citalopram	No	GSK 2011	original
Bupropion	Clozapine	Yes-seizure	GSK 2010	expansion
Bupropion	Efavirenz	???	GSK 2011	original
Bupropion	H2 blockers	Yes	GSK 2011	expansion
Bupropion	Haloperidol	Yes-seizure	GSK 2010	expansion
Bupropion	Iloperidone	Yes-seizure	GSK 2010	expansion
Bupropion	Levodopa	Yes-non-specific adverse experiences	GSK 2011	original
Bupropion	Lithium	Yes-seizure	GSK 2010	expansion
Bupropion	Methylxanthine	Yes-seizure	GSK 2010	expansion
Bupropion	Olanzapine	Yes-seizure	GSK 2010	expansion
Bupropion	Paliperidone	Yes-seizure	GSK 2010	expansion
Bupropion	Perphenazine	Yes-seizure	GSK 2010	expansion
Bupropion	Pimozide	Yes-seizure	GSK 2010	expansion
Bupropion	Protease Inhibitor	???	GSK 2011	expansion
Bupropion	Quetiapine	Yes-seizure	GSK 2010	expansion
Bupropion	Risperidone	Yes-seizure	GSK 2010	expansion
Bupropion	Ritonavir	???	GSK 2011	original
Bupropion	SSRI	No	GSK 2011	expansion
Bupropion	Theophylline	Yes-seizure	GSK 2010	original
Bupropion	Thioridazine	Yes-seizure	GSK 2010	expansion
Bupropion	Thioridizine	Yes-seizure	GSK 2010	expansion
Bupropion	Thiothixene	Yes-seizure	GSK 2010	expansion
Bupropion	Ziprasidone	Yes-seizure	GSK 2010	expansion
CNS depressants	Caffeine	Yes-behavior disturbance and thought disorder	Physicians 2009	expansion
CNS depressants	Chlorpheniramine	Yes-behavior disturbance and thought disorder	Physicians 2009	expansion
CNS depressants	Dexmethylphenidate	Yes-behavior disturbance and thought disorder	Physicians 2009	expansion
CNS depressants	Dextroamphetamine	Yes-behavior disturbance and thought disorder	Physicians 2009	expansion
CNS depressants	Isocarboxazid	Yes-hypertension	Physicians 2009	expansion
CNS depressants	Lisdexamfetamine	Yes-behavior disturbance and thought disorder	Physicians 2009	expansion
CNS depressants	MAOI	Yes-hypertension	Physicians 2009	expansion
CNS depressants	Methamphetamine	Yes-behavior disturbance and thought disorder	Physicians 2009	expansion

CNS depressants	Phenelzine	Yes-hypertension	Physicians 2009	expansion
CNS depressants	Rasagiline	Yes-hypertension	Physicians 2009	expansion
CNS depressants	Selegiline	Yes-hypertension	Physicians 2009	expansion
CNS depressants	Stimulant	Yes-behavior disturbance and thought disorder	Physicians 2009	expansion
CNS depressants	Tranylcypromine	Yes-hypertension	Physicians 2009	expansion
Citalopram	Acetaminophen	Yes-bleeding	Forest 2009, Forest 2011, Dista 2009, Alphapharm 2007, Watson 2008, Forest 2011B	expansion
Citalopram	Aminoketone	Yes-bleeding	Forest 2009, Forest 2011, Dista 2009, Alphapharm 2007, Watson 2008, Forest 2011B	expansion
Citalopram	Amitriptyline	Yes-serotonin syndrome	Forest 2009, Forest 2011, Dista 2009, Alphapharm 2007, Watson 2008, Forest 2011B (Vilazodone)	expansion
Citalopram	Amoxapine	Yes-serotonin syndrome	Forest 2009, Forest 2011, Dista 2009, Alphapharm 2007, Watson 2008, Forest 2011B (Vilazodone)	expansion
Citalopram	Antipsychotic	No	Forest 2009, Forest 2011	expansion
Citalopram	Antipsychotic	Yes-QTc interval prolongation	Forest 2009, Forest 2011, Dista 2009, Alphapharm 2007, Watson 2008	expansion
Citalopram	Antithrombin alfa	Yes-bleeding	Forest 2009, Forest 2011, Dista 2009, Alphapharm 2007, Watson 2008, Forest 2011B	expansion
Citalopram	Argatroban	Yes-bleeding	Forest 2009, Forest 2011, Dista 2009, Alphapharm 2007, Watson 2008, Forest 2011B	expansion

Citalopram	Aspirin	Yes-bleeding	Forest 2009, Forest 2011, Dista 2009, Alphapharm 2007, Watson 2008, Forest 2011B	expansion
Citalopram	Azole Antifungal	No	Forest 2009, Forest 2011	expansion
Citalopram	Bivalirudin	Yes-bleeding	Forest 2009, Forest 2011, Dista 2009, Alphapharm 2007, Watson 2008, Forest 2011B	expansion
Citalopram	Bromfenac	Yes-bleeding	Forest 2009, Forest 2011, Dista 2009, Alphapharm 2007, Watson 2008, Forest 2011B	expansion
Citalopram	Bupropion	Yes-bleeding	Forest 2009, Forest 2011, Dista 2009, Alphapharm 2007, Watson 2008, Forest 2011B	expansion
Citalopram	Buspirone	Yes-serotonin syndrome	Forest 2009, Forest 2011, Dista 2009, Alphapharm 2007, Watson 2008, Forest 2011B (Vilazodone)	expansion
Citalopram	Cardiac Glycoside	No	Forest 2009, Forest 2011	expansion
Citalopram	Celecoxib	Yes-bleeding	Forest 2009, Forest 2011, Dista 2009, Alphapharm 2007, Watson 2008, Forest 2011B	expansion
Citalopram	Cimetidine	Yes	Forest 2009, Forest 2011	original
Citalopram	Citalopram	Yes-serotonin syndrome	Forest 2009, Forest 2011, Dista 2009, Alphapharm 2007, Watson 2008, Forest 2011B (Vilazodone)	expansion

Citalopram	Clomipramine	Yes-serotonin syndrome	Forest 2009, Forest 2011, Dista 2009, Alphapharm 2007, Watson 2008, Forest 2011B (Vilazodone)	expansion
Citalopram	Desipramine	Yes-serotonin syndrome	Forest 2009, Forest 2011, Dista 2009, Alphapharm 2007, Watson 2008, Forest 2011B (Vilazodone)	expansion
Citalopram	Desirudin	Yes-bleeding	Forest 2009, Forest 2011, Dista 2009, Alphapharm 2007, Watson 2008, Forest 2011B	expansion
Citalopram	Desvenlafaxine	Yes-serotonin syndrome	Forest 2009, Forest 2011, Dista 2009, Alphapharm 2007, Watson 2008, Forest 2011B (Vilazodone)	expansion
Citalopram	Dextromethorphan	Yes-serotonin syndrome	Forest 2009, Forest 2011, Dista 2009, Alphapharm 2007, Watson 2008, Forest 2011B (Vilazodone)	expansion
Citalopram	Diclofenac	Yes-bleeding	Forest 2009, Forest 2011, Dista 2009, Alphapharm 2007, Watson 2008, Forest 2011B	expansion
Citalopram	Diflunisal	Yes-bleeding	Forest 2009, Forest 2011, Dista 2009, Alphapharm 2007, Watson 2008, Forest 2011B	expansion
Citalopram	Digoxin	No	Forest 2009, Forest 2011	original

Citalopram	Doxepin	Yes-serotonin syndrome	Forest 2009, Forest 2011, Dista 2009, Alphapharm 2007, Watson 2008, Forest 2011B (Vilazodone)	expansion
Citalopram	Duloxetine	Yes-serotonin syndrome	Forest 2009, Forest 2011, Dista 2009, Alphapharm 2007, Watson 2008, Forest 2011B (Vilazodone)	expansion
Citalopram	Escitalopram	Yes-serotonin syndrome	Forest 2009, Forest 2011, Dista 2009, Alphapharm 2007, Watson 2008, Forest 2011B (Vilazodone)	expansion
Citalopram	Etodolac	Yes-bleeding	Forest 2009, Forest 2011, Dista 2009, Alphapharm 2007, Watson 2008, Forest 2011B	expansion
Citalopram	Fenoprofen	Yes-bleeding	Forest 2009, Forest 2011, Dista 2009, Alphapharm 2007, Watson 2008, Forest 2011B	expansion
Citalopram	Fentanyl	Yes-serotonin syndrome	Forest 2009, Forest 2011, Dista 2009, Alphapharm 2007, Watson 2008, Forest 2011B (Vilazodone)	expansion
Citalopram	Fluoxetine	Yes-serotonin syndrome	Forest 2009, Forest 2011, Dista 2009, Alphapharm 2007, Watson 2008, Forest 2011B (Vilazodone)	expansion

Citalopram	Flurbiprofen	Yes-bleeding	Forest 2009, Forest 2011, Dista 2009, Alphapharm 2007, Watson 2008, Forest 2011B	expansion
Citalopram	Fluvoxamine	Yes-serotonin syndrome	Forest 2009, Forest 2011, Dista 2009, Alphapharm 2007, Watson 2008, Forest 2011B (Vilazodone)	expansion
Citalopram	H2 blockers	Yes	Forest 2009, Forest 2011	expansion
Citalopram	Heparin	Yes-bleeding	Forest 2009, Forest 2011, Dista 2009, Alphapharm 2007, Watson 2008, Forest 2011B	expansion
Citalopram	Ibuprofen	Yes-bleeding	Forest 2009, Forest 2011, Dista 2009, Alphapharm 2007, Watson 2008, Forest 2011B	expansion
Citalopram	Imipramine	Yes-serotonin syndrome	Forest 2009, Forest 2011, Dista 2009, Alphapharm 2007, Watson 2008, Forest 2011B (Vilazodone)	expansion
Citalopram	Indomethacin	Yes-bleeding	Forest 2009, Forest 2011, Dista 2009, Alphapharm 2007, Watson 2008, Forest 2011B	expansion
Citalopram	Isocarboxazid	Yes-serotonin syndrome	Forest 2009, Forest 2011, Dista 2009, Alphapharm 2007, Watson 2008, Forest 2011B (Vilazodone)	expansion
Citalopram	Ketoconazole	No	Forest 2009, Forest 2011	original

Citalopram	Ketoprofen	Yes-bleeding	Forest 2009, Forest 2011, Dista 2009, Alphapharm 2007, Watson 2008, Forest 2011B	expansion
Citalopram	Ketorolac tromethamine	Yes-bleeding	Forest 2009, Forest 2011, Dista 2009, Alphapharm 2007, Watson 2008, Forest 2011B	expansion
Citalopram	Ketorolac	Yes-bleeding	Forest 2009, Forest 2011, Dista 2009, Alphapharm 2007, Watson 2008, Forest 2011B	expansion
Citalopram	Lepirudin	Yes-bleeding	Forest 2009, Forest 2011, Dista 2009, Alphapharm 2007, Watson 2008, Forest 2011B	expansion
Citalopram	Linezolid	Yes-serotonin syndrome	Forest 2009, Forest 2011, Dista 2009, Alphapharm 2007, Watson 2008, Forest 2011B (Vilazodone)	expansion
Citalopram	Lithium	No	Forest 2009, Forest 2011	original
Citalopram	Mannitol	Yes-bleeding	Forest 2009, Forest 2011, Dista 2009, Alphapharm 2007, Watson 2008, Forest 2011B	expansion
Citalopram	Maprotiline	Yes-serotonin syndrome	Forest 2009, Forest 2011, Dista 2009, Alphapharm 2007, Watson 2008, Forest 2011B (Vilazodone)	expansion

Citalopram	Mefenamic acid	Yes-bleeding	Forest 2009, Forest 2011, Dista 2009, Alphapharm 2007, Watson 2008, Forest 2011B	expansion
Citalopram	Meloxicam	Yes-bleeding	Forest 2009, Forest 2011, Dista 2009, Alphapharm 2007, Watson 2008, Forest 2011B	expansion
Citalopram	Meperidine	Yes-serotonin syndrome	Forest 2009, Forest 2011, Dista 2009, Alphapharm 2007, Watson 2008, Forest 2011B (Vilazodone)	expansion
Citalopram	Milnacipran	Yes-serotonin syndrome	Forest 2009, Forest 2011, Dista 2009, Alphapharm 2007, Watson 2008, Forest 2011B (Vilazodone)	expansion
Citalopram	NSAID	Yes-bleeding	Forest 2009, Forest 2011, Dista 2009, Alphapharm 2007, Watson 2008, Forest 2011B	expansion
Citalopram	Nabumetone	Yes-bleeding	Forest 2009, Forest 2011, Dista 2009, Alphapharm 2007, Watson 2008, Forest 2011B	expansion
Citalopram	Naproxen	Yes-bleeding	Forest 2009, Forest 2011, Dista 2009, Alphapharm 2007, Watson 2008, Forest 2011B	expansion
Citalopram	Nefazodone	Yes-serotonin syndrome	Forest 2009, Forest 2011, Dista 2009, Alphapharm 2007, Watson 2008, Forest 2011B (Vilazodone)	expansion

Citalopram	Nepafenac	Yes-bleeding	Forest 2009, Forest 2011, Dista 2009, Alphapharm 2007, Watson 2008, Forest 2011B	expansion
Citalopram	Nortriptyline	Yes-serotonin syndrome	Forest 2009, Forest 2011, Dista 2009, Alphapharm 2007, Watson 2008, Forest 2011B (Vilazodone)	expansion
Citalopram	Odansetran	Yes-serotonin syndrome	Forest 2009, Forest 2011, Dista 2009, Alphapharm 2007, Watson 2008, Forest 2011B (Vilazodone)	expansion
Citalopram	Oxaprozin	Yes-bleeding	Forest 2009, Forest 2011, Dista 2009, Alphapharm 2007, Watson 2008, Forest 2011B	expansion
Citalopram	Paroxetine	Yes-serotonin syndrome	Forest 2009, Forest 2011, Dista 2009, Alphapharm 2007, Watson 2008, Forest 2011B (Vilazodone)	expansion
Citalopram	Pentazocine	Yes-serotonin syndrome	Forest 2009, Forest 2011, Dista 2009, Alphapharm 2007, Watson 2008, Forest 2011B (Vilazodone)	expansion
Citalopram	Phenelzine	Yes-serotonin syndrome	Forest 2009, Forest 2011, Dista 2009, Alphapharm 2007, Watson 2008, Forest 2011B (Vilazodone)	expansion

Citalopram	Pimozide	Yes-QTc interval prolongation	Forest 2009, Forest 2011, Dista 2009, Alphapharm 2007, Watson 2008	expansion
Citalopram	Piroxicam	Yes-bleeding	Forest 2009, Forest 2011, Dista 2009, Alphapharm 2007, Watson 2008, Forest 2011B	expansion
Citalopram	Protriptyline	Yes-serotonin syndrome	Forest 2009, Forest 2011, Dista 2009, Alphapharm 2007, Watson 2008, Forest 2011B (Vilazodone)	expansion
Citalopram	Rasagiline	Yes-serotonin syndrome	Forest 2009, Forest 2011, Dista 2009, Alphapharm 2007, Watson 2008, Forest 2011B (Vilazodone)	expansion
Citalopram	SEROTONIN-1B AND SERC	Yes-serotonin syndrome	Forest 2009, Forest 2011, Dista 2009, Alphapharm 2007, Watson 2008, Forest 2011B (Vilazodone)	expansion
Citalopram	SEROTONIN-1B AND SERC	Yes-weakness, hyperreflexia, and incoordination	Forest 2009, Forest 2011, Watson 2008	expansion
Citalopram	Salicylamide	Yes-bleeding	Forest 2009, Forest 2011, Dista 2009, Alphapharm 2007, Watson 2008, Forest 2011B	expansion
Citalopram	Sedative Hypnotics	No	Forest 2009, Forest 2011	expansion
Citalopram	Selegiline	Yes-serotonin syndrome	Forest 2009, Forest 2011, Dista 2009, Alphapharm 2007, Watson 2008, Forest 2011B (Vilazodone)	expansion

Citalopram	Sertraline	Yes-serotonin syndrome	Forest 2009, Forest 2011, Dista 2009, Alphapharm 2007, Watson 2008, Forest 2011B (Vilazodone)	expansion
Citalopram	St Johns wort	Yes-serotonin syndrome	Forest 2009, Forest 2011, Dista 2009, Alphapharm 2007, Watson 2008, Forest 2011B (Vilazodone)	expansion
Citalopram	Sulindac	Yes-bleeding	Forest 2009, Forest 2011, Dista 2009, Alphapharm 2007, Watson 2008, Forest 2011B	expansion
Citalopram	Sumatriptan	Yes-serotonin syndrome	Forest 2009, Forest 2011, Dista 2009, Alphapharm 2007, Watson 2008, Forest 2011B (Vilazodone)	expansion
Citalopram	Sumatriptan	Yes-weakness, hyperreflexia, and incoordination	Forest 2009, Forest 2011, Watson 2008	expansion
Citalopram	Sympathomimetic	Yes-serotonin syndrome	Forest 2009, Forest 2011, Dista 2009, Alphapharm 2007, Watson 2008, Forest 2011B (Vilazodone)	expansion
Citalopram	Tolmetin	Yes-bleeding	Forest 2009, Forest 2011, Dista 2009, Alphapharm 2007, Watson 2008, Forest 2011B	expansion
Citalopram	Tramadol	Yes-serotonin syndrome	Forest 2009, Forest 2011, Dista 2009, Alphapharm 2007, Watson 2008, Forest 2011B (Vilazodone)	expansion

Citalopram	Tranylcypromine	Yes-serotonin syndrome	Forest 2009, Forest 2011, Dista 2009, Alphapharm 2007, Watson 2008, Forest 2011B (Vilazodone)	expansion
Citalopram	Trazodone	Yes-serotonin syndrome	Forest 2009, Forest 2011, Dista 2009, Alphapharm 2007, Watson 2008, Forest 2011B (Vilazodone)	expansion
Citalopram	Triazolam	No	Forest 2009, Forest 2011	original
Citalopram	Trimipramine	Yes-serotonin syndrome	Forest 2009, Forest 2011, Dista 2009, Alphapharm 2007, Watson 2008, Forest 2011B (Vilazodone)	expansion
Citalopram	Tryptophan	Yes-serotonin syndrome	Forest 2009, Forest 2011, Dista 2009, Alphapharm 2007, Watson 2008, Forest 2011B (Vilazodone)	expansion
Citalopram	VITAMIN K ANTAGONIST	Yes-bleeding	Forest 2009, Forest 2011, Dista 2009, Alphapharm 2007, Watson 2008, Forest 2011B	expansion
Citalopram	Venlafaxine	Yes-serotonin syndrome	Forest 2009, Forest 2011, Dista 2009, Alphapharm 2007, Watson 2008, Forest 2011B (Vilazodone)	expansion
Citalopram	Vilazodone	Yes-serotonin syndrome	Forest 2009, Forest 2011, Dista 2009, Alphapharm 2007, Watson 2008, Forest 2011B (Vilazodone)	expansion

Citalopram	Warfarin	Yes-bleeding	Forest 2009, Forest 2011, Dista 2009, Alphapharm 2007, Watson 2008, Forest 2011B	expansion
Clomipramine	Isocarboxazid	Yes-Hyperpyretic crises, severe convulsions, death	Mylan 2009, Sandoz 2010, Watson 2007 (doxepin), Mylan 2007, Sandoz 2010b, Watson 2009, Duramed 2007, Ciba-Gigy 1996	expansion
Clomipramine	Phenelzine	Yes-Hyperpyretic crises, severe convulsions, death	Mylan 2009, Sandoz 2010, Watson 2007 (doxepin), Mylan 2007, Sandoz 2010b, Watson 2009, Duramed 2007, Ciba-Gigy 1996	expansion
Clomipramine	Rasagiline	Yes-Hyperpyretic crises, severe convulsions, death	Mylan 2009, Sandoz 2010, Watson 2007 (doxepin), Mylan 2007, Sandoz 2010b, Watson 2009, Duramed 2007, Ciba-Gigy 1996	expansion
Clomipramine	Selegiline	Yes-Hyperpyretic crises, severe convulsions, death	Mylan 2009, Sandoz 2010, Watson 2007 (doxepin), Mylan 2007, Sandoz 2010b, Watson 2009, Duramed 2007, Ciba-Gigy 1996	expansion
Clomipramine	Tranylcypromine	Yes-Hyperpyretic crises, severe convulsions, death	Mylan 2009, Sandoz 2010, Watson 2007 (doxepin), Mylan 2007, Sandoz 2010b, Watson 2009, Duramed 2007, Ciba-Gigy 1996	expansion
Desipramine	Amantadine	Yes-Additive side-effects	Sandoz 2010	expansion
Desipramine	Anticholinergic	Yes-Additive side-effects	Sandoz 2010	original
Desipramine	Atropine	Yes-Additive side-effects	Sandoz 2010	expansion
Desipramine	Benztropine	Yes-Additive side-effects	Sandoz 2010	expansion
Desipramine	Biperiden	Yes-Additive side-effects	Sandoz 2010	expansion
Desipramine	Dicyclomine	Yes-Additive side-effects	Sandoz 2010	expansion
Desipramine	Difenoxin	Yes-Additive side-effects	Sandoz 2010	expansion

Desipramine	Diphenoxylate	Yes-Additive side-effects	Sandoz 2010	expansion
Desipramine	Eszopiclone	Yes-Additive side-effects	Sandoz 2010	expansion
Desipramine	Glycopyrronium	Yes-Additive side-effects	Sandoz 2010	expansion
Desipramine	Hyoscyamine	Yes-Additive side-effects	Sandoz 2010	expansion
Desipramine	Ipratropium cation	Yes-Additive side-effects	Sandoz 2010	expansion
Desipramine	Ipratropium	Yes-Additive side-effects	Sandoz 2010	expansion
Desipramine	Isocarboxazid	Yes-Hyperpyretic crises, severe convulsions, death	Mylan 2009, Sandoz 2010, Watson 2007 (doxepin), Mylan 2007, Sandoz 2010b, Watson 2009, Duramed 2007, Ciba-Gigy 1996	expansion
Desipramine	Mepenzolate	Yes-Additive side-effects	Sandoz 2010	expansion
Desipramine	Methscopolamine	Yes-Additive side-effects	Sandoz 2010	expansion
Desipramine	Modafinil	Yes-Additive side-effects	Sandoz 2010	expansion
Desipramine	Phenelzine	Yes-Hyperpyretic crises, severe convulsions, death	Mylan 2009, Sandoz 2010, Watson 2007 (doxepin), Mylan 2007, Sandoz 2010b, Watson 2009, Duramed 2007, Ciba-Gigy 1996	expansion
Desipramine	Phenobarbital	Yes-Additive side-effects	Sandoz 2010	expansion
Desipramine	Phenylpropanolamine	Yes-Additive side-effects	Sandoz 2010	expansion
Desipramine	Procyclidine	Yes-Additive side-effects	Sandoz 2010	expansion
Desipramine	Propantheline	Yes-Additive side-effects	Sandoz 2010	expansion
Desipramine	Pseudoephedrine	Yes-Additive side-effects	Sandoz 2010	expansion
Desipramine	Rasagiline	Yes-Hyperpyretic crises, severe convulsions, death	Mylan 2009, Sandoz 2010, Watson 2007 (doxepin), Mylan 2007, Sandoz 2010b, Watson 2009, Duramed 2007, Ciba-Gigy 1996	expansion
Desipramine	Scopolamine	Yes-Additive side-effects	Sandoz 2010	expansion
Desipramine	Sedative Hypnotics	Yes-Additive side-effects	Sandoz 2010	original
Desipramine	Selegiline	Yes-Hyperpyretic crises, severe convulsions, death	Mylan 2009, Sandoz 2010, Watson 2007 (doxepin), Mylan 2007, Sandoz 2010b, Watson 2009, Duramed 2007, Ciba-Gigy 1996	expansion
Desipramine	Sympathomimetic	Yes-Additive side-effects	Sandoz 2010	original
Desipramine	Tiotropium	Yes-Additive side-effects	Sandoz 2010	expansion
Desipramine	Tranquilizer	Yes-Additive side-effects	Sandoz 2010	original

Desipramine	Tranylcypromine	Yes-Hyperpyretic crises, severe convulsions, death	Mylan 2009, Sandoz 2010, Watson 2007 (doxepin), Mylan 2007, Sandoz 2010b, Watson 2009, Duramed 2007, Ciba-Gigy 1996	expansion
Desipramine	Triazolam	Yes-Additive side-effects	Sandoz 2010	expansion
Desipramine	Tropicamide	Yes-Additive side-effects	Sandoz 2010	expansion
Desipramine	Tryptophan	Yes-Additive side-effects	Sandoz 2010	expansion
Desipramine	Zaleplon	Yes-Additive side-effects	Sandoz 2010	expansion
Desvenlafaxine	Acetaminophen	Yes-GI bleeding	Eli-Lilly 2010, Rebel 2010b, Wyeth 2011	expansion
Desvenlafaxine	Amitriptyline	Yes-serotonin syndrome	Eli-Lilly 2010, Rebel 2010b, Wyeth 2011	expansion
Desvenlafaxine	Amoxapine	Yes-serotonin syndrome	Eli-Lilly 2010, Rebel 2010b, Wyeth 2011	expansion
Desvenlafaxine	Antithrombin alfa	Yes-GI bleeding	Eli-Lilly 2010, Rebel 2010b, Wyeth 2011	expansion
Desvenlafaxine	Argatroban	Yes-GI bleeding	Eli-Lilly 2010, Rebel 2010b, Wyeth 2011	expansion
Desvenlafaxine	Aspirin	Yes-GI bleeding	Eli-Lilly 2010, Rebel 2010b, Wyeth 2011	expansion
Desvenlafaxine	Azole Antifungal	Yes	Cardinal Health, 2011	expansion
Desvenlafaxine	Bivalirudin	Yes-GI bleeding	Eli-Lilly 2010, Rebel 2010b, Wyeth 2011	expansion
Desvenlafaxine	Bromfenac	Yes-GI bleeding	Eli-Lilly 2010, Rebel 2010b, Wyeth 2011	expansion
Desvenlafaxine	Buspirone	Yes-serotonin syndrome	Eli-Lilly 2010, Rebel 2010b, Wyeth 2011	expansion
Desvenlafaxine	Celecoxib	Yes-GI bleeding	Eli-Lilly 2010, Rebel 2010b, Wyeth 2011	expansion
Desvenlafaxine	Citalopram	Yes-serotonin syndrome	Eli-Lilly 2010, Rebel 2010b, Wyeth 2011	expansion
Desvenlafaxine	Clomipramine	Yes-serotonin syndrome	Eli-Lilly 2010, Rebel 2010b, Wyeth 2011	expansion
Desvenlafaxine	Desipramine	Yes-serotonin syndrome	Eli-Lilly 2010, Rebel 2010b, Wyeth 2011	expansion
Desvenlafaxine	Desirudin	Yes-GI bleeding	Eli-Lilly 2010, Rebel 2010b, Wyeth 2011	expansion

Desvenlafaxine	Desvenlafaxine	Yes-serotonin syndrome	Eli-Lilly 2010, Rebel 2010b, Wyeth 2011	expansion
Desvenlafaxine	Dextromethorphan	Yes-serotonin syndrome	Eli-Lilly 2010, Rebel 2010b, Wyeth 2011	expansion
Desvenlafaxine	Diclofenac	Yes-GI bleeding	Eli-Lilly 2010, Rebel 2010b, Wyeth 2011	expansion
Desvenlafaxine	Diflunisal	Yes-GI bleeding	Eli-Lilly 2010, Rebel 2010b, Wyeth 2011	expansion
Desvenlafaxine	Doxepin	Yes-serotonin syndrome	Eli-Lilly 2010, Rebel 2010b, Wyeth 2011	expansion
Desvenlafaxine	Duloxetine	Yes-serotonin syndrome	Eli-Lilly 2010, Rebel 2010b, Wyeth 2011	expansion
Desvenlafaxine	Escitalopram	Yes-serotonin syndrome	Eli-Lilly 2010, Rebel 2010b, Wyeth 2011	expansion
Desvenlafaxine	Etodolac	Yes-GI bleeding	Eli-Lilly 2010, Rebel 2010b, Wyeth 2011	expansion
Desvenlafaxine	Fenoprofen	Yes-GI bleeding	Eli-Lilly 2010, Rebel 2010b, Wyeth 2011	expansion
Desvenlafaxine	Fentanyl	Yes-serotonin syndrome	Eli-Lilly 2010, Rebel 2010b, Wyeth 2011	expansion
Desvenlafaxine	Fluoxetine	Yes-serotonin syndrome	Eli-Lilly 2010, Rebel 2010b, Wyeth 2011	expansion
Desvenlafaxine	Flurbiprofen	Yes-GI bleeding	Eli-Lilly 2010, Rebel 2010b, Wyeth 2011	expansion
Desvenlafaxine	Fluvoxamine	Yes-serotonin syndrome	Eli-Lilly 2010, Rebel 2010b, Wyeth 2011	expansion
Desvenlafaxine	Heparin	Yes-GI bleeding	Eli-Lilly 2010, Rebel 2010b, Wyeth 2011	expansion
Desvenlafaxine	Ibuprofen	Yes-GI bleeding	Eli-Lilly 2010, Rebel 2010b, Wyeth 2011	expansion
Desvenlafaxine	Imipramine	Yes-serotonin syndrome	Eli-Lilly 2010, Rebel 2010b, Wyeth 2011	expansion
Desvenlafaxine	Indomethacin	Yes-GI bleeding	Eli-Lilly 2010, Rebel 2010b, Wyeth 2011	expansion
Desvenlafaxine	Isocarboxazid	Yes-serotonin syndrome	Eli-Lilly 2010, Rebel 2010b, Wyeth 2011	expansion
Desvenlafaxine	Ketoconazole	Yes	Cardinal Health, 2011	original
Desvenlafaxine	Ketoprofen	Yes-GI bleeding	Eli-Lilly 2010, Rebel 2010b, Wyeth 2011	expansion

Desvenlafaxine	Ketorolac tromethamine	Yes-GI bleeding	Eli-Lilly 2010, Rebel 2010b, Wyeth 2011	expansion
Desvenlafaxine	Ketorolac	Yes-GI bleeding	Eli-Lilly 2010, Rebel 2010b, Wyeth 2011	expansion
Desvenlafaxine	Lepirudin	Yes-GI bleeding	Eli-Lilly 2010, Rebel 2010b, Wyeth 2011	expansion
Desvenlafaxine	Linezolid	Yes-serotonin syndrome	Eli-Lilly 2010, Rebel 2010b, Wyeth 2011	expansion
Desvenlafaxine	Mannitol	Yes-GI bleeding	Eli-Lilly 2010, Rebel 2010b, Wyeth 2011	expansion
Desvenlafaxine	Maprotiline	Yes-serotonin syndrome	Eli-Lilly 2010, Rebel 2010b, Wyeth 2011	expansion
Desvenlafaxine	Mefenamic acid	Yes-GI bleeding	Eli-Lilly 2010, Rebel 2010b, Wyeth 2011	expansion
Desvenlafaxine	Meloxicam	Yes-GI bleeding	Eli-Lilly 2010, Rebel 2010b, Wyeth 2011	expansion
Desvenlafaxine	Meperidine	Yes-serotonin syndrome	Eli-Lilly 2010, Rebel 2010b, Wyeth 2011	expansion
Desvenlafaxine	Milnacipran	Yes-serotonin syndrome	Eli-Lilly 2010, Rebel 2010b, Wyeth 2011	expansion
Desvenlafaxine	NSAID	Yes-GI bleeding	Eli-Lilly 2010, Rebel 2010b, Wyeth 2011	expansion
Desvenlafaxine	Nabumetone	Yes-GI bleeding	Eli-Lilly 2010, Rebel 2010b, Wyeth 2011	expansion
Desvenlafaxine	Naproxen	Yes-GI bleeding	Eli-Lilly 2010, Rebel 2010b, Wyeth 2011	expansion
Desvenlafaxine	Nefazodone	Yes-serotonin syndrome	Eli-Lilly 2010, Rebel 2010b, Wyeth 2011	expansion
Desvenlafaxine	Nepafenac	Yes-GI bleeding	Eli-Lilly 2010, Rebel 2010b, Wyeth 2011	expansion
Desvenlafaxine	Nortriptyline	Yes-serotonin syndrome	Eli-Lilly 2010, Rebel 2010b, Wyeth 2011	expansion
Desvenlafaxine	Odansetran	Yes-serotonin syndrome	Eli-Lilly 2010, Rebel 2010b, Wyeth 2011	expansion
Desvenlafaxine	Oxaprozin	Yes-GI bleeding	Eli-Lilly 2010, Rebel 2010b, Wyeth 2011	expansion
Desvenlafaxine	Paroxetine	Yes-serotonin syndrome	Eli-Lilly 2010, Rebel 2010b, Wyeth 2011	expansion

Desvenlafaxine	Pentazocine	Yes-serotonin syndrome	Eli-Lilly 2010, Rebel 2010b, Wyeth 2011	expansion
Desvenlafaxine	Phenelzine	Yes-serotonin syndrome	Eli-Lilly 2010, Rebel 2010b, Wyeth 2011	expansion
Desvenlafaxine	Piroxicam	Yes-GI bleeding	Eli-Lilly 2010, Rebel 2010b, Wyeth 2011	expansion
Desvenlafaxine	Protriptyline	Yes-serotonin syndrome	Eli-Lilly 2010, Rebel 2010b, Wyeth 2011	expansion
Desvenlafaxine	Rasagiline	Yes-serotonin syndrome	Eli-Lilly 2010, Rebel 2010b, Wyeth 2011	expansion
Desvenlafaxine	SEROTONIN-1B AND SERC	Yes-serotonin syndrome	Eli-Lilly 2010, Rebel 2010b, Wyeth 2011	expansion
Desvenlafaxine	Salicylamide	Yes-GI bleeding	Eli-Lilly 2010, Rebel 2010b, Wyeth 2011	expansion
Desvenlafaxine	Selegiline	Yes-serotonin syndrome	Eli-Lilly 2010, Rebel 2010b, Wyeth 2011	expansion
Desvenlafaxine	Sertraline	Yes-serotonin syndrome	Eli-Lilly 2010, Rebel 2010b, Wyeth 2011	expansion
Desvenlafaxine	St Johns wort	Yes-serotonin syndrome	Eli-Lilly 2010, Rebel 2010b, Wyeth 2011	expansion
Desvenlafaxine	Sulindac	Yes-GI bleeding	Eli-Lilly 2010, Rebel 2010b, Wyeth 2011	expansion
Desvenlafaxine	Sumatriptan	Yes-serotonin syndrome	Eli-Lilly 2010, Rebel 2010b, Wyeth 2011	expansion
Desvenlafaxine	Sympathomimetic	Yes-serotonin syndrome	Eli-Lilly 2010, Rebel 2010b, Wyeth 2011	expansion
Desvenlafaxine	Tolmetin	Yes-GI bleeding	Eli-Lilly 2010, Rebel 2010b, Wyeth 2011	expansion
Desvenlafaxine	Tramadol	Yes-serotonin syndrome	Eli-Lilly 2010, Rebel 2010b, Wyeth 2011	expansion
Desvenlafaxine	Tranylcypromine	Yes-serotonin syndrome	Eli-Lilly 2010, Rebel 2010b, Wyeth 2011	expansion
Desvenlafaxine	Trazodone	Yes-serotonin syndrome	Eli-Lilly 2010, Rebel 2010b, Wyeth 2011	expansion
Desvenlafaxine	Trimipramine	Yes-serotonin syndrome	Eli-Lilly 2010, Rebel 2010b, Wyeth 2011	expansion
Desvenlafaxine	Tryptophan	Yes-serotonin syndrome	Eli-Lilly 2010, Rebel 2010b, Wyeth 2011	expansion

Desvenlafaxine	VITAMIN K ANTAGONIST	Yes-GI bleeding	Eli-Lilly 2010, Rebel 2010b, Wyeth 2011	expansion
Desvenlafaxine	Venlafaxine	Yes-serotonin syndrome	Eli-Lilly 2010, Rebel 2010b, Wyeth 2011	expansion
Desvenlafaxine	Vilazodone	Yes-serotonin syndrome	Eli-Lilly 2010, Rebel 2010b, Wyeth 2011	expansion
Desvenlafaxine	Warfarin	Yes-GI bleeding	Eli-Lilly 2010, Rebel 2010b, Wyeth 2011	expansion
Doxepin	Cimetidine	Yes?- anticholinergic symptoms (i.e., severe dry mo	Watson 2007	original
Doxepin	H2 blockers	Yes?- anticholinergic symptoms (i.e., severe dry mo	Watson 2007	expansion
Doxepin	Isocarboxazid	Yes-Hyperpyretic crises, severe convulsions, death	Mylan 2009, Sandoz 2010, Watson 2007 (doxepin), Mylan 2007, Sandoz 2010b, Watson 2009, Duramed 2007, Ciba-Gigy 1996	expansion
Doxepin	Phenelzine	Yes-Hyperpyretic crises, severe convulsions, death	Mylan 2009, Sandoz 2010, Watson 2007 (doxepin), Mylan 2007, Sandoz 2010b, Watson 2009, Duramed 2007, Ciba-Gigy 1996	expansion
Doxepin	Rasagiline	Yes-Hyperpyretic crises, severe convulsions, death	Mylan 2009, Sandoz 2010, Watson 2007 (doxepin), Mylan 2007, Sandoz 2010b, Watson 2009, Duramed 2007, Ciba-Gigy 1996	expansion
Doxepin	Selegiline	Yes-Hyperpyretic crises, severe convulsions, death	Mylan 2009, Sandoz 2010, Watson 2007 (doxepin), Mylan 2007, Sandoz 2010b, Watson 2009, Duramed 2007, Ciba-Gigy 1996	expansion
Doxepin	Sulfonylurea	Yes-hypoglycemia	Watson 2007	expansion
Doxepin	Tolazamide	Yes-hypoglycemia	Watson 2007	original

Doxepin	Tranylcypromine	Yes-Hyperpyretic crises, severe convulsions, death	Mylan 2009, Sandoz 2010, Watson 2007 (doxepin), Mylan 2007, Sandoz 2010b, Watson 2009, Duramed 2007, Ciba-Gigy 1996	expansion
Duloxetine	Acetaminophen	Yes-GI bleeding	Eli-Lilly 2010, Rebel 2010b, Wyeth 2011	expansion
Duloxetine	Amitriptyline	Yes-serotonin syndrome	Eli-Lilly 2010, Rebel 2010b, Wyeth 2011	expansion
Duloxetine	Amoxapine	Yes-serotonin syndrome	Eli-Lilly 2010, Rebel 2010b, Wyeth 2011	expansion
Duloxetine	Antithrombin alfa	Yes-GI bleeding	Eli-Lilly 2010, Rebel 2010b, Wyeth 2011	expansion
Duloxetine	Argatroban	Yes-GI bleeding	Eli-Lilly 2010, Rebel 2010b, Wyeth 2011	expansion
Duloxetine	Aspirin	Yes-GI bleeding	Eli-Lilly 2010, Rebel 2010b, Wyeth 2011	expansion
Duloxetine	Benzodiazepine	No	Eli-Lilly 2010	expansion
Duloxetine	Bivalirudin	Yes-GI bleeding	Eli-Lilly 2010, Rebel 2010b, Wyeth 2011	expansion
Duloxetine	Bromfenac	Yes-GI bleeding	Eli-Lilly 2010, Rebel 2010b, Wyeth 2011	expansion
Duloxetine	Buspirone	Yes-serotonin syndrome	Eli-Lilly 2010, Rebel 2010b, Wyeth 2011	expansion
Duloxetine	Celecoxib	Yes-GI bleeding	Eli-Lilly 2010, Rebel 2010b, Wyeth 2011	expansion
Duloxetine	Citalopram	Yes-serotonin syndrome	Eli-Lilly 2010, Rebel 2010b, Wyeth 2011	expansion
Duloxetine	Clomipramine	Yes-serotonin syndrome	Eli-Lilly 2010, Rebel 2010b, Wyeth 2011	expansion
Duloxetine	Desipramine	Yes-serotonin syndrome	Eli-Lilly 2010, Rebel 2010b, Wyeth 2011	expansion
Duloxetine	Desirudin	Yes-GI bleeding	Eli-Lilly 2010, Rebel 2010b, Wyeth 2011	expansion
Duloxetine	Desvenlafaxine	Yes-serotonin syndrome	Eli-Lilly 2010, Rebel 2010b, Wyeth 2011	expansion
Duloxetine	Dextromethorphan	Yes-serotonin syndrome	Eli-Lilly 2010, Rebel 2010b, Wyeth 2011	expansion

Duloxetine	Diclofenac	Yes-GI bleeding	Eli-Lilly 2010, Rebel 2010b, Wyeth 2011	expansion
Duloxetine	Diflunisal	Yes-GI bleeding	Eli-Lilly 2010, Rebel 2010b, Wyeth 2011	expansion
Duloxetine	Doxepin	Yes-serotonin syndrome	Eli-Lilly 2010, Rebel 2010b, Wyeth 2011	expansion
Duloxetine	Duloxetine	Yes-serotonin syndrome	Eli-Lilly 2010, Rebel 2010b, Wyeth 2011	expansion
Duloxetine	Escitalopram	Yes-serotonin syndrome	Eli-Lilly 2010, Rebel 2010b, Wyeth 2011	expansion
Duloxetine	Etodolac	Yes-GI bleeding	Eli-Lilly 2010, Rebel 2010b, Wyeth 2011	expansion
Duloxetine	Fenoprofen	Yes-GI bleeding	Eli-Lilly 2010, Rebel 2010b, Wyeth 2011	expansion
Duloxetine	Fentanyl	Yes-serotonin syndrome	Eli-Lilly 2010, Rebel 2010b, Wyeth 2011	expansion
Duloxetine	Fluoxetine	Yes-serotonin syndrome	Eli-Lilly 2010, Rebel 2010b, Wyeth 2011	expansion
Duloxetine	Flurbiprofen	Yes-GI bleeding	Eli-Lilly 2010, Rebel 2010b, Wyeth 2011	expansion
Duloxetine	Fluvoxamine	Yes	Eli-Lilly 2010	original
Duloxetine	Fluvoxamine	Yes-serotonin syndrome	Eli-Lilly 2010, Rebel 2010b, Wyeth 2011	expansion
Duloxetine	Heparin	Yes-GI bleeding	Eli-Lilly 2010, Rebel 2010b, Wyeth 2011	expansion
Duloxetine	Ibuprofen	Yes-GI bleeding	Eli-Lilly 2010, Rebel 2010b, Wyeth 2011	expansion
Duloxetine	Imipramine	Yes-serotonin syndrome	Eli-Lilly 2010, Rebel 2010b, Wyeth 2011	expansion
Duloxetine	Indomethacin	Yes-GI bleeding	Eli-Lilly 2010, Rebel 2010b, Wyeth 2011	expansion
Duloxetine	Isocarboxazid	Yes-serotonin syndrome	Eli-Lilly 2010, Rebel 2010b, Wyeth 2011	expansion
Duloxetine	Ketoprofen	Yes-GI bleeding	Eli-Lilly 2010, Rebel 2010b, Wyeth 2011	expansion
Duloxetine	Ketorolac tromethamine	Yes-GI bleeding	Eli-Lilly 2010, Rebel 2010b, Wyeth 2011	expansion
Duloxetine	Ketorolac	Yes-GI bleeding	Eli-Lilly 2010, Rebel 2010b, Wyeth 2011	expansion

Duloxetine	Lepirudin	Yes-GI bleeding	Eli-Lilly 2010, Rebel 2010b, Wyeth 2011	expansion
Duloxetine	Linezolid	Yes-serotonin syndrome	Eli-Lilly 2010, Rebel 2010b, Wyeth 2011	expansion
Duloxetine	Lorazepam	No	Eli-Lilly 2010	original
Duloxetine	Mannitol	Yes-GI bleeding	Eli-Lilly 2010, Rebel 2010b, Wyeth 2011	expansion
Duloxetine	Maprotiline	Yes-serotonin syndrome	Eli-Lilly 2010, Rebel 2010b, Wyeth 2011	expansion
Duloxetine	Mefenamic acid	Yes-GI bleeding	Eli-Lilly 2010, Rebel 2010b, Wyeth 2011	expansion
Duloxetine	Meloxicam	Yes-GI bleeding	Eli-Lilly 2010, Rebel 2010b, Wyeth 2011	expansion
Duloxetine	Meperidine	Yes-serotonin syndrome	Eli-Lilly 2010, Rebel 2010b, Wyeth 2011	expansion
Duloxetine	Milnacipran	Yes-serotonin syndrome	Eli-Lilly 2010, Rebel 2010b, Wyeth 2011	expansion
Duloxetine	NSAID	Yes-GI bleeding	Eli-Lilly 2010, Rebel 2010b, Wyeth 2011	expansion
Duloxetine	Nabumetone	Yes-GI bleeding	Eli-Lilly 2010, Rebel 2010b, Wyeth 2011	expansion
Duloxetine	Naproxen	Yes-GI bleeding	Eli-Lilly 2010, Rebel 2010b, Wyeth 2011	expansion
Duloxetine	Nefazodone	Yes-serotonin syndrome	Eli-Lilly 2010, Rebel 2010b, Wyeth 2011	expansion
Duloxetine	Nepafenac	Yes-GI bleeding	Eli-Lilly 2010, Rebel 2010b, Wyeth 2011	expansion
Duloxetine	Nortriptyline	Yes-serotonin syndrome	Eli-Lilly 2010, Rebel 2010b, Wyeth 2011	expansion
Duloxetine	Odansetran	Yes-serotonin syndrome	Eli-Lilly 2010, Rebel 2010b, Wyeth 2011	expansion
Duloxetine	Oxaprozin	Yes-GI bleeding	Eli-Lilly 2010, Rebel 2010b, Wyeth 2011	expansion
Duloxetine	Paroxetine	Yes	Eli-Lilly 2010	original
Duloxetine	Paroxetine	Yes-serotonin syndrome	Eli-Lilly 2010, Rebel 2010b, Wyeth 2011	expansion
Duloxetine	Pentazocine	Yes-serotonin syndrome	Eli-Lilly 2010, Rebel 2010b, Wyeth 2011	expansion
Duloxetine	Phenelzine	Yes-serotonin syndrome	Eli-Lilly 2010, Rebel 2010b, Wyeth 2011	expansion

Duloxetine	Piroxicam	Yes-GI bleeding	Eli-Lilly 2010, Rebel 2010b, Wyeth 2011	expansion
Duloxetine	Protriptyline	Yes-serotonin syndrome	Eli-Lilly 2010, Rebel 2010b, Wyeth 2011	expansion
Duloxetine	Rasagiline	Yes-serotonin syndrome	Eli-Lilly 2010, Rebel 2010b, Wyeth 2011	expansion
Duloxetine	SEROTONIN-1B AND SERC	Yes-serotonin syndrome	Eli-Lilly 2010, Rebel 2010b, Wyeth 2011	expansion
Duloxetine	SSRI	Yes	Eli-Lilly 2010	expansion
Duloxetine	Salicylamide	Yes-GI bleeding	Eli-Lilly 2010, Rebel 2010b, Wyeth 2011	expansion
Duloxetine	Selegiline	Yes-serotonin syndrome	Eli-Lilly 2010, Rebel 2010b, Wyeth 2011	expansion
Duloxetine	Sertraline	Yes-serotonin syndrome	Eli-Lilly 2010, Rebel 2010b, Wyeth 2011	expansion
Duloxetine	St Johns wort	Yes-serotonin syndrome	Eli-Lilly 2010, Rebel 2010b, Wyeth 2011	expansion
Duloxetine	Sulindac	Yes-GI bleeding	Eli-Lilly 2010, Rebel 2010b, Wyeth 2011	expansion
Duloxetine	Sumatriptan	Yes-serotonin syndrome	Eli-Lilly 2010, Rebel 2010b, Wyeth 2011	expansion
Duloxetine	Sympathomimetic	Yes-serotonin syndrome	Eli-Lilly 2010, Rebel 2010b, Wyeth 2011	expansion
Duloxetine	Temazepam	No	Eli-Lilly 2010	original
Duloxetine	Tolmetin	Yes-GI bleeding	Eli-Lilly 2010, Rebel 2010b, Wyeth 2011	expansion
Duloxetine	Tramadol	Yes-serotonin syndrome	Eli-Lilly 2010, Rebel 2010b, Wyeth 2011	expansion
Duloxetine	Tranylcypromine	Yes-serotonin syndrome	Eli-Lilly 2010, Rebel 2010b, Wyeth 2011	expansion
Duloxetine	Trazodone	Yes-serotonin syndrome	Eli-Lilly 2010, Rebel 2010b, Wyeth 2011	expansion
Duloxetine	Trimipramine	Yes-serotonin syndrome	Eli-Lilly 2010, Rebel 2010b, Wyeth 2011	expansion
Duloxetine	Tryptophan	Yes-serotonin syndrome	Eli-Lilly 2010, Rebel 2010b, Wyeth 2011	expansion
Duloxetine	VITAMIN K ANTAGONIST	Yes-GI bleeding	Eli-Lilly 2010, Rebel 2010b, Wyeth 2011	expansion
Duloxetine	Venlafaxine	Yes-serotonin syndrome	Eli-Lilly 2010, Rebel 2010b, Wyeth 2011	expansion

Duloxetine	Vilazodone	Yes-serotonin syndrome	Eli-Lilly 2010, Rebel 2010b, Wyeth 2011	expansion
Duloxetine	Warfarin	Yes-GI bleeding	Eli-Lilly 2010, Rebel 2010b, Wyeth 2011	expansion
Escitalopram	Acetaminophen	Yes-bleeding	Forest 2009, Forest 2011, Dista 2009, Alphapharm 2007, Watson 2008, Forest 2011B	expansion
Escitalopram	Aminoketone	Yes-bleeding	Forest 2009, Forest 2011, Dista 2009, Alphapharm 2007, Watson 2008, Forest 2011B	expansion
Escitalopram	Amitriptyline	Yes-serotonin syndrome	Forest 2009, Forest 2011, Dista 2009, Alphapharm 2007, Watson 2008, Forest 2011B (Vilazodone)	expansion
Escitalopram	Amoxapine	Yes-serotonin syndrome	Forest 2009, Forest 2011, Dista 2009, Alphapharm 2007, Watson 2008, Forest 2011B (Vilazodone)	expansion
Escitalopram	Antipsychotic	No	Forest 2009, Forest 2011	expansion
Escitalopram	Antipsychotic	Yes-QTc interval prolongation	Forest 2009, Forest 2011, Dista 2009, Alphapharm 2007, Watson 2008	expansion
Escitalopram	Antithrombin alfa	Yes-bleeding	Forest 2009, Forest 2011, Dista 2009, Alphapharm 2007, Watson 2008, Forest 2011B	expansion
Escitalopram	Argatroban	Yes-bleeding	Forest 2009, Forest 2011, Dista 2009, Alphapharm 2007, Watson 2008, Forest 2011B	expansion

Escitalopram	Aspirin	Yes-bleeding	Forest 2009, Forest 2011, Dista 2009, Alphapharm 2007, Watson 2008, Forest 2011B	expansion
Escitalopram	Azole Antifungal	No	Forest 2009, Forest 2011	expansion
Escitalopram	Bivalirudin	Yes-bleeding	Forest 2009, Forest 2011, Dista 2009, Alphapharm 2007, Watson 2008, Forest 2011B	expansion
Escitalopram	Bromfenac	Yes-bleeding	Forest 2009, Forest 2011, Dista 2009, Alphapharm 2007, Watson 2008, Forest 2011B	expansion
Escitalopram	Bupropion	Yes-bleeding	Forest 2009, Forest 2011, Dista 2009, Alphapharm 2007, Watson 2008, Forest 2011B	expansion
Escitalopram	Buspirone	Yes-serotonin syndrome	Forest 2009, Forest 2011, Dista 2009, Alphapharm 2007, Watson 2008, Forest 2011B (Vilazodone)	expansion
Escitalopram	Cardiac Glycoside	No	Forest 2009, Forest 2011	expansion
Escitalopram	Celecoxib	Yes-bleeding	Forest 2009, Forest 2011, Dista 2009, Alphapharm 2007, Watson 2008, Forest 2011B	expansion
Escitalopram	Cimetidine	Yes	Forest 2009, Forest 2011	original
Escitalopram	Citalopram	Yes-serotonin syndrome	Forest 2009, Forest 2011, Dista 2009, Alphapharm 2007, Watson 2008, Forest 2011B (Vilazodone)	expansion

Escitalopram	Clomipramine	Yes-serotonin syndrome	Forest 2009, Forest 2011, Dista 2009, Alphapharm 2007, Watson 2008, Forest 2011B (Vilazodone)	expansion
Escitalopram	Desipramine	Yes-serotonin syndrome	Forest 2009, Forest 2011, Dista 2009, Alphapharm 2007, Watson 2008, Forest 2011B (Vilazodone)	expansion
Escitalopram	Desirudin	Yes-bleeding	Forest 2009, Forest 2011, Dista 2009, Alphapharm 2007, Watson 2008, Forest 2011B	expansion
Escitalopram	Desvenlafaxine	Yes-serotonin syndrome	Forest 2009, Forest 2011, Dista 2009, Alphapharm 2007, Watson 2008, Forest 2011B (Vilazodone)	expansion
Escitalopram	Dextromethorphan	Yes-serotonin syndrome	Forest 2009, Forest 2011, Dista 2009, Alphapharm 2007, Watson 2008, Forest 2011B (Vilazodone)	expansion
Escitalopram	Diclofenac	Yes-bleeding	Forest 2009, Forest 2011, Dista 2009, Alphapharm 2007, Watson 2008, Forest 2011B	expansion
Escitalopram	Diflunisal	Yes-bleeding	Forest 2009, Forest 2011, Dista 2009, Alphapharm 2007, Watson 2008, Forest 2011B	expansion
Escitalopram	Digoxin	No	Forest 2009, Forest 2011	original

Escitalopram	Doxepin	Yes-serotonin syndrome	Forest 2009, Forest 2011, Dista 2009, Alphapharm 2007, Watson 2008, Forest 2011B (Vilazodone)	expansion
Escitalopram	Duloxetine	Yes-serotonin syndrome	Forest 2009, Forest 2011, Dista 2009, Alphapharm 2007, Watson 2008, Forest 2011B (Vilazodone)	expansion
Escitalopram	Escitalopram	Yes-serotonin syndrome	Forest 2009, Forest 2011, Dista 2009, Alphapharm 2007, Watson 2008, Forest 2011B (Vilazodone)	expansion
Escitalopram	Etodolac	Yes-bleeding	Forest 2009, Forest 2011, Dista 2009, Alphapharm 2007, Watson 2008, Forest 2011B	expansion
Escitalopram	Fenoprofen	Yes-bleeding	Forest 2009, Forest 2011, Dista 2009, Alphapharm 2007, Watson 2008, Forest 2011B	expansion
Escitalopram	Fentanyl	Yes-serotonin syndrome	Forest 2009, Forest 2011, Dista 2009, Alphapharm 2007, Watson 2008, Forest 2011B (Vilazodone)	expansion
Escitalopram	Fluoxetine	Yes-serotonin syndrome	Forest 2009, Forest 2011, Dista 2009, Alphapharm 2007, Watson 2008, Forest 2011B (Vilazodone)	expansion

Escitalopram	Flurbiprofen	Yes-bleeding	Forest 2009, Forest 2011, Dista 2009, Alphapharm 2007, Watson 2008, Forest 2011B	expansion
Escitalopram	Fluvoxamine	Yes-serotonin syndrome	Forest 2009, Forest 2011, Dista 2009, Alphapharm 2007, Watson 2008, Forest 2011B (Vilazodone)	expansion
Escitalopram	H2 blockers	Yes	Forest 2009, Forest 2011	expansion
Escitalopram	Heparin	Yes-bleeding	Forest 2009, Forest 2011, Dista 2009, Alphapharm 2007, Watson 2008, Forest 2011B	expansion
Escitalopram	Ibuprofen	Yes-bleeding	Forest 2009, Forest 2011, Dista 2009, Alphapharm 2007, Watson 2008, Forest 2011B	expansion
Escitalopram	Imipramine	Yes-serotonin syndrome	Forest 2009, Forest 2011, Dista 2009, Alphapharm 2007, Watson 2008, Forest 2011B (Vilazodone)	expansion
Escitalopram	Indomethacin	Yes-bleeding	Forest 2009, Forest 2011, Dista 2009, Alphapharm 2007, Watson 2008, Forest 2011B	expansion
Escitalopram	Isocarboxazid	Yes-serotonin syndrome	Forest 2009, Forest 2011, Dista 2009, Alphapharm 2007, Watson 2008, Forest 2011B (Vilazodone)	expansion
Escitalopram	Ketoconazole	No	Forest 2009, Forest 2011	original

Escitalopram	Ketoprofen	Yes-bleeding	Forest 2009, Forest 2011, Dista 2009, Alphapharm 2007, Watson 2008, Forest 2011B	expansion
Escitalopram	Ketorolac tromethamine	Yes-bleeding	Forest 2009, Forest 2011, Dista 2009, Alphapharm 2007, Watson 2008, Forest 2011B	expansion
Escitalopram	Ketorolac	Yes-bleeding	Forest 2009, Forest 2011, Dista 2009, Alphapharm 2007, Watson 2008, Forest 2011B	expansion
Escitalopram	Lepirudin	Yes-bleeding	Forest 2009, Forest 2011, Dista 2009, Alphapharm 2007, Watson 2008, Forest 2011B	expansion
Escitalopram	Linezolid	Yes-serotonin syndrome	Forest 2009, Forest 2011, Dista 2009, Alphapharm 2007, Watson 2008, Forest 2011B (Vilazodone)	expansion
Escitalopram	Lithium	No	Forest 2009, Forest 2011	original
Escitalopram	Mannitol	Yes-bleeding	Forest 2009, Forest 2011, Dista 2009, Alphapharm 2007, Watson 2008, Forest 2011B	expansion
Escitalopram	Maprotiline	Yes-serotonin syndrome	Forest 2009, Forest 2011, Dista 2009, Alphapharm 2007, Watson 2008, Forest 2011B (Vilazodone)	expansion

Escitalopram	Mefenamic acid	Yes-bleeding	Forest 2009, Forest 2011, Dista 2009, Alphapharm 2007, Watson 2008, Forest 2011B	expansion
Escitalopram	Meloxicam	Yes-bleeding	Forest 2009, Forest 2011, Dista 2009, Alphapharm 2007, Watson 2008, Forest 2011B	expansion
Escitalopram	Meperidine	Yes-serotonin syndrome	Forest 2009, Forest 2011, Dista 2009, Alphapharm 2007, Watson 2008, Forest 2011B (Vilazodone)	expansion
Escitalopram	Milnacipran	Yes-serotonin syndrome	Forest 2009, Forest 2011, Dista 2009, Alphapharm 2007, Watson 2008, Forest 2011B (Vilazodone)	expansion
Escitalopram	NSAID	Yes-bleeding	Forest 2009, Forest 2011, Dista 2009, Alphapharm 2007, Watson 2008, Forest 2011B	expansion
Escitalopram	Nabumetone	Yes-bleeding	Forest 2009, Forest 2011, Dista 2009, Alphapharm 2007, Watson 2008, Forest 2011B	expansion
Escitalopram	Naproxen	Yes-bleeding	Forest 2009, Forest 2011, Dista 2009, Alphapharm 2007, Watson 2008, Forest 2011B	expansion
Escitalopram	Nefazodone	Yes-serotonin syndrome	Forest 2009, Forest 2011, Dista 2009, Alphapharm 2007, Watson 2008, Forest 2011B (Vilazodone)	expansion

Escitalopram	Nepafenac	Yes-bleeding	Forest 2009, Forest 2011, Dista 2009, Alphapharm 2007, Watson 2008, Forest 2011B	expansion
Escitalopram	Nortriptyline	Yes-serotonin syndrome	Forest 2009, Forest 2011, Dista 2009, Alphapharm 2007, Watson 2008, Forest 2011B (Vilazodone)	expansion
Escitalopram	Odansetran	Yes-serotonin syndrome	Forest 2009, Forest 2011, Dista 2009, Alphapharm 2007, Watson 2008, Forest 2011B (Vilazodone)	expansion
Escitalopram	Oxaprozin	Yes-bleeding	Forest 2009, Forest 2011, Dista 2009, Alphapharm 2007, Watson 2008, Forest 2011B	expansion
Escitalopram	Paroxetine	Yes-serotonin syndrome	Forest 2009, Forest 2011, Dista 2009, Alphapharm 2007, Watson 2008, Forest 2011B (Vilazodone)	expansion
Escitalopram	Pentazocine	Yes-serotonin syndrome	Forest 2009, Forest 2011, Dista 2009, Alphapharm 2007, Watson 2008, Forest 2011B (Vilazodone)	expansion
Escitalopram	Phenelzine	Yes-serotonin syndrome	Forest 2009, Forest 2011, Dista 2009, Alphapharm 2007, Watson 2008, Forest 2011B (Vilazodone)	expansion

Escitalopram	Pimozide	Yes-QTc interval prolongation	Forest 2009, Forest 2011, Dista 2009, Alphapharm 2007, Watson 2008	expansion
Escitalopram	Piroxicam	Yes-bleeding	Forest 2009, Forest 2011, Dista 2009, Alphapharm 2007, Watson 2008, Forest 2011B	expansion
Escitalopram	Protease Inhibitor	No	Forest 2011	expansion
Escitalopram	Protriptyline	Yes-serotonin syndrome	Forest 2009, Forest 2011, Dista 2009, Alphapharm 2007, Watson 2008, Forest 2011B (Vilazodone)	expansion
Escitalopram	Rasagiline	Yes-serotonin syndrome	Forest 2009, Forest 2011, Dista 2009, Alphapharm 2007, Watson 2008, Forest 2011B (Vilazodone)	expansion
Escitalopram	Ritonavir	No	Forest 2011	original
Escitalopram	SEROTONIN-1B AND SERC	Yes-serotonin syndrome	Forest 2009, Forest 2011, Dista 2009, Alphapharm 2007, Watson 2008, Forest 2011B (Vilazodone)	expansion
Escitalopram	SEROTONIN-1B AND SERC	Yes-weakness, hyperreflexia, and incoordination	Forest 2009, Forest 2011, Watson 2008	expansion
Escitalopram	Salicylamide	Yes-bleeding	Forest 2009, Forest 2011, Dista 2009, Alphapharm 2007, Watson 2008, Forest 2011B	expansion
Escitalopram	Sedative Hypnotics	No	Forest 2009, Forest 2011	expansion
Escitalopram	Selegiline	Yes-serotonin syndrome	Forest 2009, Forest 2011, Dista 2009, Alphapharm 2007, Watson 2008, Forest 2011B (Vilazodone)	expansion

Escitalopram	Sertraline	Yes-serotonin syndrome	Forest 2009, Forest 2011, Dista 2009, Alphapharm 2007, Watson 2008, Forest 2011B (Vilazodone)	expansion
Escitalopram	St Johns wort	Yes-serotonin syndrome	Forest 2009, Forest 2011, Dista 2009, Alphapharm 2007, Watson 2008, Forest 2011B (Vilazodone)	expansion
Escitalopram	Sulindac	Yes-bleeding	Forest 2009, Forest 2011, Dista 2009, Alphapharm 2007, Watson 2008, Forest 2011B	expansion
Escitalopram	Sumatriptan	Yes-serotonin syndrome	Forest 2009, Forest 2011, Dista 2009, Alphapharm 2007, Watson 2008, Forest 2011B (Vilazodone)	expansion
Escitalopram	Sumatriptan	Yes-weakness, hyperreflexia, and incoordination	Forest 2009, Forest 2011, Watson 2008	expansion
Escitalopram	Sympathomimetic	Yes-serotonin syndrome	Forest 2009, Forest 2011, Dista 2009, Alphapharm 2007, Watson 2008, Forest 2011B (Vilazodone)	expansion
Escitalopram	Tolmetin	Yes-bleeding	Forest 2009, Forest 2011, Dista 2009, Alphapharm 2007, Watson 2008, Forest 2011B	expansion
Escitalopram	Tramadol	Yes-serotonin syndrome	Forest 2009, Forest 2011, Dista 2009, Alphapharm 2007, Watson 2008, Forest 2011B (Vilazodone)	expansion

Escitalopram	Tranylcypromine	Yes-serotonin syndrome	Forest 2009, Forest 2011, Dista 2009, Alphapharm 2007, Watson 2008, Forest 2011B (Vilazodone)	expansion
Escitalopram	Trazodone	Yes-serotonin syndrome	Forest 2009, Forest 2011, Dista 2009, Alphapharm 2007, Watson 2008, Forest 2011B (Vilazodone)	expansion
Escitalopram	Triazolam	No	Forest 2009, Forest 2011	original
Escitalopram	Trimipramine	Yes-serotonin syndrome	Forest 2009, Forest 2011, Dista 2009, Alphapharm 2007, Watson 2008, Forest 2011B (Vilazodone)	expansion
Escitalopram	Tryptophan	Yes-serotonin syndrome	Forest 2009, Forest 2011, Dista 2009, Alphapharm 2007, Watson 2008, Forest 2011B (Vilazodone)	expansion
Escitalopram	VITAMIN K ANTAGONIST	Yes-bleeding	Forest 2009, Forest 2011, Dista 2009, Alphapharm 2007, Watson 2008, Forest 2011B	expansion
Escitalopram	Venlafaxine	Yes-serotonin syndrome	Forest 2009, Forest 2011, Dista 2009, Alphapharm 2007, Watson 2008, Forest 2011B (Vilazodone)	expansion
Escitalopram	Vilazodone	Yes-serotonin syndrome	Forest 2009, Forest 2011, Dista 2009, Alphapharm 2007, Watson 2008, Forest 2011B (Vilazodone)	expansion

Escitalopram	Warfarin	Yes-bleeding	Forest 2009, Forest 2011, Dista 2009, Alphapharm 2007, Watson 2008, Forest 2011B	expansion
Fluoxetine	Acetaminophen	Yes-bleeding	Forest 2009, Forest 2011, Dista 2009, Alphapharm 2007, Watson 2008, Forest 2011B	expansion
Fluoxetine	Aminoketone	Yes-bleeding	Forest 2009, Forest 2011, Dista 2009, Alphapharm 2007, Watson 2008, Forest 2011B	expansion
Fluoxetine	Amitriptyline	Yes-serotonin syndrome	Forest 2009, Forest 2011, Dista 2009, Alphapharm 2007, Watson 2008, Forest 2011B (Vilazodone)	expansion
Fluoxetine	Amoxapine	Yes-serotonin syndrome	Forest 2009, Forest 2011, Dista 2009, Alphapharm 2007, Watson 2008, Forest 2011B (Vilazodone)	expansion
Fluoxetine	Antipsychotic	Yes-Lithium toxicity	Dista 2009	expansion
Fluoxetine	Antipsychotic	Yes-QTc interval prolongation	Dista 2009	expansion
Fluoxetine	Antipsychotic	Yes-QTc interval prolongation	Forest 2009, Forest 2011, Dista 2009, Alphapharm 2007, Watson 2008	expansion
Fluoxetine	Antithrombin alfa	Yes-bleeding	Forest 2009, Forest 2011, Dista 2009, Alphapharm 2007, Watson 2008, Forest 2011B	expansion
Fluoxetine	Argatroban	Yes-bleeding	Forest 2009, Forest 2011, Dista 2009, Alphapharm 2007, Watson 2008, Forest 2011B	expansion

Fluoxetine	Aspirin	Yes-bleeding	Forest 2009, Forest 2011, Dista 2009, Alphapharm 2007, Watson 2008, Forest 2011B	expansion
Fluoxetine	Bivalirudin	Yes-bleeding	Forest 2009, Forest 2011, Dista 2009, Alphapharm 2007, Watson 2008, Forest 2011B	expansion
Fluoxetine	Bromfenac	Yes-bleeding	Forest 2009, Forest 2011, Dista 2009, Alphapharm 2007, Watson 2008, Forest 2011B	expansion
Fluoxetine	Bupropion	Yes-bleeding	Forest 2009, Forest 2011, Dista 2009, Alphapharm 2007, Watson 2008, Forest 2011B	expansion
Fluoxetine	Buspirone	Yes-serotonin syndrome	Forest 2009, Forest 2011, Dista 2009, Alphapharm 2007, Watson 2008, Forest 2011B (Vilazodone)	expansion
Fluoxetine	Celecoxib	Yes-bleeding	Forest 2009, Forest 2011, Dista 2009, Alphapharm 2007, Watson 2008, Forest 2011B	expansion
Fluoxetine	Citalopram	Yes-serotonin syndrome	Forest 2009, Forest 2011, Dista 2009, Alphapharm 2007, Watson 2008, Forest 2011B (Vilazodone)	expansion
Fluoxetine	Clomipramine	Yes-serotonin syndrome	Forest 2009, Forest 2011, Dista 2009, Alphapharm 2007, Watson 2008, Forest 2011B (Vilazodone)	expansion

Fluoxetine	Desipramine	Yes-serotonin syndrome	Forest 2009, Forest 2011, Dista 2009, Alphapharm 2007, Watson 2008, Forest 2011B (Vilazodone)	expansion
Fluoxetine	Desirudin	Yes-bleeding	Forest 2009, Forest 2011, Dista 2009, Alphapharm 2007, Watson 2008, Forest 2011B	expansion
Fluoxetine	Desvenlafaxine	Yes-serotonin syndrome	Forest 2009, Forest 2011, Dista 2009, Alphapharm 2007, Watson 2008, Forest 2011B (Vilazodone)	expansion
Fluoxetine	Dextromethorphan	Yes-serotonin syndrome	Forest 2009, Forest 2011, Dista 2009, Alphapharm 2007, Watson 2008, Forest 2011B (Vilazodone)	expansion
Fluoxetine	Diclofenac	Yes-bleeding	Forest 2009, Forest 2011, Dista 2009, Alphapharm 2007, Watson 2008, Forest 2011B	expansion
Fluoxetine	Diflunisal	Yes-bleeding	Forest 2009, Forest 2011, Dista 2009, Alphapharm 2007, Watson 2008, Forest 2011B	expansion
Fluoxetine	Doxepin	Yes-serotonin syndrome	Forest 2009, Forest 2011, Dista 2009, Alphapharm 2007, Watson 2008, Forest 2011B (Vilazodone)	expansion

Fluoxetine	Duloxetine	Yes-serotonin syndrome	Forest 2009, Forest 2011, Dista 2009, Alphapharm 2007, Watson 2008, Forest 2011B (Vilazodone)	expansion
Fluoxetine	Escitalopram	Yes-serotonin syndrome	Forest 2009, Forest 2011, Dista 2009, Alphapharm 2007, Watson 2008, Forest 2011B (Vilazodone)	expansion
Fluoxetine	Etodolac	Yes-bleeding	Forest 2009, Forest 2011, Dista 2009, Alphapharm 2007, Watson 2008, Forest 2011B	expansion
Fluoxetine	Fenoprofen	Yes-bleeding	Forest 2009, Forest 2011, Dista 2009, Alphapharm 2007, Watson 2008, Forest 2011B	expansion
Fluoxetine	Fentanyl	Yes-serotonin syndrome	Forest 2009, Forest 2011, Dista 2009, Alphapharm 2007, Watson 2008, Forest 2011B (Vilazodone)	expansion
Fluoxetine	Fluoxetine	Yes-serotonin syndrome	Forest 2009, Forest 2011, Dista 2009, Alphapharm 2007, Watson 2008, Forest 2011B (Vilazodone)	expansion
Fluoxetine	Flurbiprofen	Yes-bleeding	Forest 2009, Forest 2011, Dista 2009, Alphapharm 2007, Watson 2008, Forest 2011B	expansion

Fluoxetine	Fluvoxamine	Yes-serotonin syndrome	Forest 2009, Forest 2011, Dista 2009, Alphapharm 2007, Watson 2008, Forest 2011B (Vilazodone)	expansion
Fluoxetine	Heparin	Yes-bleeding	Forest 2009, Forest 2011, Dista 2009, Alphapharm 2007, Watson 2008, Forest 2011B	expansion
Fluoxetine	Ibuprofen	Yes-bleeding	Forest 2009, Forest 2011, Dista 2009, Alphapharm 2007, Watson 2008, Forest 2011B	expansion
Fluoxetine	Imipramine	Yes-serotonin syndrome	Forest 2009, Forest 2011, Dista 2009, Alphapharm 2007, Watson 2008, Forest 2011B (Vilazodone)	expansion
Fluoxetine	Indomethacin	Yes-bleeding	Forest 2009, Forest 2011, Dista 2009, Alphapharm 2007, Watson 2008, Forest 2011B	expansion
Fluoxetine	Isocarboxazid	Yes-serotonin syndrome	Forest 2009, Forest 2011, Dista 2009, Alphapharm 2007, Watson 2008, Forest 2011B (Vilazodone)	expansion
Fluoxetine	Ketoprofen	Yes-bleeding	Forest 2009, Forest 2011, Dista 2009, Alphapharm 2007, Watson 2008, Forest 2011B	expansion
Fluoxetine	Ketorolac tromethamine	Yes-bleeding	Forest 2009, Forest 2011, Dista 2009, Alphapharm 2007, Watson 2008, Forest 2011B	expansion

Fluoxetine	Ketorolac	Yes-bleeding	Forest 2009, Forest 2011, Dista 2009, Alphapharm 2007, Watson 2008, Forest 2011B	expansion
Fluoxetine	Lepirudin	Yes-bleeding	Forest 2009, Forest 2011, Dista 2009, Alphapharm 2007, Watson 2008, Forest 2011B	expansion
Fluoxetine	Linezolid	Yes-serotonin syndrome	Forest 2009, Forest 2011, Dista 2009, Alphapharm 2007, Watson 2008, Forest 2011B (Vilazodone)	expansion
Fluoxetine	Lithium	Yes-Lithium toxicity	Dista 2009	original
Fluoxetine	Mannitol	Yes-bleeding	Forest 2009, Forest 2011, Dista 2009, Alphapharm 2007, Watson 2008, Forest 2011B	expansion
Fluoxetine	Maprotiline	Yes-serotonin syndrome	Forest 2009, Forest 2011, Dista 2009, Alphapharm 2007, Watson 2008, Forest 2011B (Vilazodone)	expansion
Fluoxetine	Mefenamic acid	Yes-bleeding	Forest 2009, Forest 2011, Dista 2009, Alphapharm 2007, Watson 2008, Forest 2011B	expansion
Fluoxetine	Meloxicam	Yes-bleeding	Forest 2009, Forest 2011, Dista 2009, Alphapharm 2007, Watson 2008, Forest 2011B	expansion

Fluoxetine	Meperidine	Yes-serotonin syndrome	Forest 2009, Forest 2011, Dista 2009, Alphapharm 2007, Watson 2008, Forest 2011B (Vilazodone)	expansion
Fluoxetine	Milnacipran	Yes-serotonin syndrome	Forest 2009, Forest 2011, Dista 2009, Alphapharm 2007, Watson 2008, Forest 2011B (Vilazodone)	expansion
Fluoxetine	NSAID	Yes-bleeding	Forest 2009, Forest 2011, Dista 2009, Alphapharm 2007, Watson 2008, Forest 2011B	expansion
Fluoxetine	Nabumetone	Yes-bleeding	Forest 2009, Forest 2011, Dista 2009, Alphapharm 2007, Watson 2008, Forest 2011B	expansion
Fluoxetine	Naproxen	Yes-bleeding	Forest 2009, Forest 2011, Dista 2009, Alphapharm 2007, Watson 2008, Forest 2011B	expansion
Fluoxetine	Nefazodone	Yes-serotonin syndrome	Forest 2009, Forest 2011, Dista 2009, Alphapharm 2007, Watson 2008, Forest 2011B (Vilazodone)	expansion
Fluoxetine	Nepafenac	Yes-bleeding	Forest 2009, Forest 2011, Dista 2009, Alphapharm 2007, Watson 2008, Forest 2011B	expansion

Fluoxetine	Nortriptyline	Yes-serotonin syndrome	Forest 2009, Forest 2011, Dista 2009, Alphapharm 2007, Watson 2008, Forest 2011B (Vilazodone)	expansion
Fluoxetine	Odansetran	Yes-serotonin syndrome	Forest 2009, Forest 2011, Dista 2009, Alphapharm 2007, Watson 2008, Forest 2011B (Vilazodone)	expansion
Fluoxetine	Oxaprozin	Yes-bleeding	Forest 2009, Forest 2011, Dista 2009, Alphapharm 2007, Watson 2008, Forest 2011B	expansion
Fluoxetine	Paroxetine	Yes-serotonin syndrome	Forest 2009, Forest 2011, Dista 2009, Alphapharm 2007, Watson 2008, Forest 2011B (Vilazodone)	expansion
Fluoxetine	Pentazocine	Yes-serotonin syndrome	Forest 2009, Forest 2011, Dista 2009, Alphapharm 2007, Watson 2008, Forest 2011B (Vilazodone)	expansion
Fluoxetine	Phenelzine	Yes-serotonin syndrome	Forest 2009, Forest 2011, Dista 2009, Alphapharm 2007, Watson 2008, Forest 2011B (Vilazodone)	expansion
Fluoxetine	Pimozide	Yes-QTc interval prolongation	Forest 2009, Forest 2011, Dista 2009, Alphapharm 2007, Watson 2008	expansion

Fluoxetine	Piroxicam	Yes-bleeding	Forest 2009, Forest 2011, Dista 2009, Alphapharm 2007, Watson 2008, Forest 2011B	expansion
Fluoxetine	Protriptyline	Yes-serotonin syndrome	Forest 2009, Forest 2011, Dista 2009, Alphapharm 2007, Watson 2008, Forest 2011B (Vilazodone)	expansion
Fluoxetine	Rasagiline	Yes-serotonin syndrome	Forest 2009, Forest 2011, Dista 2009, Alphapharm 2007, Watson 2008, Forest 2011B (Vilazodone)	expansion
Fluoxetine	SEROTONIN-1B AND SERC	Yes-serotonin syndrome	Forest 2009, Forest 2011, Dista 2009, Alphapharm 2007, Watson 2008, Forest 2011B (Vilazodone)	expansion
Fluoxetine	SEROTONIN-1B AND SERC	Yes-weakness, hyperreflexia, and incoordination	Forest 2009, Forest 2011, Watson 2008	expansion
Fluoxetine	Salicylamide	Yes-bleeding	Forest 2009, Forest 2011, Dista 2009, Alphapharm 2007, Watson 2008, Forest 2011B	expansion
Fluoxetine	Selegiline	Yes-serotonin syndrome	Forest 2009, Forest 2011, Dista 2009, Alphapharm 2007, Watson 2008, Forest 2011B (Vilazodone)	expansion
Fluoxetine	Sertraline	Yes-serotonin syndrome	Forest 2009, Forest 2011, Dista 2009, Alphapharm 2007, Watson 2008, Forest 2011B (Vilazodone)	expansion

Fluoxetine	St Johns wort	Yes-serotonin syndrome	Forest 2009, Forest 2011, Dista 2009, Alphapharm 2007, Watson 2008, Forest 2011B (Vilazodone)	expansion
Fluoxetine	Sulindac	Yes-bleeding	Forest 2009, Forest 2011, Dista 2009, Alphapharm 2007, Watson 2008, Forest 2011B	expansion
Fluoxetine	Sumatriptan	Yes-serotonin syndrome	Forest 2009, Forest 2011, Dista 2009, Alphapharm 2007, Watson 2008, Forest 2011B (Vilazodone)	expansion
Fluoxetine	Sumatriptan	Yes-weakness, hyperreflexia, and incoordination	Forest 2009, Forest 2011, Watson 2008	expansion
Fluoxetine	Sympathomimetic	Yes-agitation, restlessness, GI distress	Dista 2009	expansion
Fluoxetine	Sympathomimetic	Yes-serotonin syndrome	Forest 2009, Forest 2011, Dista 2009, Alphapharm 2007, Watson 2008, Forest 2011B (Vilazodone)	expansion
Fluoxetine	Thioridazine	Yes-QTc interval prolongation	Dista 2009	original
Fluoxetine	Tolmetin	Yes-bleeding	Forest 2009, Forest 2011, Dista 2009, Alphapharm 2007, Watson 2008, Forest 2011B	expansion
Fluoxetine	Tramadol	Yes-serotonin syndrome	Forest 2009, Forest 2011, Dista 2009, Alphapharm 2007, Watson 2008, Forest 2011B (Vilazodone)	expansion
Fluoxetine	Tranylcypromine	Yes-serotonin syndrome	Forest 2009, Forest 2011, Dista 2009, Alphapharm 2007, Watson 2008, Forest 2011B (Vilazodone)	expansion

Fluoxetine	Trazodone	Yes-serotonin syndrome	Forest 2009, Forest 2011, Dista 2009, Alphapharm 2007, Watson 2008, Forest 2011B (Vilazodone)	expansion
Fluoxetine	Trimipramine	Yes-serotonin syndrome	Forest 2009, Forest 2011, Dista 2009, Alphapharm 2007, Watson 2008, Forest 2011B (Vilazodone)	expansion
Fluoxetine	Tryptophan	Yes-agitation, restlessness, GI distress	Dista 2009	original
Fluoxetine	VITAMIN K ANTAGONIST	Yes-bleeding	Forest 2009, Forest 2011, Dista 2009, Alphapharm 2007, Watson 2008, Forest 2011B	expansion
Fluoxetine	Venlafaxine	Yes-serotonin syndrome	Forest 2009, Forest 2011, Dista 2009, Alphapharm 2007, Watson 2008, Forest 2011B (Vilazodone)	expansion
Fluoxetine	Vilazodone	Yes-serotonin syndrome	Forest 2009, Forest 2011, Dista 2009, Alphapharm 2007, Watson 2008, Forest 2011B (Vilazodone)	expansion
Fluoxetine	Warfarin	Yes-bleeding	Forest 2009, Forest 2011, Dista 2009, Alphapharm 2007, Watson 2008, Forest 2011B	expansion
Fluvoxamine	Acetaminophen	Yes-bleeding	Forest 2009, Forest 2011, Dista 2009, Alphapharm 2007, Watson 2008, Forest 2011B	expansion

Fluvoxamine	Aminoketone	Yes-bleeding	Forest 2009, Forest 2011, Dista 2009, Alphapharm 2007, Watson 2008, Forest 2011B	expansion
Fluvoxamine	Amitriptyline	Yes-serotonin syndrome	Forest 2009, Forest 2011, Dista 2009, Alphapharm 2007, Watson 2008, Forest 2011B (Vilazodone)	expansion
Fluvoxamine	Amoxapine	Yes-serotonin syndrome	Forest 2009, Forest 2011, Dista 2009, Alphapharm 2007, Watson 2008, Forest 2011B (Vilazodone)	expansion
Fluvoxamine	Antipsychotic	Yes-QTc interval prolongation	Forest 2009, Forest 2011, Dista 2009, Alphapharm 2007, Watson 2008	expansion
Fluvoxamine	Antithrombin alfa	Yes-bleeding	Forest 2009, Forest 2011, Dista 2009, Alphapharm 2007, Watson 2008, Forest 2011B	expansion
Fluvoxamine	Argatroban	Yes-bleeding	Forest 2009, Forest 2011, Dista 2009, Alphapharm 2007, Watson 2008, Forest 2011B	expansion
Fluvoxamine	Aspirin	Yes-bleeding	Forest 2009, Forest 2011, Dista 2009, Alphapharm 2007, Watson 2008, Forest 2011B	expansion
Fluvoxamine	Bivalirudin	Yes-bleeding	Forest 2009, Forest 2011, Dista 2009, Alphapharm 2007, Watson 2008, Forest 2011B	expansion

Fluvoxamine	Bromfenac	Yes-bleeding	Forest 2009, Forest 2011, Dista 2009, Alphapharm 2007, Watson 2008, Forest 2011B	expansion
Fluvoxamine	Bupropion	Yes-bleeding	Forest 2009, Forest 2011, Dista 2009, Alphapharm 2007, Watson 2008, Forest 2011B	expansion
Fluvoxamine	Buspirone	Yes-serotonin syndrome	Forest 2009, Forest 2011, Dista 2009, Alphapharm 2007, Watson 2008, Forest 2011B (Vilazodone)	expansion
Fluvoxamine	Celecoxib	Yes-bleeding	Forest 2009, Forest 2011, Dista 2009, Alphapharm 2007, Watson 2008, Forest 2011B	expansion
Fluvoxamine	Citalopram	Yes-serotonin syndrome	Forest 2009, Forest 2011, Dista 2009, Alphapharm 2007, Watson 2008, Forest 2011B (Vilazodone)	expansion
Fluvoxamine	Clomipramine	Yes-serotonin syndrome	Forest 2009, Forest 2011, Dista 2009, Alphapharm 2007, Watson 2008, Forest 2011B (Vilazodone)	expansion
Fluvoxamine	Desipramine	Yes-serotonin syndrome	Forest 2009, Forest 2011, Dista 2009, Alphapharm 2007, Watson 2008, Forest 2011B (Vilazodone)	expansion

Fluvoxamine	Desirudin	Yes-bleeding	Forest 2009, Forest 2011, Dista 2009, Alphapharm 2007, Watson 2008, Forest 2011B	expansion
Fluvoxamine	Desvenlafaxine	Yes-serotonin syndrome	Forest 2009, Forest 2011, Dista 2009, Alphapharm 2007, Watson 2008, Forest 2011B (Vilazodone)	expansion
Fluvoxamine	Dextromethorphan	Yes-serotonin syndrome	Forest 2009, Forest 2011, Dista 2009, Alphapharm 2007, Watson 2008, Forest 2011B (Vilazodone)	expansion
Fluvoxamine	Diclofenac	Yes-bleeding	Forest 2009, Forest 2011, Dista 2009, Alphapharm 2007, Watson 2008, Forest 2011B	expansion
Fluvoxamine	Diflunisal	Yes-bleeding	Forest 2009, Forest 2011, Dista 2009, Alphapharm 2007, Watson 2008, Forest 2011B	expansion
Fluvoxamine	Doxepin	Yes-serotonin syndrome	Forest 2009, Forest 2011, Dista 2009, Alphapharm 2007, Watson 2008, Forest 2011B (Vilazodone)	expansion
Fluvoxamine	Duloxetine	Yes-serotonin syndrome	Forest 2009, Forest 2011, Dista 2009, Alphapharm 2007, Watson 2008, Forest 2011B (Vilazodone)	expansion

Fluvoxamine	Escitalopram	Yes-serotonin syndrome	Forest 2009, Forest 2011, Dista 2009, Alphapharm 2007, Watson 2008, Forest 2011B (Vilazodone)	expansion
Fluvoxamine	Etodolac	Yes-bleeding	Forest 2009, Forest 2011, Dista 2009, Alphapharm 2007, Watson 2008, Forest 2011B	expansion
Fluvoxamine	Fenoprofen	Yes-bleeding	Forest 2009, Forest 2011, Dista 2009, Alphapharm 2007, Watson 2008, Forest 2011B	expansion
Fluvoxamine	Fentanyl	Yes-serotonin syndrome	Forest 2009, Forest 2011, Dista 2009, Alphapharm 2007, Watson 2008, Forest 2011B (Vilazodone)	expansion
Fluvoxamine	Fluoxetine	Yes-serotonin syndrome	Forest 2009, Forest 2011, Dista 2009, Alphapharm 2007, Watson 2008, Forest 2011B (Vilazodone)	expansion
Fluvoxamine	Flurbiprofen	Yes-bleeding	Forest 2009, Forest 2011, Dista 2009, Alphapharm 2007, Watson 2008, Forest 2011B	expansion
Fluvoxamine	Fluvoxamine	Yes-serotonin syndrome	Forest 2009, Forest 2011, Dista 2009, Alphapharm 2007, Watson 2008, Forest 2011B (Vilazodone)	expansion

Fluvoxamine	Heparin	Yes-bleeding	Forest 2009, Forest 2011, Dista 2009, Alphapharm 2007, Watson 2008, Forest 2011B	expansion
Fluvoxamine	Ibuprofen	Yes-bleeding	Forest 2009, Forest 2011, Dista 2009, Alphapharm 2007, Watson 2008, Forest 2011B	expansion
Fluvoxamine	Imipramine	Yes-serotonin syndrome	Forest 2009, Forest 2011, Dista 2009, Alphapharm 2007, Watson 2008, Forest 2011B (Vilazodone)	expansion
Fluvoxamine	Indomethacin	Yes-bleeding	Forest 2009, Forest 2011, Dista 2009, Alphapharm 2007, Watson 2008, Forest 2011B	expansion
Fluvoxamine	Isocarboxazid	Yes-serotonin syndrome	Forest 2009, Forest 2011, Dista 2009, Alphapharm 2007, Watson 2008, Forest 2011B (Vilazodone)	expansion
Fluvoxamine	Ketoprofen	Yes-bleeding	Forest 2009, Forest 2011, Dista 2009, Alphapharm 2007, Watson 2008, Forest 2011B	expansion
Fluvoxamine	Ketorolac tromethamine	Yes-bleeding	Forest 2009, Forest 2011, Dista 2009, Alphapharm 2007, Watson 2008, Forest 2011B	expansion
Fluvoxamine	Ketorolac	Yes-bleeding	Forest 2009, Forest 2011, Dista 2009, Alphapharm 2007, Watson 2008, Forest 2011B	expansion

Fluvoxamine	Lepirudin	Yes-bleeding	Forest 2009, Forest 2011, Dista 2009, Alphapharm 2007, Watson 2008, Forest 2011B	expansion
Fluvoxamine	Linezolid	Yes-serotonin syndrome	Forest 2009, Forest 2011, Dista 2009, Alphapharm 2007, Watson 2008, Forest 2011B (Vilazodone)	expansion
Fluvoxamine	Mannitol	Yes-bleeding	Forest 2009, Forest 2011, Dista 2009, Alphapharm 2007, Watson 2008, Forest 2011B	expansion
Fluvoxamine	Maprotiline	Yes-serotonin syndrome	Forest 2009, Forest 2011, Dista 2009, Alphapharm 2007, Watson 2008, Forest 2011B (Vilazodone)	expansion
Fluvoxamine	Mefenamic acid	Yes-bleeding	Forest 2009, Forest 2011, Dista 2009, Alphapharm 2007, Watson 2008, Forest 2011B	expansion
Fluvoxamine	Meloxicam	Yes-bleeding	Forest 2009, Forest 2011, Dista 2009, Alphapharm 2007, Watson 2008, Forest 2011B	expansion
Fluvoxamine	Meperidine	Yes-serotonin syndrome	Forest 2009, Forest 2011, Dista 2009, Alphapharm 2007, Watson 2008, Forest 2011B (Vilazodone)	expansion

Fluvoxamine	Milnacipran	Yes-serotonin syndrome	Forest 2009, Forest 2011, Dista 2009, Alphapharm 2007, Watson 2008, Forest 2011B (Vilazodone)	expansion
Fluvoxamine	NSAID	Yes-bleeding	Forest 2009, Forest 2011, Dista 2009, Alphapharm 2007, Watson 2008, Forest 2011B	expansion
Fluvoxamine	Nabumetone	Yes-bleeding	Forest 2009, Forest 2011, Dista 2009, Alphapharm 2007, Watson 2008, Forest 2011B	expansion
Fluvoxamine	Naproxen	Yes-bleeding	Forest 2009, Forest 2011, Dista 2009, Alphapharm 2007, Watson 2008, Forest 2011B	expansion
Fluvoxamine	Nefazodone	Yes-serotonin syndrome	Forest 2009, Forest 2011, Dista 2009, Alphapharm 2007, Watson 2008, Forest 2011B (Vilazodone)	expansion
Fluvoxamine	Nepafenac	Yes-bleeding	Forest 2009, Forest 2011, Dista 2009, Alphapharm 2007, Watson 2008, Forest 2011B	expansion
Fluvoxamine	Nortriptyline	Yes-serotonin syndrome	Forest 2009, Forest 2011, Dista 2009, Alphapharm 2007, Watson 2008, Forest 2011B (Vilazodone)	expansion

Fluvoxamine	Odansetran	Yes-serotonin syndrome	Forest 2009, Forest 2011, Dista 2009, Alphapharm 2007, Watson 2008, Forest 2011B (Vilazodone)	expansion
Fluvoxamine	Oxaprozin	Yes-bleeding	Forest 2009, Forest 2011, Dista 2009, Alphapharm 2007, Watson 2008, Forest 2011B	expansion
Fluvoxamine	Paroxetine	Yes-serotonin syndrome	Forest 2009, Forest 2011, Dista 2009, Alphapharm 2007, Watson 2008, Forest 2011B (Vilazodone)	expansion
Fluvoxamine	Pentazocine	Yes-serotonin syndrome	Forest 2009, Forest 2011, Dista 2009, Alphapharm 2007, Watson 2008, Forest 2011B (Vilazodone)	expansion
Fluvoxamine	Phenelzine	Yes-serotonin syndrome	Forest 2009, Forest 2011, Dista 2009, Alphapharm 2007, Watson 2008, Forest 2011B (Vilazodone)	expansion
Fluvoxamine	Pimozide	Yes-QTc interval prolongation	Forest 2009, Forest 2011, Dista 2009, Alphapharm 2007, Watson 2008	expansion
Fluvoxamine	Piroxicam	Yes-bleeding	Forest 2009, Forest 2011, Dista 2009, Alphapharm 2007, Watson 2008, Forest 2011B	expansion

Fluvoxamine	Protriptyline	Yes-serotonin syndrome	Forest 2009, Forest 2011, Dista 2009, Alphapharm 2007, Watson 2008, Forest 2011B (Vilazodone)	expansion
Fluvoxamine	Rasagiline	Yes-serotonin syndrome	Forest 2009, Forest 2011, Dista 2009, Alphapharm 2007, Watson 2008, Forest 2011B (Vilazodone)	expansion
Fluvoxamine	SEROTONIN-1B AND SERC	Yes-serotonin syndrome	Forest 2009, Forest 2011, Dista 2009, Alphapharm 2007, Watson 2008, Forest 2011B (Vilazodone)	expansion
Fluvoxamine	SEROTONIN-1B AND SERC	Yes-weakness, hyperreflexia, and incoordination	Forest 2009, Forest 2011, Watson 2008	expansion
Fluvoxamine	Salicylamide	Yes-bleeding	Forest 2009, Forest 2011, Dista 2009, Alphapharm 2007, Watson 2008, Forest 2011B	expansion
Fluvoxamine	Selegiline	Yes-serotonin syndrome	Forest 2009, Forest 2011, Dista 2009, Alphapharm 2007, Watson 2008, Forest 2011B (Vilazodone)	expansion
Fluvoxamine	Sertraline	Yes-serotonin syndrome	Forest 2009, Forest 2011, Dista 2009, Alphapharm 2007, Watson 2008, Forest 2011B (Vilazodone)	expansion
Fluvoxamine	St Johns wort	Yes-serotonin syndrome	Forest 2009, Forest 2011, Dista 2009, Alphapharm 2007, Watson 2008, Forest 2011B (Vilazodone)	expansion

Fluvoxamine	Sulindac	Yes-bleeding	Forest 2009, Forest 2011, Dista 2009, Alphapharm 2007, Watson 2008, Forest 2011B	expansion
Fluvoxamine	Sumatriptan	Yes-serotonin syndrome	Forest 2009, Forest 2011, Dista 2009, Alphapharm 2007, Watson 2008, Forest 2011B (Vilazodone)	expansion
Fluvoxamine	Sumatriptan	Yes-weakness, hyperreflexia, and incoordination	Forest 2009, Forest 2011, Watson 2008	expansion
Fluvoxamine	Sympathomimetic	Yes-serotonin syndrome	Forest 2009, Forest 2011, Dista 2009, Alphapharm 2007, Watson 2008, Forest 2011B (Vilazodone)	expansion
Fluvoxamine	Tolmetin	Yes-bleeding	Forest 2009, Forest 2011, Dista 2009, Alphapharm 2007, Watson 2008, Forest 2011B	expansion
Fluvoxamine	Tramadol	Yes-serotonin syndrome	Forest 2009, Forest 2011, Dista 2009, Alphapharm 2007, Watson 2008, Forest 2011B (Vilazodone)	expansion
Fluvoxamine	Tranylcypromine	Yes-serotonin syndrome	Forest 2009, Forest 2011, Dista 2009, Alphapharm 2007, Watson 2008, Forest 2011B (Vilazodone)	expansion
Fluvoxamine	Trazodone	Yes-serotonin syndrome	Forest 2009, Forest 2011, Dista 2009, Alphapharm 2007, Watson 2008, Forest 2011B (Vilazodone)	expansion

Fluvoxamine	Trimipramine	Yes-serotonin syndrome	Forest 2009, Forest 2011, Dista 2009, Alphapharm 2007, Watson 2008, Forest 2011B (Vilazodone)	expansion
Fluvoxamine	Tryptophan	Yes-serotonin syndrome	Forest 2009, Forest 2011, Dista 2009, Alphapharm 2007, Watson 2008, Forest 2011B (Vilazodone)	expansion
Fluvoxamine	VITAMIN K ANTAGONIST	Yes-bleeding	Forest 2009, Forest 2011, Dista 2009, Alphapharm 2007, Watson 2008, Forest 2011B	expansion
Fluvoxamine	Venlafaxine	Yes-serotonin syndrome	Forest 2009, Forest 2011, Dista 2009, Alphapharm 2007, Watson 2008, Forest 2011B (Vilazodone)	expansion
Fluvoxamine	Vilazodone	Yes-serotonin syndrome	Forest 2009, Forest 2011, Dista 2009, Alphapharm 2007, Watson 2008, Forest 2011B (Vilazodone)	expansion
Fluvoxamine	Warfarin	Yes-bleeding	Forest 2009, Forest 2011, Dista 2009, Alphapharm 2007, Watson 2008, Forest 2011B	expansion
Imipramine	4-hydroxybutanoic acid	Yes-additive CNS depressant effects	Sandoz 2010b, Duramed 2007	expansion
Imipramine	Amantadine	Yes	Sandoz 2010b, Duramed 2007	expansion
Imipramine	Anticholinergic	Yes	Sandoz 2010b, Duramed 2007	original
Imipramine	Atropine	Yes	Sandoz 2010b, Duramed 2007	expansion
Imipramine	Benztropine	Yes	Sandoz 2010b, Duramed 2007	expansion
Imipramine	Biperiden	Yes	Sandoz 2010b, Duramed 2007	expansion

Imipramine	CNS depressants	Yes-additive CNS depressant effects	Sandoz 2010b, Duramed 2007	original
Imipramine	Dicyclomine	Yes	Sandoz 2010b, Duramed 2007	expansion
Imipramine	Difenoxin	Yes	Sandoz 2010b, Duramed 2007	expansion
Imipramine	Diphenoxylate	Yes	Sandoz 2010b, Duramed 2007	expansion
Imipramine	Glycopyrronium	Yes	Sandoz 2010b, Duramed 2007	expansion
Imipramine	Hyoscyamine	Yes	Sandoz 2010b, Duramed 2007	expansion
Imipramine	Ipratropium cation	Yes	Sandoz 2010b, Duramed 2007	expansion
Imipramine	Ipratropium	Yes	Sandoz 2010b, Duramed 2007	expansion
Imipramine	Isocarboxazid	Yes-Hyperpyretic crises, severe convulsions, death	Mylan 2009, Sandoz 2010, Watson 2007 (doxepin), Mylan 2007, Sandoz 2010b, Watson 2009, Duramed 2007, Ciba-Gigy 1996	expansion
Imipramine	Levodopa	Yes-additive CNS depressant effects	Sandoz 2010b, Duramed 2007	expansion
Imipramine	Mepenzolate	Yes	Sandoz 2010b, Duramed 2007	expansion
Imipramine	Methscopolamine	Yes	Sandoz 2010b, Duramed 2007	expansion
Imipramine	Methylphenidate	Yes-additive CNS depressant effects	Sandoz 2010b, Duramed 2007	expansion
Imipramine	Phenelzine	Yes-Hyperpyretic crises, severe convulsions, death	Mylan 2009, Sandoz 2010, Watson 2007 (doxepin), Mylan 2007, Sandoz 2010b, Watson 2009, Duramed 2007, Ciba-Gigy 1996	expansion
Imipramine	Phenobarbital	Yes	Sandoz 2010b, Duramed 2007	expansion
Imipramine	Procyclidine	Yes	Sandoz 2010b, Duramed 2007	expansion
Imipramine	Propantheline	Yes	Sandoz 2010b, Duramed 2007	expansion
Imipramine	Pseudoephedrine	Yes	Sandoz 2010b, Duramed 2007	expansion
Imipramine	Rasagiline	Yes-Hyperpyretic crises, severe convulsions, death	Mylan 2009, Sandoz 2010, Watson 2007 (doxepin), Mylan 2007, Sandoz 2010b, Watson 2009, Duramed 2007, Ciba-Gigy 1996	expansion

Imipramine	Scopolamine	Yes	Sandoz 2010b, Duramed 2007	expansion
Imipramine	Selegiline	Yes-Hyperpyretic crises, severe convulsions, death	Mylan 2009, Sandoz 2010, Watson 2007 (doxepin), Mylan 2007, Sandoz 2010b, Watson 2009, Duramed 2007, Ciba-Gigy 1996	expansion
Imipramine	Tiotropium	Yes	Sandoz 2010b, Duramed 2007	expansion
Imipramine	Tranylcypromine	Yes-Hyperpyretic crises, severe convulsions, death	Mylan 2009, Sandoz 2010, Watson 2007 (doxepin), Mylan 2007, Sandoz 2010b, Watson 2009, Duramed 2007, Ciba-Gigy 1996	expansion
Imipramine	Tropicamide	Yes	Sandoz 2010b, Duramed 2007	expansion
Isocarboxazid	Amitriptyline	Yes-serotonin syndrome	Dey 2010, Validus 2007, Parke-Davis 2011, GSK 2010	expansion
Isocarboxazid	Amoxapine	Yes-serotonin syndrome	Dey 2010, Validus 2007, Parke-Davis 2011, GSK 2010	expansion
Isocarboxazid	Anticholinergic	Yes-hypertension	Dey 2010, Validus 2007, Parke-Davis 2011, GSK 2010	expansion
Isocarboxazid	Buspirone	Yes-hypertension	Dey 2010, Validus 2007, Parke-Davis 2011, GSK 2010	expansion
Isocarboxazid	Citalopram	Yes-serotonin syndrome	Dey 2010, Validus 2007, Parke-Davis 2011, GSK 2010	expansion
Isocarboxazid	Clomipramine	Yes-serotonin syndrome	Dey 2010, Validus 2007, Parke-Davis 2011, GSK 2010	expansion
Isocarboxazid	Desipramine	Yes-serotonin syndrome	Dey 2010, Validus 2007, Parke-Davis 2011, GSK 2010	expansion
Isocarboxazid	Desvenlafaxine	Yes-serotonin syndrome	Dey 2010, Validus 2007, Parke-Davis 2011, GSK 2010	expansion

Isocarboxazid	Dextromethorphan	Yes-serotonin syndrome	Dey 2010, Validus 2007, Parke-Davis 2011, GSK 2010	expansion
Isocarboxazid	Doxepin	Yes-serotonin syndrome	Dey 2010, Validus 2007, Parke-Davis 2011, GSK 2010	expansion
Isocarboxazid	Duloxetine	Yes-serotonin syndrome	Dey 2010, Validus 2007, Parke-Davis 2011, GSK 2010	expansion
Isocarboxazid	Escitalopram	Yes-serotonin syndrome	Dey 2010, Validus 2007, Parke-Davis 2011, GSK 2010	expansion
Isocarboxazid	Fentanyl	Yes-serotonin syndrome	Dey 2010, Validus 2007, Parke-Davis 2011, GSK 2010	expansion
Isocarboxazid	Fluoxetine	Yes-serotonin syndrome	Dey 2010, Validus 2007, Parke-Davis 2011, GSK 2010	expansion
Isocarboxazid	Fluvoxamine	Yes-serotonin syndrome	Dey 2010, Validus 2007, Parke-Davis 2011, GSK 2010	expansion
Isocarboxazid	Imipramine	Yes-serotonin syndrome	Dey 2010, Validus 2007, Parke-Davis 2011, GSK 2010	expansion
Isocarboxazid	Isocarboxazid	Yes-serotonin syndrome	Dey 2010, Validus 2007, Parke-Davis 2011, GSK 2010	expansion
Isocarboxazid	Linezolid	Yes-serotonin syndrome	Dey 2010, Validus 2007, Parke-Davis 2011, GSK 2010	expansion
Isocarboxazid	Maprotiline	Yes-serotonin syndrome	Dey 2010, Validus 2007, Parke-Davis 2011, GSK 2010	expansion
Isocarboxazid	Meperidine	Yes- coma, severe hypertension or hypotension, se	Dey 2010, Validus 2007, Parke-Davis 2011, GSK 2010	expansion
Isocarboxazid	Milnacipran	Yes-serotonin syndrome	Dey 2010, Validus 2007, Parke-Davis 2011, GSK 2010	expansion
Isocarboxazid	Modafinil	Yes-hypertension	Dey 2010, Validus 2007, Parke-Davis 2011, GSK 2010	expansion

Isocarboxazid	Nefazodone	Yes-serotonin syndrome	Dey 2010, Validus 2007, Parke-Davis 2011, GSK 2010	expansion
Isocarboxazid	Nortriptyline	Yes-serotonin syndrome	Dey 2010, Validus 2007, Parke-Davis 2011, GSK 2010	expansion
Isocarboxazid	Odansetran	Yes-serotonin syndrome	Dey 2010, Validus 2007, Parke-Davis 2011, GSK 2010	expansion
Isocarboxazid	Paroxetine	Yes-serotonin syndrome	Dey 2010, Validus 2007, Parke-Davis 2011, GSK 2010	expansion
Isocarboxazid	Pentazocine	Yes-serotonin syndrome	Dey 2010, Validus 2007, Parke-Davis 2011, GSK 2010	expansion
Isocarboxazid	Phenelzine	Yes-serotonin syndrome	Dey 2010, Validus 2007, Parke-Davis 2011, GSK 2010	expansion
Isocarboxazid	Phenylpropanolamine	Yes-hypertension	Dey 2010, Validus 2007, Parke-Davis 2011, GSK 2010	expansion
Isocarboxazid	Protriptyline	Yes-serotonin syndrome	Dey 2010, Validus 2007, Parke-Davis 2011, GSK 2010	expansion
Isocarboxazid	Pseudoephedrine	Yes-hypertension	Dey 2010, Validus 2007, Parke-Davis 2011, GSK 2010	expansion
Isocarboxazid	Rasagiline	Yes-serotonin syndrome	Dey 2010, Validus 2007, Parke-Davis 2011, GSK 2010	expansion
Isocarboxazid	SEROTONIN-1B AND SERC	Yes-serotonin syndrome	Dey 2010, Validus 2007, Parke-Davis 2011, GSK 2010	expansion
Isocarboxazid	Selegiline	Yes-serotonin syndrome	Dey 2010, Validus 2007, Parke-Davis 2011, GSK 2010	expansion
Isocarboxazid	Sertraline	Yes-serotonin syndrome	Dey 2010, Validus 2007, Parke-Davis 2011, GSK 2010	expansion
Isocarboxazid	St Johns wort	Yes-serotonin syndrome	Dey 2010, Validus 2007, Parke-Davis 2011, GSK 2010	expansion

Isocarboxazid	Sumatriptan	Yes-serotonin syndrome	Dey 2010, Validus 2007, Parke-Davis 2011, GSK 2010	expansion
Isocarboxazid	Sympathomimetic	Yes-hypertension	Dey 2010, Validus 2007, Parke-Davis 2011, GSK 2010	expansion
Isocarboxazid	Sympathomimetic	Yes-serotonin syndrome	Dey 2010, Validus 2007, Parke-Davis 2011, GSK 2010	expansion
Isocarboxazid	Tramadol	Yes-serotonin syndrome	Dey 2010, Validus 2007, Parke-Davis 2011, GSK 2010	expansion
Isocarboxazid	Tranylcypromine	Yes-serotonin syndrome	Dey 2010, Validus 2007, Parke-Davis 2011, GSK 2010	expansion
Isocarboxazid	Trazodone	Yes-serotonin syndrome	Dey 2010, Validus 2007, Parke-Davis 2011, GSK 2010	expansion
Isocarboxazid	Trimipramine	Yes-serotonin syndrome	Dey 2010, Validus 2007, Parke-Davis 2011, GSK 2010	expansion
Isocarboxazid	Tryptophan	Yes-hypertension	Dey 2010, Validus 2007, Parke-Davis 2011, GSK 2010	expansion
Isocarboxazid	Tryptophan	Yes-serotonin syndrome	Dey 2010, Validus 2007, Parke-Davis 2011, GSK 2010	expansion
Isocarboxazid	Venlafaxine	Yes-serotonin syndrome	Dey 2010, Validus 2007, Parke-Davis 2011, GSK 2010	expansion
Isocarboxazid	Vilazodone	Yes-serotonin syndrome	Dey 2010, Validus 2007, Parke-Davis 2011, GSK 2010	expansion
MAOI	Alprazolam	No	Dey 2010	expansion
MAOI	Anticholinergic	Yes-hypertension	Dey 2010, Validus 2007, Parke-Davis 2011, GSK 2010	expansion
MAOI	Antipsychotic	No	Dey 2010	expansion
MAOI	Antipsychotic	Yes-seizure	GSK 2010	expansion
MAOI	Aripiprazole	Yes-seizure	GSK 2010	expansion
MAOI	Asenapine	Yes-seizure	GSK 2010	expansion
MAOI	Azole Antifungal	No	Dey 2010	expansion
MAOI	Benzodiazepine	No	Dey 2010	expansion
MAOI	Buspirone	Yes-hypertension	Dey 2010, Validus 2007, Parke-Davis 2011, GSK 2010	original

MAOI	CNS depressants	Yes-hypertension	GSK-2010	expansion
MAOI	Chlorpromazine	Yes-seizure	GSK 2010	expansion
MAOI	Clozapine	Yes-seizure	GSK 2010	expansion
MAOI	Dextromethorphan	Yes-serotonin syndrome	Dey 2010, Validus 2007, Parke-Davis 2011, GSK 2010	original
MAOI	Dopamine	Yes-hypertension	GSK-2010	expansion
MAOI	Fentanyl	Yes-serotonin syndrome	Dey 2010, Validus 2007, Parke-Davis 2011, GSK 2010	original
MAOI	Guanethidine	Yes-hypertension	GSK-2010	expansion
MAOI	Guanethidine	Yes-hypertension	Parke-Davis 2011	expansion
MAOI	Haloperidol	Yes-seizure	GSK 2010	expansion
MAOI	Ibuprofen	No	Dey 2010	expansion
MAOI	Iloperidone	Yes-seizure	GSK 2010	expansion
MAOI	Ketoconazole	No	Dey 2010	expansion
MAOI	Levodopa	Yes-hypertension	GSK-2010	expansion
MAOI	Levothyroxine	???	???	expansion
MAOI	Linezolid	Yes-serotonin syndrome	Dey 2010, Validus 2007, Parke-Davis 2011, GSK 2010	original
MAOI	Lithium	Yes-seizure	GSK 2010	expansion
MAOI	MAOI	Yes-serotonin syndrome	Dey 2010, Validus 2007, Parke-Davis 2011, GSK 2010	original
MAOI	Meperidine	Yes- coma, severe hypertension or hypotension, se	Dey 2010, Validus 2007, Parke-Davis 2011, GSK 2010	original
MAOI	Methyldopa	Yes-hypertension	GSK-2010	expansion
MAOI	Methylxanthine	Yes-seizure	GSK 2010	expansion
MAOI	NSAID	No	Dey 2010	expansion
MAOI	Odansetran	Yes-serotonin syndrome	Dey 2010, Validus 2007, Parke-Davis 2011, GSK 2010	original
MAOI	Olanzapine	No	Dey 2010	expansion
MAOI	Olanzapine	Yes-seizure	GSK 2010	expansion
MAOI	Paliperidone	Yes-seizure	GSK 2010	expansion
MAOI	Pentazocine	Yes-serotonin syndrome	Dey 2010, Validus 2007, Parke-Davis 2011, GSK 2010	original
MAOI	Perphenazine	Yes-seizure	GSK 2010	expansion
MAOI	Phenylpropanolamine	Yes-hypertension	Dey 2010, Validus 2007, Parke-Davis 2011, GSK 2010	original
MAOI	Pimozide	Yes-seizure	GSK 2010	expansion
MAOI	Pseudoephedrine	Yes-hypertension	Dey 2010, Validus 2007, Parke-Davis 2011, GSK 2010	original
MAOI	Quetiapine	Yes-seizure	GSK 2010	expansion
MAOI	Reserpine	Yes-hypertension	GSK-2010	expansion

MAOI	Risperidone	No	Dey 2010	expansion
MAOI	Risperidone	Yes-seizure	GSK 2010	expansion
MAOI	SEROTONIN-1B AND SERC	Yes-serotonin syndrome	Dey 2010, Validus 2007, Parke-Davis 2011, GSK 2010	expansion
MAOI	SNRI	Yes-serotonin syndrome	Dey 2010, Validus 2007, Parke-Davis 2011, GSK 2010	original
MAOI	SSRI	Yes-serotonin syndrome	Dey 2010, Validus 2007, Parke-Davis 2011, GSK 2010	original
MAOI	St Johns wort	Yes-serotonin syndrome	Dey 2010, Validus 2007, Parke-Davis 2011, GSK 2010	original
MAOI	Sumatriptan	Yes-serotonin syndrome	Dey 2010, Validus 2007, Parke-Davis 2011, GSK 2010	original
MAOI	Sympathomimetic	Yes-hypertension	Dey 2010, Validus 2007, Parke-Davis 2011, GSK 2010	original
MAOI	TCA	Yes-serotonin syndrome	Dey 2010, Validus 2007, Parke-Davis 2011, GSK 2010	original
MAOI	Theophylline	Yes-seizure	GSK 2010	expansion
MAOI	Thioridazine	Yes-seizure	GSK 2010	expansion
MAOI	Thioridizine	Yes-seizure	GSK 2010	expansion
MAOI	Thiothixene	Yes-seizure	GSK 2010	expansion
MAOI	Tramadol	Yes-serotonin syndrome	Dey 2010, Validus 2007, Parke-Davis 2011, GSK 2010	original
MAOI	Tryptophan	Yes-hypertension	Dey 2010, Validus 2007, Parke-Davis 2011, GSK 2010	original
MAOI	Tryptophan	Yes-serotonin syndrome	Dey 2010, Validus 2007, Parke-Davis 2011, GSK 2010	original
MAOI	Ziprasidone	Yes-seizure	GSK 2010	expansion
Maprotiline	Amantadine	Yes-additive side-effects	Mylan 2008	expansion
Maprotiline	Amitriptyline	Yes-serotonin syndrome	Mylan 2009	expansion
Maprotiline	Amoxapine	Yes-serotonin syndrome	Mylan 2009	expansion
Maprotiline	Anticholinergic	Yes-additive side-effects	Mylan 2008	original
Maprotiline	Atropine	Yes-additive side-effects	Mylan 2008	expansion
Maprotiline	Benztropine	Yes-additive side-effects	Mylan 2008	expansion
Maprotiline	Biperiden	Yes-additive side-effects	Mylan 2008	expansion
Maprotiline	Buspirone	Yes-serotonin syndrome	Mylan 2009	original
Maprotiline	Citalopram	Yes-serotonin syndrome	Mylan 2009	expansion
Maprotiline	Clomipramine	Yes-serotonin syndrome	Mylan 2009	expansion
Maprotiline	Desipramine	Yes-serotonin syndrome	Mylan 2009	expansion
Maprotiline	Desvenlafaxine	Yes-serotonin syndrome	Mylan 2009	expansion

Maprotiline	Dextromethorphan	Yes-serotonin syndrome	Mylan 2009	original
Maprotiline	Dicyclomine	Yes-additive side-effects	Mylan 2008	expansion
Maprotiline	Difenoxin	Yes-additive side-effects	Mylan 2008	expansion
Maprotiline	Diphenoxylate	Yes-additive side-effects	Mylan 2008	expansion
Maprotiline	Doxepin	Yes-serotonin syndrome	Mylan 2009	expansion
Maprotiline	Duloxetine	Yes-serotonin syndrome	Mylan 2009	expansion
Maprotiline	Escitalopram	Yes-serotonin syndrome	Mylan 2009	expansion
Maprotiline	Fentanyl	Yes-serotonin syndrome	Mylan 2009	original
Maprotiline	Fluoxetine	Yes-serotonin syndrome	Mylan 2009	expansion
Maprotiline	Fluvoxamine	Yes-serotonin syndrome	Mylan 2009	expansion
Maprotiline	Glycopyrronium	Yes-additive side-effects	Mylan 2008	expansion
Maprotiline	Hyoscyamine	Yes-additive side-effects	Mylan 2008	expansion
Maprotiline	Imipramine	Yes-serotonin syndrome	Mylan 2009	expansion
Maprotiline	Ipratropium cation	Yes-additive side-effects	Mylan 2008	expansion
Maprotiline	Ipratropium	Yes-additive side-effects	Mylan 2008	expansion
Maprotiline	Isocarboxazid	Yes-Hyperpyretic crises, severe convulsions, death	Mylan 2009, Sandoz 2010, Watson 2007 (doxepin), Mylan 2007, Sandoz 2010b, Watson 2009, Duramed 2007, Ciba-Gigy 1996	expansion
Maprotiline	Isocarboxazid	Yes-serotonin syndrome	Mylan 2009	expansion
Maprotiline	Linezolid	Yes-serotonin syndrome	Mylan 2009	original
Maprotiline	MAOI	Yes-serotonin syndrome	Mylan 2009	original
Maprotiline	Maprotiline	Yes-serotonin syndrome	Mylan 2009	expansion
Maprotiline	Mepenzolate	Yes-additive side-effects	Mylan 2008	expansion
Maprotiline	Meperidine	Yes-serotonin syndrome	Mylan 2009	original
Maprotiline	Methscopolamine	Yes-additive side-effects	Mylan 2008	expansion
Maprotiline	Milnacipran	Yes-serotonin syndrome	Mylan 2009	expansion
Maprotiline	Modafinil	Yes-additive side-effects	Mylan 2008	expansion
Maprotiline	Nefazodone	Yes-serotonin syndrome	Mylan 2009	expansion
Maprotiline	Nortriptyline	Yes-serotonin syndrome	Mylan 2009	expansion
Maprotiline	Odansetran	Yes-serotonin syndrome	Mylan 2009	original
Maprotiline	Paroxetine	Yes-serotonin syndrome	Mylan 2009	expansion
Maprotiline	Pentazocine	Yes-serotonin syndrome	Mylan 2009	original
Maprotiline	Phenelzine	Yes-Hyperpyretic crises, severe convulsions, death	Mylan 2009, Sandoz 2010, Watson 2007 (doxepin), Mylan 2007, Sandoz 2010b, Watson 2009, Duramed 2007, Ciba-Gigy 1996	expansion
Maprotiline	Phenelzine	Yes-serotonin syndrome	Mylan 2009	expansion
Maprotiline	Phenobarbital	Yes-additive side-effects	Mylan 2008	expansion
Maprotiline	Phenylpropanolamine	Yes-additive side-effects	Mylan 2008	expansion
Maprotiline	Procyclidine	Yes-additive side-effects	Mylan 2008	expansion
Maprotiline	Propantheline	Yes-additive side-effects	Mylan 2008	expansion
Maprotiline	Protriptyline	Yes-serotonin syndrome	Mylan 2009	expansion
Maprotiline	Pseudoephedrine	Yes-additive side-effects	Mylan 2008	expansion

Maprotiline	Rasagiline	Yes-Hyperpyretic crises, severe convulsions, death	Mylan 2009, Sandoz 2010, Watson 2007 (doxepin), Mylan 2007, Sandoz 2010b, Watson 2009, Duramed 2007, Ciba-Gigy 1996	expansion
Maprotiline	Rasagiline	Yes-serotonin syndrome	Mylan 2009	expansion
Maprotiline	SEROTONIN-1B AND SERC	Yes-serotonin syndrome	Mylan 2009	expansion
Maprotiline	SNRI	Yes-serotonin syndrome	Mylan 2009	original
Maprotiline	SSRI	Yes-serotonin syndrome	Mylan 2009	original
Maprotiline	Scopolamine	Yes-additive side-effects	Mylan 2008	expansion
Maprotiline	Selegiline	Yes-Hyperpyretic crises, severe convulsions, death	Mylan 2009, Sandoz 2010, Watson 2007 (doxepin), Mylan 2007, Sandoz 2010b, Watson 2009, Duramed 2007, Ciba-Gigy 1996	expansion
Maprotiline	Selegiline	Yes-serotonin syndrome	Mylan 2009	expansion
Maprotiline	Sertraline	Yes-serotonin syndrome	Mylan 2009	expansion
Maprotiline	St Johns wort	Yes-serotonin syndrome	Mylan 2009	original
Maprotiline	Sumatriptan	Yes-serotonin syndrome	Mylan 2009	original
Maprotiline	Sympathomimetic	Yes-additive side-effects	Mylan 2008	original
Maprotiline	TCA	Yes-serotonin syndrome	Mylan 2009	original
Maprotiline	Thyroid medications	Yes-cardiovascular toxicity	Mylan 2008	original
Maprotiline	Tiotropium	Yes-additive side-effects	Mylan 2008	expansion
Maprotiline	Tramadol	Yes-serotonin syndrome	Mylan 2009	original
Maprotiline	Tranylcypromine	Yes-Hyperpyretic crises, severe convulsions, death	Mylan 2009, Sandoz 2010, Watson 2007 (doxepin), Mylan 2007, Sandoz 2010b, Watson 2009, Duramed 2007, Ciba-Gigy 1996	expansion
Maprotiline	Tranylcypromine	Yes-serotonin syndrome	Mylan 2009	expansion
Maprotiline	Trazodone	Yes-serotonin syndrome	Mylan 2009	expansion
Maprotiline	Trimipramine	Yes-serotonin syndrome	Mylan 2009	expansion
Maprotiline	Tropicamide	Yes-additive side-effects	Mylan 2008	expansion
Maprotiline	Tryptophan	Yes-serotonin syndrome	Mylan 2009	original
Maprotiline	Venlafaxine	Yes-serotonin syndrome	Mylan 2009	expansion
Maprotiline	Vilazodone	Yes-serotonin syndrome	Mylan 2009	expansion
Methylphenidate	Caffeine	Yes-behavior disturbance and thought disorder	Physicians 2009	expansion
Methylphenidate	Chlorpheniramine	Yes-behavior disturbance and thought disorder	Physicians 2009	expansion
Methylphenidate	Dexmethylphenidate	Yes-behavior disturbance and thought disorder	Physicians 2009	expansion
Methylphenidate	Dextroamphetamine	Yes-behavior disturbance and thought disorder	Physicians 2009	expansion
Methylphenidate	Isocarboxazid	Yes-hypertension	Physicians 2009	expansion
Methylphenidate	Lisdexamfetamine	Yes-behavior disturbance and thought disorder	Physicians 2009	expansion
Methylphenidate	MAOI	Yes-hypertension	Physicians 2009	original
Methylphenidate	Methamphetamine	Yes-behavior disturbance and thought disorder	Physicians 2009	expansion
Methylphenidate	Phenelzine	Yes-hypertension	Physicians 2009	expansion
Methylphenidate	Rasagiline	Yes-hypertension	Physicians 2009	expansion
Methylphenidate	Selegiline	Yes-hypertension	Physicians 2009	expansion

Methylphenidate	Stimulant	Yes-behavior disturbance and thought disorder	Physicians 2009	original
Methylphenidate	Tranylcypromine	Yes-hypertension	Physicians 2009	expansion
Milnacipran	Acetaminophen	Yes-GI bleeding	Eli-Lilly 2010, Rebel 2010b, Wyeth 2011	expansion
Milnacipran	Amitriptyline	Yes-serotonin syndrome	Eli-Lilly 2010, Rebel 2010b, Wyeth 2011	expansion
Milnacipran	Amoxapine	Yes-serotonin syndrome	Eli-Lilly 2010, Rebel 2010b, Wyeth 2011	expansion
Milnacipran	Antithrombin alfa	Yes-GI bleeding	Eli-Lilly 2010, Rebel 2010b, Wyeth 2011	expansion
Milnacipran	Argatroban	Yes-GI bleeding	Eli-Lilly 2010, Rebel 2010b, Wyeth 2011	expansion
Milnacipran	Aspirin	Yes-GI bleeding	Eli-Lilly 2010, Rebel 2010b, Wyeth 2011	expansion
Milnacipran	Bivalirudin	Yes-GI bleeding	Eli-Lilly 2010, Rebel 2010b, Wyeth 2011	expansion
Milnacipran	Bromfenac	Yes-GI bleeding	Eli-Lilly 2010, Rebel 2010b, Wyeth 2011	expansion
Milnacipran	Buspirone	Yes-serotonin syndrome	Eli-Lilly 2010, Rebel 2010b, Wyeth 2011	expansion
Milnacipran	Celecoxib	Yes-GI bleeding	Eli-Lilly 2010, Rebel 2010b, Wyeth 2011	expansion
Milnacipran	Citalopram	Yes-serotonin syndrome	Eli-Lilly 2010, Rebel 2010b, Wyeth 2011	expansion
Milnacipran	Clomipramine	Yes-serotonin syndrome	Eli-Lilly 2010, Rebel 2010b, Wyeth 2011	expansion
Milnacipran	Desipramine	Yes-serotonin syndrome	Eli-Lilly 2010, Rebel 2010b, Wyeth 2011	expansion
Milnacipran	Desirudin	Yes-GI bleeding	Eli-Lilly 2010, Rebel 2010b, Wyeth 2011	expansion
Milnacipran	Desvenlafaxine	Yes-serotonin syndrome	Eli-Lilly 2010, Rebel 2010b, Wyeth 2011	expansion
Milnacipran	Dextromethorphan	Yes-serotonin syndrome	Eli-Lilly 2010, Rebel 2010b, Wyeth 2011	expansion
Milnacipran	Diclofenac	Yes-GI bleeding	Eli-Lilly 2010, Rebel 2010b, Wyeth 2011	expansion
Milnacipran	Diflunisal	Yes-GI bleeding	Eli-Lilly 2010, Rebel 2010b, Wyeth 2011	expansion
Milnacipran	Doxepin	Yes-serotonin syndrome	Eli-Lilly 2010, Rebel 2010b, Wyeth 2011	expansion

Milnacipran	Duloxetine	Yes-serotonin syndrome	Eli-Lilly 2010, Rebel 2010b, Wyeth 2011	expansion
Milnacipran	Escitalopram	Yes-serotonin syndrome	Eli-Lilly 2010, Rebel 2010b, Wyeth 2011	expansion
Milnacipran	Etodolac	Yes-GI bleeding	Eli-Lilly 2010, Rebel 2010b, Wyeth 2011	expansion
Milnacipran	Fenoprofen	Yes-GI bleeding	Eli-Lilly 2010, Rebel 2010b, Wyeth 2011	expansion
Milnacipran	Fentanyl	Yes-serotonin syndrome	Eli-Lilly 2010, Rebel 2010b, Wyeth 2011	expansion
Milnacipran	Fluoxetine	Yes-serotonin syndrome	Eli-Lilly 2010, Rebel 2010b, Wyeth 2011	expansion
Milnacipran	Flurbiprofen	Yes-GI bleeding	Eli-Lilly 2010, Rebel 2010b, Wyeth 2011	expansion
Milnacipran	Fluvoxamine	Yes-serotonin syndrome	Eli-Lilly 2010, Rebel 2010b, Wyeth 2011	expansion
Milnacipran	Heparin	Yes-GI bleeding	Eli-Lilly 2010, Rebel 2010b, Wyeth 2011	expansion
Milnacipran	Ibuprofen	Yes-GI bleeding	Eli-Lilly 2010, Rebel 2010b, Wyeth 2011	expansion
Milnacipran	Imipramine	Yes-serotonin syndrome	Eli-Lilly 2010, Rebel 2010b, Wyeth 2011	expansion
Milnacipran	Indomethacin	Yes-GI bleeding	Eli-Lilly 2010, Rebel 2010b, Wyeth 2011	expansion
Milnacipran	Isocarboxazid	Yes-serotonin syndrome	Eli-Lilly 2010, Rebel 2010b, Wyeth 2011	expansion
Milnacipran	Ketoprofen	Yes-GI bleeding	Eli-Lilly 2010, Rebel 2010b, Wyeth 2011	expansion
Milnacipran	Ketorolac tromethamine	Yes-GI bleeding	Eli-Lilly 2010, Rebel 2010b, Wyeth 2011	expansion
Milnacipran	Ketorolac	Yes-GI bleeding	Eli-Lilly 2010, Rebel 2010b, Wyeth 2011	expansion
Milnacipran	Lepirudin	Yes-GI bleeding	Eli-Lilly 2010, Rebel 2010b, Wyeth 2011	expansion
Milnacipran	Linezolid	Yes-serotonin syndrome	Eli-Lilly 2010, Rebel 2010b, Wyeth 2011	expansion
Milnacipran	Mannitol	Yes-GI bleeding	Eli-Lilly 2010, Rebel 2010b, Wyeth 2011	expansion

Milnacipran	Maprotiline	Yes-serotonin syndrome	Eli-Lilly 2010, Rebel 2010b, Wyeth 2011	expansion
Milnacipran	Mefenamic acid	Yes-GI bleeding	Eli-Lilly 2010, Rebel 2010b, Wyeth 2011	expansion
Milnacipran	Meloxicam	Yes-GI bleeding	Eli-Lilly 2010, Rebel 2010b, Wyeth 2011	expansion
Milnacipran	Meperidine	Yes-serotonin syndrome	Eli-Lilly 2010, Rebel 2010b, Wyeth 2011	expansion
Milnacipran	Milnacipran	Yes-serotonin syndrome	Eli-Lilly 2010, Rebel 2010b, Wyeth 2011	expansion
Milnacipran	NSAID	Yes-GI bleeding	Eli-Lilly 2010, Rebel 2010b, Wyeth 2011	expansion
Milnacipran	Nabumetone	Yes-GI bleeding	Eli-Lilly 2010, Rebel 2010b, Wyeth 2011	expansion
Milnacipran	Naproxen	Yes-GI bleeding	Eli-Lilly 2010, Rebel 2010b, Wyeth 2011	expansion
Milnacipran	Nefazodone	Yes-serotonin syndrome	Eli-Lilly 2010, Rebel 2010b, Wyeth 2011	expansion
Milnacipran	Nepafenac	Yes-GI bleeding	Eli-Lilly 2010, Rebel 2010b, Wyeth 2011	expansion
Milnacipran	Nortriptyline	Yes-serotonin syndrome	Eli-Lilly 2010, Rebel 2010b, Wyeth 2011	expansion
Milnacipran	Odansetran	Yes-serotonin syndrome	Eli-Lilly 2010, Rebel 2010b, Wyeth 2011	expansion
Milnacipran	Oxaprozin	Yes-GI bleeding	Eli-Lilly 2010, Rebel 2010b, Wyeth 2011	expansion
Milnacipran	Paroxetine	Yes-serotonin syndrome	Eli-Lilly 2010, Rebel 2010b, Wyeth 2011	expansion
Milnacipran	Pentazocine	Yes-serotonin syndrome	Eli-Lilly 2010, Rebel 2010b, Wyeth 2011	expansion
Milnacipran	Phenelzine	Yes-serotonin syndrome	Eli-Lilly 2010, Rebel 2010b, Wyeth 2011	expansion
Milnacipran	Piroxicam	Yes-GI bleeding	Eli-Lilly 2010, Rebel 2010b, Wyeth 2011	expansion
Milnacipran	Protriptyline	Yes-serotonin syndrome	Eli-Lilly 2010, Rebel 2010b, Wyeth 2011	expansion
Milnacipran	Rasagiline	Yes-serotonin syndrome	Eli-Lilly 2010, Rebel 2010b, Wyeth 2011	expansion

Milnacipran	SEROTONIN-1B AND SERC	Yes-serotonin syndrome	Eli-Lilly 2010, Rebel 2010b, Wyeth 2011	expansion
Milnacipran	Salicylamide	Yes-GI bleeding	Eli-Lilly 2010, Rebel 2010b, Wyeth 2011	expansion
Milnacipran	Selegiline	Yes-serotonin syndrome	Eli-Lilly 2010, Rebel 2010b, Wyeth 2011	expansion
Milnacipran	Sertraline	Yes-serotonin syndrome	Eli-Lilly 2010, Rebel 2010b, Wyeth 2011	expansion
Milnacipran	St Johns wort	Yes-serotonin syndrome	Eli-Lilly 2010, Rebel 2010b, Wyeth 2011	expansion
Milnacipran	Sulindac	Yes-GI bleeding	Eli-Lilly 2010, Rebel 2010b, Wyeth 2011	expansion
Milnacipran	Sumatriptan	Yes-serotonin syndrome	Eli-Lilly 2010, Rebel 2010b, Wyeth 2011	expansion
Milnacipran	Sympathomimetic	Yes-serotonin syndrome	Eli-Lilly 2010, Rebel 2010b, Wyeth 2011	expansion
Milnacipran	Tolmetin	Yes-GI bleeding	Eli-Lilly 2010, Rebel 2010b, Wyeth 2011	expansion
Milnacipran	Tramadol	Yes-serotonin syndrome	Eli-Lilly 2010, Rebel 2010b, Wyeth 2011	expansion
Milnacipran	Tranylcypromine	Yes-serotonin syndrome	Eli-Lilly 2010, Rebel 2010b, Wyeth 2011	expansion
Milnacipran	Trazodone	Yes-serotonin syndrome	Eli-Lilly 2010, Rebel 2010b, Wyeth 2011	expansion
Milnacipran	Trimipramine	Yes-serotonin syndrome	Eli-Lilly 2010, Rebel 2010b, Wyeth 2011	expansion
Milnacipran	Tryptophan	Yes-serotonin syndrome	Eli-Lilly 2010, Rebel 2010b, Wyeth 2011	expansion
Milnacipran	VITAMIN K ANTAGONIST	Yes-GI bleeding	Eli-Lilly 2010, Rebel 2010b, Wyeth 2011	expansion
Milnacipran	Venlafaxine	Yes-serotonin syndrome	Eli-Lilly 2010, Rebel 2010b, Wyeth 2011	expansion
Milnacipran	Vilazodone	Yes-serotonin syndrome	Eli-Lilly 2010, Rebel 2010b, Wyeth 2011	expansion
Milnacipran	Warfarin	Yes-GI bleeding	Eli-Lilly 2010, Rebel 2010b, Wyeth 2011	expansion
Mirtazapine	Amitriptyline	No	Organon 2010	original
Mirtazapine	Amitriptyline	Yes-serotonin syndrome	Organon 2010, Rebel 2010, Labopharm 2010	expansion

Mirtazapine	Amoxapine	Yes-serotonin syndrome	Organon 2010, Rebel 2010, Labopharm 2010	expansion
Mirtazapine	Antipsychotic	No	Organon 2010	expansion
Mirtazapine	Azole Antifungal	Yes	Organon 2010	expansion
Mirtazapine	Benzodiazepine	???	???	expansion
Mirtazapine	Benzodiazepine	Yes-additive impairment of motor skills	Organon 2010	expansion
Mirtazapine	Buspirone	Yes-serotonin syndrome	Organon 2010, Rebel 2010, Labopharm 2010	original
Mirtazapine	Cimetidine	Yes	Organon 2010	original
Mirtazapine	Citalopram	Yes-serotonin syndrome	Organon 2010, Rebel 2010, Labopharm 2010	expansion
Mirtazapine	Clomipramine	Yes-serotonin syndrome	Organon 2010, Rebel 2010, Labopharm 2010	expansion
Mirtazapine	Desipramine	Yes-serotonin syndrome	Organon 2010, Rebel 2010, Labopharm 2010	expansion
Mirtazapine	Desvenlafaxine	Yes-serotonin syndrome	Organon 2010, Rebel 2010, Labopharm 2010	expansion
Mirtazapine	Dextromethorphan	Yes-serotonin syndrome	Organon 2010, Rebel 2010, Labopharm 2010	original
Mirtazapine	Diazepam	???	???	original
Mirtazapine	Diazepam	Yes-additive impairment of motor skills	Organon 2010	original
Mirtazapine	Doxepin	Yes-serotonin syndrome	Organon 2010, Rebel 2010, Labopharm 2010	expansion
Mirtazapine	Duloxetine	Yes-serotonin syndrome	Organon 2010, Rebel 2010, Labopharm 2010	expansion
Mirtazapine	Escitalopram	Yes-serotonin syndrome	Organon 2010, Rebel 2010, Labopharm 2010	expansion
Mirtazapine	Fentanyl	Yes-serotonin syndrome	Organon 2010, Rebel 2010, Labopharm 2010	original
Mirtazapine	Fluoxetine	Yes-serotonin syndrome	Organon 2010, Rebel 2010, Labopharm 2010	expansion
Mirtazapine	Fluvoxamine	Yes-serotonin syndrome	Organon 2010, Rebel 2010, Labopharm 2010	expansion
Mirtazapine	H2 blockers	Yes	Organon 2010	expansion
Mirtazapine	Imipramine	Yes-serotonin syndrome	Organon 2010, Rebel 2010, Labopharm 2010	expansion
Mirtazapine	Isocarboxazid	Yes-serotonin syndrome	Organon 2010, Rebel 2010, Labopharm 2010	expansion
Mirtazapine	Ketoconazole	Yes	Organon 2010	original
Mirtazapine	Linezolid	Yes-serotonin syndrome	Organon 2010, Rebel 2010, Labopharm 2010	original
Mirtazapine	Lithium	No	Organon 2010	original

Mirtazapine	MAOI	Yes-serotonin syndrome	Organon 2010, Rebel 2010, Labopharm 2010	original
Mirtazapine	Maprotiline	Yes-serotonin syndrome	Organon 2010, Rebel 2010, Labopharm 2010	expansion
Mirtazapine	Meperidine	Yes-serotonin syndrome	Organon 2010, Rebel 2010, Labopharm 2010	original
Mirtazapine	Milnacipran	Yes-serotonin syndrome	Organon 2010, Rebel 2010, Labopharm 2010	expansion
Mirtazapine	Nefazodone	Yes-serotonin syndrome	Organon 2010, Rebel 2010, Labopharm 2010	expansion
Mirtazapine	Nortriptyline	Yes-serotonin syndrome	Organon 2010, Rebel 2010, Labopharm 2010	expansion
Mirtazapine	Odansetran	Yes-serotonin syndrome	Organon 2010, Rebel 2010, Labopharm 2010	original
Mirtazapine	Paroxetine	No	Organon 2010	original
Mirtazapine	Paroxetine	Yes-serotonin syndrome	Organon 2010, Rebel 2010, Labopharm 2010	expansion
Mirtazapine	Pentazocine	Yes-serotonin syndrome	Organon 2010, Rebel 2010, Labopharm 2010	original
Mirtazapine	Phenelzine	Yes-serotonin syndrome	Organon 2010, Rebel 2010, Labopharm 2010	expansion
Mirtazapine	Protriptyline	Yes-serotonin syndrome	Organon 2010, Rebel 2010, Labopharm 2010	expansion
Mirtazapine	Rasagiline	Yes-serotonin syndrome	Organon 2010, Rebel 2010, Labopharm 2010	expansion
Mirtazapine	SEROTONIN-1B AND SERC	Yes-serotonin syndrome	Organon 2010, Rebel 2010, Labopharm 2010	expansion
Mirtazapine	SNRI	Yes-serotonin syndrome	Organon 2010, Rebel 2010, Labopharm 2010	original
Mirtazapine	SSRI	No	Organon 2010	expansion
Mirtazapine	SSRI	Yes-serotonin syndrome	Organon 2010, Rebel 2010, Labopharm 2010	original
Mirtazapine	Selegiline	Yes-serotonin syndrome	Organon 2010, Rebel 2010, Labopharm 2010	expansion
Mirtazapine	Sertraline	Yes-serotonin syndrome	Organon 2010, Rebel 2010, Labopharm 2010	expansion
Mirtazapine	St Johns wort	Yes-serotonin syndrome	Organon 2010, Rebel 2010, Labopharm 2010	original
Mirtazapine	Sumatriptan	Yes-serotonin syndrome	Organon 2010, Rebel 2010, Labopharm 2010	original

Mirtazapine	Sympathomimetic	Yes-serotonin syndrome	Organon 2010, Rebel 2010, Labopharm 2010	expansion
Mirtazapine	TCA	No	Organon 2010	expansion
Mirtazapine	TCA	Yes-serotonin syndrome	Organon 2010, Rebel 2010, Labopharm 2010	original
Mirtazapine	Tramadol	Yes-serotonin syndrome	Organon 2010, Rebel 2010, Labopharm 2010	original
Mirtazapine	Tranylcypromine	Yes-serotonin syndrome	Organon 2010, Rebel 2010, Labopharm 2010	expansion
Mirtazapine	Trazodone	Yes-serotonin syndrome	Organon 2010, Rebel 2010, Labopharm 2010	expansion
Mirtazapine	Trimipramine	Yes-serotonin syndrome	Organon 2010, Rebel 2010, Labopharm 2010	expansion
Mirtazapine	Tryptophan	Yes-serotonin syndrome	Organon 2010, Rebel 2010, Labopharm 2010	original
Mirtazapine	Venlafaxine	Yes-serotonin syndrome	Organon 2010, Rebel 2010, Labopharm 2010	expansion
Mirtazapine	Vilazodone	Yes-serotonin syndrome	Organon 2010, Rebel 2010, Labopharm 2010	expansion
Nefazodone	Acetaminophen	Yes-bleeding	Forest 2009, Forest 2011, Dista 2009, Alphapharm 2007, Watson 2008, Forest 2011B	expansion
Nefazodone	Alprazolam	???	???	original
Nefazodone	Aminoketone	Yes-bleeding	Forest 2009, Forest 2011, Dista 2009, Alphapharm 2007, Watson 2008, Forest 2011B	expansion
Nefazodone	Amitriptyline	Yes-serotonin syndrome	Forest 2009, Forest 2011, Dista 2009, Alphapharm 2007, Watson 2008, Forest 2011B (Vilazodone)	expansion
Nefazodone	Amitriptyline	Yes-serotonin syndrome	Organon 2010, Rebel 2010, Labopharm 2010	expansion

Nefazodone	Amoxapine	Yes-serotonin syndrome	Forest 2009, Forest 2011, Dista 2009, Alphapharm 2007, Watson 2008, Forest 2011B (Vilazodone)	expansion
Nefazodone	Amoxapine	Yes-serotonin syndrome	Organon 2010, Rebel 2010, Labopharm 2010	expansion
Nefazodone	Antihistamine	Yes-QTc interval prolongation	Rebel 2010	expansion
Nefazodone	Antipsychotic	No	Rebel 2010	expansion
Nefazodone	Antipsychotic	Yes-QTc interval prolongation	Forest 2009, Forest 2011, Dista 2009, Alphapharm 2007, Watson 2008	expansion
Nefazodone	Antipsychotic	Yes-QTc interval prolongation	Rebel 2010	expansion
Nefazodone	Antithrombin alfa	Yes-bleeding	Forest 2009, Forest 2011, Dista 2009, Alphapharm 2007, Watson 2008, Forest 2011B	expansion
Nefazodone	Argatroban	Yes-bleeding	Forest 2009, Forest 2011, Dista 2009, Alphapharm 2007, Watson 2008, Forest 2011B	expansion
Nefazodone	Aspirin	Yes-bleeding	Forest 2009, Forest 2011, Dista 2009, Alphapharm 2007, Watson 2008, Forest 2011B	expansion
Nefazodone	Astemizole	Yes-QTc interval prolongation	Rebel 2010	original
Nefazodone	Atorvastatin	Yes-Rhabdomyolyses	Rebel 2010	expansion
Nefazodone	Benzodiazepine	???	???	expansion
Nefazodone	Benzodiazepine	No	Rebel 2010	expansion
Nefazodone	Beta-adrenergic blocker	Yes	Rebel 2010	expansion
Nefazodone	Bivalirudin	Yes-bleeding	Forest 2009, Forest 2011, Dista 2009, Alphapharm 2007, Watson 2008, Forest 2011B	expansion

Nefazodone	Bromfenac	Yes-bleeding	Forest 2009, Forest 2011, Dista 2009, Alphapharm 2007, Watson 2008, Forest 2011B	expansion
Nefazodone	Bupropion	Yes-bleeding	Forest 2009, Forest 2011, Dista 2009, Alphapharm 2007, Watson 2008, Forest 2011B	expansion
Nefazodone	Buspirone	Yes	Rebel 2010	original
Nefazodone	Buspirone	Yes-lightheadedness, asthenia, dizziness, and somr	Rebel 2010	original
Nefazodone	Buspirone	Yes-serotonin syndrome	Organon 2010, Rebel 2010, Labopharm 2010	original
Nefazodone	Cardiac Glycoside	No	Rebel 2010	expansion
Nefazodone	Celecoxib	Yes-bleeding	Forest 2009, Forest 2011, Dista 2009, Alphapharm 2007, Watson 2008, Forest 2011B	expansion
Nefazodone	Cimetidine	No	Rebel 2010	original
Nefazodone	Cisapride	Yes-QTc interval prolongation	Rebel 2010	original
Nefazodone	Citalopram	Yes-serotonin syndrome	Forest 2009, Forest 2011, Dista 2009, Alphapharm 2007, Watson 2008, Forest 2011B (Vilazodone)	expansion
Nefazodone	Citalopram	Yes-serotonin syndrome	Organon 2010, Rebel 2010, Labopharm 2010	expansion
Nefazodone	Clomipramine	Yes-serotonin syndrome	Forest 2009, Forest 2011, Dista 2009, Alphapharm 2007, Watson 2008, Forest 2011B (Vilazodone)	expansion
Nefazodone	Clomipramine	Yes-serotonin syndrome	Organon 2010, Rebel 2010, Labopharm 2010	expansion
Nefazodone	Desipramine	Yes	Rebel 2010	original

Nefazodone	Desipramine	Yes-serotonin syndrome	Forest 2009, Forest 2011, Dista 2009, Alphapharm 2007, Watson 2008, Forest 2011B (Vilazodone)	expansion
Nefazodone	Desipramine	Yes-serotonin syndrome	Organon 2010, Rebel 2010, Labopharm 2010	expansion
Nefazodone	Desirudin	Yes-bleeding	Forest 2009, Forest 2011, Dista 2009, Alphapharm 2007, Watson 2008, Forest 2011B	expansion
Nefazodone	Desvenlafaxine	Yes-serotonin syndrome	Forest 2009, Forest 2011, Dista 2009, Alphapharm 2007, Watson 2008, Forest 2011B (Vilazodone)	expansion
Nefazodone	Desvenlafaxine	Yes-serotonin syndrome	Organon 2010, Rebel 2010, Labopharm 2010	expansion
Nefazodone	Dextromethorphan	Yes-serotonin syndrome	Organon 2010, Rebel 2010, Labopharm 2010	original
Nefazodone	Diclofenac	Yes-bleeding	Forest 2009, Forest 2011, Dista 2009, Alphapharm 2007, Watson 2008, Forest 2011B	expansion
Nefazodone	Diflunisal	Yes-bleeding	Forest 2009, Forest 2011, Dista 2009, Alphapharm 2007, Watson 2008, Forest 2011B	expansion
Nefazodone	Digoxin	No	Rebel 2010	original
Nefazodone	Doxepin	Yes-serotonin syndrome	Forest 2009, Forest 2011, Dista 2009, Alphapharm 2007, Watson 2008, Forest 2011B (Vilazodone)	expansion
Nefazodone	Doxepin	Yes-serotonin syndrome	Organon 2010, Rebel 2010, Labopharm 2010	expansion

Nefazodone	Duloxetine	Yes-serotonin syndrome	Forest 2009, Forest 2011, Dista 2009, Alphapharm 2007, Watson 2008, Forest 2011B (Vilazodone)	expansion
Nefazodone	Duloxetine	Yes-serotonin syndrome	Organon 2010, Rebel 2010, Labopharm 2010	expansion
Nefazodone	Escitalopram	Yes-serotonin syndrome	Forest 2009, Forest 2011, Dista 2009, Alphapharm 2007, Watson 2008, Forest 2011B (Vilazodone)	expansion
Nefazodone	Escitalopram	Yes-serotonin syndrome	Organon 2010, Rebel 2010, Labopharm 2010	expansion
Nefazodone	Etodolac	Yes-bleeding	Forest 2009, Forest 2011, Dista 2009, Alphapharm 2007, Watson 2008, Forest 2011B	expansion
Nefazodone	Ezetimibe	Yes-Rhabdomyolyses	Rebel 2010	expansion
Nefazodone	Fenoprofen	Yes-bleeding	Forest 2009, Forest 2011, Dista 2009, Alphapharm 2007, Watson 2008, Forest 2011B	expansion
Nefazodone	Fentanyl	Yes-serotonin syndrome	Organon 2010, Rebel 2010, Labopharm 2010	original
Nefazodone	Fluoxetine	Yes	Rebel 2010	original
Nefazodone	Fluoxetine	Yes-headache, lightheadedness, nausea, or paresth	Rebel 2010	original
Nefazodone	Flurbiprofen	Yes-bleeding	Forest 2009, Forest 2011, Dista 2009, Alphapharm 2007, Watson 2008, Forest 2011B	expansion
Nefazodone	Fluvastatin	Yes-Rhabdomyolyses	Rebel 2010	expansion
Nefazodone	Fluvoxamine	Yes-serotonin syndrome	Forest 2009, Forest 2011, Dista 2009, Alphapharm 2007, Watson 2008, Forest 2011B (Vilazodone)	expansion

Nefazodone	Fluvoxamine	Yes-serotonin syndrome	Organon 2010, Rebel 2010, Labopharm 2010	expansion
Nefazodone	H2 blockers	No	Rebel 2010	expansion
Nefazodone	HMG-CoA Reductase Inhil	Yes-Rhabdomyolyses	Rebel 2010	original
Nefazodone	Haloperidol	No	Rebel 2010	original
Nefazodone	Heparin	Yes-bleeding	Forest 2009, Forest 2011, Dista 2009, Alphapharm 2007, Watson 2008, Forest 2011B	expansion
Nefazodone	Ibuprofen	Yes-bleeding	Forest 2009, Forest 2011, Dista 2009, Alphapharm 2007, Watson 2008, Forest 2011B	expansion
Nefazodone	Imipramine	Yes-serotonin syndrome	Forest 2009, Forest 2011, Dista 2009, Alphapharm 2007, Watson 2008, Forest 2011B (Vilazodone)	expansion
Nefazodone	Imipramine	Yes-serotonin syndrome	Organon 2010, Rebel 2010, Labopharm 2010	expansion
Nefazodone	Indomethacin	Yes-bleeding	Forest 2009, Forest 2011, Dista 2009, Alphapharm 2007, Watson 2008, Forest 2011B	expansion
Nefazodone	Isocarboxazid	Yes-serotonin syndrome	Forest 2009, Forest 2011, Dista 2009, Alphapharm 2007, Watson 2008, Forest 2011B (Vilazodone)	expansion
Nefazodone	Isocarboxazid	Yes-serotonin syndrome	Organon 2010, Rebel 2010, Labopharm 2010	expansion
Nefazodone	Ketoprofen	Yes-bleeding	Forest 2009, Forest 2011, Dista 2009, Alphapharm 2007, Watson 2008, Forest 2011B	expansion

Nefazodone	Ketorolac tromethamine	Yes-bleeding	Forest 2009, Forest 2011, Dista 2009, Alphapharm 2007, Watson 2008, Forest 2011B	expansion
Nefazodone	Ketorolac	Yes-bleeding	Forest 2009, Forest 2011, Dista 2009, Alphapharm 2007, Watson 2008, Forest 2011B	expansion
Nefazodone	Lepirudin	Yes-bleeding	Forest 2009, Forest 2011, Dista 2009, Alphapharm 2007, Watson 2008, Forest 2011B	expansion
Nefazodone	Linezolid	Yes-serotonin syndrome	Organon 2010, Rebel 2010, Labopharm 2010	original
Nefazodone	Lithium	No	Rebel 2010	original
Nefazodone	Lorazepam	No	Rebel 2010	original
Nefazodone	Lovastatin	Yes-Rhabdomyolyses	Rebel 2010	expansion
Nefazodone	MAOI	Yes-serotonin syndrome	Organon 2010, Rebel 2010, Labopharm 2010	original
Nefazodone	Mannitol	Yes-bleeding	Forest 2009, Forest 2011, Dista 2009, Alphapharm 2007, Watson 2008, Forest 2011B	expansion
Nefazodone	Maprotiline	Yes-serotonin syndrome	Forest 2009, Forest 2011, Dista 2009, Alphapharm 2007, Watson 2008, Forest 2011B (Vilazodone)	expansion
Nefazodone	Maprotiline	Yes-serotonin syndrome	Organon 2010, Rebel 2010, Labopharm 2010	expansion
Nefazodone	Mefenamic acid	Yes-bleeding	Forest 2009, Forest 2011, Dista 2009, Alphapharm 2007, Watson 2008, Forest 2011B	expansion

Nefazodone	Meloxicam	Yes-bleeding	Forest 2009, Forest 2011, Dista 2009, Alphapharm 2007, Watson 2008, Forest 2011B	expansion
Nefazodone	Meperidine	Yes-serotonin syndrome	Organon 2010, Rebel 2010, Labopharm 2010	original
Nefazodone	Methylxanthine	No	Rebel 2010	expansion
Nefazodone	Milnacipran	Yes-serotonin syndrome	Forest 2009, Forest 2011, Dista 2009, Alphapharm 2007, Watson 2008, Forest 2011B (Vilazodone)	expansion
Nefazodone	Milnacipran	Yes-serotonin syndrome	Organon 2010, Rebel 2010, Labopharm 2010	expansion
Nefazodone	NSAID	Yes-bleeding	Forest 2009, Forest 2011, Dista 2009, Alphapharm 2007, Watson 2008, Forest 2011B	expansion
Nefazodone	Nabumetone	Yes-bleeding	Forest 2009, Forest 2011, Dista 2009, Alphapharm 2007, Watson 2008, Forest 2011B	expansion
Nefazodone	Naproxen	Yes-bleeding	Forest 2009, Forest 2011, Dista 2009, Alphapharm 2007, Watson 2008, Forest 2011B	expansion
Nefazodone	Nefazodone	Yes-serotonin syndrome	Forest 2009, Forest 2011, Dista 2009, Alphapharm 2007, Watson 2008, Forest 2011B (Vilazodone)	expansion
Nefazodone	Nefazodone	Yes-serotonin syndrome	Organon 2010, Rebel 2010, Labopharm 2010	expansion

Nefazodone	Nepafenac	Yes-bleeding	Forest 2009, Forest 2011, Dista 2009, Alphapharm 2007, Watson 2008, Forest 2011B	expansion
Nefazodone	Nortriptyline	Yes-serotonin syndrome	Forest 2009, Forest 2011, Dista 2009, Alphapharm 2007, Watson 2008, Forest 2011B (Vilazodone)	expansion
Nefazodone	Nortriptyline	Yes-serotonin syndrome	Organon 2010, Rebel 2010, Labopharm 2010	expansion
Nefazodone	Odansetran	Yes-serotonin syndrome	Organon 2010, Rebel 2010, Labopharm 2010	original
Nefazodone	Oxaprozin	Yes-bleeding	Forest 2009, Forest 2011, Dista 2009, Alphapharm 2007, Watson 2008, Forest 2011B	expansion
Nefazodone	Paroxetine	Yes-serotonin syndrome	Forest 2009, Forest 2011, Dista 2009, Alphapharm 2007, Watson 2008, Forest 2011B (Vilazodone)	expansion
Nefazodone	Paroxetine	Yes-serotonin syndrome	Organon 2010, Rebel 2010, Labopharm 2010	expansion
Nefazodone	Pentazocine	Yes-serotonin syndrome	Organon 2010, Rebel 2010, Labopharm 2010	original
Nefazodone	Phenelzine	Yes-serotonin syndrome	Forest 2009, Forest 2011, Dista 2009, Alphapharm 2007, Watson 2008, Forest 2011B (Vilazodone)	expansion
Nefazodone	Phenelzine	Yes-serotonin syndrome	Organon 2010, Rebel 2010, Labopharm 2010	expansion
Nefazodone	Pimozide	Yes-QTc interval prolongation	Rebel 2010	original

Nefazodone	Piroxicam	Yes-bleeding	Forest 2009, Forest 2011, Dista 2009, Alphapharm 2007, Watson 2008, Forest 2011B	expansion
Nefazodone	Pitavastatin	Yes-Rhabdomyolyses	Rebel 2010	expansion
Nefazodone	Pravastatin	Yes-Rhabdomyolyses	Rebel 2010	expansion
Nefazodone	Propranolol	Yes	Rebel 2010	original
Nefazodone	Protriptyline	Yes-serotonin syndrome	Forest 2009, Forest 2011, Dista 2009, Alphapharm 2007, Watson 2008, Forest 2011B (Vilazodone)	expansion
Nefazodone	Protriptyline	Yes-serotonin syndrome	Organon 2010, Rebel 2010, Labopharm 2010	expansion
Nefazodone	Rasagiline	Yes-serotonin syndrome	Forest 2009, Forest 2011, Dista 2009, Alphapharm 2007, Watson 2008, Forest 2011B (Vilazodone)	expansion
Nefazodone	Rasagiline	Yes-serotonin syndrome	Organon 2010, Rebel 2010, Labopharm 2010	expansion
Nefazodone	Rosuvastatin	Yes-Rhabdomyolyses	Rebel 2010	expansion
Nefazodone	SEROTONIN-1B AND SERC	Yes-serotonin syndrome	Forest 2009, Forest 2011, Dista 2009, Alphapharm 2007, Watson 2008, Forest 2011B (Vilazodone)	expansion
Nefazodone	SEROTONIN-1B AND SERC	Yes-serotonin syndrome	Organon 2010, Rebel 2010, Labopharm 2010	expansion
Nefazodone	SEROTONIN-1B AND SERC	Yes-weakness, hyperreflexia, and incoordination	Forest 2009, Forest 2011, Watson 2008	expansion
Nefazodone	SNRI	Yes-serotonin syndrome	Organon 2010, Rebel 2010, Labopharm 2010	original
Nefazodone	SSRI	Yes	Rebel 2010	expansion
Nefazodone	SSRI	Yes-serotonin syndrome	Organon 2010, Rebel 2010, Labopharm 2010	original

Nefazodone	Salicylamide	Yes-bleeding	Forest 2009, Forest 2011, Dista 2009, Alphapharm 2007, Watson 2008, Forest 2011B	expansion
Nefazodone	Sedative Hypnotics	???	???	expansion
Nefazodone	Selegiline	Yes-serotonin syndrome	Forest 2009, Forest 2011, Dista 2009, Alphapharm 2007, Watson 2008, Forest 2011B (Vilazodone)	expansion
Nefazodone	Selegiline	Yes-serotonin syndrome	Organon 2010, Rebel 2010, Labopharm 2010	expansion
Nefazodone	Sertraline	Yes-serotonin syndrome	Forest 2009, Forest 2011, Dista 2009, Alphapharm 2007, Watson 2008, Forest 2011B (Vilazodone)	expansion
Nefazodone	Sertraline	Yes-serotonin syndrome	Organon 2010, Rebel 2010, Labopharm 2010	expansion
Nefazodone	Simvastatin	Yes-Rhabdomyolyses	Rebel 2010	expansion
Nefazodone	St Johns wort	Yes-serotonin syndrome	Organon 2010, Rebel 2010, Labopharm 2010	original
Nefazodone	Sulindac	Yes-bleeding	Forest 2009, Forest 2011, Dista 2009, Alphapharm 2007, Watson 2008, Forest 2011B	expansion
Nefazodone	Sumatriptan	Yes-serotonin syndrome	Organon 2010, Rebel 2010, Labopharm 2010	original
Nefazodone	Sympathomimetic	Yes-serotonin syndrome	Forest 2009, Forest 2011, Dista 2009, Alphapharm 2007, Watson 2008, Forest 2011B (Vilazodone)	expansion
Nefazodone	Sympathomimetic	Yes-serotonin syndrome	Organon 2010, Rebel 2010, Labopharm 2010	expansion
Nefazodone	TCA	Yes	Rebel 2010	expansion
Nefazodone	TCA	Yes-serotonin syndrome	Organon 2010, Rebel 2010, Labopharm 2010	original

Nefazodone	Terfenadine	Yes-QTc interval prolongation	Rebel 2010	original
Nefazodone	Theophylline	No	Rebel 2010	original
Nefazodone	Tolmetin	Yes-bleeding	Forest 2009, Forest 2011, Dista 2009, Alphapharm 2007, Watson 2008, Forest 2011B	expansion
Nefazodone	Tramadol	Yes-serotonin syndrome	Organon 2010, Rebel 2010, Labopharm 2010	original
Nefazodone	Tranylcypromine	Yes-serotonin syndrome	Forest 2009, Forest 2011, Dista 2009, Alphapharm 2007, Watson 2008, Forest 2011B (Vilazodone)	expansion
Nefazodone	Tranylcypromine	Yes-serotonin syndrome	Organon 2010, Rebel 2010, Labopharm 2010	expansion
Nefazodone	Trazodone	Yes-serotonin syndrome	Forest 2009, Forest 2011, Dista 2009, Alphapharm 2007, Watson 2008, Forest 2011B (Vilazodone)	expansion
Nefazodone	Trazodone	Yes-serotonin syndrome	Organon 2010, Rebel 2010, Labopharm 2010	expansion
Nefazodone	Triazolam	???	???	original
Nefazodone	Trimipramine	Yes-serotonin syndrome	Forest 2009, Forest 2011, Dista 2009, Alphapharm 2007, Watson 2008, Forest 2011B (Vilazodone)	expansion
Nefazodone	Trimipramine	Yes-serotonin syndrome	Organon 2010, Rebel 2010, Labopharm 2010	expansion
Nefazodone	Tryptophan	Yes-serotonin syndrome	Organon 2010, Rebel 2010, Labopharm 2010	original
Nefazodone	VITAMIN K ANTAGONIST	Yes-bleeding	Forest 2009, Forest 2011, Dista 2009, Alphapharm 2007, Watson 2008, Forest 2011B	expansion

Nefazodone	Venlafaxine	Yes-serotonin syndrome	Forest 2009, Forest 2011, Dista 2009, Alphapharm 2007, Watson 2008, Forest 2011B (Vilazodone)	expansion
Nefazodone	Venlafaxine	Yes-serotonin syndrome	Organon 2010, Rebel 2010, Labopharm 2010	expansion
Nefazodone	Vilazodone	Yes-serotonin syndrome	Forest 2009, Forest 2011, Dista 2009, Alphapharm 2007, Watson 2008, Forest 2011B (Vilazodone)	expansion
Nefazodone	Vilazodone	Yes-serotonin syndrome	Organon 2010, Rebel 2010, Labopharm 2010	expansion
Nefazodone	Warfarin	Yes-bleeding	Forest 2009, Forest 2011, Dista 2009, Alphapharm 2007, Watson 2008, Forest 2011B	expansion
Nortriptyline	Amantadine	Yes-Additive side-effects	Mylan 2007	expansion
Nortriptyline	Antiarrhythmic	Yes	Mylan 2007	expansion
Nortriptyline	Anticholinergic	Yes-Additive side-effects	Mylan 2007	original
Nortriptyline	Atropine	Yes-Additive side-effects	Mylan 2007	expansion
Nortriptyline	Benztropine	Yes-Additive side-effects	Mylan 2007	expansion
Nortriptyline	Biperiden	Yes-Additive side-effects	Mylan 2007	expansion
Nortriptyline	Chlorpropamide	Yes-hyperglycemia	Mylan 2007	original
Nortriptyline	Dicyclomine	Yes-Additive side-effects	Mylan 2007	expansion
Nortriptyline	Difenoxin	Yes-Additive side-effects	Mylan 2007	expansion
Nortriptyline	Diphenoxylate	Yes-Additive side-effects	Mylan 2007	expansion
Nortriptyline	Glycopyrronium	Yes-Additive side-effects	Mylan 2007	expansion
Nortriptyline	Hyoscyamine	Yes-Additive side-effects	Mylan 2007	expansion
Nortriptyline	Ipratropium cation	Yes-Additive side-effects	Mylan 2007	expansion
Nortriptyline	Ipratropium	Yes-Additive side-effects	Mylan 2007	expansion
Nortriptyline	Isocarboxazid	Yes-Hyperpyretic crises, severe convulsions, death	Mylan 2009, Sandoz 2010, Watson 2007 (doxepin), Mylan 2007, Sandoz 2010b, Watson 2009, Duramed 2007, Ciba-Gigy 1996	expansion
Nortriptyline	Mepenzolate	Yes-Additive side-effects	Mylan 2007	expansion
Nortriptyline	Methscopolamine	Yes-Additive side-effects	Mylan 2007	expansion
Nortriptyline	Modafinil	Yes-Additive side-effects	Mylan 2007	expansion

Nortriptyline	Phenelzine	Yes-Hyperpyretic crises, severe convulsions, death	Mylan 2009, Sandoz 2010, Watson 2007 (doxepin), Mylan 2007, Sandoz 2010b, Watson 2009, Duramed 2007, Ciba-Gigy 1996	expansion
Nortriptyline	Phenobarbital	Yes-Additive side-effects	Mylan 2007	expansion
Nortriptyline	Phenylpropanolamine	Yes-Additive side-effects	Mylan 2007	expansion
Nortriptyline	Procyclidine	Yes-Additive side-effects	Mylan 2007	expansion
Nortriptyline	Propantheline	Yes-Additive side-effects	Mylan 2007	expansion
Nortriptyline	Pseudoephedrine	Yes-Additive side-effects	Mylan 2007	expansion
Nortriptyline	Quinidine	Yes	Mylan 2007	original
Nortriptyline	Rasagiline	Yes-Hyperpyretic crises, severe convulsions, death	Mylan 2009, Sandoz 2010, Watson 2007 (doxepin), Mylan 2007, Sandoz 2010b, Watson 2009, Duramed 2007, Ciba-Gigy 1996	expansion
Nortriptyline	Reserpine	?	Mylan 2007	original
Nortriptyline	Scopolamine	Yes-Additive side-effects	Mylan 2007	expansion
Nortriptyline	Selegiline	Yes-Hyperpyretic crises, severe convulsions, death	Mylan 2009, Sandoz 2010, Watson 2007 (doxepin), Mylan 2007, Sandoz 2010b, Watson 2009, Duramed 2007, Ciba-Gigy 1996	expansion
Nortriptyline	Sulfonylurea	Yes-hyperglycemia	Mylan 2007	expansion
Nortriptyline	Sympathomimetic	Yes-Additive side-effects	Mylan 2007	original
Nortriptyline	Tiotropium	Yes-Additive side-effects	Mylan 2007	expansion
Nortriptyline	Tranylcypromine	Yes-Hyperpyretic crises, severe convulsions, death	Mylan 2009, Sandoz 2010, Watson 2007 (doxepin), Mylan 2007, Sandoz 2010b, Watson 2009, Duramed 2007, Ciba-Gigy 1996	expansion
Nortriptyline	Tropicamide	Yes-Additive side-effects	Mylan 2007	expansion
Nortriptyline	Tryptophan	Yes-Additive side-effects	Mylan 2007	expansion
Paroxetine	Acetaminophen	Yes-bleeding	Forest 2009, Forest 2011, Dista 2009, Alphapharm 2007, Watson 2008, Forest 2011B	expansion

Paroxetine	Aminoketone	Yes-bleeding	Forest 2009, Forest 2011, Dista 2009, Alphapharm 2007, Watson 2008, Forest 2011B	expansion
Paroxetine	Amitriptyline	Yes-serotonin syndrome	Forest 2009, Forest 2011, Dista 2009, Alphapharm 2007, Watson 2008, Forest 2011B (Vilazodone)	expansion
Paroxetine	Amoxapine	Yes-serotonin syndrome	Forest 2009, Forest 2011, Dista 2009, Alphapharm 2007, Watson 2008, Forest 2011B (Vilazodone)	expansion
Paroxetine	Anticholinergic	Yes-anticholinergic effects	Alphapharm 2007	expansion
Paroxetine	Antipsychotic	No	Alpharpharm 2007	expansion
Paroxetine	Antipsychotic	Yes-QTc interval prolongation	Dista 2009	expansion
Paroxetine	Antipsychotic	Yes-QTc interval prolongation	Forest 2009, Forest 2011, Dista 2009, Alphapharm 2007, Watson 2008	expansion
Paroxetine	Antithrombin alfa	Yes-bleeding	Forest 2009, Forest 2011, Dista 2009, Alphapharm 2007, Watson 2008, Forest 2011B	expansion
Paroxetine	Argatroban	Yes-bleeding	Forest 2009, Forest 2011, Dista 2009, Alphapharm 2007, Watson 2008, Forest 2011B	expansion
Paroxetine	Aspirin	Yes-bleeding	Forest 2009, Forest 2011, Dista 2009, Alphapharm 2007, Watson 2008, Forest 2011B	expansion
Paroxetine	Benzodiazepine	No	Alpharpharm 2007	expansion

Paroxetine	Bivalirudin	Yes-bleeding	Forest 2009, Forest 2011, Dista 2009, Alphapharm 2007, Watson 2008, Forest 2011B	expansion
Paroxetine	Bromfenac	Yes-bleeding	Forest 2009, Forest 2011, Dista 2009, Alphapharm 2007, Watson 2008, Forest 2011B	expansion
Paroxetine	Bupropion	Yes-bleeding	Forest 2009, Forest 2011, Dista 2009, Alphapharm 2007, Watson 2008, Forest 2011B	expansion
Paroxetine	Buspirone	Yes-serotonin syndrome	Forest 2009, Forest 2011, Dista 2009, Alphapharm 2007, Watson 2008, Forest 2011B (Vilazodone)	expansion
Paroxetine	Cardiac Glycoside	No	Alpharpharm 2007	expansion
Paroxetine	Celecoxib	Yes-bleeding	Forest 2009, Forest 2011, Dista 2009, Alphapharm 2007, Watson 2008, Forest 2011B	expansion
Paroxetine	Cimetidine	Yes	Alpharpharm 2007	original
Paroxetine	Citalopram	Yes-serotonin syndrome	Forest 2009, Forest 2011, Dista 2009, Alphapharm 2007, Watson 2008, Forest 2011B (Vilazodone)	expansion
Paroxetine	Clomipramine	Yes-serotonin syndrome	Forest 2009, Forest 2011, Dista 2009, Alphapharm 2007, Watson 2008, Forest 2011B (Vilazodone)	expansion

Paroxetine	Desipramine	Yes-serotonin syndrome	Forest 2009, Forest 2011, Dista 2009, Alphapharm 2007, Watson 2008, Forest 2011B (Vilazodone)	expansion
Paroxetine	Desirudin	Yes-bleeding	Forest 2009, Forest 2011, Dista 2009, Alphapharm 2007, Watson 2008, Forest 2011B	expansion
Paroxetine	Desvenlafaxine	Yes-serotonin syndrome	Forest 2009, Forest 2011, Dista 2009, Alphapharm 2007, Watson 2008, Forest 2011B (Vilazodone)	expansion
Paroxetine	Dextromethorphan	Yes-serotonin syndrome	Forest 2009, Forest 2011, Dista 2009, Alphapharm 2007, Watson 2008, Forest 2011B (Vilazodone)	expansion
Paroxetine	Diazepam	No	Alpharpharm 2007	original
Paroxetine	Diclofenac	Yes-bleeding	Forest 2009, Forest 2011, Dista 2009, Alphapharm 2007, Watson 2008, Forest 2011B	expansion
Paroxetine	Diflunisal	Yes-bleeding	Forest 2009, Forest 2011, Dista 2009, Alphapharm 2007, Watson 2008, Forest 2011B	expansion
Paroxetine	Digoxin	No	Alpharpharm 2007	original
Paroxetine	Doxepin	Yes-serotonin syndrome	Forest 2009, Forest 2011, Dista 2009, Alphapharm 2007, Watson 2008, Forest 2011B (Vilazodone)	expansion

Paroxetine	Duloxetine	Yes-serotonin syndrome	Forest 2009, Forest 2011, Dista 2009, Alphapharm 2007, Watson 2008, Forest 2011B (Vilazodone)	expansion
Paroxetine	Escitalopram	Yes-serotonin syndrome	Forest 2009, Forest 2011, Dista 2009, Alphapharm 2007, Watson 2008, Forest 2011B (Vilazodone)	expansion
Paroxetine	Etodolac	Yes-bleeding	Forest 2009, Forest 2011, Dista 2009, Alphapharm 2007, Watson 2008, Forest 2011B	expansion
Paroxetine	Fenoprofen	Yes-bleeding	Forest 2009, Forest 2011, Dista 2009, Alphapharm 2007, Watson 2008, Forest 2011B	expansion
Paroxetine	Fentanyl	Yes-serotonin syndrome	Forest 2009, Forest 2011, Dista 2009, Alphapharm 2007, Watson 2008, Forest 2011B (Vilazodone)	expansion
Paroxetine	Fluoxetine	Yes-serotonin syndrome	Forest 2009, Forest 2011, Dista 2009, Alphapharm 2007, Watson 2008, Forest 2011B (Vilazodone)	expansion
Paroxetine	Flurbiprofen	Yes-bleeding	Forest 2009, Forest 2011, Dista 2009, Alphapharm 2007, Watson 2008, Forest 2011B	expansion

Paroxetine	Fluvoxamine	Yes-serotonin syndrome	Forest 2009, Forest 2011, Dista 2009, Alphapharm 2007, Watson 2008, Forest 2011B (Vilazodone)	expansion
Paroxetine	H2 blockers	Yes	Alpharpharm 2007	expansion
Paroxetine	Heparin	Yes-bleeding	Forest 2009, Forest 2011, Dista 2009, Alphapharm 2007, Watson 2008, Forest 2011B	expansion
Paroxetine	Ibuprofen	Yes-bleeding	Forest 2009, Forest 2011, Dista 2009, Alphapharm 2007, Watson 2008, Forest 2011B	expansion
Paroxetine	Imipramine	Yes-serotonin syndrome	Forest 2009, Forest 2011, Dista 2009, Alphapharm 2007, Watson 2008, Forest 2011B (Vilazodone)	expansion
Paroxetine	Indomethacin	Yes-bleeding	Forest 2009, Forest 2011, Dista 2009, Alphapharm 2007, Watson 2008, Forest 2011B	expansion
Paroxetine	Isocarboxazid	Yes-serotonin syndrome	Forest 2009, Forest 2011, Dista 2009, Alphapharm 2007, Watson 2008, Forest 2011B (Vilazodone)	expansion
Paroxetine	Ketoprofen	Yes-bleeding	Forest 2009, Forest 2011, Dista 2009, Alphapharm 2007, Watson 2008, Forest 2011B	expansion

Paroxetine	Ketorolac tromethamine	Yes-bleeding	Forest 2009, Forest 2011, Dista 2009, Alphapharm 2007, Watson 2008, Forest 2011B	expansion
Paroxetine	Ketorolac	Yes-bleeding	Forest 2009, Forest 2011, Dista 2009, Alphapharm 2007, Watson 2008, Forest 2011B	expansion
Paroxetine	Lepirudin	Yes-bleeding	Forest 2009, Forest 2011, Dista 2009, Alphapharm 2007, Watson 2008, Forest 2011B	expansion
Paroxetine	Linezolid	Yes-serotonin syndrome	Forest 2009, Forest 2011, Dista 2009, Alphapharm 2007, Watson 2008, Forest 2011B (Vilazodone)	expansion
Paroxetine	Lithium	No	Alpharpharm 2007	original
Paroxetine	Mannitol	Yes-bleeding	Forest 2009, Forest 2011, Dista 2009, Alphapharm 2007, Watson 2008, Forest 2011B	expansion
Paroxetine	Maprotiline	Yes-serotonin syndrome	Forest 2009, Forest 2011, Dista 2009, Alphapharm 2007, Watson 2008, Forest 2011B (Vilazodone)	expansion
Paroxetine	Mefenamic acid	Yes-bleeding	Forest 2009, Forest 2011, Dista 2009, Alphapharm 2007, Watson 2008, Forest 2011B	expansion

Paroxetine	Meloxicam	Yes-bleeding	Forest 2009, Forest 2011, Dista 2009, Alphapharm 2007, Watson 2008, Forest 2011B	expansion
Paroxetine	Meperidine	Yes-serotonin syndrome	Forest 2009, Forest 2011, Dista 2009, Alphapharm 2007, Watson 2008, Forest 2011B (Vilazodone)	expansion
Paroxetine	Milnacipran	Yes-serotonin syndrome	Forest 2009, Forest 2011, Dista 2009, Alphapharm 2007, Watson 2008, Forest 2011B (Vilazodone)	expansion
Paroxetine	NSAID	Yes-bleeding	Forest 2009, Forest 2011, Dista 2009, Alphapharm 2007, Watson 2008, Forest 2011B	expansion
Paroxetine	Nabumetone	Yes-bleeding	Forest 2009, Forest 2011, Dista 2009, Alphapharm 2007, Watson 2008, Forest 2011B	expansion
Paroxetine	Naproxen	Yes-bleeding	Forest 2009, Forest 2011, Dista 2009, Alphapharm 2007, Watson 2008, Forest 2011B	expansion
Paroxetine	Nefazodone	Yes-serotonin syndrome	Forest 2009, Forest 2011, Dista 2009, Alphapharm 2007, Watson 2008, Forest 2011B (Vilazodone)	expansion
Paroxetine	Nepafenac	Yes-bleeding	Forest 2009, Forest 2011, Dista 2009, Alphapharm 2007, Watson 2008, Forest 2011B	expansion

Paroxetine	Nortriptyline	Yes-serotonin syndrome	Forest 2009, Forest 2011, Dista 2009, Alphapharm 2007, Watson 2008, Forest 2011B (Vilazodone)	expansion
Paroxetine	Odansetran	Yes-serotonin syndrome	Forest 2009, Forest 2011, Dista 2009, Alphapharm 2007, Watson 2008, Forest 2011B (Vilazodone)	expansion
Paroxetine	Oxaprozin	Yes-bleeding	Forest 2009, Forest 2011, Dista 2009, Alphapharm 2007, Watson 2008, Forest 2011B	expansion
Paroxetine	Paroxetine	Yes-serotonin syndrome	Forest 2009, Forest 2011, Dista 2009, Alphapharm 2007, Watson 2008, Forest 2011B (Vilazodone)	expansion
Paroxetine	Pentazocine	Yes-serotonin syndrome	Forest 2009, Forest 2011, Dista 2009, Alphapharm 2007, Watson 2008, Forest 2011B (Vilazodone)	expansion
Paroxetine	Phenelzine	Yes-serotonin syndrome	Forest 2009, Forest 2011, Dista 2009, Alphapharm 2007, Watson 2008, Forest 2011B (Vilazodone)	expansion
Paroxetine	Pimozide	Yes-QTc interval prolongation	Forest 2009, Forest 2011, Dista 2009, Alphapharm 2007, Watson 2008	expansion

Paroxetine	Piroxicam	Yes-bleeding	Forest 2009, Forest 2011, Dista 2009, Alphapharm 2007, Watson 2008, Forest 2011B	expansion
Paroxetine	Procyclidine	Yes-anticholinergic effects	Alphapharm 2007	original
Paroxetine	Protriptyline	Yes-serotonin syndrome	Forest 2009, Forest 2011, Dista 2009, Alphapharm 2007, Watson 2008, Forest 2011B (Vilazodone)	expansion
Paroxetine	Rasagiline	Yes-serotonin syndrome	Forest 2009, Forest 2011, Dista 2009, Alphapharm 2007, Watson 2008, Forest 2011B (Vilazodone)	expansion
Paroxetine	SEROTONIN-1B AND SERC	Yes-serotonin syndrome	Forest 2009, Forest 2011, Dista 2009, Alphapharm 2007, Watson 2008, Forest 2011B (Vilazodone)	expansion
Paroxetine	SEROTONIN-1B AND SERC	Yes-weakness, hyperreflexia, and incoordination	Forest 2009, Forest 2011, Watson 2008	expansion
Paroxetine	Salicylamide	Yes-bleeding	Forest 2009, Forest 2011, Dista 2009, Alphapharm 2007, Watson 2008, Forest 2011B	expansion
Paroxetine	Selegiline	Yes-serotonin syndrome	Forest 2009, Forest 2011, Dista 2009, Alphapharm 2007, Watson 2008, Forest 2011B (Vilazodone)	expansion
Paroxetine	Sertraline	Yes-serotonin syndrome	Forest 2009, Forest 2011, Dista 2009, Alphapharm 2007, Watson 2008, Forest 2011B (Vilazodone)	expansion

Paroxetine	St Johns wort	Yes-serotonin syndrome	Forest 2009, Forest 2011, Dista 2009, Alphapharm 2007, Watson 2008, Forest 2011B (Vilazodone)	expansion
Paroxetine	Sulindac	Yes-bleeding	Forest 2009, Forest 2011, Dista 2009, Alphapharm 2007, Watson 2008, Forest 2011B	expansion
Paroxetine	Sumatriptan	Yes-serotonin syndrome	Forest 2009, Forest 2011, Dista 2009, Alphapharm 2007, Watson 2008, Forest 2011B (Vilazodone)	expansion
Paroxetine	Sumatriptan	Yes-weakness, hyperreflexia, and incoordination	Forest 2009, Forest 2011, Watson 2008	expansion
Paroxetine	Sympathomimetic	Yes-headache, nausea, sweating, dizziness	Alphapharm 2007	expansion
Paroxetine	Sympathomimetic	Yes-serotonin syndrome	Forest 2009, Forest 2011, Dista 2009, Alphapharm 2007, Watson 2008, Forest 2011B (Vilazodone)	expansion
Paroxetine	Thioridazine	Yes-QTc interval prolongation	Dista 2009	original
Paroxetine	Tolmetin	Yes-bleeding	Forest 2009, Forest 2011, Dista 2009, Alphapharm 2007, Watson 2008, Forest 2011B	expansion
Paroxetine	Tramadol	Yes-serotonin syndrome	Forest 2009, Forest 2011, Dista 2009, Alphapharm 2007, Watson 2008, Forest 2011B (Vilazodone)	expansion
Paroxetine	Tranylcypromine	Yes-serotonin syndrome	Forest 2009, Forest 2011, Dista 2009, Alphapharm 2007, Watson 2008, Forest 2011B (Vilazodone)	expansion

Paroxetine	Trazodone	Yes-serotonin syndrome	Forest 2009, Forest 2011, Dista 2009, Alphapharm 2007, Watson 2008, Forest 2011B (Vilazodone)	expansion
Paroxetine	Trimipramine	Yes-serotonin syndrome	Forest 2009, Forest 2011, Dista 2009, Alphapharm 2007, Watson 2008, Forest 2011B (Vilazodone)	expansion
Paroxetine	Tryptophan	Yes-headache, nausea, sweating, dizziness	Alphapharm 2007	original
Paroxetine	VITAMIN K ANTAGONIST	Yes-bleeding	Forest 2009, Forest 2011, Dista 2009, Alphapharm 2007, Watson 2008, Forest 2011B	expansion
Paroxetine	Venlafaxine	Yes-serotonin syndrome	Forest 2009, Forest 2011, Dista 2009, Alphapharm 2007, Watson 2008, Forest 2011B (Vilazodone)	expansion
Paroxetine	Vilazodone	Yes-serotonin syndrome	Forest 2009, Forest 2011, Dista 2009, Alphapharm 2007, Watson 2008, Forest 2011B (Vilazodone)	expansion
Paroxetine	Warfarin	Yes-bleeding	Forest 2009, Forest 2011, Dista 2009, Alphapharm 2007, Watson 2008, Forest 2011B	expansion
Phenelzine	Amitriptyline	Yes-serotonin syndrome	Dey 2010, Validus 2007, Parke-Davis 2011, GSK 2010	expansion
Phenelzine	Amoxapine	Yes-serotonin syndrome	Dey 2010, Validus 2007, Parke-Davis 2011, GSK 2010	expansion
Phenelzine	Anticholinergic	Yes-hypertension	Dey 2010, Validus 2007, Parke-Davis 2011, GSK 2010	expansion

Phenelzine	Buspirone	Yes-hypertension	Dey 2010, Validus 2007, Parke-Davis 2011, GSK 2010	expansion
Phenelzine	Citalopram	Yes-serotonin syndrome	Dey 2010, Validus 2007, Parke-Davis 2011, GSK 2010	expansion
Phenelzine	Clomipramine	Yes-serotonin syndrome	Dey 2010, Validus 2007, Parke-Davis 2011, GSK 2010	expansion
Phenelzine	Desipramine	Yes-serotonin syndrome	Dey 2010, Validus 2007, Parke-Davis 2011, GSK 2010	expansion
Phenelzine	Desvenlafaxine	Yes-serotonin syndrome	Dey 2010, Validus 2007, Parke-Davis 2011, GSK 2010	expansion
Phenelzine	Dextromethorphan	Yes-serotonin syndrome	Dey 2010, Validus 2007, Parke-Davis 2011, GSK 2010	expansion
Phenelzine	Doxepin	Yes-serotonin syndrome	Dey 2010, Validus 2007, Parke-Davis 2011, GSK 2010	expansion
Phenelzine	Duloxetine	Yes-serotonin syndrome	Dey 2010, Validus 2007, Parke-Davis 2011, GSK 2010	expansion
Phenelzine	Escitalopram	Yes-serotonin syndrome	Dey 2010, Validus 2007, Parke-Davis 2011, GSK 2010	expansion
Phenelzine	Fentanyl	Yes-serotonin syndrome	Dey 2010, Validus 2007, Parke-Davis 2011, GSK 2010	expansion
Phenelzine	Fluoxetine	Yes-serotonin syndrome	Dey 2010, Validus 2007, Parke-Davis 2011, GSK 2010	expansion
Phenelzine	Fluvoxamine	Yes-serotonin syndrome	Dey 2010, Validus 2007, Parke-Davis 2011, GSK 2010	expansion
Phenelzine	Guanethidine	Yes-hypertension	Parke-Davis 2011	original
Phenelzine	Imipramine	Yes-serotonin syndrome	Dey 2010, Validus 2007, Parke-Davis 2011, GSK 2010	expansion
Phenelzine	Isocarboxazid	Yes-serotonin syndrome	Dey 2010, Validus 2007, Parke-Davis 2011, GSK 2010	expansion

Phenelzine	Linezolid	Yes-serotonin syndrome	Dey 2010, Validus 2007, Parke-Davis 2011, GSK 2010	expansion
Phenelzine	Maprotiline	Yes-serotonin syndrome	Dey 2010, Validus 2007, Parke-Davis 2011, GSK 2010	expansion
Phenelzine	Meperidine	Yes- coma, severe hypertension or hypotension, se	Dey 2010, Validus 2007, Parke-Davis 2011, GSK 2010	expansion
Phenelzine	Milnacipran	Yes-serotonin syndrome	Dey 2010, Validus 2007, Parke-Davis 2011, GSK 2010	expansion
Phenelzine	Modafinil	Yes-hypertension	Dey 2010, Validus 2007, Parke-Davis 2011, GSK 2010	expansion
Phenelzine	Nefazodone	Yes-serotonin syndrome	Dey 2010, Validus 2007, Parke-Davis 2011, GSK 2010	expansion
Phenelzine	Nortriptyline	Yes-serotonin syndrome	Dey 2010, Validus 2007, Parke-Davis 2011, GSK 2010	expansion
Phenelzine	Odansetran	Yes-serotonin syndrome	Dey 2010, Validus 2007, Parke-Davis 2011, GSK 2010	expansion
Phenelzine	Paroxetine	Yes-serotonin syndrome	Dey 2010, Validus 2007, Parke-Davis 2011, GSK 2010	expansion
Phenelzine	Pentazocine	Yes-serotonin syndrome	Dey 2010, Validus 2007, Parke-Davis 2011, GSK 2010	expansion
Phenelzine	Phenelzine	Yes-serotonin syndrome	Dey 2010, Validus 2007, Parke-Davis 2011, GSK 2010	expansion
Phenelzine	Phenylpropanolamine	Yes-hypertension	Dey 2010, Validus 2007, Parke-Davis 2011, GSK 2010	expansion
Phenelzine	Protriptyline	Yes-serotonin syndrome	Dey 2010, Validus 2007, Parke-Davis 2011, GSK 2010	expansion
Phenelzine	Pseudoephedrine	Yes-hypertension	Dey 2010, Validus 2007, Parke-Davis 2011, GSK 2010	expansion

Phenelzine	Rasagiline	Yes-serotonin syndrome	Dey 2010, Validus 2007, Parke-Davis 2011, GSK 2010	expansion
Phenelzine	SEROTONIN-1B AND SERC	Yes-serotonin syndrome	Dey 2010, Validus 2007, Parke-Davis 2011, GSK 2010	expansion
Phenelzine	Selegiline	Yes-serotonin syndrome	Dey 2010, Validus 2007, Parke-Davis 2011, GSK 2010	expansion
Phenelzine	Sertraline	Yes-serotonin syndrome	Dey 2010, Validus 2007, Parke-Davis 2011, GSK 2010	expansion
Phenelzine	St Johns wort	Yes-serotonin syndrome	Dey 2010, Validus 2007, Parke-Davis 2011, GSK 2010	expansion
Phenelzine	Sumatriptan	Yes-serotonin syndrome	Dey 2010, Validus 2007, Parke-Davis 2011, GSK 2010	expansion
Phenelzine	Sympathomimetic	Yes-behavioral and neurological syndromes	Parke-Davis 2011	expansion
Phenelzine	Sympathomimetic	Yes-hypertension	Dey 2010, Validus 2007, Parke-Davis 2011, GSK 2010	expansion
Phenelzine	Sympathomimetic	Yes-serotonin syndrome	Dey 2010, Validus 2007, Parke-Davis 2011, GSK 2010	expansion
Phenelzine	Tramadol	Yes-serotonin syndrome	Dey 2010, Validus 2007, Parke-Davis 2011, GSK 2010	expansion
Phenelzine	Tranylcypromine	Yes-serotonin syndrome	Dey 2010, Validus 2007, Parke-Davis 2011, GSK 2010	expansion
Phenelzine	Trazodone	Yes-serotonin syndrome	Dey 2010, Validus 2007, Parke-Davis 2011, GSK 2010	expansion
Phenelzine	Trimipramine	Yes-serotonin syndrome	Dey 2010, Validus 2007, Parke-Davis 2011, GSK 2010	expansion
Phenelzine	Tryptophan	Yes-behavioral and neurological syndromes	Parke-Davis 2011	original
Phenelzine	Venlafaxine	Yes-serotonin syndrome	Dey 2010, Validus 2007, Parke-Davis 2011, GSK 2010	expansion
Phenelzine	Vilazodone	Yes-serotonin syndrome	Dey 2010, Validus 2007, Parke-Davis 2011, GSK 2010	expansion

Protriptyline	Isocarboxazid	Yes-Hyperpyretic crises, severe convulsions, death	Mylan 2009, Sandoz 2010, Watson 2007 (doxepin), Mylan 2007, Sandoz 2010b, Watson 2009, Duramed 2007, Ciba-Gigy 1996	expansion
Protriptyline	Phenelzine	Yes-Hyperpyretic crises, severe convulsions, death	Mylan 2009, Sandoz 2010, Watson 2007 (doxepin), Mylan 2007, Sandoz 2010b, Watson 2009, Duramed 2007, Ciba-Gigy 1996	expansion
Protriptyline	Rasagiline	Yes-Hyperpyretic crises, severe convulsions, death	Mylan 2009, Sandoz 2010, Watson 2007 (doxepin), Mylan 2007, Sandoz 2010b, Watson 2009, Duramed 2007, Ciba-Gigy 1996	expansion
Protriptyline	Selegiline	Yes-Hyperpyretic crises, severe convulsions, death	Mylan 2009, Sandoz 2010, Watson 2007 (doxepin), Mylan 2007, Sandoz 2010b, Watson 2009, Duramed 2007, Ciba-Gigy 1996	expansion
Protriptyline	Tranylcypromine	Yes-Hyperpyretic crises, severe convulsions, death	Mylan 2009, Sandoz 2010, Watson 2007 (doxepin), Mylan 2007, Sandoz 2010b, Watson 2009, Duramed 2007, Ciba-Gigy 1996	expansion
Rasagiline	Amitriptyline	Yes-serotonin syndrome	Dey 2010, Validus 2007, Parke-Davis 2011, GSK 2010	expansion
Rasagiline	Amoxapine	Yes-serotonin syndrome	Dey 2010, Validus 2007, Parke-Davis 2011, GSK 2010	expansion
Rasagiline	Anticholinergic	Yes-hypertension	Dey 2010, Validus 2007, Parke-Davis 2011, GSK 2010	expansion

Rasagiline	Buspirone	Yes-hypertension		Dey 2010, Validus 2007, Parke-Davis 2011, GSK 2010	expansion
Rasagiline	Citalopram	Yes-serotonin syndrome		Dey 2010, Validus 2007, Parke-Davis 2011, GSK 2010	expansion
Rasagiline	Clomipramine	Yes-serotonin syndrome		Dey 2010, Validus 2007, Parke-Davis 2011, GSK 2010	expansion
Rasagiline	Desipramine	Yes-serotonin syndrome		Dey 2010, Validus 2007, Parke-Davis 2011, GSK 2010	expansion
Rasagiline	Desvenlafaxine	Yes-serotonin syndrome		Dey 2010, Validus 2007, Parke-Davis 2011, GSK 2010	expansion
Rasagiline	Dextromethorphan	Yes-serotonin syndrome		Dey 2010, Validus 2007, Parke-Davis 2011, GSK 2010	expansion
Rasagiline	Doxepin	Yes-serotonin syndrome		Dey 2010, Validus 2007, Parke-Davis 2011, GSK 2010	expansion
Rasagiline	Duloxetine	Yes-serotonin syndrome		Dey 2010, Validus 2007, Parke-Davis 2011, GSK 2010	expansion
Rasagiline	Escitalopram	Yes-serotonin syndrome		Dey 2010, Validus 2007, Parke-Davis 2011, GSK 2010	expansion
Rasagiline	Fentanyl	Yes-serotonin syndrome		Dey 2010, Validus 2007, Parke-Davis 2011, GSK 2010	expansion
Rasagiline	Fluoxetine	Yes-serotonin syndrome		Dey 2010, Validus 2007, Parke-Davis 2011, GSK 2010	expansion
Rasagiline	Fluvoxamine	Yes-serotonin syndrome		Dey 2010, Validus 2007, Parke-Davis 2011, GSK 2010	expansion
Rasagiline	Imipramine	Yes-serotonin syndrome		Dey 2010, Validus 2007, Parke-Davis 2011, GSK 2010	expansion
Rasagiline	Isocarboxazid	Yes-serotonin syndrome		Dey 2010, Validus 2007, Parke-Davis 2011, GSK 2010	expansion

Rasagiline	Linezolid	Yes-serotonin syndrome	Dey 2010, Validus 2007, Parke-Davis 2011, GSK 2010	expansion
Rasagiline	Maprotiline	Yes-serotonin syndrome	Dey 2010, Validus 2007, Parke-Davis 2011, GSK 2010	expansion
Rasagiline	Meperidine	Yes- coma, severe hypertension or hypotension, se	Dey 2010, Validus 2007, Parke-Davis 2011, GSK 2010	expansion
Rasagiline	Milnacipran	Yes-serotonin syndrome	Dey 2010, Validus 2007, Parke-Davis 2011, GSK 2010	expansion
Rasagiline	Modafinil	Yes-hypertension	Dey 2010, Validus 2007, Parke-Davis 2011, GSK 2010	expansion
Rasagiline	Nefazodone	Yes-serotonin syndrome	Dey 2010, Validus 2007, Parke-Davis 2011, GSK 2010	expansion
Rasagiline	Nortriptyline	Yes-serotonin syndrome	Dey 2010, Validus 2007, Parke-Davis 2011, GSK 2010	expansion
Rasagiline	Odansetran	Yes-serotonin syndrome	Dey 2010, Validus 2007, Parke-Davis 2011, GSK 2010	expansion
Rasagiline	Paroxetine	Yes-serotonin syndrome	Dey 2010, Validus 2007, Parke-Davis 2011, GSK 2010	expansion
Rasagiline	Pentazocine	Yes-serotonin syndrome	Dey 2010, Validus 2007, Parke-Davis 2011, GSK 2010	expansion
Rasagiline	Phenelzine	Yes-serotonin syndrome	Dey 2010, Validus 2007, Parke-Davis 2011, GSK 2010	expansion
Rasagiline	Phenylpropanolamine	Yes-hypertension	Dey 2010, Validus 2007, Parke-Davis 2011, GSK 2010	expansion
Rasagiline	Protriptyline	Yes-serotonin syndrome	Dey 2010, Validus 2007, Parke-Davis 2011, GSK 2010	expansion
Rasagiline	Pseudoephedrine	Yes-hypertension	Dey 2010, Validus 2007, Parke-Davis 2011, GSK 2010	expansion

Rasagiline	Rasagiline	Yes-serotonin syndrome		Dey 2010, Validus 2007, Parke-Davis 2011, GSK 2010	expansion
Rasagiline	SEROTONIN-1B AND SERC	Yes-serotonin syndrome		Dey 2010, Validus 2007, Parke-Davis 2011, GSK 2010	expansion
Rasagiline	Selegiline	Yes-serotonin syndrome		Dey 2010, Validus 2007, Parke-Davis 2011, GSK 2010	expansion
Rasagiline	Sertraline	Yes-serotonin syndrome		Dey 2010, Validus 2007, Parke-Davis 2011, GSK 2010	expansion
Rasagiline	St Johns wort	Yes-serotonin syndrome		Dey 2010, Validus 2007, Parke-Davis 2011, GSK 2010	expansion
Rasagiline	Sumatriptan	Yes-serotonin syndrome		Dey 2010, Validus 2007, Parke-Davis 2011, GSK 2010	expansion
Rasagiline	Sympathomimetic	Yes-hypertension		Dey 2010, Validus 2007, Parke-Davis 2011, GSK 2010	expansion
Rasagiline	Sympathomimetic	Yes-serotonin syndrome		Dey 2010, Validus 2007, Parke-Davis 2011, GSK 2010	expansion
Rasagiline	Tramadol	Yes-serotonin syndrome		Dey 2010, Validus 2007, Parke-Davis 2011, GSK 2010	expansion
Rasagiline	Tranylcypromine	Yes-serotonin syndrome		Dey 2010, Validus 2007, Parke-Davis 2011, GSK 2010	expansion
Rasagiline	Trazodone	Yes-serotonin syndrome		Dey 2010, Validus 2007, Parke-Davis 2011, GSK 2010	expansion
Rasagiline	Trimipramine	Yes-serotonin syndrome		Dey 2010, Validus 2007, Parke-Davis 2011, GSK 2010	expansion
Rasagiline	Tryptophan	Yes-hypertension		Dey 2010, Validus 2007, Parke-Davis 2011, GSK 2010	expansion
Rasagiline	Tryptophan	Yes-serotonin syndrome		Dey 2010, Validus 2007, Parke-Davis 2011, GSK 2010	expansion

Rasagiline	Venlafaxine	Yes-serotonin syndrome	Dey 2010, Validus 2007, Parke-Davis 2011, GSK 2010	expansion
Rasagiline	Vilazodone	Yes-serotonin syndrome	Dey 2010, Validus 2007, Parke-Davis 2011, GSK 2010	expansion
SNRI	Antipsychotic	No	Rebel 2010b	expansion
SNRI	Antipsychotic	Yes-seizure	Rebel 2010b	expansion
SNRI	Aspirin	Yes-GI bleeding	Eli-Lilly 2010, Rebel 2010b, Wyeth 2011	original
SNRI	Azole Antifungal	Yes	Cardinal Health, 2011	expansion
SNRI	Azole Antifungal	Yes	Rebel 2010b	expansion
SNRI	Benzodiazepine	No	Eli-Lilly 2010	expansion
SNRI	Benzodiazepine	No	Rebel 2010b	expansion
SNRI	Beta-adrenergic blocker	No	Rebel 2010b	expansion
SNRI	Beta-adrenergic blocker	Yes-hypertension	Rebel 2010b	expansion
SNRI	Buspirone	Yes-serotonin syndrome	Eli-Lilly 2010, Rebel 2010b, Wyeth 2011	original
SNRI	Cimetidine	Yes	Rebel 2010b	expansion
SNRI	Clozapine	Yes-seizure	Rebel 2010b	expansion
SNRI	Dextromethorphan	Yes-serotonin syndrome	Eli-Lilly 2010, Rebel 2010b, Wyeth 2011	original
SNRI	Diazepam	No	Rebel 2010b	expansion
SNRI	Fentanyl	Yes-serotonin syndrome	Eli-Lilly 2010, Rebel 2010b, Wyeth 2011	original
SNRI	Fluvoxamine	Yes	Eli-Lilly 2010	expansion
SNRI	H2 blockers	Yes	Rebel 2010b	expansion
SNRI	Heparin	Yes-GI bleeding	Eli-Lilly 2010, Rebel 2010b, Wyeth 2011	original
SNRI	Imipramine	No	Rebel 2010b	expansion
SNRI	Indinavir	No	Rebel 2010b	expansion
SNRI	Ketoconazole	Yes	Cardinal Health, 2011	expansion
SNRI	Ketoconazole	Yes	Rebel 2010b	expansion
SNRI	Linezolid	Yes-serotonin syndrome	Eli-Lilly 2010, Rebel 2010b, Wyeth 2011	original
SNRI	Lithium	No	Rebel 2010b	expansion
SNRI	Lorazepam	No	Eli-Lilly 2010	expansion
SNRI	MAOI	Yes-serotonin syndrome	Eli-Lilly 2010, Rebel 2010b, Wyeth 2011	original
SNRI	Meperidine	Yes-serotonin syndrome	Eli-Lilly 2010, Rebel 2010b, Wyeth 2011	original
SNRI	Metoprolol	No	Rebel 2010b	expansion
SNRI	Metoprolol	Yes-hypertension	Rebel 2010b	expansion
SNRI	NSAID	Yes-GI bleeding	Eli-Lilly 2010, Rebel 2010b, Wyeth 2011	original

SNRI	Odansetran	Yes-serotonin syndrome	Eli-Lilly 2010, Rebel 2010b, Wyeth 2011	original
SNRI	Paroxetine	Yes	Eli-Lilly 2010	expansion
SNRI	Pentazocine	Yes-serotonin syndrome	Eli-Lilly 2010, Rebel 2010b, Wyeth 2011	original
SNRI	Protease Inhibitor	No	Rebel 2010b	expansion
SNRI	SEROTONIN-1B AND SERC	Yes-serotonin syndrome	Eli-Lilly 2010, Rebel 2010b, Wyeth 2011	expansion
SNRI	SNRI	Yes-serotonin syndrome	Eli-Lilly 2010, Rebel 2010b, Wyeth 2011	original
SNRI	SSRI	Yes	Eli-Lilly 2010	expansion
SNRI	SSRI	Yes-serotonin syndrome	Eli-Lilly 2010, Rebel 2010b, Wyeth 2011	original
SNRI	St Johns wort	Yes-serotonin syndrome	Eli-Lilly 2010, Rebel 2010b, Wyeth 2011	original
SNRI	Sumatriptan	Yes-serotonin syndrome	Eli-Lilly 2010, Rebel 2010b, Wyeth 2011	original
SNRI	Sympathomimetic	Yes-serotonin syndrome	Eli-Lilly 2010, Rebel 2010b, Wyeth 2011	expansion
SNRI	TCA	No	Rebel 2010b	expansion
SNRI	TCA	Yes-serotonin syndrome	Eli-Lilly 2010, Rebel 2010b, Wyeth 2011	original
SNRI	Temazepam	No	Eli-Lilly 2010	expansion
SNRI	Thrombin Inhibitors	Yes-GI bleeding	Eli-Lilly 2010, Rebel 2010b, Wyeth 2011	original
SNRI	Tramadol	Yes-serotonin syndrome	Eli-Lilly 2010, Rebel 2010b, Wyeth 2011	original
SNRI	Tryptophan	Yes-serotonin syndrome	Eli-Lilly 2010, Rebel 2010b, Wyeth 2011	original
SNRI	VITAMIN K ANTAGONIST	Yes-GI bleeding	Eli-Lilly 2010, Rebel 2010b, Wyeth 2011	expansion
SNRI	Warfarin	Yes-GI bleeding	Eli-Lilly 2010, Rebel 2010b, Wyeth 2011	original
SSRI	Acetaminophen	Yes-GI bleeding	Labopharm 2010	expansion
SSRI	Alprazolam	???	???	expansion
SSRI	Aminoketone	Yes-bleeding	Forest 2009, Forest 2011, Dista 2009, Alphapharm 2007, Watson 2008, Forest 2011B	expansion
SSRI	Amitriptyline	Yes-serotonin syndrome	Organon 2010, Rebel 2010, Labopharm 2010	expansion

SSRI	Amoxapine	Yes-serotonin syndrome	Organon 2010, Rebel 2010, Labopharm 2010	expansion
SSRI	Anticholinergic	Yes-anticholinergic effects	Alphapharm 2007	expansion
SSRI	Antihistamine	Yes-QTc interval prolongation	Rebel 2010	expansion
SSRI	Antipsychotic	No	Alpharpharm 2007	expansion
SSRI	Antipsychotic	No	Forest 2009, Forest 2011	expansion
SSRI	Antipsychotic	No	Rebel 2010	expansion
SSRI	Antipsychotic	Yes-Lithium toxicity	Dista 2009	expansion
SSRI	Antipsychotic	Yes-QTc interval prolongation	Dista 2009	expansion
SSRI	Antipsychotic	Yes-QTc interval prolongation	Forest 2009, Forest 2011, Dista 2009, Alphapharm 2007, Watson 2008	expansion
SSRI	Antipsychotic	Yes-QTc interval prolongation	Rebel 2010	expansion
SSRI	Antithrombin alfa	Yes-GI bleeding	Labopharm 2010	expansion
SSRI	Argatroban	Yes-GI bleeding	Labopharm 2010	expansion
SSRI	Aspirin	Yes-bleeding	Forest 2009, Forest 2011, Dista 2009, Alphapharm 2007, Watson 2008, Forest 2011B	original
SSRI	Astemizole	Yes-QTc interval prolongation	Rebel 2010	expansion
SSRI	Atenolol	No	Watson 2008	expansion
SSRI	Atorvastatin	Yes-Rhabdomyolyses	Rebel 2010	expansion
SSRI	Azole Antifungal	No	Forest 2009, Forest 2011	expansion
SSRI	Azole Antifungal	Yes	Forest 2011B	expansion
SSRI	Benzodiazepine	???	???	expansion
SSRI	Benzodiazepine	No	Alpharpharm 2007	expansion
SSRI	Benzodiazepine	No	Rebel 2010	expansion
SSRI	Beta-adrenergic blocker	No	Watson 2008	expansion
SSRI	Beta-adrenergic blocker	Yes	Rebel 2010	expansion
SSRI	Bivalirudin	Yes-GI bleeding	Labopharm 2010	expansion
SSRI	Bromfenac	Yes-GI bleeding	Labopharm 2010	expansion
SSRI	Bupropion	Yes-bleeding	Forest 2009, Forest 2011, Dista 2009, Alphapharm 2007, Watson 2008, Forest 2011B	original
SSRI	Buspirone	Yes	Rebel 2010	expansion
SSRI	Buspirone	Yes-serotonin syndrome	Forest 2009, Forest 2011, Dista 2009, Alphapharm 2007, Watson 2008, Forest 2011B (Vilazodone)	original

SSRI	Cardiac Glycoside	No	Alpharpharm 2007	expansion
SSRI	Cardiac Glycoside	No	Forest 2009, Forest 2011	expansion
SSRI	Cardiac Glycoside	No	Rebel 2010	expansion
SSRI	Celecoxib	Yes-GI bleeding	Labopharm 2010	expansion
SSRI	Cimetidine	No	Rebel 2010	expansion
SSRI	Cimetidine	Yes	Alpharpharm 2007	expansion
SSRI	Cimetidine	Yes	Forest 2009, Forest 2011	expansion
SSRI	Cimetidine	Yes	Watson 2008	expansion
SSRI	Cisapride	Yes-QTc interval prolongation	Rebel 2010	expansion
SSRI	Citalopram	Yes-serotonin syndrome	Organon 2010, Rebel 2010, Labopharm 2010	expansion
SSRI	Clomipramine	Yes-serotonin syndrome	Organon 2010, Rebel 2010, Labopharm 2010	expansion
SSRI	Desipramine	Yes	Rebel 2010	expansion
SSRI	Desipramine	Yes-serotonin syndrome	Organon 2010, Rebel 2010, Labopharm 2010	expansion
SSRI	Desirudin	Yes-GI bleeding	Labopharm 2010	expansion
SSRI	Desvenlafaxine	Yes-serotonin syndrome	Organon 2010, Rebel 2010, Labopharm 2010	expansion
SSRI	Dextromethorphan	Yes-serotonin syndrome	Forest 2009, Forest 2011, Dista 2009, Alphapharm 2007, Watson 2008, Forest 2011B (Vilazodone)	original
SSRI	Diazepam	No	Alpharpharm 2007	expansion
SSRI	Diclofenac	Yes-GI bleeding	Labopharm 2010	expansion
SSRI	Diflunisal	Yes-GI bleeding	Labopharm 2010	expansion
SSRI	Digoxin	No	Alpharpharm 2007	expansion
SSRI	Digoxin	No	Forest 2009, Forest 2011	expansion
SSRI	Digoxin	No	Rebel 2010	expansion
SSRI	Doxepin	Yes-serotonin syndrome	Organon 2010, Rebel 2010, Labopharm 2010	expansion
SSRI	Duloxetine	Yes-serotonin syndrome	Organon 2010, Rebel 2010, Labopharm 2010	expansion
SSRI	Escitalopram	Yes-serotonin syndrome	Organon 2010, Rebel 2010, Labopharm 2010	expansion
SSRI	Etodolac	Yes-GI bleeding	Labopharm 2010	expansion
SSRI	Ezetimibe	Yes-Rhabdomyolyses	Rebel 2010	expansion
SSRI	Fenoprofen	Yes-GI bleeding	Labopharm 2010	expansion

SSRI	Fentanyl	Yes-serotonin syndrome	Forest 2009, Forest 2011, Dista 2009, Alphapharm 2007, Watson 2008, Forest 2011B (Vilazodone)	original
SSRI	Fluoxetine	Yes	Rebel 2010	expansion
SSRI	Fluoxetine	Yes-headache, lightheadedness, nausea, or paresth	Rebel 2010	expansion
SSRI	Fluoxetine	Yes-serotonin syndrome	Organon 2010, Rebel 2010, Labopharm 2010	expansion
SSRI	Flurbiprofen	Yes-GI bleeding	Labopharm 2010	expansion
SSRI	Fluvastatin	Yes-Rhabdomyolyses	Rebel 2010	expansion
SSRI	Fluvoxamine	Yes-serotonin syndrome	Organon 2010, Rebel 2010, Labopharm 2010	expansion
SSRI	H2 blockers	No	Rebel 2010	expansion
SSRI	H2 blockers	Yes	Alpharpharm 2007	expansion
SSRI	H2 blockers	Yes	Forest 2009, Forest 2011	expansion
SSRI	H2 blockers	Yes	Watson 2008	expansion
SSRI	HMG-CoA Reductase Inhil	Yes-Rhabdomyolyses	Rebel 2010	expansion
SSRI	Haloperidol	No	Rebel 2010	expansion
SSRI	Heparin	Yes-bleeding	Forest 2009, Forest 2011, Dista 2009, Alphapharm 2007, Watson 2008, Forest 2011B	original
SSRI	Ibuprofen	Yes-GI bleeding	Labopharm 2010	expansion
SSRI	Imipramine	Yes-serotonin syndrome	Organon 2010, Rebel 2010, Labopharm 2010	expansion
SSRI	Indomethacin	Yes-GI bleeding	Labopharm 2010	expansion
SSRI	Isocarboxazid	Yes-serotonin syndrome	Organon 2010, Rebel 2010, Labopharm 2010	expansion
SSRI	Ketoconazole	No	Forest 2009, Forest 2011	expansion
SSRI	Ketoconazole	Yes	Forest 2011B	expansion
SSRI	Ketoprofen	Yes-GI bleeding	Labopharm 2010	expansion
SSRI	Ketorolac tromethamine	Yes-GI bleeding	Labopharm 2010	expansion
SSRI	Ketorolac	Yes-GI bleeding	Labopharm 2010	expansion
SSRI	Lepirudin	Yes-GI bleeding	Labopharm 2010	expansion
SSRI	Linezolid	Yes-serotonin syndrome	Forest 2009, Forest 2011, Dista 2009, Alphapharm 2007, Watson 2008, Forest 2011B (Vilazodone)	original
SSRI	Lithium	No	Alpharpharm 2007	expansion

SSRI	Lithium	No	Forest 2009, Forest 2011	expansion
SSRI	Lithium	No	Rebel 2010	expansion
SSRI	Lithium	Yes-Lithium toxicity	Dista 2009	expansion
SSRI	Lorazepam	No	Rebel 2010	expansion
SSRI	Lovastatin	Yes-Rhabdomyolyses	Rebel 2010	expansion
SSRI	MAOI	Yes-serotonin syndrome	Forest 2009, Forest 2011, Dista 2009, Alphapharm 2007, Watson 2008, Forest 2011B (Vilazodone)	original
SSRI	Mannitol	Yes-GI bleeding	Labopharm 2010	expansion
SSRI	Maprotiline	Yes-serotonin syndrome	Organon 2010, Rebel 2010, Labopharm 2010	expansion
SSRI	Mefenamic acid	Yes-GI bleeding	Labopharm 2010	expansion
SSRI	Meloxicam	Yes-GI bleeding	Labopharm 2010	expansion
SSRI	Meperidine	Yes-serotonin syndrome	Forest 2009, Forest 2011, Dista 2009, Alphapharm 2007, Watson 2008, Forest 2011B (Vilazodone)	original
SSRI	Methylxanthine	No	Rebel 2010	expansion
SSRI	Milnacipran	Yes-serotonin syndrome	Organon 2010, Rebel 2010, Labopharm 2010	expansion
SSRI	NSAID	Yes-bleeding	Forest 2009, Forest 2011, Dista 2009, Alphapharm 2007, Watson 2008, Forest 2011B	original
SSRI	Nabumetone	Yes-GI bleeding	Labopharm 2010	expansion
SSRI	Naproxen	Yes-GI bleeding	Labopharm 2010	expansion
SSRI	Nefazodone	Yes-serotonin syndrome	Organon 2010, Rebel 2010, Labopharm 2010	expansion
SSRI	Nepafenac	Yes-GI bleeding	Labopharm 2010	expansion
SSRI	Nortriptyline	Yes-serotonin syndrome	Organon 2010, Rebel 2010, Labopharm 2010	expansion
SSRI	Odansetran	Yes-serotonin syndrome	Forest 2009, Forest 2011, Dista 2009, Alphapharm 2007, Watson 2008, Forest 2011B (Vilazodone)	original
SSRI	Oxaprozin	Yes-GI bleeding	Labopharm 2010	expansion

SSRI	Paroxetine	Yes-serotonin syndrome	Organon 2010, Rebel 2010, Labopharm 2010	expansion
SSRI	Pentazocine	Yes-serotonin syndrome	Forest 2009, Forest 2011, Dista 2009, Alphapharm 2007, Watson 2008, Forest 2011B (Vilazodone)	original
SSRI	Phenelzine	Yes-serotonin syndrome	Organon 2010, Rebel 2010, Labopharm 2010	expansion
SSRI	Pimozide	Yes-QTc interval prolongation	Forest 2009, Forest 2011, Dista 2009, Alphapharm 2007, Watson 2008	original
SSRI	Piroxicam	Yes-GI bleeding	Labopharm 2010	expansion
SSRI	Pitavastatin	Yes-Rhabdomyolyses	Rebel 2010	expansion
SSRI	Pravastatin	Yes-Rhabdomyolyses	Rebel 2010	expansion
SSRI	Procyclidine	Yes-anticholinergic effects	Alphapharm 2007	expansion
SSRI	Propranolol	Yes	Rebel 2010	expansion
SSRI	Protease Inhibitor	No	Forest 2011	expansion
SSRI	Protease Inhibitor	Yes	Labopharm 2010	expansion
SSRI	Protriptyline	Yes-serotonin syndrome	Organon 2010, Rebel 2010, Labopharm 2010	expansion
SSRI	Rasagiline	Yes-serotonin syndrome	Organon 2010, Rebel 2010, Labopharm 2010	expansion
SSRI	Ritonavir	No	Forest 2011	expansion
SSRI	Ritonavir	Yes	Labopharm 2010	expansion
SSRI	Rosuvastatin	Yes-Rhabdomyolyses	Rebel 2010	expansion
SSRI	SEROTONIN-1B AND SERC	Yes-serotonin syndrome	Forest 2009, Forest 2011, Dista 2009, Alphapharm 2007, Watson 2008, Forest 2011B (Vilazodone)	expansion
SSRI	SEROTONIN-1B AND SERC	Yes-serotonin syndrome	Organon 2010, Rebel 2010, Labopharm 2010	expansion
SSRI	SEROTONIN-1B AND SERC	Yes-weakness, hyperreflexia, and incoordination	Forest 2009, Forest 2011, Watson 2008	expansion
SSRI	SNRI	Yes-serotonin syndrome	Forest 2009, Forest 2011, Dista 2009, Alphapharm 2007, Watson 2008, Forest 2011B (Vilazodone)	original
SSRI	SSRI	Yes	Rebel 2010	expansion

SSRI	SSRI	Yes-serotonin syndrome	Forest 2009, Forest 2011, Dista 2009, Alphapharm 2007, Watson 2008, Forest 2011B (Vilazodone)	original
SSRI	Salicylamide	Yes-GI bleeding	Labopharm 2010	expansion
SSRI	Sedative Hypnotics	???	???	expansion
SSRI	Sedative Hypnotics	No	Forest 2009, Forest 2011	expansion
SSRI	Selegiline	Yes-serotonin syndrome	Organon 2010, Rebel 2010, Labopharm 2010	expansion
SSRI	Sertraline	Yes-serotonin syndrome	Organon 2010, Rebel 2010, Labopharm 2010	expansion
SSRI	Simvastatin	Yes-Rhabdomyolyses	Rebel 2010	expansion
SSRI	St Johns wort	Yes-serotonin syndrome	Forest 2009, Forest 2011, Dista 2009, Alphapharm 2007, Watson 2008, Forest 2011B (Vilazodone)	original
SSRI	Sulindac	Yes-GI bleeding	Labopharm 2010	expansion
SSRI	Sumatriptan	Yes-serotonin syndrome	Forest 2009, Forest 2011, Dista 2009, Alphapharm 2007, Watson 2008, Forest 2011B (Vilazodone)	original
SSRI	Sumatriptan	Yes-weakness, hyperreflexia, and incoordination	Forest 2009, Forest 2011, Watson 2008	original
SSRI	Sympathomimetic	Yes-agitation, restlessness, GI distress	Dista 2009	expansion
SSRI	Sympathomimetic	Yes-headache, nausea, sweating, dizziness	Alphapharm 2007	expansion
SSRI	Sympathomimetic	Yes-serotonin syndrome	Forest 2009, Forest 2011, Dista 2009, Alphapharm 2007, Watson 2008, Forest 2011B (Vilazodone)	expansion
SSRI	Sympathomimetic	Yes-serotonin syndrome	Organon 2010, Rebel 2010, Labopharm 2010	expansion
SSRI	TCA	Yes	Rebel 2010	expansion

SSRI	TCA	Yes-serotonin syndrome	Forest 2009, Forest 2011, Dista 2009, Alphapharm 2007, Watson 2008, Forest 2011B (Vilazodone)	original
SSRI	Terfenadine	Yes-QTc interval prolongation	Rebel 2010	expansion
SSRI	Theophylline	No	Rebel 2010	expansion
SSRI	Thioridazine	Yes-QTc interval prolongation	Dista 2009	expansion
SSRI	Thrombin Inhibitors	Yes-bleeding	Forest 2009, Forest 2011, Dista 2009, Alphapharm 2007, Watson 2008, Forest 2011B	original
SSRI	Tolmetin	Yes-GI bleeding	Labopharm 2010	expansion
SSRI	Tramadol	Yes-serotonin syndrome	Forest 2009, Forest 2011, Dista 2009, Alphapharm 2007, Watson 2008, Forest 2011B (Vilazodone)	original
SSRI	Tranylcypromine	Yes-serotonin syndrome	Organon 2010, Rebel 2010, Labopharm 2010	expansion
SSRI	Trazodone	Yes-serotonin syndrome	Organon 2010, Rebel 2010, Labopharm 2010	expansion
SSRI	Triazolam	???	???	expansion
SSRI	Triazolam	No	Forest 2009, Forest 2011	expansion
SSRI	Trimipramine	Yes-serotonin syndrome	Organon 2010, Rebel 2010, Labopharm 2010	expansion
SSRI	Tryptophan	Yes-serotonin syndrome	Forest 2009, Forest 2011, Dista 2009, Alphapharm 2007, Watson 2008, Forest 2011B (Vilazodone)	original
SSRI	VITAMIN K ANTAGONIST	Yes-GI bleeding	Labopharm 2010	expansion
SSRI	VITAMIN K ANTAGONIST	Yes-bleeding	Forest 2009, Forest 2011, Dista 2009, Alphapharm 2007, Watson 2008, Forest 2011B	expansion
SSRI	Venlafaxine	Yes-serotonin syndrome	Organon 2010, Rebel 2010, Labopharm 2010	expansion

SSRI	Vilazodone	Yes-serotonin syndrome	Organon 2010, Rebel 2010, Labopharm 2010	expansion
SSRI	Warfarin	Yes-bleeding	Forest 2009, Forest 2011, Dista 2009, Alphapharm 2007, Watson 2008, Forest 2011B	original
Selegiline	Alprazolam	No	Dey 2010	original
Selegiline	Amitriptyline	Yes-serotonin syndrome	Dey 2010, Validus 2007, Parke-Davis 2011, GSK 2010	expansion
Selegiline	Amoxapine	Yes-serotonin syndrome	Dey 2010, Validus 2007, Parke-Davis 2011, GSK 2010	expansion
Selegiline	Anticholinergic	Yes-hypertension	Dey 2010, Validus 2007, Parke-Davis 2011, GSK 2010	expansion
Selegiline	Antipsychotic	No	Dey 2010	expansion
Selegiline	Azole Antifungal	No	Dey 2010	expansion
Selegiline	Benzodiazepine	No	Dey 2010	expansion
Selegiline	Buspirone	Yes-hypertension	Dey 2010, Validus 2007, Parke-Davis 2011, GSK 2010	expansion
Selegiline	Citalopram	Yes-serotonin syndrome	Dey 2010, Validus 2007, Parke-Davis 2011, GSK 2010	expansion
Selegiline	Clomipramine	Yes-serotonin syndrome	Dey 2010, Validus 2007, Parke-Davis 2011, GSK 2010	expansion
Selegiline	Desipramine	Yes-serotonin syndrome	Dey 2010, Validus 2007, Parke-Davis 2011, GSK 2010	expansion
Selegiline	Desvenlafaxine	Yes-serotonin syndrome	Dey 2010, Validus 2007, Parke-Davis 2011, GSK 2010	expansion
Selegiline	Dextromethorphan	Yes-serotonin syndrome	Dey 2010, Validus 2007, Parke-Davis 2011, GSK 2010	expansion
Selegiline	Doxepin	Yes-serotonin syndrome	Dey 2010, Validus 2007, Parke-Davis 2011, GSK 2010	expansion
Selegiline	Duloxetine	Yes-serotonin syndrome	Dey 2010, Validus 2007, Parke-Davis 2011, GSK 2010	expansion

Selegiline	Escitalopram	Yes-serotonin syndrome	Dey 2010, Validus 2007, Parke-Davis 2011, GSK 2010	expansion
Selegiline	Fentanyl	Yes-serotonin syndrome	Dey 2010, Validus 2007, Parke-Davis 2011, GSK 2010	expansion
Selegiline	Fluoxetine	Yes-serotonin syndrome	Dey 2010, Validus 2007, Parke-Davis 2011, GSK 2010	expansion
Selegiline	Fluvoxamine	Yes-serotonin syndrome	Dey 2010, Validus 2007, Parke-Davis 2011, GSK 2010	expansion
Selegiline	Ibuprofen	No	Dey 2010	original
Selegiline	Imipramine	Yes-serotonin syndrome	Dey 2010, Validus 2007, Parke-Davis 2011, GSK 2010	expansion
Selegiline	Isocarboxazid	Yes-serotonin syndrome	Dey 2010, Validus 2007, Parke-Davis 2011, GSK 2010	expansion
Selegiline	Ketoconazole	No	Dey 2010	original
Selegiline	Levothyroxine	???	???	original
Selegiline	Linezolid	Yes-serotonin syndrome	Dey 2010, Validus 2007, Parke-Davis 2011, GSK 2010	expansion
Selegiline	Maprotiline	Yes-serotonin syndrome	Dey 2010, Validus 2007, Parke-Davis 2011, GSK 2010	expansion
Selegiline	Meperidine	Yes- coma, severe hypertension or hypotension, se	Dey 2010, Validus 2007, Parke-Davis 2011, GSK 2010	expansion
Selegiline	Milnacipran	Yes-serotonin syndrome	Dey 2010, Validus 2007, Parke-Davis 2011, GSK 2010	expansion
Selegiline	Modafinil	Yes-hypertension	Dey 2010, Validus 2007, Parke-Davis 2011, GSK 2010	expansion
Selegiline	NSAID	No	Dey 2010	expansion
Selegiline	Nefazodone	Yes-serotonin syndrome	Dey 2010, Validus 2007, Parke-Davis 2011, GSK 2010	expansion
Selegiline	Nortriptyline	Yes-serotonin syndrome	Dey 2010, Validus 2007, Parke-Davis 2011, GSK 2010	expansion

Selegiline	Odansetran	Yes-serotonin syndrome	Dey 2010, Validus 2007, Parke-Davis 2011, GSK 2010	expansion
Selegiline	Olanzapine	No	Dey 2010	original
Selegiline	Paroxetine	Yes-serotonin syndrome	Dey 2010, Validus 2007, Parke-Davis 2011, GSK 2010	expansion
Selegiline	Pentazocine	Yes-serotonin syndrome	Dey 2010, Validus 2007, Parke-Davis 2011, GSK 2010	expansion
Selegiline	Phenelzine	Yes-serotonin syndrome	Dey 2010, Validus 2007, Parke-Davis 2011, GSK 2010	expansion
Selegiline	Phenylpropanolamine	Yes-hypertension	Dey 2010, Validus 2007, Parke-Davis 2011, GSK 2010	expansion
Selegiline	Protriptyline	Yes-serotonin syndrome	Dey 2010, Validus 2007, Parke-Davis 2011, GSK 2010	expansion
Selegiline	Pseudoephedrine	Yes-hypertension	Dey 2010, Validus 2007, Parke-Davis 2011, GSK 2010	expansion
Selegiline	Rasagiline	Yes-serotonin syndrome	Dey 2010, Validus 2007, Parke-Davis 2011, GSK 2010	expansion
Selegiline	Risperidone	No	Dey 2010	original
Selegiline	SEROTONIN-1B AND SERC	Yes-serotonin syndrome	Dey 2010, Validus 2007, Parke-Davis 2011, GSK 2010	expansion
Selegiline	Selegiline	Yes-serotonin syndrome	Dey 2010, Validus 2007, Parke-Davis 2011, GSK 2010	expansion
Selegiline	Sertraline	Yes-serotonin syndrome	Dey 2010, Validus 2007, Parke-Davis 2011, GSK 2010	expansion
Selegiline	St Johns wort	Yes-serotonin syndrome	Dey 2010, Validus 2007, Parke-Davis 2011, GSK 2010	expansion
Selegiline	Sumatriptan	Yes-serotonin syndrome	Dey 2010, Validus 2007, Parke-Davis 2011, GSK 2010	expansion
Selegiline	Sympathomimetic	Yes-hypertension	Dey 2010, Validus 2007, Parke-Davis 2011, GSK 2010	expansion

Selegiline	Sympathomimetic	Yes-serotonin syndrome	Dey 2010, Validus 2007, Parke-Davis 2011, GSK 2010	expansion
Selegiline	Tramadol	Yes-serotonin syndrome	Dey 2010, Validus 2007, Parke-Davis 2011, GSK 2010	expansion
Selegiline	Tranylcypromine	Yes-serotonin syndrome	Dey 2010, Validus 2007, Parke-Davis 2011, GSK 2010	expansion
Selegiline	Trazodone	Yes-serotonin syndrome	Dey 2010, Validus 2007, Parke-Davis 2011, GSK 2010	expansion
Selegiline	Trimipramine	Yes-serotonin syndrome	Dey 2010, Validus 2007, Parke-Davis 2011, GSK 2010	expansion
Selegiline	Tryptophan	Yes-hypertension	Dey 2010, Validus 2007, Parke-Davis 2011, GSK 2010	expansion
Selegiline	Tryptophan	Yes-serotonin syndrome	Dey 2010, Validus 2007, Parke-Davis 2011, GSK 2010	expansion
Selegiline	Venlafaxine	Yes-serotonin syndrome	Dey 2010, Validus 2007, Parke-Davis 2011, GSK 2010	expansion
Selegiline	Vilazodone	Yes-serotonin syndrome	Dey 2010, Validus 2007, Parke-Davis 2011, GSK 2010	expansion
Sertraline	Acetaminophen	Yes-bleeding	Forest 2009, Forest 2011, Dista 2009, Alphapharm 2007, Watson 2008, Forest 2011B	expansion
Sertraline	Aminoketone	Yes-bleeding	Forest 2009, Forest 2011, Dista 2009, Alphapharm 2007, Watson 2008, Forest 2011B	expansion
Sertraline	Amitriptyline	Yes-serotonin syndrome	Forest 2009, Forest 2011, Dista 2009, Alphapharm 2007, Watson 2008, Forest 2011B (Vilazodone)	expansion

Sertraline	Amoxapine	Yes-serotonin syndrome	Forest 2009, Forest 2011, Dista 2009, Alphapharm 2007, Watson 2008, Forest 2011B (Vilazodone)	expansion
Sertraline	Antipsychotic	Yes-QTc interval prolongation	Forest 2009, Forest 2011, Dista 2009, Alphapharm 2007, Watson 2008	expansion
Sertraline	Antithrombin alfa	Yes-bleeding	Forest 2009, Forest 2011, Dista 2009, Alphapharm 2007, Watson 2008, Forest 2011B	expansion
Sertraline	Argatroban	Yes-bleeding	Forest 2009, Forest 2011, Dista 2009, Alphapharm 2007, Watson 2008, Forest 2011B	expansion
Sertraline	Aspirin	Yes-bleeding	Forest 2009, Forest 2011, Dista 2009, Alphapharm 2007, Watson 2008, Forest 2011B	expansion
Sertraline	Atenolol	No	Watson 2008	original
Sertraline	Beta-adrenergic blocker	No	Watson 2008	expansion
Sertraline	Bivalirudin	Yes-bleeding	Forest 2009, Forest 2011, Dista 2009, Alphapharm 2007, Watson 2008, Forest 2011B	expansion
Sertraline	Bromfenac	Yes-bleeding	Forest 2009, Forest 2011, Dista 2009, Alphapharm 2007, Watson 2008, Forest 2011B	expansion
Sertraline	Bupropion	Yes-bleeding	Forest 2009, Forest 2011, Dista 2009, Alphapharm 2007, Watson 2008, Forest 2011B	expansion

Sertraline	Buspirone	Yes-serotonin syndrome	Forest 2009, Forest 2011, Dista 2009, Alphapharm 2007, Watson 2008, Forest 2011B (Vilazodone)	expansion
Sertraline	Celecoxib	Yes-bleeding	Forest 2009, Forest 2011, Dista 2009, Alphapharm 2007, Watson 2008, Forest 2011B	expansion
Sertraline	Cimetidine	Yes	Watson 2008	original
Sertraline	Citalopram	Yes-serotonin syndrome	Forest 2009, Forest 2011, Dista 2009, Alphapharm 2007, Watson 2008, Forest 2011B (Vilazodone)	expansion
Sertraline	Clomipramine	Yes-serotonin syndrome	Forest 2009, Forest 2011, Dista 2009, Alphapharm 2007, Watson 2008, Forest 2011B (Vilazodone)	expansion
Sertraline	Desipramine	Yes-serotonin syndrome	Forest 2009, Forest 2011, Dista 2009, Alphapharm 2007, Watson 2008, Forest 2011B (Vilazodone)	expansion
Sertraline	Desirudin	Yes-bleeding	Forest 2009, Forest 2011, Dista 2009, Alphapharm 2007, Watson 2008, Forest 2011B	expansion
Sertraline	Desvenlafaxine	Yes-serotonin syndrome	Forest 2009, Forest 2011, Dista 2009, Alphapharm 2007, Watson 2008, Forest 2011B (Vilazodone)	expansion

Sertraline	Dextromethorphan	Yes-serotonin syndrome	Forest 2009, Forest 2011, Dista 2009, Alphapharm 2007, Watson 2008, Forest 2011B (Vilazodone)	expansion
Sertraline	Diclofenac	Yes-bleeding	Forest 2009, Forest 2011, Dista 2009, Alphapharm 2007, Watson 2008, Forest 2011B	expansion
Sertraline	Diflunisal	Yes-bleeding	Forest 2009, Forest 2011, Dista 2009, Alphapharm 2007, Watson 2008, Forest 2011B	expansion
Sertraline	Doxepin	Yes-serotonin syndrome	Forest 2009, Forest 2011, Dista 2009, Alphapharm 2007, Watson 2008, Forest 2011B (Vilazodone)	expansion
Sertraline	Duloxetine	Yes-serotonin syndrome	Forest 2009, Forest 2011, Dista 2009, Alphapharm 2007, Watson 2008, Forest 2011B (Vilazodone)	expansion
Sertraline	Escitalopram	Yes-serotonin syndrome	Forest 2009, Forest 2011, Dista 2009, Alphapharm 2007, Watson 2008, Forest 2011B (Vilazodone)	expansion
Sertraline	Etodolac	Yes-bleeding	Forest 2009, Forest 2011, Dista 2009, Alphapharm 2007, Watson 2008, Forest 2011B	expansion

Sertraline	Fenoprofen	Yes-bleeding	Forest 2009, Forest 2011, Dista 2009, Alphapharm 2007, Watson 2008, Forest 2011B	expansion
Sertraline	Fentanyl	Yes-serotonin syndrome	Forest 2009, Forest 2011, Dista 2009, Alphapharm 2007, Watson 2008, Forest 2011B (Vilazodone)	expansion
Sertraline	Fluoxetine	Yes-serotonin syndrome	Forest 2009, Forest 2011, Dista 2009, Alphapharm 2007, Watson 2008, Forest 2011B (Vilazodone)	expansion
Sertraline	Flurbiprofen	Yes-bleeding	Forest 2009, Forest 2011, Dista 2009, Alphapharm 2007, Watson 2008, Forest 2011B	expansion
Sertraline	Fluvoxamine	Yes-serotonin syndrome	Forest 2009, Forest 2011, Dista 2009, Alphapharm 2007, Watson 2008, Forest 2011B (Vilazodone)	expansion
Sertraline	H2 blockers	Yes	Watson 2008	expansion
Sertraline	Heparin	Yes-bleeding	Forest 2009, Forest 2011, Dista 2009, Alphapharm 2007, Watson 2008, Forest 2011B	expansion
Sertraline	Ibuprofen	Yes-bleeding	Forest 2009, Forest 2011, Dista 2009, Alphapharm 2007, Watson 2008, Forest 2011B	expansion

Sertraline	Imipramine	Yes-serotonin syndrome	Forest 2009, Forest 2011, Dista 2009, Alphapharm 2007, Watson 2008, Forest 2011B (Vilazodone)	expansion
Sertraline	Indomethacin	Yes-bleeding	Forest 2009, Forest 2011, Dista 2009, Alphapharm 2007, Watson 2008, Forest 2011B	expansion
Sertraline	Isocarboxazid	Yes-serotonin syndrome	Forest 2009, Forest 2011, Dista 2009, Alphapharm 2007, Watson 2008, Forest 2011B (Vilazodone)	expansion
Sertraline	Ketoprofen	Yes-bleeding	Forest 2009, Forest 2011, Dista 2009, Alphapharm 2007, Watson 2008, Forest 2011B	expansion
Sertraline	Ketorolac tromethamine	Yes-bleeding	Forest 2009, Forest 2011, Dista 2009, Alphapharm 2007, Watson 2008, Forest 2011B	expansion
Sertraline	Ketorolac	Yes-bleeding	Forest 2009, Forest 2011, Dista 2009, Alphapharm 2007, Watson 2008, Forest 2011B	expansion
Sertraline	Lepirudin	Yes-bleeding	Forest 2009, Forest 2011, Dista 2009, Alphapharm 2007, Watson 2008, Forest 2011B	expansion
Sertraline	Linezolid	Yes-serotonin syndrome	Forest 2009, Forest 2011, Dista 2009, Alphapharm 2007, Watson 2008, Forest 2011B (Vilazodone)	expansion

Sertraline	Mannitol	Yes-bleeding	Forest 2009, Forest 2011, Dista 2009, Alphapharm 2007, Watson 2008, Forest 2011B	expansion
Sertraline	Maprotiline	Yes-serotonin syndrome	Forest 2009, Forest 2011, Dista 2009, Alphapharm 2007, Watson 2008, Forest 2011B (Vilazodone)	expansion
Sertraline	Mefenamic acid	Yes-bleeding	Forest 2009, Forest 2011, Dista 2009, Alphapharm 2007, Watson 2008, Forest 2011B	expansion
Sertraline	Meloxicam	Yes-bleeding	Forest 2009, Forest 2011, Dista 2009, Alphapharm 2007, Watson 2008, Forest 2011B	expansion
Sertraline	Meperidine	Yes-serotonin syndrome	Forest 2009, Forest 2011, Dista 2009, Alphapharm 2007, Watson 2008, Forest 2011B (Vilazodone)	expansion
Sertraline	Milnacipran	Yes-serotonin syndrome	Forest 2009, Forest 2011, Dista 2009, Alphapharm 2007, Watson 2008, Forest 2011B (Vilazodone)	expansion
Sertraline	NSAID	Yes-bleeding	Forest 2009, Forest 2011, Dista 2009, Alphapharm 2007, Watson 2008, Forest 2011B	expansion
Sertraline	Nabumetone	Yes-bleeding	Forest 2009, Forest 2011, Dista 2009, Alphapharm 2007, Watson 2008, Forest 2011B	expansion

Sertraline	Naproxen	Yes-bleeding	Forest 2009, Forest 2011, Dista 2009, Alphapharm 2007, Watson 2008, Forest 2011B	expansion
Sertraline	Nefazodone	Yes-serotonin syndrome	Forest 2009, Forest 2011, Dista 2009, Alphapharm 2007, Watson 2008, Forest 2011B (Vilazodone)	expansion
Sertraline	Nepafenac	Yes-bleeding	Forest 2009, Forest 2011, Dista 2009, Alphapharm 2007, Watson 2008, Forest 2011B	expansion
Sertraline	Nortriptyline	Yes-serotonin syndrome	Forest 2009, Forest 2011, Dista 2009, Alphapharm 2007, Watson 2008, Forest 2011B (Vilazodone)	expansion
Sertraline	Odansetran	Yes-serotonin syndrome	Forest 2009, Forest 2011, Dista 2009, Alphapharm 2007, Watson 2008, Forest 2011B (Vilazodone)	expansion
Sertraline	Oxaprozin	Yes-bleeding	Forest 2009, Forest 2011, Dista 2009, Alphapharm 2007, Watson 2008, Forest 2011B	expansion
Sertraline	Paroxetine	Yes-serotonin syndrome	Forest 2009, Forest 2011, Dista 2009, Alphapharm 2007, Watson 2008, Forest 2011B (Vilazodone)	expansion

Sertraline	Pentazocine	Yes-serotonin syndrome	Forest 2009, Forest 2011, Dista 2009, Alphapharm 2007, Watson 2008, Forest 2011B (Vilazodone)	expansion
Sertraline	Phenelzine	Yes-serotonin syndrome	Forest 2009, Forest 2011, Dista 2009, Alphapharm 2007, Watson 2008, Forest 2011B (Vilazodone)	expansion
Sertraline	Pimozide	Yes-QTc interval prolongation	Forest 2009, Forest 2011, Dista 2009, Alphapharm 2007, Watson 2008	expansion
Sertraline	Piroxicam	Yes-bleeding	Forest 2009, Forest 2011, Dista 2009, Alphapharm 2007, Watson 2008, Forest 2011B	expansion
Sertraline	Protriptyline	Yes-serotonin syndrome	Forest 2009, Forest 2011, Dista 2009, Alphapharm 2007, Watson 2008, Forest 2011B (Vilazodone)	expansion
Sertraline	Rasagiline	Yes-serotonin syndrome	Forest 2009, Forest 2011, Dista 2009, Alphapharm 2007, Watson 2008, Forest 2011B (Vilazodone)	expansion
Sertraline	SEROTONIN-1B AND SERC	Yes-serotonin syndrome	Forest 2009, Forest 2011, Dista 2009, Alphapharm 2007, Watson 2008, Forest 2011B (Vilazodone)	expansion
Sertraline	SEROTONIN-1B AND SERC	Yes-weakness, hyperreflexia, and incoordination	Forest 2009, Forest 2011, Watson 2008	expansion

Sertraline	Salicylamide	Yes-bleeding	Forest 2009, Forest 2011, Dista 2009, Alphapharm 2007, Watson 2008, Forest 2011B	expansion
Sertraline	Selegiline	Yes-serotonin syndrome	Forest 2009, Forest 2011, Dista 2009, Alphapharm 2007, Watson 2008, Forest 2011B (Vilazodone)	expansion
Sertraline	Sertraline	Yes-serotonin syndrome	Forest 2009, Forest 2011, Dista 2009, Alphapharm 2007, Watson 2008, Forest 2011B (Vilazodone)	expansion
Sertraline	St Johns wort	Yes-serotonin syndrome	Forest 2009, Forest 2011, Dista 2009, Alphapharm 2007, Watson 2008, Forest 2011B (Vilazodone)	expansion
Sertraline	Sulindac	Yes-bleeding	Forest 2009, Forest 2011, Dista 2009, Alphapharm 2007, Watson 2008, Forest 2011B	expansion
Sertraline	Sumatriptan	Yes-serotonin syndrome	Forest 2009, Forest 2011, Dista 2009, Alphapharm 2007, Watson 2008, Forest 2011B (Vilazodone)	expansion
Sertraline	Sumatriptan	Yes-weakness, hyperreflexia, and incoordination	Forest 2009, Forest 2011, Watson 2008	expansion
Sertraline	Sympathomimetic	Yes-serotonin syndrome	Forest 2009, Forest 2011, Dista 2009, Alphapharm 2007, Watson 2008, Forest 2011B (Vilazodone)	expansion

Sertraline	Tolmetin	Yes-bleeding	Forest 2009, Forest 2011, Dista 2009, Alphapharm 2007, Watson 2008, Forest 2011B	expansion
Sertraline	Tramadol	Yes-serotonin syndrome	Forest 2009, Forest 2011, Dista 2009, Alphapharm 2007, Watson 2008, Forest 2011B (Vilazodone)	expansion
Sertraline	Tranylcypromine	Yes-serotonin syndrome	Forest 2009, Forest 2011, Dista 2009, Alphapharm 2007, Watson 2008, Forest 2011B (Vilazodone)	expansion
Sertraline	Trazodone	Yes-serotonin syndrome	Forest 2009, Forest 2011, Dista 2009, Alphapharm 2007, Watson 2008, Forest 2011B (Vilazodone)	expansion
Sertraline	Trimipramine	Yes-serotonin syndrome	Forest 2009, Forest 2011, Dista 2009, Alphapharm 2007, Watson 2008, Forest 2011B (Vilazodone)	expansion
Sertraline	Tryptophan	Yes-serotonin syndrome	Forest 2009, Forest 2011, Dista 2009, Alphapharm 2007, Watson 2008, Forest 2011B (Vilazodone)	expansion
Sertraline	VITAMIN K ANTAGONIST	Yes-bleeding	Forest 2009, Forest 2011, Dista 2009, Alphapharm 2007, Watson 2008, Forest 2011B	expansion

Sertraline	Venlafaxine	Yes-serotonin syndrome	Forest 2009, Forest 2011, Dista 2009, Alphapharm 2007, Watson 2008, Forest 2011B (Vilazodone)	expansion
Sertraline	Vilazodone	Yes-serotonin syndrome	Forest 2009, Forest 2011, Dista 2009, Alphapharm 2007, Watson 2008, Forest 2011B (Vilazodone)	expansion
Sertraline	Warfarin	Yes-bleeding	Forest 2009, Forest 2011, Dista 2009, Alphapharm 2007, Watson 2008, Forest 2011B	expansion
TCA	4-hydroxybutanoic acid	Yes-additive CNS depressant effects	Sandoz 2010b, Duramed 2007	expansion
TCA	4-hydroxybutanoic acid	Yes-additive CNS depressant effects	Watson 2009	expansion
TCA	Alprazolam	Yes- Neuroleptic Malignant Syndrome	Mylan 2009	expansion
TCA	Amantadine	Yes	Sandoz 2010b, Duramed 2007	expansion
TCA	Amantadine	Yes-Additive side-effects	Mylan 2007	expansion
TCA	Amantadine	Yes-Additive side-effects	Sandoz 2010	expansion
TCA	Amantadine	Yes-Hyperpyrexia, Paralytic ileus	Mylan 2009	expansion
TCA	Amantadine	Yes-Paralytic ileus	Watson 2009	expansion
TCA	Amantadine	Yes-additive side-effects	Mylan 2008	expansion
TCA	Amitriptyline	Yes-serotonin syndrome	Mylan 2009	expansion
TCA	Amoxapine	Yes-serotonin syndrome	Mylan 2009	expansion
TCA	Antiarrhythmic	Yes	Mylan 2007	expansion
TCA	Anticholinergic	Yes	Sandoz 2010b, Duramed 2007	expansion
TCA	Anticholinergic	Yes-Additive side-effects	Mylan 2007	expansion
TCA	Anticholinergic	Yes-Additive side-effects	Sandoz 2010	expansion
TCA	Anticholinergic	Yes-Hyperpyrexia, Paralytic ileus	Mylan 2009	expansion
TCA	Anticholinergic	Yes-Paralytic ileus	Watson 2009	expansion
TCA	Anticholinergic	Yes-additive side-effects	Mylan 2008	expansion
TCA	Antipsychotic	Yes- Neuroleptic Malignant Syndrome	Mylan 2009	expansion
TCA	Aripiprazole	Yes- Neuroleptic Malignant Syndrome	Mylan 2009	expansion
TCA	Asenapine	Yes- Neuroleptic Malignant Syndrome	Mylan 2009	expansion
TCA	Atropine	Yes	Sandoz 2010b, Duramed 2007	expansion
TCA	Atropine	Yes-Additive side-effects	Mylan 2007	expansion
TCA	Atropine	Yes-Additive side-effects	Sandoz 2010	expansion
TCA	Atropine	Yes-Hyperpyrexia, Paralytic ileus	Mylan 2009	expansion
TCA	Atropine	Yes-Paralytic ileus	Watson 2009	expansion
TCA	Atropine	Yes-additive side-effects	Mylan 2008	expansion
TCA	Benzodiazepine	Yes- Neuroleptic Malignant Syndrome	Mylan 2009	expansion
TCA	Benztropine	Yes	Sandoz 2010b, Duramed 2007	expansion
TCA	Benztropine	Yes-Additive side-effects	Mylan 2007	expansion
TCA	Benztropine	Yes-Additive side-effects	Sandoz 2010	expansion

TCA	Benztropine	Yes-Hyperpyrexia, Paralytic ileus	Mylan 2009	expansion
TCA	Benztropine	Yes-Paralytic ileus	Watson 2009	expansion
TCA	Benztropine	Yes-additive side-effects	Mylan 2008	expansion
TCA	Biperiden	Yes	Sandoz 2010b, Duramed 2007	expansion
TCA	Biperiden	Yes-Additive side-effects	Mylan 2007	expansion
TCA	Biperiden	Yes-Additive side-effects	Sandoz 2010	expansion
TCA	Biperiden	Yes-Hyperpyrexia, Paralytic ileus	Mylan 2009	expansion
TCA	Biperiden	Yes-Paralytic ileus	Watson 2009	expansion
TCA	Biperiden	Yes-additive side-effects	Mylan 2008	expansion
TCA	Buspirone	Yes-serotonin syndrome	Mylan 2009	expansion
TCA	CNS depressants	Yes-additive CNS depressant effects	Sandoz 2010b, Duramed 2007	expansion
TCA	CNS depressants	Yes-additive CNS depressant effects	Watson 2009	expansion
TCA	Chlorpromazine	Yes- Neuroleptic Malignant Syndrome	Mylan 2009	expansion
TCA	Chlorpropamide	Yes-hyperglycemia	Mylan 2007	expansion
TCA	Cimetidine	Yes?- anticholinergic symptoms (i.e., severe dry mo	Watson 2007	expansion
TCA	Citalopram	Yes-serotonin syndrome	Mylan 2009	expansion
TCA	Clomipramine	Yes-serotonin syndrome	Mylan 2009	expansion
TCA	Clozapine	Yes- Neuroleptic Malignant Syndrome	Mylan 2009	expansion
TCA	Desipramine	Yes-serotonin syndrome	Mylan 2009	expansion
TCA	Desvenlafaxine	Yes-serotonin syndrome	Mylan 2009	expansion
TCA	Dextromethorphan	Yes-serotonin syndrome	Mylan 2009	expansion
TCA	Diazepam	Yes- Neuroleptic Malignant Syndrome	Mylan 2009	expansion
TCA	Dicyclomine	Yes	Sandoz 2010b, Duramed 2007	expansion
TCA	Dicyclomine	Yes-Additive side-effects	Mylan 2007	expansion
TCA	Dicyclomine	Yes-Additive side-effects	Sandoz 2010	expansion
TCA	Dicyclomine	Yes-Hyperpyrexia, Paralytic ileus	Mylan 2009	expansion
TCA	Dicyclomine	Yes-Paralytic ileus	Watson 2009	expansion
TCA	Dicyclomine	Yes-additive side-effects	Mylan 2008	expansion
TCA	Difenoxin	Yes	Sandoz 2010b, Duramed 2007	expansion
TCA	Difenoxin	Yes-Additive side-effects	Mylan 2007	expansion
TCA	Difenoxin	Yes-Additive side-effects	Sandoz 2010	expansion
TCA	Difenoxin	Yes-Hyperpyrexia, Paralytic ileus	Mylan 2009	expansion
TCA	Difenoxin	Yes-Paralytic ileus	Watson 2009	expansion
TCA	Difenoxin	Yes-additive side-effects	Mylan 2008	expansion
TCA	Diphenoxylate	Yes	Sandoz 2010b, Duramed 2007	expansion
TCA	Diphenoxylate	Yes-Additive side-effects	Mylan 2007	expansion
TCA	Diphenoxylate	Yes-Additive side-effects	Sandoz 2010	expansion
TCA	Diphenoxylate	Yes-Hyperpyrexia, Paralytic ileus	Mylan 2009	expansion
TCA	Diphenoxylate	Yes-Paralytic ileus	Watson 2009	expansion
TCA	Diphenoxylate	Yes-additive side-effects	Mylan 2008	expansion
TCA	Doxepin	Yes-serotonin syndrome	Mylan 2009	expansion
TCA	Duloxetine	Yes-serotonin syndrome	Mylan 2009	expansion
TCA	Escitalopram	Yes-serotonin syndrome	Mylan 2009	expansion
TCA	Eszopiclone	Yes-Additive side-effects	Sandoz 2010	expansion
TCA	Fentanyl	Yes-serotonin syndrome	Mylan 2009	expansion
TCA	Fluoxetine	Yes-serotonin syndrome	Mylan 2009	expansion
TCA	Fluvoxamine	Yes-serotonin syndrome	Mylan 2009	expansion
TCA	Glycopyrronium	Yes	Sandoz 2010b, Duramed 2007	expansion
TCA	Glycopyrronium	Yes-Additive side-effects	Mylan 2007	expansion
TCA	Glycopyrronium	Yes-Additive side-effects	Sandoz 2010	expansion
TCA	Glycopyrronium	Yes-Hyperpyrexia, Paralytic ileus	Mylan 2009	expansion
TCA	Glycopyrronium	Yes-Paralytic ileus	Watson 2009	expansion
TCA	Glycopyrronium	Yes-additive side-effects	Mylan 2008	expansion

TCA	H2 blockers	Yes?- anticholinergic symptoms (i.e., severe dry mo	Watson 2007	expansion
TCA	Haloperidol	Yes- Neuroleptic Malignant Syndrome	Mylan 2009	expansion
TCA	Hyoscyamine	Yes	Sandoz 2010b, Duramed 2007	expansion
TCA	Hyoscyamine	Yes-Additive side-effects	Mylan 2007	expansion
TCA	Hyoscyamine	Yes-Additive side-effects	Sandoz 2010	expansion
TCA	Hyoscyamine	Yes-Hyperpyrexia, Paralytic ileus	Mylan 2009	expansion
TCA	Hyoscyamine	Yes-Paralytic ileus	Watson 2009	expansion
TCA	Hyoscyamine	Yes-additive side-effects	Mylan 2008	expansion
TCA	Iloperidone	Yes- Neuroleptic Malignant Syndrome	Mylan 2009	expansion
TCA	Imipramine	Yes-serotonin syndrome	Mylan 2009	expansion
TCA	Ipratropium cation	Yes	Sandoz 2010b, Duramed 2007	expansion
TCA	Ipratropium cation	Yes-Additive side-effects	Mylan 2007	expansion
TCA	Ipratropium cation	Yes-Additive side-effects	Sandoz 2010	expansion
TCA	Ipratropium cation	Yes-Hyperpyrexia, Paralytic ileus	Mylan 2009	expansion
TCA	Ipratropium cation	Yes-Paralytic ileus	Watson 2009	expansion
TCA	Ipratropium cation	Yes-additive side-effects	Mylan 2008	expansion
TCA	Ipratropium	Yes	Sandoz 2010b, Duramed 2007	expansion
TCA	Ipratropium	Yes-Additive side-effects	Mylan 2007	expansion
TCA	Ipratropium	Yes-Additive side-effects	Sandoz 2010	expansion
TCA	Ipratropium	Yes-Hyperpyrexia, Paralytic ileus	Mylan 2009	expansion
TCA	Ipratropium	Yes-Paralytic ileus	Watson 2009	expansion
TCA	Ipratropium	Yes-additive side-effects	Mylan 2008	expansion
TCA	Isocarboxazid	Yes-serotonin syndrome	Mylan 2009	expansion
TCA	Levodopa	Yes-additive CNS depressant effects	Sandoz 2010b, Duramed 2007	expansion
TCA	Levodopa	Yes-additive CNS depressant effects	Watson 2009	expansion
TCA	Linezolid	Yes-serotonin syndrome	Mylan 2009	expansion
TCA	Lithium	Yes- Neuroleptic Malignant Syndrome	Mylan 2009	expansion
TCA	Lorazepam	Yes- Neuroleptic Malignant Syndrome	Mylan 2009	expansion
TCA	MAOI	Yes-Hyperpyretic crises, severe convulsions, death	Mylan 2009, Sandoz 2010, Watson 2007 (doxepin), Mylan 2007, Sandoz 2010b, Watson 2009, Duramed 2007, Ciba-Gigy 1996	original
TCA	Maprotiline	Yes-serotonin syndrome	Mylan 2009	expansion
TCA	Mepenzolate	Yes	Sandoz 2010b, Duramed 2007	expansion
TCA	Mepenzolate	Yes-Additive side-effects	Mylan 2007	expansion
TCA	Mepenzolate	Yes-Additive side-effects	Sandoz 2010	expansion
TCA	Mepenzolate	Yes-Hyperpyrexia, Paralytic ileus	Mylan 2009	expansion
TCA	Mepenzolate	Yes-Paralytic ileus	Watson 2009	expansion
TCA	Mepenzolate	Yes-additive side-effects	Mylan 2008	expansion
TCA	Meperidine	Yes-serotonin syndrome	Mylan 2009	expansion
TCA	Methscopolamine	Yes	Sandoz 2010b, Duramed 2007	expansion
TCA	Methscopolamine	Yes-Additive side-effects	Mylan 2007	expansion
TCA	Methscopolamine	Yes-Additive side-effects	Sandoz 2010	expansion
TCA	Methscopolamine	Yes-Hyperpyrexia, Paralytic ileus	Mylan 2009	expansion
TCA	Methscopolamine	Yes-Paralytic ileus	Watson 2009	expansion
TCA	Methscopolamine	Yes-additive side-effects	Mylan 2008	expansion
TCA	Methylphenidate	Yes-additive CNS depressant effects	Sandoz 2010b, Duramed 2007	expansion

TCA	Methylphenidate	Yes-additive CNS depressant effects	Watson 2009	expansion
TCA	Milnacipran	Yes-serotonin syndrome	Mylan 2009	expansion
TCA	Modafinil	Yes-Additive side-effects	Mylan 2007	expansion
TCA	Modafinil	Yes-Additive side-effects	Sandoz 2010	expansion
TCA	Modafinil	Yes-additive side-effects	Mylan 2008	expansion
TCA	Nefazodone	Yes-serotonin syndrome	Mylan 2009	expansion
TCA	Nortriptyline	Yes-serotonin syndrome	Mylan 2009	expansion
TCA	Odansetran	Yes-serotonin syndrome	Mylan 2009	expansion
TCA	Olanzapine	Yes- Neuroleptic Malignant Syndrome	Mylan 2009	expansion
TCA	Paliperidone	Yes- Neuroleptic Malignant Syndrome	Mylan 2009	expansion
TCA	Paroxetine	Yes-serotonin syndrome	Mylan 2009	expansion
TCA	Pentazocine	Yes-serotonin syndrome	Mylan 2009	expansion
TCA	Perphenazine	Yes- Neuroleptic Malignant Syndrome	Mylan 2009	expansion
TCA	Phenelzine	Yes-serotonin syndrome	Mylan 2009	expansion
TCA	Phenobarbital	Yes	Sandoz 2010b, Duramed 2007	expansion
TCA	Phenobarbital	Yes-Additive side-effects	Mylan 2007	expansion
TCA	Phenobarbital	Yes-Additive side-effects	Sandoz 2010	expansion
TCA	Phenobarbital	Yes-Hyperpyrexia, Paralytic ileus	Mylan 2009	expansion
TCA	Phenobarbital	Yes-Paralytic ileus	Watson 2009	expansion
TCA	Phenobarbital	Yes-additive side-effects	Mylan 2008	expansion
TCA	Phenylpropanolamine	Yes-Additive side-effects	Mylan 2007	expansion
TCA	Phenylpropanolamine	Yes-Additive side-effects	Sandoz 2010	expansion
TCA	Phenylpropanolamine	Yes-additive side-effects	Mylan 2008	expansion
TCA	Pimozide	Yes- Neuroleptic Malignant Syndrome	Mylan 2009	expansion
TCA	Procyclidine	Yes	Sandoz 2010b, Duramed 2007	expansion
TCA	Procyclidine	Yes-Additive side-effects	Mylan 2007	expansion
TCA	Procyclidine	Yes-Additive side-effects	Sandoz 2010	expansion
TCA	Procyclidine	Yes-Hyperpyrexia, Paralytic ileus	Mylan 2009	expansion
TCA	Procyclidine	Yes-Paralytic ileus	Watson 2009	expansion
TCA	Procyclidine	Yes-additive side-effects	Mylan 2008	expansion
TCA	Propantheline	Yes	Sandoz 2010b, Duramed 2007	expansion
TCA	Propantheline	Yes-Additive side-effects	Mylan 2007	expansion
TCA	Propantheline	Yes-Additive side-effects	Sandoz 2010	expansion
TCA	Propantheline	Yes-Hyperpyrexia, Paralytic ileus	Mylan 2009	expansion
TCA	Propantheline	Yes-Paralytic ileus	Watson 2009	expansion
TCA	Propantheline	Yes-additive side-effects	Mylan 2008	expansion
TCA	Protriptyline	Yes-serotonin syndrome	Mylan 2009	expansion
TCA	Pseudoephedrine	Yes	Sandoz 2010b, Duramed 2007	expansion
TCA	Pseudoephedrine	Yes-Additive side-effects	Mylan 2007	expansion
TCA	Pseudoephedrine	Yes-Additive side-effects	Sandoz 2010	expansion
TCA	Pseudoephedrine	Yes-Hyperpyrexia, Paralytic ileus	Mylan 2009	expansion
TCA	Pseudoephedrine	Yes-Paralytic ileus	Watson 2009	expansion
TCA	Pseudoephedrine	Yes-additive side-effects	Mylan 2008	expansion
TCA	Quetiapine	Yes- Neuroleptic Malignant Syndrome	Mylan 2009	expansion
TCA	Quinidine	Yes	Mylan 2007	expansion
TCA	Rasagiline	Yes-serotonin syndrome	Mylan 2009	expansion
TCA	Reserpine	?	Mylan 2007	expansion
TCA	Risperidone	Yes- Neuroleptic Malignant Syndrome	Mylan 2009	expansion
TCA	SEROTONIN-1B AND SERC	Yes-serotonin syndrome	Mylan 2009	expansion
TCA	SNRI	Yes-serotonin syndrome	Mylan 2009	expansion
TCA	SSRI	Yes-serotonin syndrome	Mylan 2009	expansion
TCA	Scopolamine	Yes	Sandoz 2010b, Duramed 2007	expansion
TCA	Scopolamine	Yes-Additive side-effects	Mylan 2007	expansion
TCA	Scopolamine	Yes-Additive side-effects	Sandoz 2010	expansion

TCA	Scopolamine	Yes-Hyperpyrexia, Paralytic ileus	Mylan 2009	expansion
TCA	Scopolamine	Yes-Paralytic ileus	Watson 2009	expansion
TCA	Scopolamine	Yes-additive side-effects	Mylan 2008	expansion
TCA	Sedative Hypnotics	Yes-Additive side-effects	Sandoz 2010	expansion
TCA	Selegiline	Yes-serotonin syndrome	Mylan 2009	expansion
TCA	Sertraline	Yes-serotonin syndrome	Mylan 2009	expansion
TCA	St Johns wort	Yes-serotonin syndrome	Mylan 2009	expansion
TCA	Sulfonylurea	Yes-hyperglycemia	Mylan 2007	expansion
TCA	Sulfonylurea	Yes-hypoglycemia	Watson 2007	expansion
TCA	Sumatriptan	Yes-serotonin syndrome	Mylan 2009	expansion
TCA	Sympathomimetic	Yes-Additive side-effects	Mylan 2007	expansion
TCA	Sympathomimetic	Yes-Additive side-effects	Sandoz 2010	expansion
TCA	Sympathomimetic	Yes-additive side-effects	Mylan 2008	expansion
TCA	Sympathomimetic	Yes-serotonin syndrome	Mylan 2009	expansion
TCA	TCA	Yes-serotonin syndrome	Mylan 2009	expansion
TCA	Temazepam	Yes- Neuroleptic Malignant Syndrome	Mylan 2009	expansion
TCA	Thioridazine	Yes- Neuroleptic Malignant Syndrome	Mylan 2009	expansion
TCA	Thioridizine	Yes- Neuroleptic Malignant Syndrome	Mylan 2009	expansion
TCA	Thiothixene	Yes- Neuroleptic Malignant Syndrome	Mylan 2009	expansion
TCA	Thyroid medications	Yes-cardiovascular toxicity	Mylan 2008	expansion
TCA	Tiotropium	Yes	Sandoz 2010b, Duramed 2007	expansion
TCA	Tiotropium	Yes-Additive side-effects	Mylan 2007	expansion
TCA	Tiotropium	Yes-Additive side-effects	Sandoz 2010	expansion
TCA	Tiotropium	Yes-Hyperpyrexia, Paralytic ileus	Mylan 2009	expansion
TCA	Tiotropium	Yes-Paralytic ileus	Watson 2009	expansion
TCA	Tiotropium	Yes-additive side-effects	Mylan 2008	expansion
TCA	Tolazamide	Yes-hypoglycemia	Watson 2007	expansion
TCA	Tramadol	Yes-serotonin syndrome	Mylan 2009	expansion
TCA	Tranquilizer	Yes-Additive side-effects	Sandoz 2010	expansion
TCA	Tranylcypromine	Yes-serotonin syndrome	Mylan 2009	expansion
TCA	Trazodone	Yes-serotonin syndrome	Mylan 2009	expansion
TCA	Triazolam	Yes-Additive side-effects	Sandoz 2010	expansion
TCA	Trimipramine	Yes-serotonin syndrome	Mylan 2009	expansion
TCA	Tropicamide	Yes	Sandoz 2010b, Duramed 2007	expansion
TCA	Tropicamide	Yes-Additive side-effects	Mylan 2007	expansion
TCA	Tropicamide	Yes-Additive side-effects	Sandoz 2010	expansion
TCA	Tropicamide	Yes-Hyperpyrexia, Paralytic ileus	Mylan 2009	expansion
TCA	Tropicamide	Yes-Paralytic ileus	Watson 2009	expansion
TCA	Tropicamide	Yes-additive side-effects	Mylan 2008	expansion
TCA	Tryptophan	Yes-Additive side-effects	Mylan 2007	expansion
TCA	Tryptophan	Yes-Additive side-effects	Sandoz 2010	expansion
TCA	Tryptophan	Yes-additive side-effects	Mylan 2008	expansion
TCA	Tryptophan	Yes-serotonin syndrome	Mylan 2009	expansion
TCA	Venlafaxine	Yes-serotonin syndrome	Mylan 2009	expansion
TCA	Vilazodone	Yes-serotonin syndrome	Mylan 2009	expansion
TCA	Zaleplon	Yes-Additive side-effects	Sandoz 2010	expansion
TCA	Ziprasidone	Yes- Neuroleptic Malignant Syndrome	Mylan 2009	expansion
TCA	chlordiazepoxide	Yes- Neuroleptic Malignant Syndrome	Mylan 2009	expansion
TCA	clonazepam	Yes- Neuroleptic Malignant Syndrome	Mylan 2009	expansion
TCA	clorazepic acid	Yes- Neuroleptic Malignant Syndrome	Mylan 2009	expansion
TCA	estazolam	Yes- Neuroleptic Malignant Syndrome	Mylan 2009	expansion
TCA	flurazepam	Yes- Neuroleptic Malignant Syndrome	Mylan 2009	expansion
TCA	midazolam	Yes- Neuroleptic Malignant Syndrome	Mylan 2009	expansion
TCA	oxazepam	Yes- Neuroleptic Malignant Syndrome	Mylan 2009	expansion

Tranylcypromine	Amitriptyline	Yes-serotonin syndrome	Dey 2010, Validus 2007, Parke-Davis 2011, GSK 2010	expansion
Tranylcypromine	Amoxapine	Yes-serotonin syndrome	Dey 2010, Validus 2007, Parke-Davis 2011, GSK 2010	expansion
Tranylcypromine	Anticholinergic	Yes-hypertension	Dey 2010, Validus 2007, Parke-Davis 2011, GSK 2010	expansion
Tranylcypromine	Antipsychotic	Yes-seizure	GSK 2010	original
Tranylcypromine	Aripiprazole	Yes-seizure	GSK 2010	expansion
Tranylcypromine	Asenapine	Yes-seizure	GSK 2010	expansion
Tranylcypromine	Buspirone	Yes-hypertension	Dey 2010, Validus 2007, Parke-Davis 2011, GSK 2010	expansion
Tranylcypromine	CNS depressants	Yes-hypertension	GSK-2010	expansion
Tranylcypromine	Chlorpromazine	Yes-seizure	GSK 2010	expansion
Tranylcypromine	Citalopram	Yes-serotonin syndrome	Dey 2010, Validus 2007, Parke-Davis 2011, GSK 2010	expansion
Tranylcypromine	Clomipramine	Yes-serotonin syndrome	Dey 2010, Validus 2007, Parke-Davis 2011, GSK 2010	expansion
Tranylcypromine	Clozapine	Yes-seizure	GSK 2010	expansion
Tranylcypromine	Desipramine	Yes-serotonin syndrome	Dey 2010, Validus 2007, Parke-Davis 2011, GSK 2010	expansion
Tranylcypromine	Desvenlafaxine	Yes-serotonin syndrome	Dey 2010, Validus 2007, Parke-Davis 2011, GSK 2010	expansion
Tranylcypromine	Dextromethorphan	Yes-serotonin syndrome	Dey 2010, Validus 2007, Parke-Davis 2011, GSK 2010	expansion
Tranylcypromine	Dopamine	Yes-hypertension	GSK-2010	original
Tranylcypromine	Doxepin	Yes-serotonin syndrome	Dey 2010, Validus 2007, Parke-Davis 2011, GSK 2010	expansion
Tranylcypromine	Duloxetine	Yes-serotonin syndrome	Dey 2010, Validus 2007, Parke-Davis 2011, GSK 2010	expansion
Tranylcypromine	Escitalopram	Yes-serotonin syndrome	Dey 2010, Validus 2007, Parke-Davis 2011, GSK 2010	expansion
Tranylcypromine	Fentanyl	Yes-serotonin syndrome	Dey 2010, Validus 2007, Parke-Davis 2011, GSK 2010	expansion

Tranylcypromine	Fluoxetine	Yes-serotonin syndrome	Dey 2010, Validus 2007, Parke-Davis 2011, GSK 2010	expansion
Tranylcypromine	Fluvoxamine	Yes-serotonin syndrome	Dey 2010, Validus 2007, Parke-Davis 2011, GSK 2010	expansion
Tranylcypromine	Guanethidine	Yes-hypertension	GSK-2010	original
Tranylcypromine	Haloperidol	Yes-seizure	GSK 2010	expansion
Tranylcypromine	Iloperidone	Yes-seizure	GSK 2010	expansion
Tranylcypromine	Imipramine	Yes-serotonin syndrome	Dey 2010, Validus 2007, Parke-Davis 2011, GSK 2010	expansion
Tranylcypromine	Isocarboxazid	Yes-serotonin syndrome	Dey 2010, Validus 2007, Parke-Davis 2011, GSK 2010	expansion
Tranylcypromine	Levodopa	Yes-hypertension	GSK-2010	original
Tranylcypromine	Linezolid	Yes-serotonin syndrome	Dey 2010, Validus 2007, Parke-Davis 2011, GSK 2010	expansion
Tranylcypromine	Lithium	Yes-seizure	GSK 2010	expansion
Tranylcypromine	Maprotiline	Yes-serotonin syndrome	Dey 2010, Validus 2007, Parke-Davis 2011, GSK 2010	expansion
Tranylcypromine	Meperidine	Yes- coma, severe hypertension or hypotension, se	Dey 2010, Validus 2007, Parke-Davis 2011, GSK 2010	expansion
Tranylcypromine	Methyldopa	Yes-hypertension	GSK-2010	original
Tranylcypromine	Methylxanthine	Yes-seizure	GSK 2010	expansion
Tranylcypromine	Milnacipran	Yes-serotonin syndrome	Dey 2010, Validus 2007, Parke-Davis 2011, GSK 2010	expansion
Tranylcypromine	Modafinil	Yes-hypertension	Dey 2010, Validus 2007, Parke-Davis 2011, GSK 2010	expansion
Tranylcypromine	Nefazodone	Yes-serotonin syndrome	Dey 2010, Validus 2007, Parke-Davis 2011, GSK 2010	expansion
Tranylcypromine	Nortriptyline	Yes-serotonin syndrome	Dey 2010, Validus 2007, Parke-Davis 2011, GSK 2010	expansion
Tranylcypromine	Odansetran	Yes-serotonin syndrome	Dey 2010, Validus 2007, Parke-Davis 2011, GSK 2010	expansion
Tranylcypromine	Olanzapine	Yes-seizure	GSK 2010	expansion
Tranylcypromine	Paliperidone	Yes-seizure	GSK 2010	expansion

Tranylcypromine	Paroxetine	Yes-serotonin syndrome	Dey 2010, Validus 2007, Parke-Davis 2011, GSK 2010	expansion
Tranylcypromine	Pentazocine	Yes-serotonin syndrome	Dey 2010, Validus 2007, Parke-Davis 2011, GSK 2010	expansion
Tranylcypromine	Perphenazine	Yes-seizure	GSK 2010	expansion
Tranylcypromine	Phenelzine	Yes-serotonin syndrome	Dey 2010, Validus 2007, Parke-Davis 2011, GSK 2010	expansion
Tranylcypromine	Phenylpropanolamine	Yes-hypertension	Dey 2010, Validus 2007, Parke-Davis 2011, GSK 2010	expansion
Tranylcypromine	Pimozide	Yes-seizure	GSK 2010	expansion
Tranylcypromine	Protriptyline	Yes-serotonin syndrome	Dey 2010, Validus 2007, Parke-Davis 2011, GSK 2010	expansion
Tranylcypromine	Pseudoephedrine	Yes-hypertension	Dey 2010, Validus 2007, Parke-Davis 2011, GSK 2010	expansion
Tranylcypromine	Quetiapine	Yes-seizure	GSK 2010	expansion
Tranylcypromine	Rasagiline	Yes-serotonin syndrome	Dey 2010, Validus 2007, Parke-Davis 2011, GSK 2010	expansion
Tranylcypromine	Reserpine	Yes-hypertension	GSK-2010	original
Tranylcypromine	Risperidone	Yes-seizure	GSK 2010	expansion
Tranylcypromine	SEROTONIN-1B AND SERC	Yes-serotonin syndrome	Dey 2010, Validus 2007, Parke-Davis 2011, GSK 2010	expansion
Tranylcypromine	Selegiline	Yes-serotonin syndrome	Dey 2010, Validus 2007, Parke-Davis 2011, GSK 2010	expansion
Tranylcypromine	Sertraline	Yes-serotonin syndrome	Dey 2010, Validus 2007, Parke-Davis 2011, GSK 2010	expansion
Tranylcypromine	St Johns wort	Yes-serotonin syndrome	Dey 2010, Validus 2007, Parke-Davis 2011, GSK 2010	expansion
Tranylcypromine	Sumatriptan	Yes-serotonin syndrome	Dey 2010, Validus 2007, Parke-Davis 2011, GSK 2010	expansion
Tranylcypromine	Sympathomimetic	Yes-hypertension	Dey 2010, Validus 2007, Parke-Davis 2011, GSK 2010	expansion
Tranylcypromine	Sympathomimetic	Yes-hypertension	GSK-2010	expansion

Tranylcypromine	Sympathomimetic	Yes-serotonin syndrome	Dey 2010, Validus 2007, Parke-Davis 2011, GSK 2010	expansion
Tranylcypromine	Theophylline	Yes-seizure	GSK 2010	original
Tranylcypromine	Thioridazine	Yes-seizure	GSK 2010	expansion
Tranylcypromine	Thioridizine	Yes-seizure	GSK 2010	expansion
Tranylcypromine	Thiothixene	Yes-seizure	GSK 2010	expansion
Tranylcypromine	Tramadol	Yes-serotonin syndrome	Dey 2010, Validus 2007, Parke-Davis 2011, GSK 2010	expansion
Tranylcypromine	Tranylcypromine	Yes-serotonin syndrome	Dey 2010, Validus 2007, Parke-Davis 2011, GSK 2010	expansion
Tranylcypromine	Trazodone	Yes-serotonin syndrome	Dey 2010, Validus 2007, Parke-Davis 2011, GSK 2010	expansion
Tranylcypromine	Trimipramine	Yes-serotonin syndrome	Dey 2010, Validus 2007, Parke-Davis 2011, GSK 2010	expansion
Tranylcypromine	Tryptophan	Yes-hypertension	GSK-2010	original
Tranylcypromine	Venlafaxine	Yes-serotonin syndrome	Dey 2010, Validus 2007, Parke-Davis 2011, GSK 2010	expansion
Tranylcypromine	Vilazodone	Yes-serotonin syndrome	Dey 2010, Validus 2007, Parke-Davis 2011, GSK 2010	expansion
Tranylcypromine	Ziprasidone	Yes-seizure	GSK 2010	expansion
Trazodone	Acetaminophen	Yes-GI bleeding	Labopharm 2010	expansion
Trazodone	Acetaminophen	Yes-bleeding	Forest 2009, Forest 2011, Dista 2009, Alphapharm 2007, Watson 2008, Forest 2011B	expansion
Trazodone	Aminoketone	Yes-bleeding	Forest 2009, Forest 2011, Dista 2009, Alphapharm 2007, Watson 2008, Forest 2011B	expansion
Trazodone	Amitriptyline	Yes-serotonin syndrome	Forest 2009, Forest 2011, Dista 2009, Alphapharm 2007, Watson 2008, Forest 2011B (Vilazodone)	expansion

Trazodone	Amitriptyline	Yes-serotonin syndrome	Organon 2010, Rebel 2010, Labopharm 2010	expansion
Trazodone	Amoxapine	Yes-serotonin syndrome	Forest 2009, Forest 2011, Dista 2009, Alphapharm 2007, Watson 2008, Forest 2011B (Vilazodone)	expansion
Trazodone	Amoxapine	Yes-serotonin syndrome	Organon 2010, Rebel 2010, Labopharm 2010	expansion
Trazodone	Antipsychotic	Yes-QTc interval prolongation	Forest 2009, Forest 2011, Dista 2009, Alphapharm 2007, Watson 2008	expansion
Trazodone	Antithrombin alfa	Yes-GI bleeding	Labopharm 2010	expansion
Trazodone	Antithrombin alfa	Yes-bleeding	Forest 2009, Forest 2011, Dista 2009, Alphapharm 2007, Watson 2008, Forest 2011B	expansion
Trazodone	Argatroban	Yes-GI bleeding	Labopharm 2010	expansion
Trazodone	Argatroban	Yes-bleeding	Forest 2009, Forest 2011, Dista 2009, Alphapharm 2007, Watson 2008, Forest 2011B	expansion
Trazodone	Aspirin	Yes-GI bleeding	Labopharm 2010	original
Trazodone	Bivalirudin	Yes-GI bleeding	Labopharm 2010	expansion
Trazodone	Bivalirudin	Yes-bleeding	Forest 2009, Forest 2011, Dista 2009, Alphapharm 2007, Watson 2008, Forest 2011B	expansion
Trazodone	Bromfenac	Yes-GI bleeding	Labopharm 2010	expansion
Trazodone	Bromfenac	Yes-bleeding	Forest 2009, Forest 2011, Dista 2009, Alphapharm 2007, Watson 2008, Forest 2011B	expansion

Trazodone	Bupropion	Yes-bleeding	Forest 2009, Forest 2011, Dista 2009, Alphapharm 2007, Watson 2008, Forest 2011B	expansion
Trazodone	Buspirone	Yes-serotonin syndrome	Organon 2010, Rebel 2010, Labopharm 2010	original
Trazodone	Celecoxib	Yes-GI bleeding	Labopharm 2010	expansion
Trazodone	Celecoxib	Yes-bleeding	Forest 2009, Forest 2011, Dista 2009, Alphapharm 2007, Watson 2008, Forest 2011B	expansion
Trazodone	Citalopram	Yes-serotonin syndrome	Forest 2009, Forest 2011, Dista 2009, Alphapharm 2007, Watson 2008, Forest 2011B (Vilazodone)	expansion
Trazodone	Citalopram	Yes-serotonin syndrome	Organon 2010, Rebel 2010, Labopharm 2010	expansion
Trazodone	Clomipramine	Yes-serotonin syndrome	Forest 2009, Forest 2011, Dista 2009, Alphapharm 2007, Watson 2008, Forest 2011B (Vilazodone)	expansion
Trazodone	Clomipramine	Yes-serotonin syndrome	Organon 2010, Rebel 2010, Labopharm 2010	expansion
Trazodone	Desipramine	Yes-serotonin syndrome	Forest 2009, Forest 2011, Dista 2009, Alphapharm 2007, Watson 2008, Forest 2011B (Vilazodone)	expansion
Trazodone	Desipramine	Yes-serotonin syndrome	Organon 2010, Rebel 2010, Labopharm 2010	expansion
Trazodone	Desirudin	Yes-GI bleeding	Labopharm 2010	expansion
Trazodone	Desirudin	Yes-bleeding	Forest 2009, Forest 2011, Dista 2009, Alphapharm 2007, Watson 2008, Forest 2011B	expansion

Trazodone	Desvenlafaxine	Yes-serotonin syndrome	Forest 2009, Forest 2011, Dista 2009, Alphapharm 2007, Watson 2008, Forest 2011B (Vilazodone)	expansion
Trazodone	Desvenlafaxine	Yes-serotonin syndrome	Organon 2010, Rebel 2010, Labopharm 2010	expansion
Trazodone	Dextromethorphan	Yes-serotonin syndrome	Organon 2010, Rebel 2010, Labopharm 2010	original
Trazodone	Diclofenac	Yes-GI bleeding	Labopharm 2010	expansion
Trazodone	Diclofenac	Yes-bleeding	Forest 2009, Forest 2011, Dista 2009, Alphapharm 2007, Watson 2008, Forest 2011B	expansion
Trazodone	Diflunisal	Yes-GI bleeding	Labopharm 2010	expansion
Trazodone	Diflunisal	Yes-bleeding	Forest 2009, Forest 2011, Dista 2009, Alphapharm 2007, Watson 2008, Forest 2011B	expansion
Trazodone	Doxepin	Yes-serotonin syndrome	Forest 2009, Forest 2011, Dista 2009, Alphapharm 2007, Watson 2008, Forest 2011B (Vilazodone)	expansion
Trazodone	Doxepin	Yes-serotonin syndrome	Organon 2010, Rebel 2010, Labopharm 2010	expansion
Trazodone	Duloxetine	Yes-serotonin syndrome	Forest 2009, Forest 2011, Dista 2009, Alphapharm 2007, Watson 2008, Forest 2011B (Vilazodone)	expansion
Trazodone	Duloxetine	Yes-serotonin syndrome	Organon 2010, Rebel 2010, Labopharm 2010	expansion

Trazodone	Escitalopram	Yes-serotonin syndrome	Forest 2009, Forest 2011, Dista 2009, Alphapharm 2007, Watson 2008, Forest 2011B (Vilazodone)	expansion
Trazodone	Escitalopram	Yes-serotonin syndrome	Organon 2010, Rebel 2010, Labopharm 2010	expansion
Trazodone	Etodolac	Yes-GI bleeding	Labopharm 2010	expansion
Trazodone	Etodolac	Yes-bleeding	Forest 2009, Forest 2011, Dista 2009, Alphapharm 2007, Watson 2008, Forest 2011B	expansion
Trazodone	Fenoprofen	Yes-GI bleeding	Labopharm 2010	expansion
Trazodone	Fenoprofen	Yes-bleeding	Forest 2009, Forest 2011, Dista 2009, Alphapharm 2007, Watson 2008, Forest 2011B	expansion
Trazodone	Fentanyl	Yes-serotonin syndrome	Organon 2010, Rebel 2010, Labopharm 2010	original
Trazodone	Fluoxetine	Yes-serotonin syndrome	Forest 2009, Forest 2011, Dista 2009, Alphapharm 2007, Watson 2008, Forest 2011B (Vilazodone)	expansion
Trazodone	Fluoxetine	Yes-serotonin syndrome	Organon 2010, Rebel 2010, Labopharm 2010	expansion
Trazodone	Flurbiprofen	Yes-GI bleeding	Labopharm 2010	expansion
Trazodone	Flurbiprofen	Yes-bleeding	Forest 2009, Forest 2011, Dista 2009, Alphapharm 2007, Watson 2008, Forest 2011B	expansion
Trazodone	Fluvoxamine	Yes-serotonin syndrome	Forest 2009, Forest 2011, Dista 2009, Alphapharm 2007, Watson 2008, Forest 2011B (Vilazodone)	expansion

Trazodone	Fluvoxamine	Yes-serotonin syndrome	Organon 2010, Rebel 2010, Labopharm 2010	expansion
Trazodone	Heparin	Yes-GI bleeding	Labopharm 2010	original
Trazodone	Ibuprofen	Yes-GI bleeding	Labopharm 2010	expansion
Trazodone	Ibuprofen	Yes-bleeding	Forest 2009, Forest 2011, Dista 2009, Alphapharm 2007, Watson 2008, Forest 2011B	expansion
Trazodone	Imipramine	Yes-serotonin syndrome	Forest 2009, Forest 2011, Dista 2009, Alphapharm 2007, Watson 2008, Forest 2011B (Vilazodone)	expansion
Trazodone	Imipramine	Yes-serotonin syndrome	Organon 2010, Rebel 2010, Labopharm 2010	expansion
Trazodone	Indomethacin	Yes-GI bleeding	Labopharm 2010	expansion
Trazodone	Indomethacin	Yes-bleeding	Forest 2009, Forest 2011, Dista 2009, Alphapharm 2007, Watson 2008, Forest 2011B	expansion
Trazodone	Isocarboxazid	Yes-serotonin syndrome	Forest 2009, Forest 2011, Dista 2009, Alphapharm 2007, Watson 2008, Forest 2011B (Vilazodone)	expansion
Trazodone	Isocarboxazid	Yes-serotonin syndrome	Organon 2010, Rebel 2010, Labopharm 2010	expansion
Trazodone	Ketoprofen	Yes-GI bleeding	Labopharm 2010	expansion
Trazodone	Ketoprofen	Yes-bleeding	Forest 2009, Forest 2011, Dista 2009, Alphapharm 2007, Watson 2008, Forest 2011B	expansion
Trazodone	Ketorolac tromethamine	Yes-GI bleeding	Labopharm 2010	expansion
Trazodone	Ketorolac tromethamine	Yes-bleeding	Forest 2009, Forest 2011, Dista 2009, Alphapharm 2007, Watson 2008, Forest 2011B	expansion
Trazodone	Ketorolac	Yes-GI bleeding	Labopharm 2010	expansion

Trazodone	Ketorolac	Yes-bleeding	Forest 2009, Forest 2011, Dista 2009, Alphapharm 2007, Watson 2008, Forest 2011B	expansion
Trazodone	Lepirudin	Yes-GI bleeding	Labopharm 2010	expansion
Trazodone	Lepirudin	Yes-bleeding	Forest 2009, Forest 2011, Dista 2009, Alphapharm 2007, Watson 2008, Forest 2011B	expansion
Trazodone	Linezolid	Yes-serotonin syndrome	Organon 2010, Rebel 2010, Labopharm 2010	original
Trazodone	MAOI	Yes-serotonin syndrome	Organon 2010, Rebel 2010, Labopharm 2010	original
Trazodone	Mannitol	Yes-GI bleeding	Labopharm 2010	expansion
Trazodone	Mannitol	Yes-bleeding	Forest 2009, Forest 2011, Dista 2009, Alphapharm 2007, Watson 2008, Forest 2011B	expansion
Trazodone	Maprotiline	Yes-serotonin syndrome	Forest 2009, Forest 2011, Dista 2009, Alphapharm 2007, Watson 2008, Forest 2011B (Vilazodone)	expansion
Trazodone	Maprotiline	Yes-serotonin syndrome	Organon 2010, Rebel 2010, Labopharm 2010	expansion
Trazodone	Mefenamic acid	Yes-GI bleeding	Labopharm 2010	expansion
Trazodone	Mefenamic acid	Yes-bleeding	Forest 2009, Forest 2011, Dista 2009, Alphapharm 2007, Watson 2008, Forest 2011B	expansion
Trazodone	Meloxicam	Yes-GI bleeding	Labopharm 2010	expansion
Trazodone	Meloxicam	Yes-bleeding	Forest 2009, Forest 2011, Dista 2009, Alphapharm 2007, Watson 2008, Forest 2011B	expansion
Trazodone	Meperidine	Yes-serotonin syndrome	Organon 2010, Rebel 2010, Labopharm 2010	original

Trazodone	Milnacipran	Yes-serotonin syndrome	Forest 2009, Forest 2011, Dista 2009, Alphapharm 2007, Watson 2008, Forest 2011B (Vilazodone)	expansion
Trazodone	Milnacipran	Yes-serotonin syndrome	Organon 2010, Rebel 2010, Labopharm 2010	expansion
Trazodone	NSAID	Yes-GI bleeding	Labopharm 2010	original
Trazodone	Nabumetone	Yes-GI bleeding	Labopharm 2010	expansion
Trazodone	Nabumetone	Yes-bleeding	Forest 2009, Forest 2011, Dista 2009, Alphapharm 2007, Watson 2008, Forest 2011B	expansion
Trazodone	Naproxen	Yes-GI bleeding	Labopharm 2010	expansion
Trazodone	Naproxen	Yes-bleeding	Forest 2009, Forest 2011, Dista 2009, Alphapharm 2007, Watson 2008, Forest 2011B	expansion
Trazodone	Nefazodone	Yes-serotonin syndrome	Forest 2009, Forest 2011, Dista 2009, Alphapharm 2007, Watson 2008, Forest 2011B (Vilazodone)	expansion
Trazodone	Nefazodone	Yes-serotonin syndrome	Organon 2010, Rebel 2010, Labopharm 2010	expansion
Trazodone	Nepafenac	Yes-GI bleeding	Labopharm 2010	expansion
Trazodone	Nepafenac	Yes-bleeding	Forest 2009, Forest 2011, Dista 2009, Alphapharm 2007, Watson 2008, Forest 2011B	expansion
Trazodone	Nortriptyline	Yes-serotonin syndrome	Forest 2009, Forest 2011, Dista 2009, Alphapharm 2007, Watson 2008, Forest 2011B (Vilazodone)	expansion
Trazodone	Nortriptyline	Yes-serotonin syndrome	Organon 2010, Rebel 2010, Labopharm 2010	expansion

Trazodone	Odansetran	Yes-serotonin syndrome	Organon 2010, Rebel 2010, Labopharm 2010	original
Trazodone	Oxaprozin	Yes-GI bleeding	Labopharm 2010	expansion
Trazodone	Oxaprozin	Yes-bleeding	Forest 2009, Forest 2011, Dista 2009, Alphapharm 2007, Watson 2008, Forest 2011B	expansion
Trazodone	Paroxetine	Yes-serotonin syndrome	Forest 2009, Forest 2011, Dista 2009, Alphapharm 2007, Watson 2008, Forest 2011B (Vilazodone)	expansion
Trazodone	Paroxetine	Yes-serotonin syndrome	Organon 2010, Rebel 2010, Labopharm 2010	expansion
Trazodone	Pentazocine	Yes-serotonin syndrome	Organon 2010, Rebel 2010, Labopharm 2010	original
Trazodone	Phenelzine	Yes-serotonin syndrome	Forest 2009, Forest 2011, Dista 2009, Alphapharm 2007, Watson 2008, Forest 2011B (Vilazodone)	expansion
Trazodone	Phenelzine	Yes-serotonin syndrome	Organon 2010, Rebel 2010, Labopharm 2010	expansion
Trazodone	Pimozide	Yes-QTc interval prolongation	Forest 2009, Forest 2011, Dista 2009, Alphapharm 2007, Watson 2008	expansion
Trazodone	Piroxicam	Yes-GI bleeding	Labopharm 2010	expansion
Trazodone	Piroxicam	Yes-bleeding	Forest 2009, Forest 2011, Dista 2009, Alphapharm 2007, Watson 2008, Forest 2011B	expansion
Trazodone	Protease Inhibitor	Yes	Labopharm 2010	expansion
Trazodone	Protriptyline	Yes-serotonin syndrome	Forest 2009, Forest 2011, Dista 2009, Alphapharm 2007, Watson 2008, Forest 2011B (Vilazodone)	expansion

Trazodone	Protriptyline	Yes-serotonin syndrome	Organon 2010, Rebel 2010, Labopharm 2010	expansion
Trazodone	Rasagiline	Yes-serotonin syndrome	Forest 2009, Forest 2011, Dista 2009, Alphapharm 2007, Watson 2008, Forest 2011B (Vilazodone)	expansion
Trazodone	Rasagiline	Yes-serotonin syndrome	Organon 2010, Rebel 2010, Labopharm 2010	expansion
Trazodone	Ritonavir	Yes	Labopharm 2010	original
Trazodone	SEROTONIN-1B AND SERC	Yes-serotonin syndrome	Forest 2009, Forest 2011, Dista 2009, Alphapharm 2007, Watson 2008, Forest 2011B (Vilazodone)	expansion
Trazodone	SEROTONIN-1B AND SERC	Yes-serotonin syndrome	Organon 2010, Rebel 2010, Labopharm 2010	expansion
Trazodone	SEROTONIN-1B AND SERC	Yes-weakness, hyperreflexia, and incoordination	Forest 2009, Forest 2011, Watson 2008	expansion
Trazodone	SNRI	Yes-serotonin syndrome	Organon 2010, Rebel 2010, Labopharm 2010	original
Trazodone	SSRI	Yes-serotonin syndrome	Organon 2010, Rebel 2010, Labopharm 2010	original
Trazodone	Salicylamide	Yes-GI bleeding	Labopharm 2010	expansion
Trazodone	Salicylamide	Yes-bleeding	Forest 2009, Forest 2011, Dista 2009, Alphapharm 2007, Watson 2008, Forest 2011B	expansion
Trazodone	Selegiline	Yes-serotonin syndrome	Forest 2009, Forest 2011, Dista 2009, Alphapharm 2007, Watson 2008, Forest 2011B (Vilazodone)	expansion
Trazodone	Selegiline	Yes-serotonin syndrome	Organon 2010, Rebel 2010, Labopharm 2010	expansion

Trazodone	Sertraline	Yes-serotonin syndrome	Forest 2009, Forest 2011, Dista 2009, Alphapharm 2007, Watson 2008, Forest 2011B (Vilazodone)	expansion
Trazodone	Sertraline	Yes-serotonin syndrome	Organon 2010, Rebel 2010, Labopharm 2010	expansion
Trazodone	St Johns wort	Yes-serotonin syndrome	Organon 2010, Rebel 2010, Labopharm 2010	original
Trazodone	Sulindac	Yes-GI bleeding	Labopharm 2010	expansion
Trazodone	Sulindac	Yes-bleeding	Forest 2009, Forest 2011, Dista 2009, Alphapharm 2007, Watson 2008, Forest 2011B	expansion
Trazodone	Sumatriptan	Yes-serotonin syndrome	Organon 2010, Rebel 2010, Labopharm 2010	original
Trazodone	Sympathomimetic	Yes-serotonin syndrome	Forest 2009, Forest 2011, Dista 2009, Alphapharm 2007, Watson 2008, Forest 2011B (Vilazodone)	expansion
Trazodone	Sympathomimetic	Yes-serotonin syndrome	Organon 2010, Rebel 2010, Labopharm 2010	expansion
Trazodone	TCA	Yes-serotonin syndrome	Organon 2010, Rebel 2010, Labopharm 2010	original
Trazodone	Thrombin Inhibitors	Yes-GI bleeding	Labopharm 2010	original
Trazodone	Tolmetin	Yes-GI bleeding	Labopharm 2010	expansion
Trazodone	Tolmetin	Yes-bleeding	Forest 2009, Forest 2011, Dista 2009, Alphapharm 2007, Watson 2008, Forest 2011B	expansion
Trazodone	Tramadol	Yes-serotonin syndrome	Organon 2010, Rebel 2010, Labopharm 2010	original
Trazodone	Tranylcypromine	Yes-serotonin syndrome	Forest 2009, Forest 2011, Dista 2009, Alphapharm 2007, Watson 2008, Forest 2011B (Vilazodone)	expansion

Trazodone	Tranylcypromine	Yes-serotonin syndrome	Organon 2010, Rebel 2010, Labopharm 2010	expansion
Trazodone	Trazodone	Yes-serotonin syndrome	Forest 2009, Forest 2011, Dista 2009, Alphapharm 2007, Watson 2008, Forest 2011B (Vilazodone)	expansion
Trazodone	Trazodone	Yes-serotonin syndrome	Organon 2010, Rebel 2010, Labopharm 2010	expansion
Trazodone	Trimipramine	Yes-serotonin syndrome	Forest 2009, Forest 2011, Dista 2009, Alphapharm 2007, Watson 2008, Forest 2011B (Vilazodone)	expansion
Trazodone	Trimipramine	Yes-serotonin syndrome	Organon 2010, Rebel 2010, Labopharm 2010	expansion
Trazodone	Tryptophan	Yes-serotonin syndrome	Organon 2010, Rebel 2010, Labopharm 2010	original
Trazodone	VITAMIN K ANTAGONIST	Yes-GI bleeding	Labopharm 2010	expansion
Trazodone	VITAMIN K ANTAGONIST	Yes-bleeding	Forest 2009, Forest 2011, Dista 2009, Alphapharm 2007, Watson 2008, Forest 2011B	expansion
Trazodone	Venlafaxine	Yes-serotonin syndrome	Forest 2009, Forest 2011, Dista 2009, Alphapharm 2007, Watson 2008, Forest 2011B (Vilazodone)	expansion
Trazodone	Venlafaxine	Yes-serotonin syndrome	Organon 2010, Rebel 2010, Labopharm 2010	expansion
Trazodone	Vilazodone	Yes-serotonin syndrome	Forest 2009, Forest 2011, Dista 2009, Alphapharm 2007, Watson 2008, Forest 2011B (Vilazodone)	expansion
Trazodone	Vilazodone	Yes-serotonin syndrome	Organon 2010, Rebel 2010, Labopharm 2010	expansion
Trazodone	Warfarin	Yes-GI bleeding	Labopharm 2010	original

Trimipramine	4-hydroxybutanoic acid	Yes-additive CNS depressant effects	Sandoz 2010b, Duramed 2007	expansion
Trimipramine	Amantadine	Yes	Sandoz 2010b, Duramed 2007	expansion
Trimipramine	Anticholinergic	Yes	Sandoz 2010b, Duramed 2007	original
Trimipramine	Atropine	Yes	Sandoz 2010b, Duramed 2007	expansion
Trimipramine	Benztropine	Yes	Sandoz 2010b, Duramed 2007	expansion
Trimipramine	Biperiden	Yes	Sandoz 2010b, Duramed 2007	expansion
Trimipramine	CNS depressants	Yes-additive CNS depressant effects	Sandoz 2010b, Duramed 2007	original
Trimipramine	Dicyclomine	Yes	Sandoz 2010b, Duramed 2007	expansion
Trimipramine	Difenoxin	Yes	Sandoz 2010b, Duramed 2007	expansion
Trimipramine	Diphenoxylate	Yes	Sandoz 2010b, Duramed 2007	expansion
Trimipramine	Glycopyrronium	Yes	Sandoz 2010b, Duramed 2007	expansion
Trimipramine	Hyoscyamine	Yes	Sandoz 2010b, Duramed 2007	expansion
Trimipramine	Ipratropium cation	Yes	Sandoz 2010b, Duramed 2007	expansion
Trimipramine	Ipratropium	Yes	Sandoz 2010b, Duramed 2007	expansion
Trimipramine	Isocarboxazid	Yes-Hyperpyretic crises, severe convulsions, death	Mylan 2009, Sandoz 2010, Watson 2007 (doxepin), Mylan 2007, Sandoz 2010b, Watson 2009, Duramed 2007, Ciba-Gigy 1996	expansion
Trimipramine	Levodopa	Yes-additive CNS depressant effects	Sandoz 2010b, Duramed 2007	expansion
Trimipramine	Mepenzolate	Yes	Sandoz 2010b, Duramed 2007	expansion
Trimipramine	Methscopolamine	Yes	Sandoz 2010b, Duramed 2007	expansion
Trimipramine	Methylphenidate	Yes-additive CNS depressant effects	Sandoz 2010b, Duramed 2007	expansion
Trimipramine	Phenelzine	Yes-Hyperpyretic crises, severe convulsions, death	Mylan 2009, Sandoz 2010, Watson 2007 (doxepin), Mylan 2007, Sandoz 2010b, Watson 2009, Duramed 2007, Ciba-Gigy 1996	expansion
Trimipramine	Phenobarbital	Yes	Sandoz 2010b, Duramed 2007	expansion
Trimipramine	Procyclidine	Yes	Sandoz 2010b, Duramed 2007	expansion

Trimipramine	Propantheline	Yes	Sandoz 2010b, Duramed 2007	expansion
Trimipramine	Pseudoephedrine	Yes	Sandoz 2010b, Duramed 2007	expansion
Trimipramine	Rasagiline	Yes-Hyperpyretic crises, severe convulsions, death	Mylan 2009, Sandoz 2010, Watson 2007 (doxepin), Mylan 2007, Sandoz 2010b, Watson 2009, Duramed 2007, Ciba-Gigy 1996	expansion
Trimipramine	Scopolamine	Yes	Sandoz 2010b, Duramed 2007	expansion
Trimipramine	Selegiline	Yes-Hyperpyretic crises, severe convulsions, death	Mylan 2009, Sandoz 2010, Watson 2007 (doxepin), Mylan 2007, Sandoz 2010b, Watson 2009, Duramed 2007, Ciba-Gigy 1996	expansion
Trimipramine	Tiotropium	Yes	Sandoz 2010b, Duramed 2007	expansion
Trimipramine	Tranylcypromine	Yes-Hyperpyretic crises, severe convulsions, death	Mylan 2009, Sandoz 2010, Watson 2007 (doxepin), Mylan 2007, Sandoz 2010b, Watson 2009, Duramed 2007, Ciba-Gigy 1996	expansion
Trimipramine	Tropicamide	Yes	Sandoz 2010b, Duramed 2007	expansion
Venlafaxine	Acetaminophen	Yes-GI bleeding	Eli-Lilly 2010, Rebel 2010b, Wyeth 2011	expansion
Venlafaxine	Amitriptyline	Yes-serotonin syndrome	Eli-Lilly 2010, Rebel 2010b, Wyeth 2011	expansion
Venlafaxine	Amoxapine	Yes-serotonin syndrome	Eli-Lilly 2010, Rebel 2010b, Wyeth 2011	expansion
Venlafaxine	Antipsychotic	No	Rebel 2010b	expansion
Venlafaxine	Antipsychotic	Yes-seizure	Rebel 2010b	expansion
Venlafaxine	Antithrombin alfa	Yes-GI bleeding	Eli-Lilly 2010, Rebel 2010b, Wyeth 2011	expansion
Venlafaxine	Argatroban	Yes-GI bleeding	Eli-Lilly 2010, Rebel 2010b, Wyeth 2011	expansion
Venlafaxine	Aspirin	Yes-GI bleeding	Eli-Lilly 2010, Rebel 2010b, Wyeth 2011	expansion
Venlafaxine	Azole Antifungal	Yes	Rebel 2010b	expansion
Venlafaxine	Benzodiazepine	No	Rebel 2010b	expansion

Venlafaxine	Beta-adrenergic blocker	No	Rebel 2010b	expansion
Venlafaxine	Beta-adrenergic blocker	Yes-hypertension	Rebel 2010b	expansion
Venlafaxine	Bivalirudin	Yes-GI bleeding	Eli-Lilly 2010, Rebel 2010b, Wyeth 2011	expansion
Venlafaxine	Bromfenac	Yes-GI bleeding	Eli-Lilly 2010, Rebel 2010b, Wyeth 2011	expansion
Venlafaxine	Buspirone	Yes-serotonin syndrome	Eli-Lilly 2010, Rebel 2010b, Wyeth 2011	expansion
Venlafaxine	Celecoxib	Yes-GI bleeding	Eli-Lilly 2010, Rebel 2010b, Wyeth 2011	expansion
Venlafaxine	Cimetidine	Yes	Rebel 2010b	original
Venlafaxine	Citalopram	Yes-serotonin syndrome	Eli-Lilly 2010, Rebel 2010b, Wyeth 2011	expansion
Venlafaxine	Clomipramine	Yes-serotonin syndrome	Eli-Lilly 2010, Rebel 2010b, Wyeth 2011	expansion
Venlafaxine	Clozapine	Yes-seizure	Rebel 2010b	original
Venlafaxine	Desipramine	Yes-serotonin syndrome	Eli-Lilly 2010, Rebel 2010b, Wyeth 2011	expansion
Venlafaxine	Desirudin	Yes-GI bleeding	Eli-Lilly 2010, Rebel 2010b, Wyeth 2011	expansion
Venlafaxine	Desvenlafaxine	Yes-serotonin syndrome	Eli-Lilly 2010, Rebel 2010b, Wyeth 2011	expansion
Venlafaxine	Dextromethorphan	Yes-serotonin syndrome	Eli-Lilly 2010, Rebel 2010b, Wyeth 2011	expansion
Venlafaxine	Diazepam	No	Rebel 2010b	original
Venlafaxine	Diclofenac	Yes-GI bleeding	Eli-Lilly 2010, Rebel 2010b, Wyeth 2011	expansion
Venlafaxine	Diflunisal	Yes-GI bleeding	Eli-Lilly 2010, Rebel 2010b, Wyeth 2011	expansion
Venlafaxine	Doxepin	Yes-serotonin syndrome	Eli-Lilly 2010, Rebel 2010b, Wyeth 2011	expansion
Venlafaxine	Duloxetine	Yes-serotonin syndrome	Eli-Lilly 2010, Rebel 2010b, Wyeth 2011	expansion
Venlafaxine	Escitalopram	Yes-serotonin syndrome	Eli-Lilly 2010, Rebel 2010b, Wyeth 2011	expansion
Venlafaxine	Etodolac	Yes-GI bleeding	Eli-Lilly 2010, Rebel 2010b, Wyeth 2011	expansion
Venlafaxine	Fenoprofen	Yes-GI bleeding	Eli-Lilly 2010, Rebel 2010b, Wyeth 2011	expansion
Venlafaxine	Fentanyl	Yes-serotonin syndrome	Eli-Lilly 2010, Rebel 2010b, Wyeth 2011	expansion

Venlafaxine	Fluoxetine	Yes-serotonin syndrome	Eli-Lilly 2010, Rebel 2010b, Wyeth 2011	expansion
Venlafaxine	Flurbiprofen	Yes-GI bleeding	Eli-Lilly 2010, Rebel 2010b, Wyeth 2011	expansion
Venlafaxine	Fluvoxamine	Yes-serotonin syndrome	Eli-Lilly 2010, Rebel 2010b, Wyeth 2011	expansion
Venlafaxine	H2 blockers	Yes	Rebel 2010b	expansion
Venlafaxine	Heparin	Yes-GI bleeding	Eli-Lilly 2010, Rebel 2010b, Wyeth 2011	expansion
Venlafaxine	Ibuprofen	Yes-GI bleeding	Eli-Lilly 2010, Rebel 2010b, Wyeth 2011	expansion
Venlafaxine	Imipramine	No	Rebel 2010b	original
Venlafaxine	Imipramine	Yes-serotonin syndrome	Eli-Lilly 2010, Rebel 2010b, Wyeth 2011	expansion
Venlafaxine	Indinavir	No	Rebel 2010b	original
Venlafaxine	Indomethacin	Yes-GI bleeding	Eli-Lilly 2010, Rebel 2010b, Wyeth 2011	expansion
Venlafaxine	Isocarboxazid	Yes-serotonin syndrome	Eli-Lilly 2010, Rebel 2010b, Wyeth 2011	expansion
Venlafaxine	Ketoconazole	Yes	Rebel 2010b	original
Venlafaxine	Ketoprofen	Yes-GI bleeding	Eli-Lilly 2010, Rebel 2010b, Wyeth 2011	expansion
Venlafaxine	Ketorolac tromethamine	Yes-GI bleeding	Eli-Lilly 2010, Rebel 2010b, Wyeth 2011	expansion
Venlafaxine	Ketorolac	Yes-GI bleeding	Eli-Lilly 2010, Rebel 2010b, Wyeth 2011	expansion
Venlafaxine	Lepirudin	Yes-GI bleeding	Eli-Lilly 2010, Rebel 2010b, Wyeth 2011	expansion
Venlafaxine	Linezolid	Yes-serotonin syndrome	Eli-Lilly 2010, Rebel 2010b, Wyeth 2011	expansion
Venlafaxine	Lithium	No	Rebel 2010b	original
Venlafaxine	Mannitol	Yes-GI bleeding	Eli-Lilly 2010, Rebel 2010b, Wyeth 2011	expansion
Venlafaxine	Maprotiline	Yes-serotonin syndrome	Eli-Lilly 2010, Rebel 2010b, Wyeth 2011	expansion
Venlafaxine	Mefenamic acid	Yes-GI bleeding	Eli-Lilly 2010, Rebel 2010b, Wyeth 2011	expansion
Venlafaxine	Meloxicam	Yes-GI bleeding	Eli-Lilly 2010, Rebel 2010b, Wyeth 2011	expansion
Venlafaxine	Meperidine	Yes-serotonin syndrome	Eli-Lilly 2010, Rebel 2010b, Wyeth 2011	expansion

Venlafaxine	Metoprolol	No	Rebel 2010b	original
Venlafaxine	Metoprolol	Yes-hypertension	Rebel 2010b	original
Venlafaxine	Milnacipran	Yes-serotonin syndrome	Eli-Lilly 2010, Rebel 2010b, Wyeth 2011	expansion
Venlafaxine	NSAID	Yes-GI bleeding	Eli-Lilly 2010, Rebel 2010b, Wyeth 2011	expansion
Venlafaxine	Nabumetone	Yes-GI bleeding	Eli-Lilly 2010, Rebel 2010b, Wyeth 2011	expansion
Venlafaxine	Naproxen	Yes-GI bleeding	Eli-Lilly 2010, Rebel 2010b, Wyeth 2011	expansion
Venlafaxine	Nefazodone	Yes-serotonin syndrome	Eli-Lilly 2010, Rebel 2010b, Wyeth 2011	expansion
Venlafaxine	Nepafenac	Yes-GI bleeding	Eli-Lilly 2010, Rebel 2010b, Wyeth 2011	expansion
Venlafaxine	Nortriptyline	Yes-serotonin syndrome	Eli-Lilly 2010, Rebel 2010b, Wyeth 2011	expansion
Venlafaxine	Odansetran	Yes-serotonin syndrome	Eli-Lilly 2010, Rebel 2010b, Wyeth 2011	expansion
Venlafaxine	Oxaprozin	Yes-GI bleeding	Eli-Lilly 2010, Rebel 2010b, Wyeth 2011	expansion
Venlafaxine	Paroxetine	Yes-serotonin syndrome	Eli-Lilly 2010, Rebel 2010b, Wyeth 2011	expansion
Venlafaxine	Pentazocine	Yes-serotonin syndrome	Eli-Lilly 2010, Rebel 2010b, Wyeth 2011	expansion
Venlafaxine	Phenelzine	Yes-serotonin syndrome	Eli-Lilly 2010, Rebel 2010b, Wyeth 2011	expansion
Venlafaxine	Piroxicam	Yes-GI bleeding	Eli-Lilly 2010, Rebel 2010b, Wyeth 2011	expansion
Venlafaxine	Protease Inhibitor	No	Rebel 2010b	expansion
Venlafaxine	Protriptyline	Yes-serotonin syndrome	Eli-Lilly 2010, Rebel 2010b, Wyeth 2011	expansion
Venlafaxine	Rasagiline	Yes-serotonin syndrome	Eli-Lilly 2010, Rebel 2010b, Wyeth 2011	expansion
Venlafaxine	SEROTONIN-1B AND SERC	Yes-serotonin syndrome	Eli-Lilly 2010, Rebel 2010b, Wyeth 2011	expansion
Venlafaxine	Salicylamide	Yes-GI bleeding	Eli-Lilly 2010, Rebel 2010b, Wyeth 2011	expansion
Venlafaxine	Selegiline	Yes-serotonin syndrome	Eli-Lilly 2010, Rebel 2010b, Wyeth 2011	expansion

Venlafaxine	Sertraline	Yes-serotonin syndrome	Eli-Lilly 2010, Rebel 2010b, Wyeth 2011	expansion
Venlafaxine	St Johns wort	Yes-serotonin syndrome	Eli-Lilly 2010, Rebel 2010b, Wyeth 2011	expansion
Venlafaxine	Sulindac	Yes-GI bleeding	Eli-Lilly 2010, Rebel 2010b, Wyeth 2011	expansion
Venlafaxine	Sumatriptan	Yes-serotonin syndrome	Eli-Lilly 2010, Rebel 2010b, Wyeth 2011	expansion
Venlafaxine	Sympathomimetic	Yes-serotonin syndrome	Eli-Lilly 2010, Rebel 2010b, Wyeth 2011	expansion
Venlafaxine	TCA	No	Rebel 2010b	expansion
Venlafaxine	Tolmetin	Yes-GI bleeding	Eli-Lilly 2010, Rebel 2010b, Wyeth 2011	expansion
Venlafaxine	Tramadol	Yes-serotonin syndrome	Eli-Lilly 2010, Rebel 2010b, Wyeth 2011	expansion
Venlafaxine	Tranylcypromine	Yes-serotonin syndrome	Eli-Lilly 2010, Rebel 2010b, Wyeth 2011	expansion
Venlafaxine	Trazodone	Yes-serotonin syndrome	Eli-Lilly 2010, Rebel 2010b, Wyeth 2011	expansion
Venlafaxine	Trimipramine	Yes-serotonin syndrome	Eli-Lilly 2010, Rebel 2010b, Wyeth 2011	expansion
Venlafaxine	Tryptophan	Yes-serotonin syndrome	Eli-Lilly 2010, Rebel 2010b, Wyeth 2011	expansion
Venlafaxine	VITAMIN K ANTAGONIST	Yes-GI bleeding	Eli-Lilly 2010, Rebel 2010b, Wyeth 2011	expansion
Venlafaxine	Venlafaxine	Yes-serotonin syndrome	Eli-Lilly 2010, Rebel 2010b, Wyeth 2011	expansion
Venlafaxine	Vilazodone	Yes-serotonin syndrome	Eli-Lilly 2010, Rebel 2010b, Wyeth 2011	expansion
Venlafaxine	Warfarin	Yes-GI bleeding	Eli-Lilly 2010, Rebel 2010b, Wyeth 2011	expansion
Vilazodone	Acetaminophen	Yes-bleeding	Forest 2009, Forest 2011, Dista 2009, Alphapharm 2007, Watson 2008, Forest 2011B	expansion

Vilazodone	Aminoketone	Yes-bleeding	Forest 2009, Forest 2011, Dista 2009, Alphapharm 2007, Watson 2008, Forest 2011B	expansion
Vilazodone	Amitriptyline	Yes-serotonin syndrome	Forest 2009, Forest 2011, Dista 2009, Alphapharm 2007, Watson 2008, Forest 2011B (Vilazodone)	expansion
Vilazodone	Amoxapine	Yes-serotonin syndrome	Forest 2009, Forest 2011, Dista 2009, Alphapharm 2007, Watson 2008, Forest 2011B (Vilazodone)	expansion
Vilazodone	Antipsychotic	Yes-QTc interval prolongation	Forest 2009, Forest 2011, Dista 2009, Alphapharm 2007, Watson 2008	expansion
Vilazodone	Antithrombin alfa	Yes-bleeding	Forest 2009, Forest 2011, Dista 2009, Alphapharm 2007, Watson 2008, Forest 2011B	expansion
Vilazodone	Argatroban	Yes-bleeding	Forest 2009, Forest 2011, Dista 2009, Alphapharm 2007, Watson 2008, Forest 2011B	expansion
Vilazodone	Aspirin	Yes-bleeding	Forest 2009, Forest 2011, Dista 2009, Alphapharm 2007, Watson 2008, Forest 2011B	expansion
Vilazodone	Azole Antifungal	Yes	Forest 2011B	expansion
Vilazodone	Bivalirudin	Yes-bleeding	Forest 2009, Forest 2011, Dista 2009, Alphapharm 2007, Watson 2008, Forest 2011B	expansion

Vilazodone	Bromfenac	Yes-bleeding	Forest 2009, Forest 2011, Dista 2009, Alphapharm 2007, Watson 2008, Forest 2011B	expansion
Vilazodone	Bupropion	Yes-bleeding	Forest 2009, Forest 2011, Dista 2009, Alphapharm 2007, Watson 2008, Forest 2011B	expansion
Vilazodone	Buspirone	Yes-serotonin syndrome	Forest 2009, Forest 2011, Dista 2009, Alphapharm 2007, Watson 2008, Forest 2011B (Vilazodone)	expansion
Vilazodone	Celecoxib	Yes-bleeding	Forest 2009, Forest 2011, Dista 2009, Alphapharm 2007, Watson 2008, Forest 2011B	expansion
Vilazodone	Citalopram	Yes-serotonin syndrome	Forest 2009, Forest 2011, Dista 2009, Alphapharm 2007, Watson 2008, Forest 2011B (Vilazodone)	expansion
Vilazodone	Clomipramine	Yes-serotonin syndrome	Forest 2009, Forest 2011, Dista 2009, Alphapharm 2007, Watson 2008, Forest 2011B (Vilazodone)	expansion
Vilazodone	Desipramine	Yes-serotonin syndrome	Forest 2009, Forest 2011, Dista 2009, Alphapharm 2007, Watson 2008, Forest 2011B (Vilazodone)	expansion

Vilazodone	Desirudin	Yes-bleeding	Forest 2009, Forest 2011, Dista 2009, Alphapharm 2007, Watson 2008, Forest 2011B	expansion
Vilazodone	Desvenlafaxine	Yes-serotonin syndrome	Forest 2009, Forest 2011, Dista 2009, Alphapharm 2007, Watson 2008, Forest 2011B (Vilazodone)	expansion
Vilazodone	Dextromethorphan	Yes-serotonin syndrome	Forest 2009, Forest 2011, Dista 2009, Alphapharm 2007, Watson 2008, Forest 2011B (Vilazodone)	expansion
Vilazodone	Diclofenac	Yes-bleeding	Forest 2009, Forest 2011, Dista 2009, Alphapharm 2007, Watson 2008, Forest 2011B	expansion
Vilazodone	Diflunisal	Yes-bleeding	Forest 2009, Forest 2011, Dista 2009, Alphapharm 2007, Watson 2008, Forest 2011B	expansion
Vilazodone	Doxepin	Yes-serotonin syndrome	Forest 2009, Forest 2011, Dista 2009, Alphapharm 2007, Watson 2008, Forest 2011B (Vilazodone)	expansion
Vilazodone	Duloxetine	Yes-serotonin syndrome	Forest 2009, Forest 2011, Dista 2009, Alphapharm 2007, Watson 2008, Forest 2011B (Vilazodone)	expansion

Vilazodone	Escitalopram	Yes-serotonin syndrome	Forest 2009, Forest 2011, Dista 2009, Alphapharm 2007, Watson 2008, Forest 2011B (Vilazodone)	expansion
Vilazodone	Etodolac	Yes-bleeding	Forest 2009, Forest 2011, Dista 2009, Alphapharm 2007, Watson 2008, Forest 2011B	expansion
Vilazodone	Fenoprofen	Yes-bleeding	Forest 2009, Forest 2011, Dista 2009, Alphapharm 2007, Watson 2008, Forest 2011B	expansion
Vilazodone	Fentanyl	Yes-serotonin syndrome	Forest 2009, Forest 2011, Dista 2009, Alphapharm 2007, Watson 2008, Forest 2011B (Vilazodone)	expansion
Vilazodone	Fluoxetine	Yes-serotonin syndrome	Forest 2009, Forest 2011, Dista 2009, Alphapharm 2007, Watson 2008, Forest 2011B (Vilazodone)	expansion
Vilazodone	Flurbiprofen	Yes-bleeding	Forest 2009, Forest 2011, Dista 2009, Alphapharm 2007, Watson 2008, Forest 2011B	expansion
Vilazodone	Fluvoxamine	Yes-serotonin syndrome	Forest 2009, Forest 2011, Dista 2009, Alphapharm 2007, Watson 2008, Forest 2011B (Vilazodone)	expansion

Vilazodone	Heparin	Yes-bleeding	Forest 2009, Forest 2011, Dista 2009, Alphapharm 2007, Watson 2008, Forest 2011B	expansion
Vilazodone	Ibuprofen	Yes-bleeding	Forest 2009, Forest 2011, Dista 2009, Alphapharm 2007, Watson 2008, Forest 2011B	expansion
Vilazodone	Imipramine	Yes-serotonin syndrome	Forest 2009, Forest 2011, Dista 2009, Alphapharm 2007, Watson 2008, Forest 2011B (Vilazodone)	expansion
Vilazodone	Indomethacin	Yes-bleeding	Forest 2009, Forest 2011, Dista 2009, Alphapharm 2007, Watson 2008, Forest 2011B	expansion
Vilazodone	Isocarboxazid	Yes-serotonin syndrome	Forest 2009, Forest 2011, Dista 2009, Alphapharm 2007, Watson 2008, Forest 2011B (Vilazodone)	expansion
Vilazodone	Ketoconazole	Yes	Forest 2011B	original
Vilazodone	Ketoprofen	Yes-bleeding	Forest 2009, Forest 2011, Dista 2009, Alphapharm 2007, Watson 2008, Forest 2011B	expansion
Vilazodone	Ketorolac tromethamine	Yes-bleeding	Forest 2009, Forest 2011, Dista 2009, Alphapharm 2007, Watson 2008, Forest 2011B	expansion
Vilazodone	Ketorolac	Yes-bleeding	Forest 2009, Forest 2011, Dista 2009, Alphapharm 2007, Watson 2008, Forest 2011B	expansion

Vilazodone	Lepirudin	Yes-bleeding	Forest 2009, Forest 2011, Dista 2009, Alphapharm 2007, Watson 2008, Forest 2011B	expansion
Vilazodone	Linezolid	Yes-serotonin syndrome	Forest 2009, Forest 2011, Dista 2009, Alphapharm 2007, Watson 2008, Forest 2011B (Vilazodone)	expansion
Vilazodone	Mannitol	Yes-bleeding	Forest 2009, Forest 2011, Dista 2009, Alphapharm 2007, Watson 2008, Forest 2011B	expansion
Vilazodone	Maprotiline	Yes-serotonin syndrome	Forest 2009, Forest 2011, Dista 2009, Alphapharm 2007, Watson 2008, Forest 2011B (Vilazodone)	expansion
Vilazodone	Mefenamic acid	Yes-bleeding	Forest 2009, Forest 2011, Dista 2009, Alphapharm 2007, Watson 2008, Forest 2011B	expansion
Vilazodone	Meloxicam	Yes-bleeding	Forest 2009, Forest 2011, Dista 2009, Alphapharm 2007, Watson 2008, Forest 2011B	expansion
Vilazodone	Meperidine	Yes-serotonin syndrome	Forest 2009, Forest 2011, Dista 2009, Alphapharm 2007, Watson 2008, Forest 2011B (Vilazodone)	expansion

Vilazodone	Milnacipran	Yes-serotonin syndrome	Forest 2009, Forest 2011, Dista 2009, Alphapharm 2007, Watson 2008, Forest 2011B (Vilazodone)	expansion
Vilazodone	NSAID	Yes-bleeding	Forest 2009, Forest 2011, Dista 2009, Alphapharm 2007, Watson 2008, Forest 2011B	expansion
Vilazodone	Nabumetone	Yes-bleeding	Forest 2009, Forest 2011, Dista 2009, Alphapharm 2007, Watson 2008, Forest 2011B	expansion
Vilazodone	Naproxen	Yes-bleeding	Forest 2009, Forest 2011, Dista 2009, Alphapharm 2007, Watson 2008, Forest 2011B	expansion
Vilazodone	Nefazodone	Yes-serotonin syndrome	Forest 2009, Forest 2011, Dista 2009, Alphapharm 2007, Watson 2008, Forest 2011B (Vilazodone)	expansion
Vilazodone	Nepafenac	Yes-bleeding	Forest 2009, Forest 2011, Dista 2009, Alphapharm 2007, Watson 2008, Forest 2011B	expansion
Vilazodone	Nortriptyline	Yes-serotonin syndrome	Forest 2009, Forest 2011, Dista 2009, Alphapharm 2007, Watson 2008, Forest 2011B (Vilazodone)	expansion

Vilazodone	Odansetran	Yes-serotonin syndrome	Forest 2009, Forest 2011, Dista 2009, Alphapharm 2007, Watson 2008, Forest 2011B (Vilazodone)	expansion
Vilazodone	Oxaprozin	Yes-bleeding	Forest 2009, Forest 2011, Dista 2009, Alphapharm 2007, Watson 2008, Forest 2011B	expansion
Vilazodone	Paroxetine	Yes-serotonin syndrome	Forest 2009, Forest 2011, Dista 2009, Alphapharm 2007, Watson 2008, Forest 2011B (Vilazodone)	expansion
Vilazodone	Pentazocine	Yes-serotonin syndrome	Forest 2009, Forest 2011, Dista 2009, Alphapharm 2007, Watson 2008, Forest 2011B (Vilazodone)	expansion
Vilazodone	Phenelzine	Yes-serotonin syndrome	Forest 2009, Forest 2011, Dista 2009, Alphapharm 2007, Watson 2008, Forest 2011B (Vilazodone)	expansion
Vilazodone	Pimozide	Yes-QTc interval prolongation	Forest 2009, Forest 2011, Dista 2009, Alphapharm 2007, Watson 2008	expansion
Vilazodone	Piroxicam	Yes-bleeding	Forest 2009, Forest 2011, Dista 2009, Alphapharm 2007, Watson 2008, Forest 2011B	expansion

Vilazodone	Protriptyline	Yes-serotonin syndrome	Forest 2009, Forest 2011, Dista 2009, Alphapharm 2007, Watson 2008, Forest 2011B (Vilazodone)	expansion
Vilazodone	Rasagiline	Yes-serotonin syndrome	Forest 2009, Forest 2011, Dista 2009, Alphapharm 2007, Watson 2008, Forest 2011B (Vilazodone)	expansion
Vilazodone	SEROTONIN-1B AND SERC	Yes-serotonin syndrome	Forest 2009, Forest 2011, Dista 2009, Alphapharm 2007, Watson 2008, Forest 2011B (Vilazodone)	expansion
Vilazodone	SEROTONIN-1B AND SERC	Yes-weakness, hyperreflexia, and incoordination	Forest 2009, Forest 2011, Watson 2008	expansion
Vilazodone	Salicylamide	Yes-bleeding	Forest 2009, Forest 2011, Dista 2009, Alphapharm 2007, Watson 2008, Forest 2011B	expansion
Vilazodone	Selegiline	Yes-serotonin syndrome	Forest 2009, Forest 2011, Dista 2009, Alphapharm 2007, Watson 2008, Forest 2011B (Vilazodone)	expansion
Vilazodone	Sertraline	Yes-serotonin syndrome	Forest 2009, Forest 2011, Dista 2009, Alphapharm 2007, Watson 2008, Forest 2011B (Vilazodone)	expansion
Vilazodone	St Johns wort	Yes-serotonin syndrome	Forest 2009, Forest 2011, Dista 2009, Alphapharm 2007, Watson 2008, Forest 2011B (Vilazodone)	expansion

Vilazodone	Sulindac	Yes-bleeding	Forest 2009, Forest 2011, Dista 2009, Alphapharm 2007, Watson 2008, Forest 2011B	expansion
Vilazodone	Sumatriptan	Yes-serotonin syndrome	Forest 2009, Forest 2011, Dista 2009, Alphapharm 2007, Watson 2008, Forest 2011B (Vilazodone)	expansion
Vilazodone	Sumatriptan	Yes-weakness, hyperreflexia, and incoordination	Forest 2009, Forest 2011, Watson 2008	expansion
Vilazodone	Sympathomimetic	Yes-serotonin syndrome	Forest 2009, Forest 2011, Dista 2009, Alphapharm 2007, Watson 2008, Forest 2011B (Vilazodone)	expansion
Vilazodone	Tolmetin	Yes-bleeding	Forest 2009, Forest 2011, Dista 2009, Alphapharm 2007, Watson 2008, Forest 2011B	expansion
Vilazodone	Tramadol	Yes-serotonin syndrome	Forest 2009, Forest 2011, Dista 2009, Alphapharm 2007, Watson 2008, Forest 2011B (Vilazodone)	expansion
Vilazodone	Tranylcypromine	Yes-serotonin syndrome	Forest 2009, Forest 2011, Dista 2009, Alphapharm 2007, Watson 2008, Forest 2011B (Vilazodone)	expansion
Vilazodone	Trazodone	Yes-serotonin syndrome	Forest 2009, Forest 2011, Dista 2009, Alphapharm 2007, Watson 2008, Forest 2011B (Vilazodone)	expansion

Vilazodone	Trimipramine	Yes-serotonin syndrome	Forest 2009, Forest 2011, Dista 2009, Alphapharm 2007, Watson 2008, Forest 2011B (Vilazodone)	expansion
Vilazodone	Tryptophan	Yes-serotonin syndrome	Forest 2009, Forest 2011, Dista 2009, Alphapharm 2007, Watson 2008, Forest 2011B (Vilazodone)	expansion
Vilazodone	VITAMIN K ANTAGONIST	Yes-bleeding	Forest 2009, Forest 2011, Dista 2009, Alphapharm 2007, Watson 2008, Forest 2011B	expansion
Vilazodone	Venlafaxine	Yes-serotonin syndrome	Forest 2009, Forest 2011, Dista 2009, Alphapharm 2007, Watson 2008, Forest 2011B (Vilazodone)	expansion
Vilazodone	Vilazodone	Yes-serotonin syndrome	Forest 2009, Forest 2011, Dista 2009, Alphapharm 2007, Watson 2008, Forest 2011B (Vilazodone)	expansion
Vilazodone	Warfarin	Yes-bleeding	Forest 2009, Forest 2011, Dista 2009, Alphapharm 2007, Watson 2008, Forest 2011B	expansion
